# Light-driven redox deracemization of indolines and tetrahydroquinolines using a photocatalyst coupled with chiral phosphoric acid†

**DOI:** 10.1039/d2sc06340a

**Published:** 2023-01-10

**Authors:** Qipeng Chen, Yuanli Zhu, Xujing Shi, Renfu Huang, Chuang Jiang, Kun Zhang, Guohua Liu

**Affiliations:** a International Joint Laboratory on Resource Chemistry of Ministry of Education, Shanghai Key Laboratory of Rare Earth Functional Materials, Shanghai Normal University Shanghai 200234 P. R. China ghliu@shnu.edu.cn zhangkun@shnu.edu.cn

## Abstract

The integration of oxidation and enantioselective reduction enables a redox deracemization to directly access enantioenriched products from their corresponding racemates. However, the solution of the kinetically microscopic reversibility of substrates used in this oxidation/reduction unidirectional event is a great challenge. To address this issue, we have developed a light-driven strategy to enable an efficient redox deracemization of cyclamines. The method combines a photocatalyst and a chiral phosphoric acid in a toluene/aqueous cyclodextrin emulsion biphasic co-solvent system to drive the cascade out-of-equilibrium. Systemic optimizations achieve a feasible oxidation/reduction cascade sequence, and mechanistic investigations demonstrate a unidirectional process. This single-operation cascade route, which involves initial photocatalyzed oxidation of achiral cyclamines to cyclimines and subsequent chiral phosphoric acid-catalyzed enantioselective reduction of cyclimines to chiral cyclamines, is suitable for constructing optically pure indolines and tetrahydroquinolines.

## Abstract 2

A light-driven redox deracemization to prepare optically pure cyclamines from their racemates is developed. This reaction provides a wide range of chiral indolines and tetrahydroquinolines with high yields and enantioselectivities.

## Graphical abstract

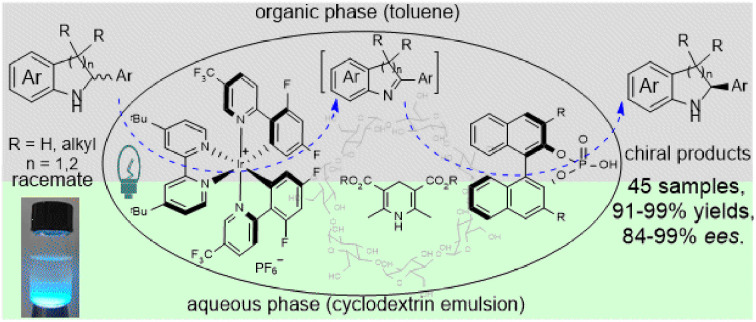

## 1. Introduction

**Fig. 1:**
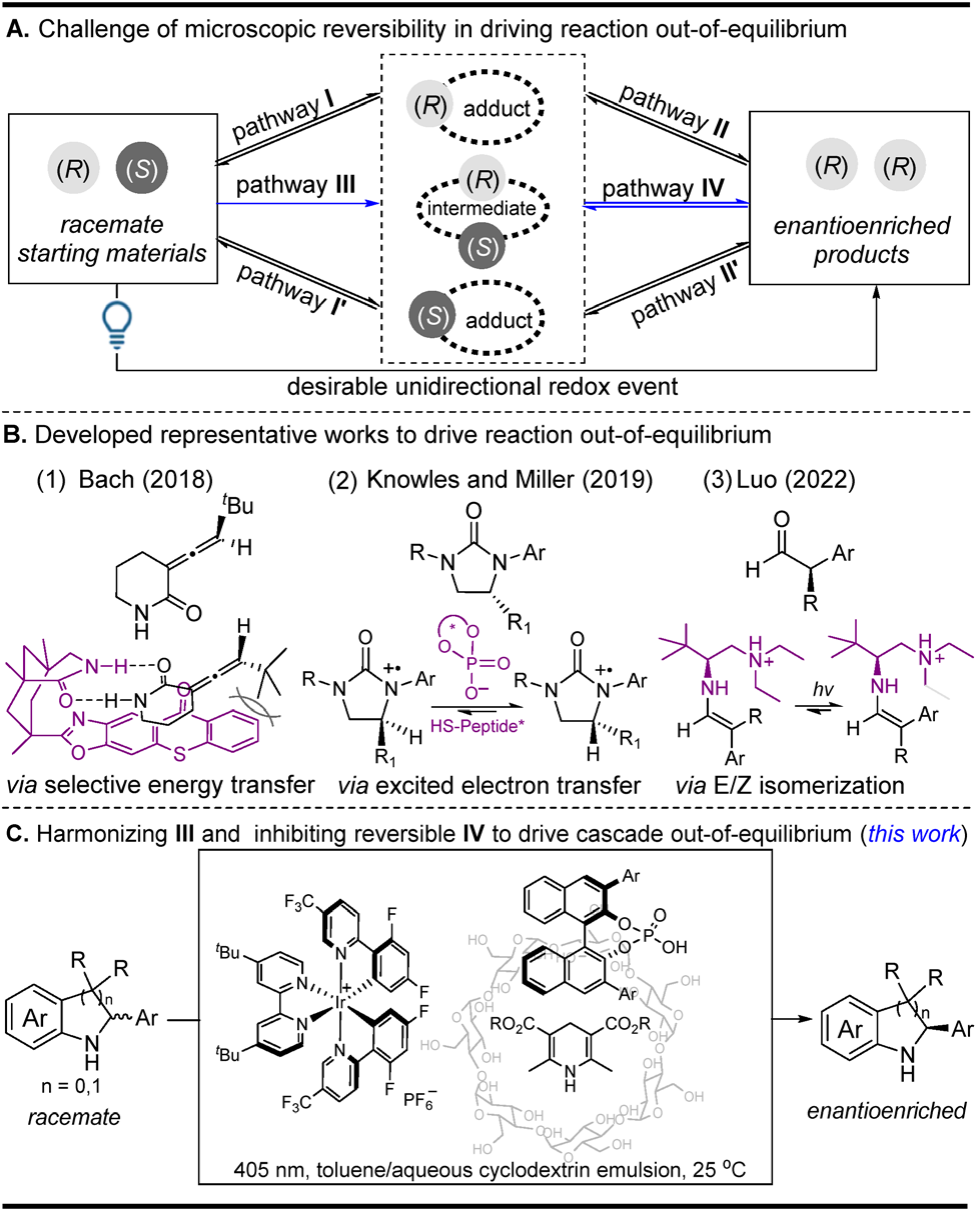
A schematic illustration of light-driven deracemization strategies: (A) the general challenge of kinetically microscopic reversibility in driving the reaction out-of-equilibrium, (B) three representative works that drive the reaction out-of-equilibrium, and (C) this work, which harmonizes pathway III and inhibits reversible pathway IV to drive the cascade out-of-equilibrium.

Enantioselective photocatalysis has grown into a well-established field, and a large number of works on integrating photocatalysts and chiral catalysts into co-catalyst systems have been summarized.^1^ Among these enantioselective photochemical reactions, light-driven deracemization has attracted a great deal of attention because optically pure products can be directly achieved from their corresponding racemates.^2–4^ Summarizing these deracemization reactions reveals that they mainly focus on selective energy transfer and excited-state electron transfer strategies, which are well known to solve the bottleneck problem of kinetically microscopic reversibility in driving reactions out-of-equilibrium when converting a pair of racemates into a single enantiomer (Fig. 1A). For the selective energy transfer strategy,^2^ the outstanding work reported by the Bach group (Fig. 1B_1_) uses a chiral sensitizer as a unidirectional catalyst to control the different energy-transfer efficiencies of two enantiomers, thereby driving the reaction out-of-equilibrium (pathway I *via* the inhibition of unfavorable I′ in the first step in Fig. 1A (racemization *via* triplet energy transfer)).^2*a*^ For the excited-state electron transfer strategy,^3^ the representative work reported by Knowles and Miller and co-workers (Fig. 1B_2_) utilizes two distinct chiral catalysts to mediate the two independent enantioselective steps, where the two *in situ* generated radical cations can be differentiated using a single chiral catalyst because of the different energy differences (pathway II *via* the inhibition of unfavorable II′ in the second step in Fig. 1A (racemization *via* a photoredox reductive quenching cycle)).^3*a*^ Very recently, the Luo group developed an elegant *E*/*Z* isomerization strategy to drive the reaction out-of-equilibrium owing to the energy differences of the *E*/*Z* adducts (Fig. 1B_3_) (pathways I and II *via* the inhibition of unfavorable I′ and II′ in the two steps in Fig. 1A (racemization *via* an *E*/*Z* isomerization)), where α-branched aldehydes can be directly converted into enantiopure α-tertiary carbonyls.^4^ Despite their effectiveness, the three typical strategies still have some drawbacks because they heavily depend on the energy differences of two chiral adducts (or intermediates) in a unidirectional redox event. The selective energy transfer strategy is substrate-specific since only substrates themselves that can undergo a configuration switch upon triplet-sensitized excitation are suitable in the deracemization reaction. In the excited-state electron transfer and the *E*/*Z* isomerization strategy, they are often used to differentiate two chiral adducts (or intermediates) with different energy differences in overcoming the kinetically microscopic reversibility. Therefore, the development of a new light-induced method used to achieve the efficient deracemization of those challenging intermediates with the same energy difference may nicely complement the methodological deficiency and will greatly extend the applied scope of the racemates in a single operation.

Optically pure amines as important industrial chemicals have extensive applications in the pharmaceutical and fine chemical industries.^5^ Recently, traditional thermochemical deracemizations used for the synthesis of chiral amines have also achieved great development.^6^ However, direct preparation of those chiral cyclamines, such as indolines and tetrahydroquinolines, is still an unmet challenge. To date, only two examples, biological deracemization and thermochemical deracemization, have been used for their construction through a two-step sequential process.^7^ In the former, a co-enzyme system through the combination of an amine oxidase and an imine reductase can convert racemates into chiral pyrrolidines and tetrahydroisoquinolines.^7*a*,*b*^ In the latter, an initial achiral catalyst-mediated oxidation followed by a chiral phosphoric acid-catalyzed reduction process has been used for the synthesis of chiral indolines, tetrahydropyrano-[3,4-*b*]indoles, and dihydropyrimidines.^7*c*–*e*^ Inspired by the prominent superiority of enantioselective photocatalysis,^2–4^ the development of a light-driven redox deracemization to directly prepare these chiral indolines and tetrahydroquinolines is highly desirable.

On the basis of recent achievements in the photocatalytic oxidation of cyclamines to cyclimines, together with the well-known electron transfer and proton transfer as tandem common steps,^8^ we hypothesize that a light-driven redox process may convert the *in situ* generated cyclimines to directly access the chiral products in a single operation. During this transformation, it is obligatory that the H-atom transfer reagent (Hantzsch ester (HTE)) in a normal enantioselective reduction simultaneously works as an electron transfer reagent. The key point lies in the competitive match of the bifunctionality of HTE as an electron transfer and H-atom transfer reagent, which not only needs the inhibition of the inverse photooxidation of the product (reversible pathway IV in Fig. 1A) but also needs the control of the expected photooxidation of the substrate (pathway III in Fig. 1A). As presented in this study, through the exploration of a toluene/aqueous cyclodextrin emulsion biphasic system in the manipulation of the balance of Hantzsch ester as a H-atom transfer reagent and electron acceptor, this single-operation redox deracemization cascade, involving initial photocatalyzed oxidation followed by subsequent chiral phosphoric acid-catalyzed reduction, enables the cascade to be driven out-of-equilibrium and to directly access the optically pure indolines and tetrahydroquinolines (Fig. 1C).

## 2. Experimental

### 2.1. General procedure for photocatalytic oxidation

A typical procedure was as follows. In an open-to-air test tube, to a suspension of aqueous cyclodextrin emulsion (1.0 mL) was added slowly a solution of photocatalyst (2.0 mol%), racemic indolines (0.10 mmol), and additives in 1.0 mL of toluene at room temperature. The resulting mixture under light irradiation was then stirred at 25 °C for the first 1–5 h. Upon completion, the organic layers were collected and the aqueous solution was extracted with toluene (3 × 1.0 mL). After evaporation of the solvent, the resulting residue was purified by silica gel flash column chromatography to afford the pure oxidation products.

### 2.2. General procedure for redox deracemization

A typical procedure was as follows. In an open-to-air test tube, to a suspension of Hantzsch ester (3.0 equiv.) in 1.0 mL of aqueous cyclodextrin emulsion was added slowly a solution of photocatalyst (2.0 mol%), chiral phosphoric acid (10.0 mol%) and racemic indolines or tetrahydroquinolines (0.10 mmol) in 1.0 mL of toluene at room temperature. The resulting mixture under light irradiation was then stirred at 25 °C for the first 2.5 h. Upon completion, the organic layers were collected and the aqueous solution was extracted with toluene (3 × 1.0 mL). After evaporation of the solvent, the resulting residue was purified by silica gel flash column chromatography to afford the chiral products. The ee values were determined using an HPLC analysis with a UV-Vis detector and a Daicel Chiral-Cel column (*Φ* 0.46 × 25 cm).

## 3. Results and discussion

### 3.1. Design of a biphasic system used for a light-driven redox deracemization

**Fig. 2:**
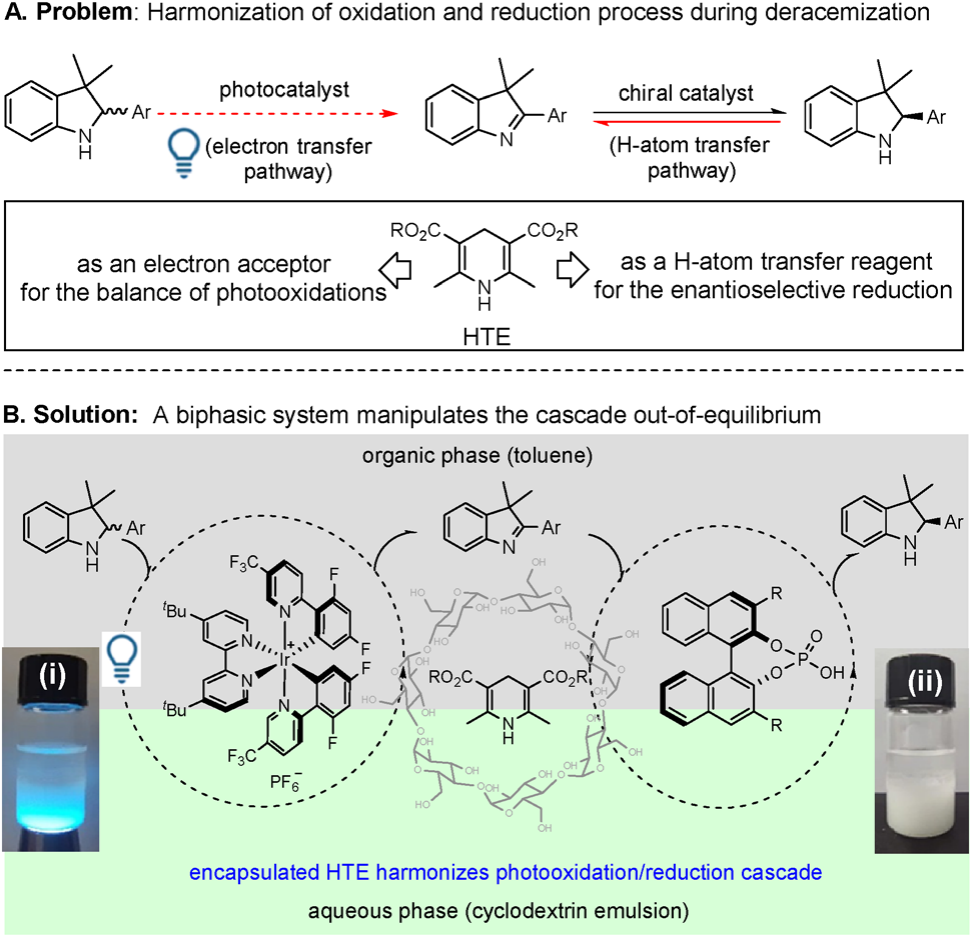
A schematic illustration of the design of the redox deracemization of indolines: (A) problem and (B) solution; (i and ii) a mixture of toluene and aqueous β-cyclodextrin emulsion containing HTE with and without light irradiation.

The design of a desirable oxidation/reduction cascade in a unidirectional event must match the photooxidation and enantioselective reduction reactions (*e.g.* the use of deracemization of indolines, as shown in Fig. 2). In the photocatalytic oxidation step, the photocatalysts excited under visible-light irradiation *via* a single electron transfer (SET) process are well known to convert racemic cyclamines into cyclimines.^8^ However, the problem lies in that the unmatched light energy also leads to excessive oxidation of the chiral products (chiral cyclamines). Therefore, irradiating this cascade with a suitable light source is obligatory. In the chiral phosphoric acid-catalyzed reduction step, the stoichiometric HTE acts as a reductant for the H-atom transfer and is needed to promote the enantioselective reduction. However, the HTE itself is also an electron acceptor, which can restrain and/or terminate the first-step photocatalytic oxidation of cyclamines.^8*e*^ Therefore, harmonization of the competition of HTE and cyclamine (substrate) as electron acceptors is essential. Accordingly, in this oxidation/reduction unidirectional process, the manipulation of the bifunctionality of HTE both in the competitive photooxidation of the substrates (red dotted arrow) and in the inhibition of the reversible pathway of the products (red solid arrow) is the key problem in driving the cascade out-of-equilibrium (Fig. 2A).

To overcome the above problems, we have provided a solution through the use of a toluene/aqueous cyclodextrin emulsion biphasic reaction system (Fig. 2B), in which a photocatalyst and a chiral phosphoric acid catalyst work together based on three considerations in this redox deracemization of indolines. First, the selection of the chiral phosphoric acid (CPA) as an asymmetric transfer hydrogenation catalyst is attributed to the fact that CPA can assist photooxidation to promote catalytic transformations,^9^ which is a prerequisite because the compatibility eliminates the mutual deactivation between the Ir-based photocatalyst and CPA. Second, the utilization of the aqueous cyclodextrin emulsion as one of the co-solvents lies in the cone-like morphology of cyclodextrin (composed of a hydrophobic central cavity and a hydrophilic outer surface), which can not only produce long-term stable emulsions but also encapsulates HTE molecules within its cavity. Such a selection is beneficial to control the release of HTE,^10^ thereby providing an opportunity to balance the functions of HTE as an H-atom transfer reagent and/or an electron acceptor. Thirdly, the design of a biphasic co-solvent system (toluene/aqueous β-cyclodextrin emulsion) can manipulate the competition between the substrate and HTE as electron acceptors. Due to the poor solubility of HTE both in toluene and in water, we entrap HTE into the cavity of cyclodextrin, making the photocatalyzed indoline oxidation occur in the upper toluene phase and the CPA-catalyzed enantioselective reduction proceed in the lower aqueous phase. If the release of HTE could be controlled at a suitable concentration in the biphasic interface, HTE will not interfere with the photooxidation of the substrates in the upper toluene phase. More importantly, the suitable release of HTE can provide a shield-like layer to replace the chiral products as an electron acceptor in the elimination of the excessive photooxidation of the *in situ* generated chiral product (the lower oxidative potential of HTE is lower than those of substrates^8*b*,*c*,11^) as the CPA-catalyzed enantioselective reduction proceeds. In particular, when the photooxidation rate of the racemate in the upper toluene phase is much faster than the photooxidation rate of the chiral product in the lower aqueous phase, this will drive the cascade out-of-equilibrium to allow the desirable unidirectional event to occur. As we design in the experiments, the saturated aqueous β-cyclodextrin presents a long-term stable emulsion, which can encapsulate HTE to form a suspension in the lower aqueous phase with and without the light irradiation ((i) and (ii) in Fig. 2B). The clear biphasic layers could be observed, in which the catalysts and substrate dissolve in the upper toluene phase and HTE is suspended in the corresponding lower aqueous phase. As presented in this single-operation deracemization process, the suitable release of HTE in the lower aqueous phase on the competition between the substrate and product, together with the different photooxidation rates of substrate/products under biphasic conditions, efficiently manipulates the driving force to overcome the problem of the microscopic reversibility.

### 3.2. Optimization investigations

With the desirable deracemization of indolines *via* a combination of a photocatalyst and CPA in mind, we initially optimized the single-step oxidations by screening different light irradiation wavelengths in a mixture of toluene and cyclodextrin emulsion (CE) with a volume ratio of one to one (v/v = 1/1). During this transformation, 3,3-dimethyl-2-phenylindoline (*rac*-1a) was chosen as a model substrate to examine the differentiation of the photocatalysts and relevant additives, as shown in Table 1. It was found that the model reaction catalyzed by A under visible light irradiation (*λ*_exc_ ≤ 405 nm) could reach completion within 2 h, which was better than that under the other conditions (*λ*_exc_ > 405 nm), when catalyzed by its analog (B) and the control reaction (entries 1–3 *versus* entries 4–9, Table 1). Therefore, light irradiation at *λ*_exc_ of 405 nm was chosen as the suitable light source.

**Table 1:** Optimization of the catalysts, light sources, and additives in the single-step oxidation of *rac*-1a^a^

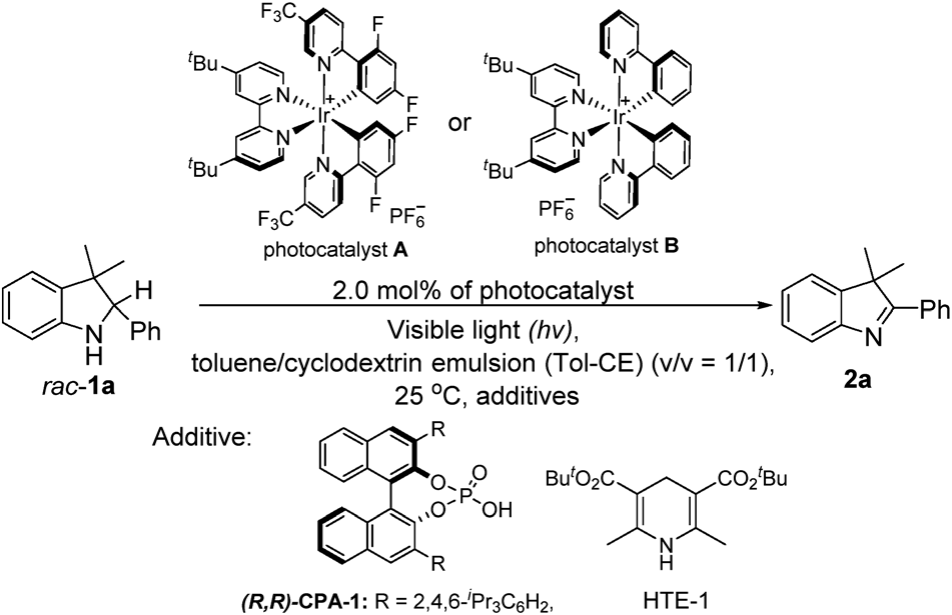			
Entry	Catalyst/solvent/additive/conditions/*hv* (nm)	Time (h)	% yield^b^
1	A/Tol-β-CE/—/air/365	2.0	>99
2	A/Tol-β-CE/—/air/395	2.0	>99
3	A/Tol-β-CE/—/air/405	2.0	99
4	B/Tol-β-CE/—/air/405	2.0	78
5	A/Tol-β-CE/—/air/420	2.0	87
6	A/Tol-β-CE/—/air/455	5.0	74
7	A/Tol-β-CE/—/air/470	5.0	54
8	A/Tol-β-CE/—/air/500	5.0	25
9	A/Tol-β-CE/—/air/405	5.0	8
10	A/Tol-β-CE/CPA-1 (10%)/air/405	2.0	99
11	A/Tol-β-CE/CPA-1 (10%)/N_2_/405	2.0	99
12	A/Tol-β-CE/CPA-1 (10%)/dark/405	2.0	ND
13	A/Tol-β-CE/CPA-1 (8%)/air/405	2.0	96
14	A/Tol-β-CE/CPA-1 (12%)/air/405	2.0	98
15	A/Tol-β-CE/HTE-1 (2.0 equiv.)/air/405	2.0	99
16	A/Tol-β-CE/HTE-1 (3.0 equiv.)/air/405	2.0	94
17	A/Tol-β-CE/HTE-1 (2.0 equiv.) in toluene/air/405	2.0	65
18	A/Tol-α-CE/HTE-1 (2.0 equiv.)/air/405	2.0	92
19	A/Tol-γ-CE/HTE-1 (2.0 equiv.)/air/405	2.0	95
20	A/Tol–water/HTE-1 (2.0 equiv.)/air/405	2.0	92

a Reaction conditions: to a suspension of aqueous cyclodextrin emulsion with/without HTE-1 (1.0 mL) was added slowly a solution of A or B (2.0 mol%), *rac*-1a (0.10 mmol), and/or CPA-1, and/or HTE-1 in 1.0 mL of toluene at room temperature with stirring at 400 rpm under light irradiation at 25 °C.

b Yields were determined by ^1^H-NMR analysis. ND = not detected. Tol = toluene. CE = cyclodextrin emulsion.

To consolidate the compatibility between the photocatalyst and CPA, the model reaction of *rac*-1a under light irradiation in the presence of 10.0 mol% CPA-1 was also investigated. The result showed that a slightly enhanced yield was achieved, confirming their compatibility (entry 10 *versus* entry 3, Table 1).^12^ Furthermore, the model reaction conducted under a nitrogen atmosphere still afforded a similar yield, whereas that under dark conditions did not occur, demonstrating that the reaction was a light-driven oxidation process and oxygen did not participate in the transformation (entry 10 *versus* entries 11 and 12, Table 1). Further optimization of the amounts revealed that 10.0 mol% of CPA-1 was the optimal amount during the photocatalytic oxidation process (entry 10 *versus* entries 13 and 14, Table 1).

Subsequently, to explore the function of the biphasic co-solvent system in balancing the competition between the substrate (*rac*-1a) and HTE-1, the model reaction in the presence of HTE-1 under light irradiation was also investigated. It was found that the suitable concentrations of HTE-1 in 1.0 mL of the aqueous β-cyclodextrin emulsion varied in the range of 0–2.0 equivalents relative to the mole amount of *rac*-1a, and the yield slightly decreased when the concentration was increased to 3 equivalents (entry 15 *versus* entry 16, Table 1). This result was markedly better than that obtained in the toluene/aqueous emulsion (v/v = 1/1) with the HTE-1 directly suspended in the upper toluene (entry 15 *versus* entry 17, Table 1), and slightly higher than those obtained in the toluene/aqueous α-cyclodextrin emulsion (entry 15 *versus* entry 18, Table 1), toluene/aqueous γ-cyclodextrin emulsion (entry 15 *versus* entry 19, Table 1), and toluene/water systems (entry 15 *versus* entry 20, Table 1). In the former, this comparison indicates that the HTE-1 as an electron acceptor could inhibit the photooxidation of *rac*-1a. In the latter, these observations suggest that the aqueous β-cyclodextrin emulsion can provide the best suitable release of HTE to eliminate the effect on the photooxidation.

Finally, we integrated two single-step reactions into a cascade process using the deuterated *rac*-1a-*d* as a substrate because the deuterium erosion could be calculated for the determination of the ^1^H-NMR yield of the chiral product ((*R*)-1a). In this case, the reaction was performed in the presence of 3.0 equivalents of HTE since more than 2.0 equivalents of HTEs were needed to promote the H-atom transfer in the single-step enantioselective reduction. As shown in Table 2, we first optimized the solvents and found that the model reaction under the only toluene conditions mainly produced intermediate 2a without (*R*)-1a, whereas the reaction conducted under the only aqueous β-cyclodextrin emulsion conditions could provide (*R*)-1a in a 15% yield with 19% ee (entry 2, Table 2). Interestingly, the model reaction carried out under toluene/aqueous β-cyclodextrin emulsion (v/v = 1/1) conditions produced (*R*)-1a in a 99% yield with 98% ee (entry 3, Table 2). Further comparisons of the other Hantzsch esters disclosed that HTE-1 was the best H-atom transfer reagent (entries 3–6, Table 2). Optimizations of the co-solvents then confirmed that the toluene/β-cyclodextrin emulsion (v/v = 1/1) was the best co-solvent system (entry 3 *versus* entries 7 and 8, Table 2). Finally, upon screening the chiral phosphoric acids,^13^ CPA-1 was determined to be the best ATH catalyst used in this redox deracemization due to the highest ee value (entry 3 *versus* entries 9 and 10, Table 2), in which the dispersion interactions may play a role in explaining the superiority of CPA-1.^13*e*^

**Table 2:** Optimization of the redox deracemization of *rac*-1a-*d* in a single operation^a^

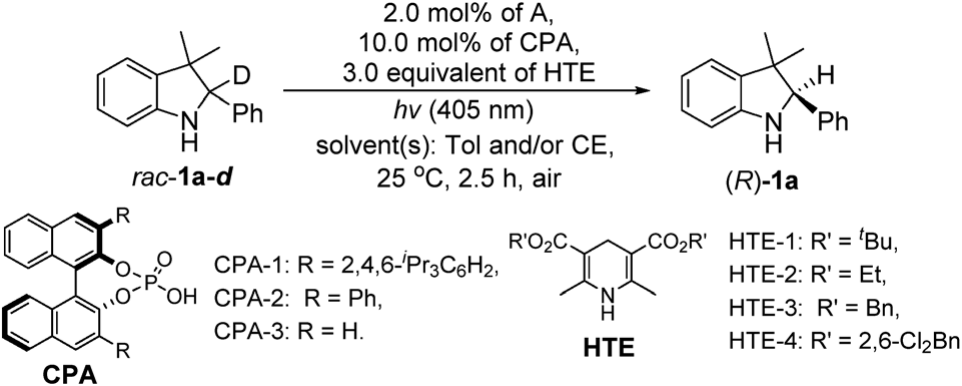				
Entry	CPA/HTE	Solvent(s)	% yield^b^	% ee^b^
1	CPA-1/HTE-1	Tol	ND	—
2	CPA-1/HTE-1	β-CE	15	19
3	CPA-1/HTE-1	Tol-β-CE (1/1)	99	98
4	CPA-1/HTE-2	Tol-β-CE (1/1)	92	85
5	CPA-1/HTE-3	Tol-β-CE (1/1)	99	90
6	CPA-1/HTE-4	Tol-β-CE (1/1)	99	93
7	CPA-1/HTE-1	Tol-β-CE (3/2)	95	95
8	CPA-1/HTE-1	Tol-β-CE (2/3)	99	94
9	CPA-2/HTE-1	Tol-β-CE (1/1)	97	15
10	CPA-3/HTE-1	Tol-β-CE (1/1)	99	6

a Reaction conditions: to a suspension of HTE (3.0 equiv.) in 1.0 mL of solvent(s) (1.0 mL) was added slowly a solution of A (2.0 mol%), chiral CPA (10.0 mol%), and *rac*-1-*d* (0.10 mmol) in 1.0 mL of toluene at room temperature with stirring at 400 rpm under light irradiation conditions (*λ* = 405 nm) at 25 °C for 2.5 h. ND = not detected. Tol = toluene. CE = cyclodextrin emulsion.

b Yields were the ^1^H-NMR yields calculated from the percentage of deuterium erosion and ee values were determined by chiral HPLC analysis (see ESI Fig. S1).

### 3.3. Mechanistic studies

**Scheme 1:**
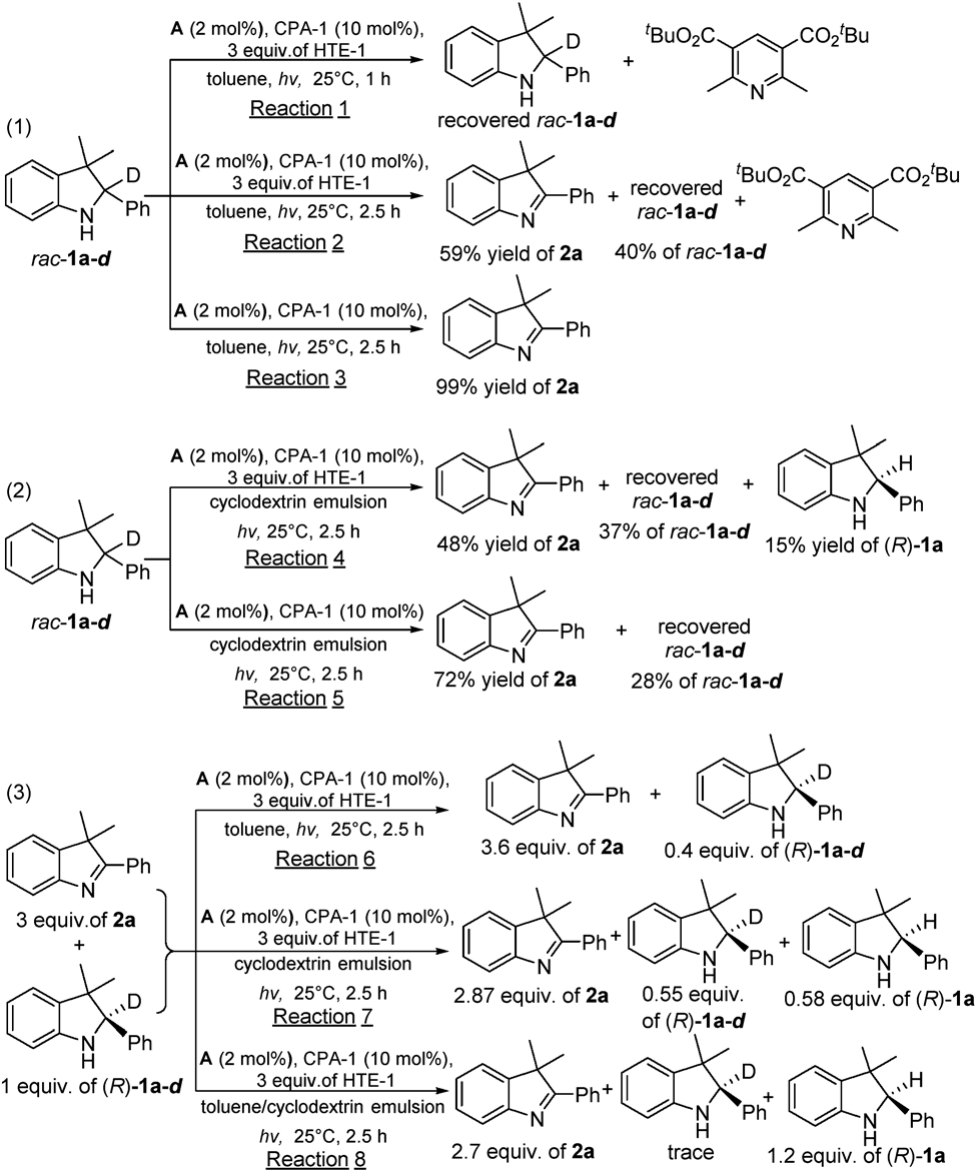
The investigation of the deuterium labeling experiments.

To provide insight into the driving force for the cascade out-of-equilibrium process in this biphasic system, a series of control experiments under light irradiation (*λ* = 405 nm) conditions were performed, as shown in Scheme 1. In the first type of control experiment using only toluene as the single solvent (eqn (1) in Scheme 1), reaction (1) of *rac*-1a-*d* in 1 h mainly produced the byproduct of di-*tert*-butyl 2,6-diisobutylpyridine-3,5-dicarboxylate (the final oxidative byproduct of HTE-1) with the completely recovered *rac*-1a-*d*, demonstrating that HTE-1 restrained the photooxidation of *rac*-1a-*d* because of the low oxidation potential of HTE-1 relative to *rac*-1a-*d*. Monitoring reaction (2) for a further 1.5 h showed that the photooxidation of *rac*-1a-*d* could occur and gave 2a in a 59% yield with the concomitant disappearance of HTE-1. The absence of final product (*R*)-1a in entry 1 of Table 2 should be attributed to the fact that HTE-1 had been consumed, which is proven by the complete transformation of di-*tert*-butyl 2,6-diisobutylpyridine-3,5-dicarboxylate. As a result, the second enantioselective reduction step did not occur because there was no HTE-1 for the H-atom transfer process. Through the comparison of 99% yield in reaction (3) without HTE-1, these control experiments suggested that HTE-1 in toluene mainly acted as an electron acceptor rather than an H-atom transfer reagent. In the second type of control experiment using only aqueous β-cyclodextrin emulsion as the single solvent (eqn (2) in Scheme 1), reaction (4) could produce (*R*)-1a, but it was a mixture of *rac*-1a-*d*, 2a, and (*R*)-1a (entry 2 in Table 2). This control experiment revealed that HTE-1 in the aqueous phase could simultaneously work as an electron acceptor and H-atom transfer reagent. For comparison, the control reaction (5) performed without HTE-1 exhibited an enhancement of the yield of 2a from 48 to 72%. However, the low yields of 2a in both reactions (4) and (5) relative to the complete inhibition in reaction (1) disclosed that HTE-1 inhibited the photocatalytic oxidation of *rac*-1a-*d* to a certain extent rather than completely in the only aqueous β-cyclodextrin emulsion. The above two types of comparisons suggest that there is a fast and complete inhibition in the toluene phase, and a slow and partial inhibition in the aqueous phase, thereby disclosing the feasibility of HTE-1 in the manipulation of the competitive photooxidation of the substrate/product.

To consolidate the different inhibitory behaviors imposed on the photocatalytic oxidation of the *in situ* generated chiral product and free *rac*-1a-*d* substrate, in the third type of control experiment, we also investigated the single-step enantioselective reactions of a mixture of 2a (3 equivalents) and (*R*)-1a-*d* (1 equivalent) under different solvent conditions (eqn (3) in Scheme 1). It was found that in reaction (6) in toluene 0.40 equivalents of (*R*)-1a-*d* still remained without any (*R*)-1a, which meant that the 0.60 equivalents of free (*R*)-1a-*d* could be converted into 2a in toluene, which was consistent with the result attained with reaction (2). Meanwhile, reaction (7) in aqueous β-cyclodextrin emulsion could give 0.58 equivalents of (*R*)-1a with the concomitant residual (*R*)-1a-*d* (0.55 equivalents) and unreacted 2a (2.87 equivalents). This observation demonstrated that 0.45 equivalents of free (*R*)-1a-*d* could be photooxidized to 2a, whereas only a small amount of *in situ* generated (*R*)-1a (0.02 equivalents relative to reaction (4)) was photooxidized owing to the residual 0.13 equivalents of (*R*)-1a. This comparison demonstrated that there is a different inhibitory behavior in toluene and aqueous β-cyclodextrin emulsion. To our delight, reaction (8) in the design of toluene/aqueous β-cyclodextrin emulsion biphasic conditions could provide 1.20 equivalents of (*R*)-1a with the concomitant trace (*R*)-1a-*d* and unreacted 2a, confirming that the encapsulated HTE-1 could efficiently inhibit photooxidation of the *in situ* generated chiral product. Through these investigations, their different inhibitory behaviors imposed on the photooxidation of the substrate and/or the *in situ* generated chiral product could drive the cascade out-of-equilibrium, thereby enabling an efficient redox deracemization process.

**Fig. 3:**
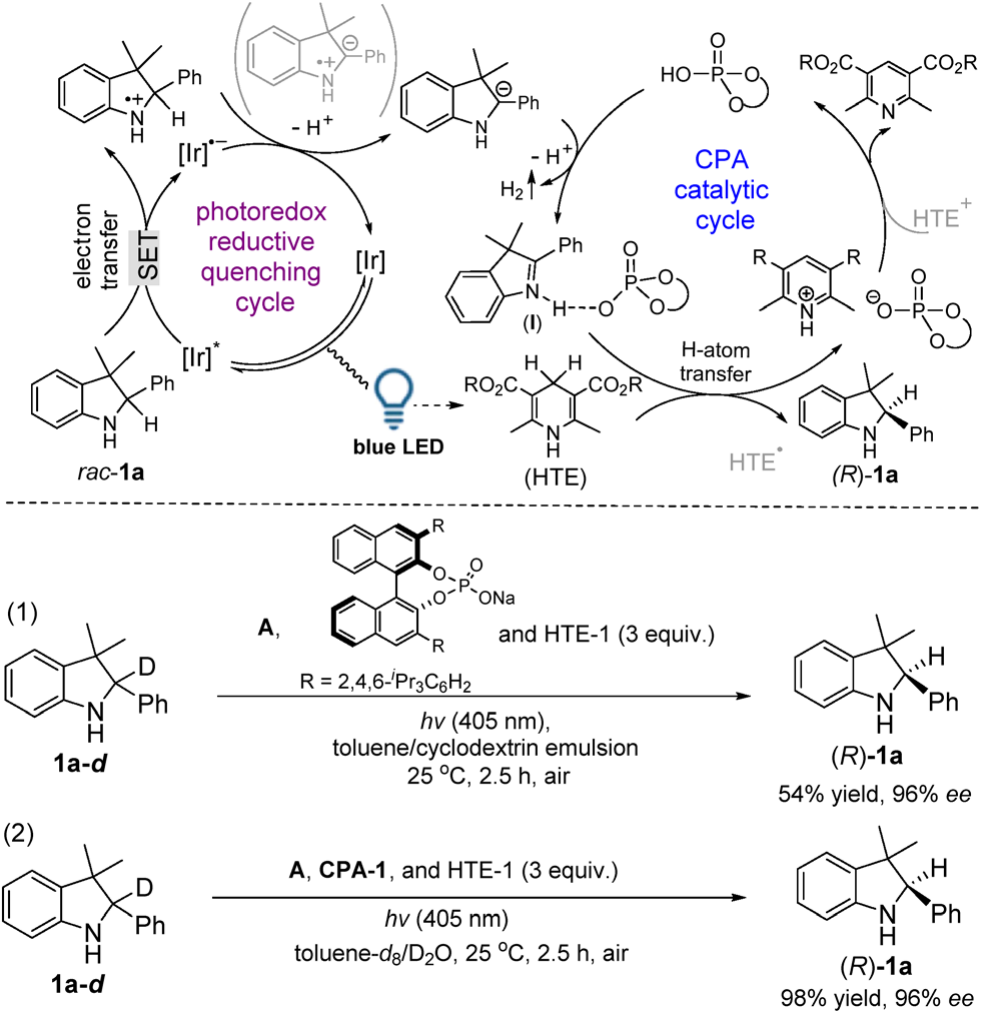
A proposed light-driven deracemization route.

Based on our deuterium labeling control experiments, a plausible cascade route, an initial photoredox reductive quenching cycle followed by a CPA catalytic cycle, was proposed as shown in Fig. 3. In a photoredox reductive quenching cycle occurring *via* a SET pathway,^12*a*,*b*^ the light irradiation produces an excited state [Ir]* and the [Ir]* is then reductively quenched to form [Ir]˙^−^, which transfers the radical anion for the regeneration of the photocatalyst to complete the first step of the catalytic cycle. During this cycle, *rac*-1a goes through a SET process to give a sulfonamide N-radical cation, which was further reduced by [Ir]˙^−^ with the concomitance of loss of a proton to lead to a benzylic carbanion of *rac*-1a. By *in situ* GC analysis, the detection of H_2_ suggested the release of two protons and two electrons from the catalytic system (see ESI Fig. S2†).^8*b*,*c*,12*e*,*f*^ In a CPA catalytic cycle, the protonation (acidic activation) of 2a by CPA-1 initially forms a chiral ion pair (I), in which an interaction between 2a and CPA-1 could be observed by using a ^1^H-NMR analysis in the mixed spectra of 2a and CPA-1 (see ESI Fig. S3†). The control experiment also confirmed the acidic activation step because the redox deracemization of *rac*-1a-*d* catalyzed by the salt of CPA-1 only gave (*R*)-1a in 54% yield (eqn (1) in Fig. 3). Subsequently, an H-atom transfer from HTE-1 gives a chiral product (*R*)-1a. The parallel deuterium labeling experiment in toluene-*d*_8_/D_2_O (v/v = 1/1) demonstrated that the hydrogen comes from HTE-1 rather than the solvent (H_2_O) since the chiral product of (*R*)-1a was not incorporated with deuterium (eqn (2) in Fig. 3). Finally, the chiral phosphate acid is regenerated after the release of the final oxidative adduct of HTE-1 *via* an HTE/HTE˙ to HTE˙^+^/HTE^+^ pathway,^12*c*,*d*^ performing the second step of the catalytic cycle.^14^ Through the integration of two catalytic cycles, it is proposed that a cascade pathway consisting of photocatalytic oxidation followed by enantioselective reduction occurs during this redox deracemization.

### 3.4. Scope of the substrates

**Scheme 2:**
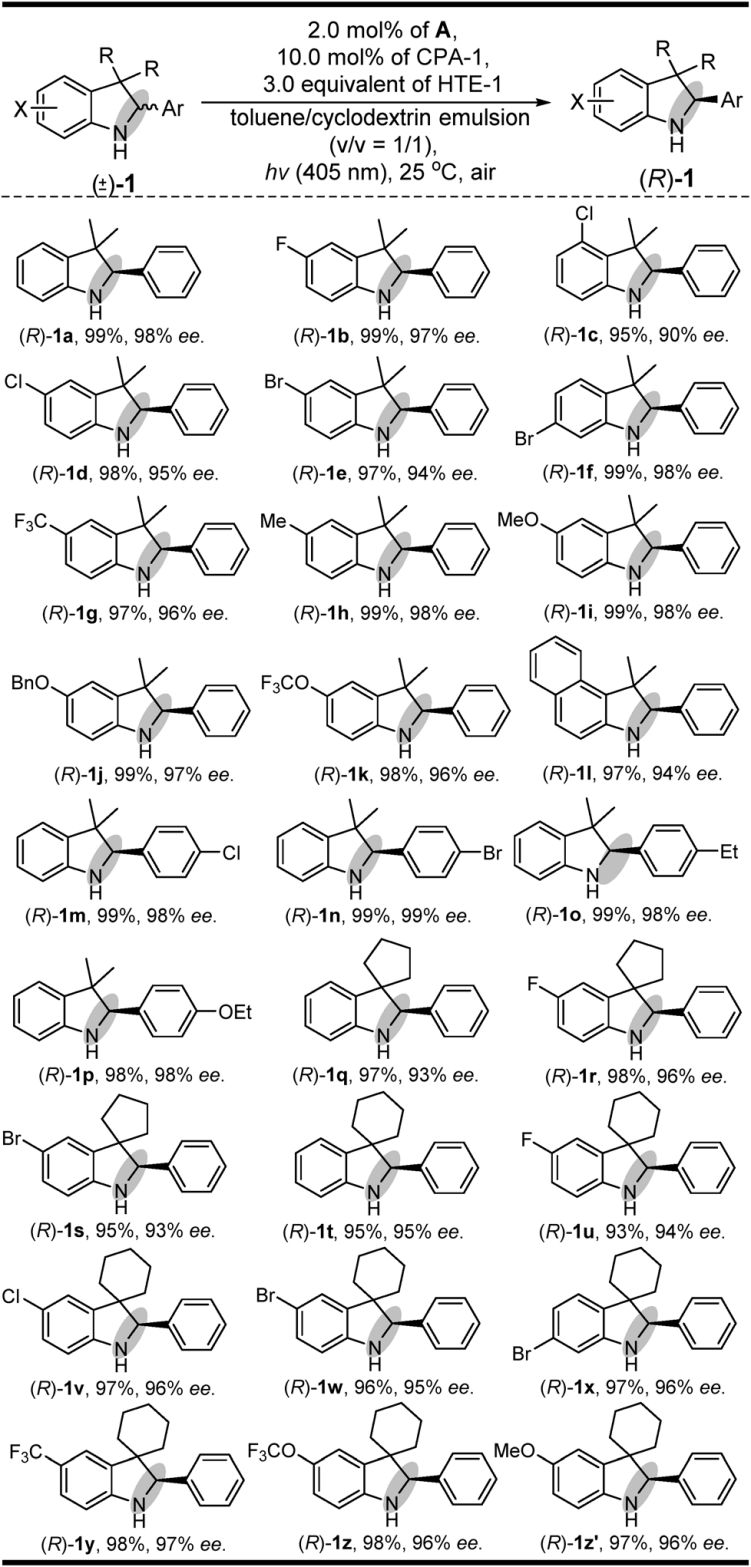
The substrate scope of indolines.

Having established a clear light-driven redox deracemization, a series of indoline substrates were further investigated, as shown in Scheme 2 (see ESI Fig. S4 and S5†). The results showed that all of the racemic 3*H*-indolines studied could be steadily converted into their corresponding optically pure indolines in high yields and enantioselectivities, which are comparable to their single-step enantioselective reduction reactions.^14*c*^ It was also found that 3,3-dimethyl substituted 3*H*-indolines with various substitution patterns were well tolerated under the same single-operation deracemization conditions, whereas those bearing different electron-withdrawing and -donating substituents at the X substitution and/or Ar group were equally efficient in terms of their yields and enantioselectivities ((*R*)-1b–(*R*)-1l and (*R*)-1m–(*R*)-1p). It was also notable that the spirocyclic products, either with five-membered substituents at the 3,3-position ((*R*)-1q–(*R*)-1s) or six-membered ones ((*R*)-1t–(*R*)-1z′), were readily obtained in good yields and high enantiomeric excesses. Furthermore, this light-driven deracemization could also be used for the gram-scale preparation of chiral indolines, wherein 5.0 mmol (1.26 g) of *rac*-1i could be converted into (*R*)-1i in 98% yield (1.24 g) with maintainable enantioselectivity (98% ee).

**Scheme 3:**
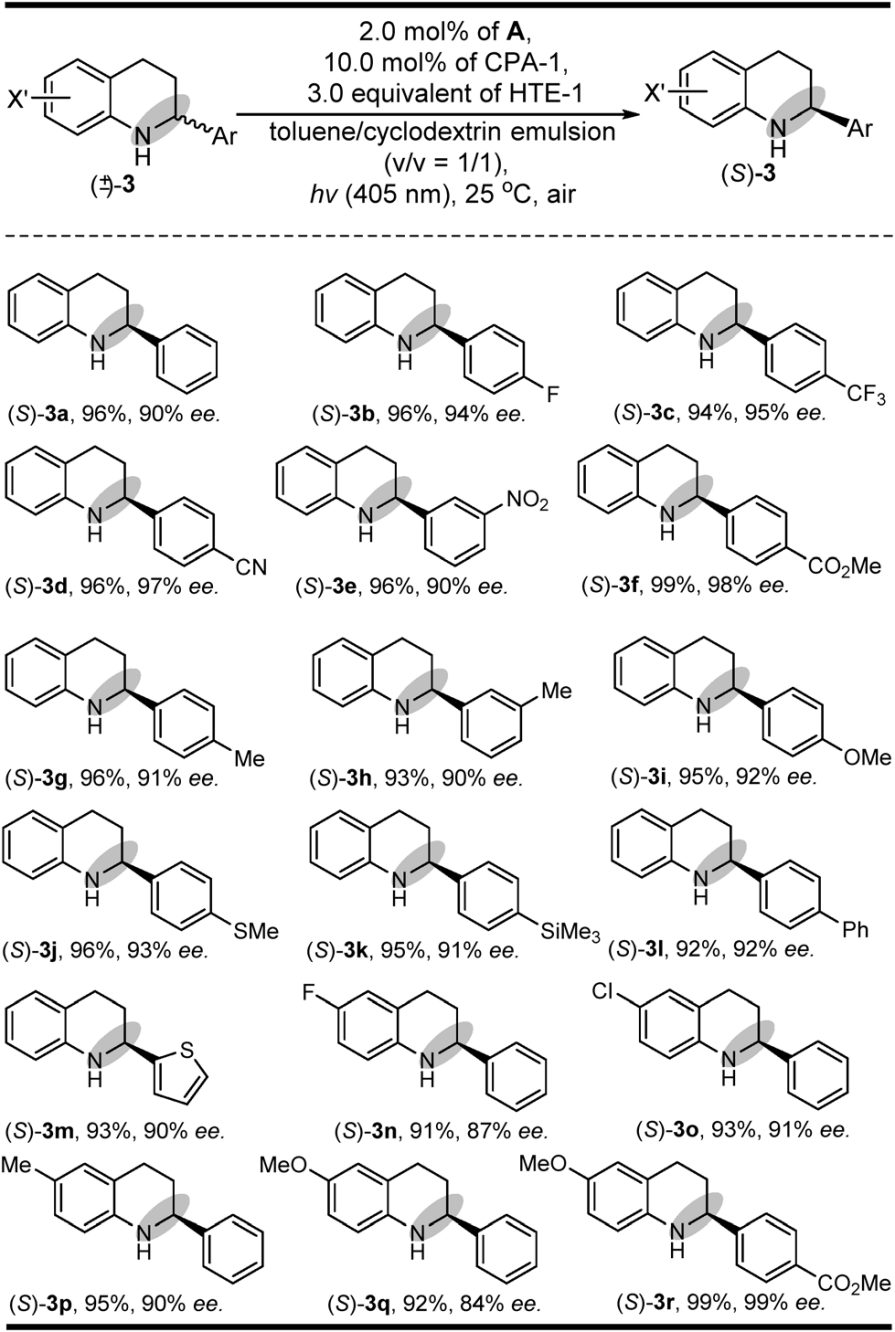
The substrate scope of tetrahydroquinolines.

In addition to its general feasibility in the redox deracemization of indolines (Scheme 2), this deracemization cascade process was also suitable for the construction of chiral tetrahydroquinolines,^10,15^ as shown in Scheme 3 (see ESI Fig. S4 and S5†). This light-driven oxidation/reduction cascade process using a series of tetrahydroquinolines as the substrates could produce their corresponding chiral tetrahydroquinolines in high yields and enantioselectivities although in generally lower enantioselectivities relative to those obtained with indolines, likely due to differing conformational flexibility of the α-amino radical intermediate.^16^ Also, the stereoelectronic properties of the substituents on the Ar group did not significantly affect their yields and enantioselectivities ((*S*)-3b–(*S*)-3l). Moreover, the quinolones with different X′ substituents proceeded well and could afford chiral tetrahydroquinolines ((*S*)-3m–(*S*)-3r) with high yields and enantioselectivities.

## 4. Conclusions

In conclusion, by combining a photocatalyst and chiral phosphoric acid as co-catalysts, we have exploited a simple light-driven redox deracemization of racemic indolines and tetrahydroquinolines. This toluene/aqueous cyclodextrin emulsion biphasic co-solvent system not only balances the functions of HTE as an H-atom transfer reagent and/or an electron acceptor but also manipulates the competition between the substrate and HTE as electron acceptors, providing a wide range of chiral indolines and tetrahydroquinolines in high yields and enantioselectivities from their corresponding racemates. Mechanistic investigations have disclosed an oxidation/reduction cascade pathway, where this route involves an initial photocatalyzed oxidation followed by a chiral phosphoric acid-catalyzed reduction that allows direct access to optically pure chiral cyclamines. Our study also provides a complementary methodology for light-driven redox deracemization to synthesize chiral indolines and tetrahydroquinolines for future applications.

## Data availability

The data that support the findings of this study are available in the main manuscript, the ESI† and also from the authors upon reasonable request.

## Author contributions

G. L. conceived the idea. Q. C., Y. Z., X. S., R. H., C. J., and K. Z. performed the experiments. Q. C., K. Z. and G. L. wrote the paper.

## Conflicts of interest

The authors declare no competing interests.

## Supplementary Material

SC-014-D2SC06340A-s001
