# Supplementary material for: Light-driven redox deracemization of indolines and tetrahydroquinolines using a photocatalyst coupled with chiral phosphoric acid
Source: Chem Sci. 2023 Jan 10;14(7):1715–23. doi: 10.1039/d2sc06340a (PMC9930931; doi:10.1039/d2sc06340a)

Electronic Supplementary Material (ESI) for Chemical Science.
This journal is © The Royal Society of Chemistry 2023

Supporting Information

Light-driven redox deracemization of indolines and tetrahydroquinolines using a photocatalyst coupled with chiral phosphoric acid

Qipeng Chen, Yuanli Zhu, Xujing Shi, Renfu Huang, Chuang Jiang, Kun Zhang,* and Guohua Liu*

Key Laboratory of Resource Chemistry of Ministry of Education, Shanghai Key Laboratory of Rare Earth Functional Materials, Shanghai Normal University, Shanghai, 200234, PR China. Email: ghliu@shnu.edu.cn, kunzhang@shnu.edu.cn

CONTENTS

Experimental. S2

Figure S1. ^{1}H–NMR and HPLC spectra of (*R*)-**1a** in the reactions of *rac*-**1a-*d₁*** in Table 2 .... S15

Figure S2. GC analysis of H_2 in the single-step oxidations of *rac*-**1a** in toluene. S25

Figure S3. ^{1}H-NMR spectra of **2a**, CPA-1, and **2a**+CPA (mole ratio = 1:1) in $CDCl_3$.......... S26

Figure S4. HPLC analyses of chiral products S27

Figure S5. Characterization of chiral products S72

Experimental

1. General:

All manipulations were carried out under an inert atmosphere using a nitrogen-filled glovebox or Schlenk techniques. Deuterated solvents were purchased commercially and were degassed and stored over activated 4 Å molecular sieves. Photocatalysts, chiral phosphoric acids ((*R*,*R*)-CPA-1-(*R*,*R*)-CPA-4), and α-cyclodextrin, β-cyclodextrin, γ-cyclodextrin were purchased from Sigma−Aldrich Company Ltd and used as received. The racemic indolines or tetrahydroquinolines were prepared according to the published procedures (*J. Am. Chem. Soc.* **2013**, *135*, 14090-14093). All other reagents were obtained from commercial sources and used without further purification. The ^{1}H NMR spectra were performed on a Bruker Avance DPX-400 spectrometer in $CDCl_3$ solutions. Chemicals shifts are given in parts per million (δ units) downfield from tetramethylsilane using the residual solvent signal ($CHCl_3$, δ 7.26) as an internal standard. ^{1}H NMR information is given in the following format: multiplicity (s, singlet; d, doublet; t, triplet; q, quartet; qui, quintet; sept, septet; m, multiplet), coupling constant(s) (J) in Hertz (Hz), the number of protons. The prefix app is occasionally applied when the true signal multiplicity was unresolved and br indicates the signal in question broadened. ^{13}C{1H} NMR spectra are reported in ppm (δ) relative to residual $CHCl_3$ (δ 77.36) unless otherwise noted. The enantiomeric excesses (*ee*) were determined using a Daicel Chiralcel column IC–H or OD-H or AD-H with the above HPLC setup.

2. General procedure for the synthesis of starting materials.

2.1 General procedure for the synthesis of racemic indolines:

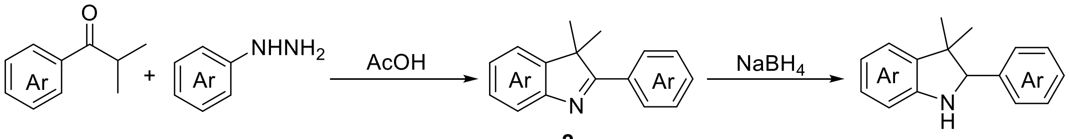

2

rac-**1**

General procedure for the synthesis of **2:** In a typical synthetic route, the ketone (2.0 mmol, 1.0 eq) and arylhydrazine (3.0 mmol, 1.50 eq) were suspended in 10.0 mL of glacial acetic acid at 25 ℃ and the resulting mixture was heated to 120 °C for 16 h. The reaction mixture was poured onto saturated aqueous Na_2CO_3 and the crude product was extracted with CH_2Cl_2 (3 ×5.0 mL). The combined organic layers were dried with Na_2SO_4, filtered, concentrated, and purified by flash column chromatography to give the responding imines **(2a-2z')** in 65%-91% yields.

General procedure for the synthesis of *rac*-**1:** In a typical synthetic route, to a solution of the imine (1.0 mmol, 1.0 eq) in 10 mL of MeOH was added $NaBH_4$ (5.0 g, 5.0 eq) at 0 ℃. The resulting mixture was allowed to warm to 25 ℃ and stirred for a further 5 h. Upon completion, the solvent was removed by evaporation, and 5.0 mL of water was added. The mixture was extracted with CH_2Cl_2 (3 × 5.0 mL). The combined extracts were washed with the saturated NaCl solution and then dehydrated with Na_2SO_4.

After evaporation of the solvent, the resulting residue was purified by silica gel flash column chromatography to afford the racemic amine (*rac*-**1a**-*rac*-**1z')** in 48%-86% yields.

2.2 General procedure for the synthesis of racemic tetrahydroquinolines:

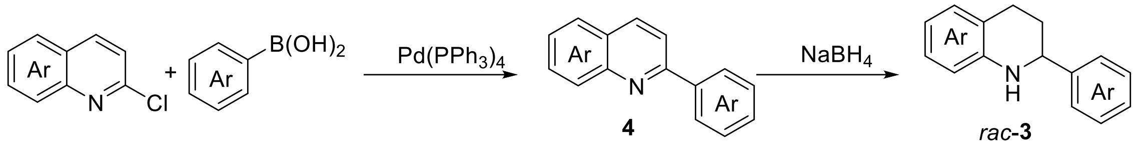

<u>General procedure for the synthesis of **4**</u>: In a typical synthetic route, to this suspension of tetrakis(triphenylphosphine)palladium (0.03 mmol, 1 mol%) was added 2-chloroquinoline (3.0 mmol, 1 equiv), arylboronic acid (3.9 mmol, 1.3 equiv) and anhydrous sodium carbonate (15 mmol, 5 equiv) in 30 mL of dioxane/water (v/v = 4/1). The resulting mixture was stirred at reflux for 12 h. The reaction mixture was then allowed to cool to room temperature and filtered through Celite. The filter cake was washed with ethyl acetate, and the organic layer of the filtrate was separated, washed with brine, dried ($NaSO_4$), and concentrated under reduced pressure. The resulting residue was purified by flash column chromatography to afford the responding imines **(4a-4r)** in 51%-87% yields.

<u>General procedure for the synthesis of *rac*-**3**</u>**:** In a typical synthetic route, to a solution of the imine (1.0 mmol, 1.0 eq) in 15 mL of glacial acetic acid was added sodium cyanoborohydride (5.0 eq) at 0 ℃. The reaction mixture was stirred overnight at room temperature, poured into saturated aqueous sodium carbonate, stirred for 15 min, and diluted with CH_2Cl_2. The organic layer was separated and the aqueous layer was extracted twice more with CH_2Cl_2 (3 × 5.0 mL). The combined organic extracts were dried ($NaSO_4$), concentrated under reduced pressure, and purified by column chromatography to afford the racemic amine (*rac*-**3a**-*rac*-**3r)** in 54%-89% yields.

2.3 General procedure for the synthesis of Hantzch esters (HTE):

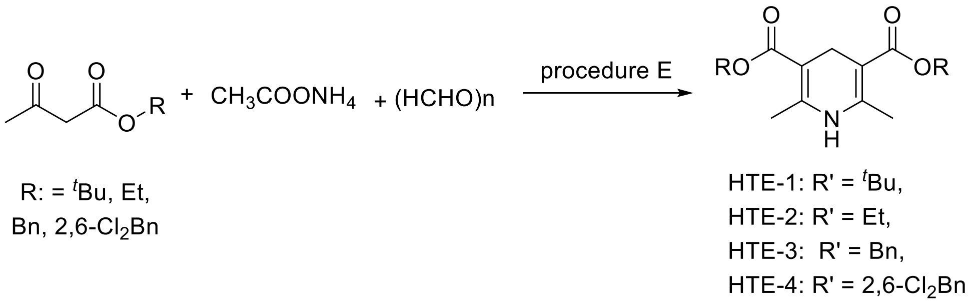

In a typical synthetic route, to a solution of ammonium acetate (10 mmol) and paraformaldehyde (10 mmol) was added ester (10 mmol) was added and the resulting mixture was stirred at 80 ℃ for 30 minutes under an argon atmosphere. A pale yellow precipitate formed through the course of the reaction, and the crude mixture was allowed to cool to room temperature and filtered through filter paper. The precipitate was washed with ethanol and diethyl ether and purified by column chromatography to afford the Hantzch esters (HTE-1—THE-4) in 58%-73% yields.

2.4. A schematic illustration of the reaction system of the light-driven redox deracemization of substrates (i) a saturated β-cyclodextrin emulsion, (ii-iv) a mixture of toluene and aqueous β-cyclodextrin emulsion containing HTE with and without the light irradiation, and (v-vi) the mixture before and after the reaction.

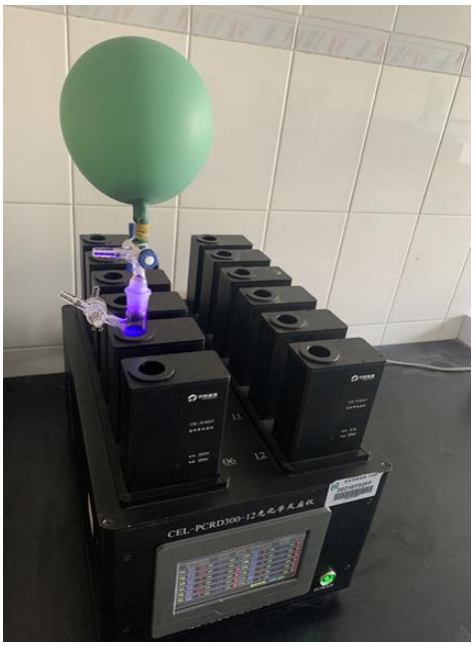

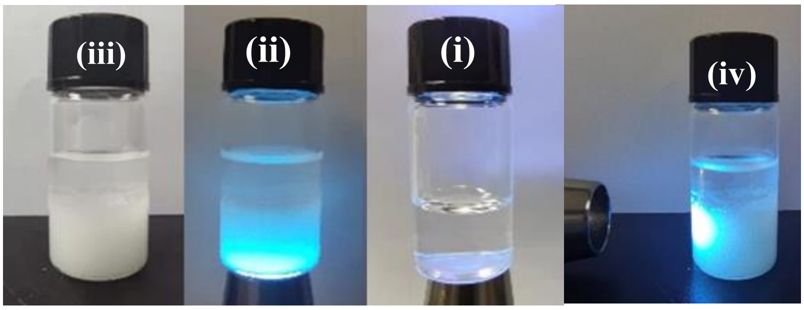

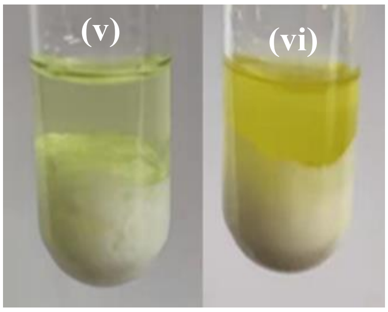

2.5. General procedure for the gram-scale preparation of (*R*)-**1i**.

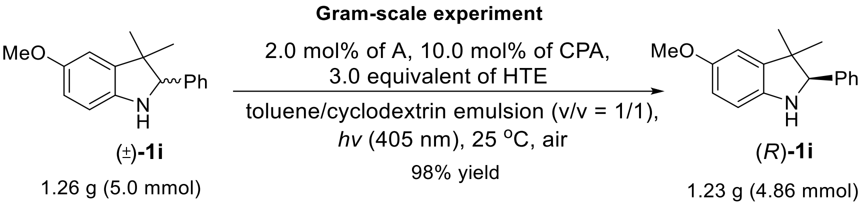

In an open-to-air test tube, to a suspension of di-*tert*-butyl 2,6-dimethyl-1,4-dihydropyridine-3,5-dicarboxylate (HTE-1) (3.0 equiv.) in 15.0 mL of the aqueous β-cyclodextrin emulsion was added slowly a solution of photocatalyst (2.0 mol%), chiral phosphoric acid (10.0 mol%) and racemic 5-methoxy-3,3-dimethyl-2-phenylindoline (1.26 g, 5.0 mmol) in 15.0 mL of toluene at room temperature. The resulting mixture under the light irradiations was then stirred at 25 ℃ for the first 2.5 h. Upon completion, the organic layers were collected and the aqueous solution was extracted with toluene (3 × 10.0 mL). After evaporation of the solvent, the resulting residue was purified by silica gel flash column chromatography (petroleum ether/ethyl acetate = 10:1) to afford (*R*)-**1i** (1.23 g, 98% yield) as a white solid.

3. Data of chiral products.

(*R*)-1a: (*R*)-3,3-dimethyl-2-phenylindoline. White solid, 99% yield, 98% *ee*. ^{1}H NMR (400 MHz, $CDCl_3$) δ 7.38 – 7.30 (m, 2H), 7.29 – 7.20 (m, 3H), 7.02 – 6.96 (m,2H), 6.73 – 6.68 (m,1H), 6.66 – 6.64 (m, 1H)4.52 (s, 1H), 1.35 (s, 3H), 0.66 (s, 3H). ^{13}C NMR (101 MHz, $CDCl_3$) δ 149.39, 140.01, 138.14, 128.18, 127.58, 122.57, 119.04, 109.26, 74.58, 45.42, 26.58, 24.61. HPLC (Chiralpak IC-H, elute: Hexanes/*i*-PrOH = 97/3, detector: 254 nm, flow rate: 0.5 mL/min, 25 ℃).

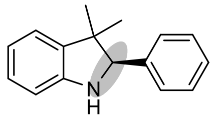

(*R*)-**1a**

(*R*)-1b: (*R*)-5-fluoro-3,3-dimethyl-2-phenylindoline. White solid, 99% yield, 97% *ee*.^{1}H NMR (400 MHz, $CDCl_3$) δ 7.37 – 7.34 (m, 2H), 7.29 – 7.21 (m, 3H), 6.71 – 6.66 (m, 2H), 6.55 – 6.52 (dd, J = 9.1, 4.3 Hz, 1H), 4.52 (s, 1H), 1.33 (s, 3H), 0.65 (s, 3H). ^{13}C NMR (101 MHz, $CDCl_3$) δ 158.62, 156.27, 144.85, 140.05, 139.98, 139.38, 128.17, 127.68, 127.45, 113.43, 113.20, 110.17, 109.93, 109.65, 109.58, 75.04, 45.71, 26.25, 24.24. ^{19}F NMR (376 MHz, $CDCl_3$) δ -125.62. HPLC (Chiralpak IC-H, elute: Hexanes/*i*-PrOH = 97/3, detector: 254 nm, flow rate: 0.5 mL/min, 25 ℃).

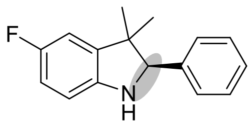

(*R*)-**1b**

(*R*)-1c: (*R*)-4-chloro-3,3-dimethyl-2-phenylindoline. Yellow solid, 95% yield, 90% *ee*. ^{1}H NMR (400 MHz, $CDCl_3$) δ 7.38 – 7.36 (m, 2H), 7.30 – 7.23 (m, 3H), 7.01 – 6.98 (dd, J = 8.0, 1.1 Hz, 1H), 6.86 – 6.84 (dd, J = 7.3, 1.1 Hz, 1H), 6.65 – 6.61 (dd, J = 8.0, 7.3 Hz, 1H), 4.56 (s, 1H), 1.35 (s, 3H), 0.66 (s, 3H). ^{13}C NMR (101 MHz, $CDCl_3$) δ 146.36, 139.59, 139.18, 128.21, 127.74, 127.40, 127.13, 120.70, 119.75, 114.62, 74.32, 46.58, 26.62, 24.51. HPLC(Chiracel OD-H, elute: Hexanes/i-PrOH = 97/3, detector: 254 nm, flow rate: 0.5 mL/min, 25 ℃).

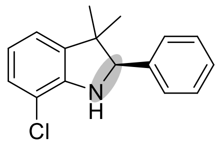

(*R*)-**1c**

(*R*)-1d: (*R*)-5-chloro-3,3-dimethyl-2-phenylindoline. White solid, 98% yield, 95% *ee*. ^{1}H NMR (400 MHz, $CDCl_3$) δ 7.36 – 7.33 (m, 2H), 7.30 – 7.23 (m, 3H), 6.96 – 6.91 (m, 2H), 6.58 – 6.56 (d, J = 8.2 Hz, 1H), 4.53 (s, 1H), 1.33 (s, 3H), 0.66 (s, 3H). ^{13}C NMR (101 MHz, $CDCl_3$) δ 147.81, 140.04, 139.36, 128.19, 127.71, 127.36, 127.12, 123.48, 122.91, 109.98, 74.78, 45.66, 26.46, 24.37. HPLC (Chiralpak IC-H, elute: Hexanes/*i*-PrOH = 97/3, detector: 254 nm, flow rate: 0.5 mL/min, 25 ℃).

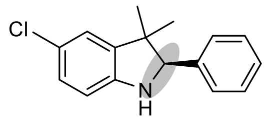

(*R*)-**1d**

(*R*)-1e: (*R*)-5-bromo-3,3-dimethyl-2-phenylindoline. White solid, 97% yield, 94% *ee*. ^{1}H NMR (400 MHz, $CDCl_3$) δ 7.35 – 7.23 (m, 5H), 7.10 – 7.04 (m, 2H), 6.52 – 6.50(d, J = 8.2 Hz, 1H), 4.52 (s, 1H), 1.33 (s, 3H), 0.65 (s, 3H). ^{13}C NMR (101 MHz, $CDCl_3$) δ 148.35, 140.48, 139.35, 130.01, 128.20, 127.72, 127.35, 125.73, 110.53, 74.71, 45.67, 26.52, 24.41. HPLC (Chiralpak IC-H, elute: Hexanes/*i*-PrOH = 97/3, detector: 254 nm, flow rate: 0.5 mL/min, 25 ℃).

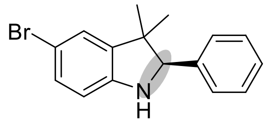

(*R*)-**1e**

(*R*)-1f: (*R*)-6-bromo-3,3-dimethyl-2-phenylindoline. White solid, 99% yield, 98% *ee*. ^{1}H NMR (400 MHz, $CDCl_3$) δ 7.34 – 7.23 (m, 5H), 6.81 (d, J = 1.0 Hz, 2H), 6.77 – 6.76(d, J = 1.1 Hz, 1H), 4.52 (s, 1H), 1.33 (s, 3H), 0.64 (s, 3H). ^{13}C NMR (101 MHz, $CDCl_3$) δ 150.69, 139.35, 137.19, 128.21, 127.72, 127.30, 123.77, 121.63, 120.80, 112.12, 74.60, 45.08, 26.61, 24.43. HPLC (Chiralpak IC-H, elute: Hexanes/*i*-PrOH = 97/3, detector: 254 nm, flow rate: 0.5 mL/min, 25 ℃).

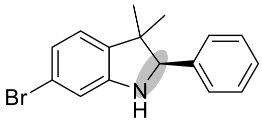

(*R*)-**1f**

(*R*)-1g: (*R*)-3,3-dimethyl-2-phenyl-5-(trifluoromethyl)indoline. White solid, 97% yield, 96% *ee*. ^{1}H NMR (400 MHz, $CDCl_3$) δ 7.32 – 7.22 (m, 6H), 7.15 – 7.14 (d, J = 2.1 Hz, 1H), 6.61 – 6.59 (d, J = 8.2 Hz, 1H), 4.56 (s, 1H), 1.35 (s, 3H), 0.65 (s, 3H). ^{13}C NMR (101 MHz, $CDCl_3$) δ 152.15, 139.14, 138.22, 128.27, 127.82, 127.23, 125.40, 119.69, 108.04, 74.59, 45.28, 26.76, 24.55. ^{19}F NMR (376 MHz, $CDCl_3$) δ -60.63. HPLC (Chiralpak IC-H, elute: Hexanes/*i*-PrOH = 97/3, detector: 254 nm, flow rate: 0.5 mL/min, 25 ℃).

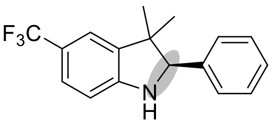

(*R*)-**1g**

(*R*)-1h: (*R*)-3,3,5-trimethyl-2-phenylindoline. White solid, 99% yield, 98% *ee*. ^{1}H NMR (400 MHz, $CDCl_3$) δ 7.42 – 7.39 (m, 2H), 7.32– 7.23 (m, 3H), 6.85 – 6.82(m, 2H), 6.60 – 6.58(d, J = 7.7 Hz, 1H), 4.52 (s, 1H), 2.25 (s, 3H), 1.37 (s, 3H), 0.68 (s, 3H). ^{13}C NMR (101 MHz, $CDCl_3$) δ 146.79, 139.97, 138.40, 128.08, 127.74, 127.51, 127.48, 123.28, 109.22, 74.78, 45.38, 26.44, 24.47, 21.01. HPLC (Chiralpak IC-H, elute: Hexanes/*i*-PrOH = 97/3, detector: 254 nm, flow rate: 0.5 mL/min, 25 ℃).

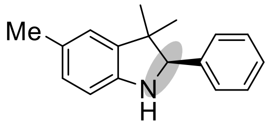

(*R*)-**1h**

(*R*)-1i: (*R*)-5-methoxy-3,3-dimethyl-2-phenylindoline. White solid, 99% yield, 98% *ee*. ^{1}H NMR (400 MHz, $CDCl_3$) δ 7.38 – 7.35 (m, 2H), 7.27 – 7.18 (m, 3H), 6.59– 6.55 (m, 3H), 4.47 (s, 1H), 3.68 (s, 3H), 1.32 (s, 3H), 0.64 (s, 3H). ^{13}C NMR (101 MHz, $CDCl_3$) δ 153.77, 142.96, 139.87, 128.09, 127.54, 111.92, 109.73, 75.02, 55.99, 45.71, 26.26, 24.28. HPLC (Chiralpak IC-H, elute: Hexanes/*i*-PrOH = 97/3, detector: 254 nm, flow rate: 0.5 mL/min, 25 ℃).

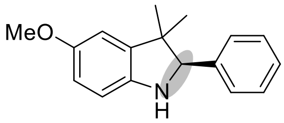

(*R*)-**1i**

(*R*)-1j: (*R*)-5-(benzyloxy)-3,3-dimethyl-2-phenylindoline. White solid, 99% yield, 97% *ee*. ^{1}H NMR (400 MHz, $CDCl_3$) δ 7.38 – 7.35 (m, 4H), 7.32 – 7.21 (m, 6H), 6.68 – 6.55 (m, 3H), 4.92 (s, 2H), 4.49 (s, 1H), 1.32 (s, 3H), 0.65 (s, 3H). ^{13}C NMR (101 MHz, $CDCl_3$) δ 152.97, 143.32, 139.89, 137.60, 128.66, 128.63, 128.54, 128.09, 127.85, 127.66, 127.53, 113.05, 110.97, 109.57, 75.00, 71.07, 45.71, 26.28, 24.31. HPLC (Chiralpak IC-H, elute: Hexanes/*i*-PrOH = 97/3, detector: 254 nm, flow rate: 0.5 mL/min, 25 ℃).

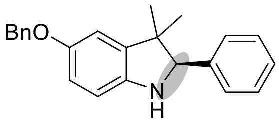

(*R*)-**1j**

(*R*)-1k: (*R*)-3,3-dimethyl-2-phenyl-5-(trifluoromethoxy)indoline. White solid, 98% yield, 96% *ee.* ^{1}H NMR (400 MHz, $CDCl_3$) δ 7.36 – 7.33 (m, 2H), 7.30 – 7.21 (m, 3H), 6.86 – 6.80 (m, 2H), 6.57 – 6.55 (d, J = 8.2 Hz, 1H), 4.58 (s, 1H), 1.36 (s, 3H), 0.67 (s, 3H). ^{13}C NMR (101 MHz, $CDCl_3$) δ 147.88, 142.05, 139.57, 139.25, 128.78, 128.43, 128.21, 127.74, 127.36, 120.46, 116.38, 109.01, 74.92, 45.59, 26.32, 24.68, 24.39. ^{19}F NMR (376 MHz, $CDCl_3$) δ -58.34. HPLC (Chiralpak IC-H, elute: Hexanes/*i*-PrOH = 97/3, detector: 254 nm, flow rate: 0.5 mL/min, 25 ℃).

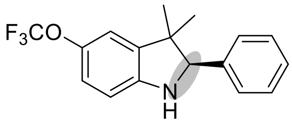

(*R*)-**1k**

(*R*)-1l: (*R*)-1,1-dimethyl-2-phenyl-2,3-dihydro-1H-benzo[e]indole. White solid, 97% yield, 94% *ee.* ^{1}H NMR (400 MHz, $CDCl_3$) δ 7.88 – 7.85 (dd, J = 8.7, 1.1 Hz, 1H), 7.70 – 7.68 (dd, J = 8.3, 1.4 Hz, 1H), 7.56 – 7.54 (d, J = 8.5 Hz, 1H), 7.46 – 7.44 (m, 2H), 7.32 – 7.23 (m, 4H), 7.15 – 7.11 (m, 1H), 6.97 – 6.95 (d, J = 8.5 Hz, 1H), 4.61 (s, 1H), 1.67 (s, 3H), 0.90 (s, 3H). ^{13}C NMR (101 MHz, $CDCl_3$) δ 146.93, 139.50, 131.06, 129.75, 129.52, 128.96, 128.18, 128.10, 127.72, 126.75, 126.20, 121.62, 112.94, 75.18, 47.19, 27.41, 23.05. HPLC (Chiracel OD-H, elute: Hexanes/*i*-PrOH = 90/10, detector: 254 nm, flow rate: 1.0 mL/min, 25 ℃)

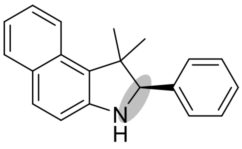

(*R*)-**1l**

(*R*)-1m：(*R*)-2-(4-chlorophenyl)-3,3-dimethylindoline. White solid, 99% yield, 98% *ee.* ^{1}H NMR (400 MHz, $CDCl_3$) δ 7.34 (d, J = 8.2 Hz, 2H), 7.29 – 7.22 (m, 2H), 7.06 – 6.96 (m, 2H), 6.78 (t, J = 7.4 Hz, 1H), 6.71 (d, J = 7.7 Hz, 1H), 4.52 (s, 1H), 1.34 (s, 3H), 0.67 (s, 3H). ^{13}C NMR (101 MHz, $CDCl_3$) δ 148.70, 138.22, 137.96, 133.19, 128.78, 128.29, 127.52, 122.54, 119.48, 109.58, 73.86, 45.40, 26.38, 24.54. HPLC (Chiralpak IC-H, elute: Hexanes/*i*-PrOH = 97/3, detector: 254 nm, flow rate: 0.5 mL/min, 25 ℃).

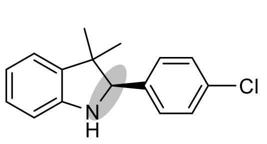

(*R*)-**1m**

(*R*)-1n: (*R*)-2-(4-ethylphenyl)-3,3-dimethylindoline. White solid, 99% yield, 99% *ee.* ^{1}H NMR (400 MHz, $CDCl_3$) δ 7.42 – 7.40 (m, 2H), 7.29 – 7.27 (m, 2H), 7.04 – 6.98 (m, 2H), 6.76 – 6.73 (t, J = 7.4 Hz, 1H), 6.68 – 6.66 (d, J = 7.7 Hz, 1H), 4.49 (s, 1H), 1.34 (s, 3H), 0.65 (s, 3H). ^{13}C NMR (101 MHz, $CDCl_3$) δ 148.89, 138.89, 137.86, 131.23, 129.13, 127.51, 122.53, 121.27, 119.32, 109.41, 73.90, 45.36, 26.39, 24.55. HPLC (Chiralpak IC-H, elute: Hexanes/*i*-PrOH = 97/3, detector: 254 nm, flow rate: 0.5 mL/min, 25 ℃).

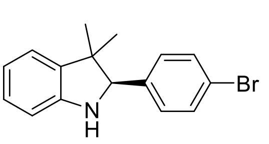

(*R*)-**1n**

(*R*)-1o: (*R*)-2-(4-bromophenyl)-3,3-dimethylindoline. White solid, 99% yield, 98% *ee*.^{1}H NMR (400 MHz, $CDCl_3$) δ 7.29 – 7.27 (d, J = 8.1 Hz, 2H), 7.12 – 7.09 (m, 2H), 7.02 – 6.96 (m, 2H), 6.72 – 6.68 (m, 1H), 6.65 – 6.62 (m, 1H), 4.49 (s, 1H), 2.61 – 2.56 (q, J = 7.6 Hz, 2H), 1.34 (s, 3H), 1.19 – 1.15 (t, J = 7.6 Hz, 3H), 0.66 (s, 3H). ^{13}C NMR (101 MHz, $CDCl_3$) δ 149.40, 143.53, 138.19, 137.10, 127.58, 127.44, 127.35, 122.50, 118.89, 109.11, 74.37, 45.28, 28.57, 26.48, 24.51, 15.67. HPLC (Chiralpak OD-H, elute: Hexanes/*i*-PrOH = 90/10, detector: 254 nm, flow rate: 1.0 mL/min, 25 ℃).

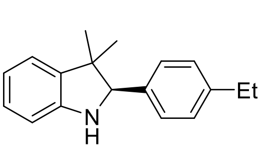

(*R*)-**1o**

(*R*)-1p: (*R*)-3,3-dimethyl-2-(4-propoxyphenyl)indoline. White solid, 98% yield, 98% *ee*.^{1}H NMR (400 MHz, $CDCl_3$) δ 7.39 – 7.36 (d, J = 8.7 Hz, 2H), 7.12 – 7.06 (m, 2H), 6.91 – 6.89 (m, 2H), 6.82 – 6.79 (t, J = 7.4 Hz, 1H), 6.75 – 6.73 (d, J = 7.7 Hz, 1H), 4.57 (s, 1H), 4.10 – 4.04 (q, J = 7.0 Hz, 2H), 1.47 – 1.43 (t, J = 7.1 Hz, 3H), 1.42 (s, 3H), 0.76 (s, 3H). ^{13}C NMR (101 MHz, $CDCl_3$) δ 158.43, 149.26, 138.23, 131.68, 128.48, 127.33, 122.53, 118.97, 113.99, 109.18, 74.07, 63.42, 45.24, 26.43, 24.44, 14.92. HPLC (Chiralpak OD-H, elute: Hexanes/*i*-PrOH = 90/10, detector: 254 nm, flow rate: 1.0 mL/min, 25 ℃).

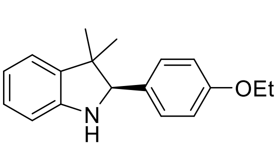

(*R*)-**1p**

(*R*)-1q: (*R*)-2'-phenylspiro[cyclopentane-1,3'-indoline. White solid, 97% yield, 93% *ee*.[1] H NMR (400 MHz, $CDCl_3$) δ 7.31 – 7.20 (m, 5H), 7.02 – 7.68 (t, J = 7.5 Hz, 2H), 6.73 – 6.63 (m, 2H), 4.58 (s, 1H), 1.96 – 1.92 (m, 2H), 1.77 – 1.50 (m, 4H), 1.39 – 1.35 (t, J = 7.1 Hz, 2H). ^{13}C NMR (101 MHz, $CDCl_3$) δ 149.74, 141.10, 138.06, 128.21, 127.64, 127.31, 122.82, 119.08, 108.80, 73.70, 57.24, 39.60, 34.91, 24.57. HPLC (Chiralpak IC-H, elute: Hexanes/*i*-PrOH = 97/3, detector: 254 nm, flow rate: 0.5 mL/min, 25 ℃).

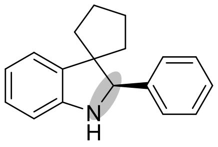

(*R*)-**1q**

(*R*)-1r: (R)-5'-fluoro-2'-phenylspiro[cyclopentane-1,3'-indoline. White solid, 98% yield, 96% *ee*.[1] H NMR (400 MHz, $CDCl_3$) δ 7.45 – 7.30 (m, 5H), 6.85 – 6.74 (m, 2H), 6.62 (dd, J = 8.2, 4.4 Hz, 1H), 4.68 (s, 1H), 2.03 (t, J = 7.4 Hz, 2H), 1.88 – 1.58 (m, 3H), 1.54 – 1.40 (m, 2H), 1.28 – 1.22 (m, 1H). ^{13}C NMR (101 MHz, $CDCl_3$) δ 158.58, 156.24, 145.61, 140.76, 128.25, 127.76, 127.59, 113.23, 113.00, 110.40, 110.17, 74.27, 39.45, 34.78, 24.61, 24.53. ^{19}F NMR (376 MHz, $CDCl_3$) δ -125.62. HPLC (Chiralpak IC-H, elute: Hexanes/*i*-PrOH = 97/3, detector: 254 nm, flow rate: 0.5 mL/min, 25 ℃).

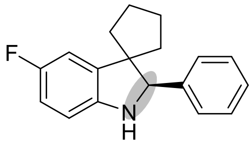

(*R*)-**1r**

(*R*)-1s: 5'-bromo-2'-phenylspiro[cyclopentane-1,3'-indoline. White solid, 95% yield, 93% *ee*. ^{1}H NMR (400 MHz, $CDCl_3$) δ 7.28 – 7.19 (m, 5H), 7.10 – 7.05 (m, 2H), 6.50 – 6.48 (d, J = 8.1 Hz, 1H), 4.56 (s, 1H), 1.91 – 1.87 (m, 2H), 1.88 – 1.58 (m, 1H), 1.53 – 1.50 (m, 3H), 1.38 – 1.34 (m, 2H), 1.22 – 1.12 (m, 1H). ^{13}C NMR (101 MHz, $CDCl_3$) δ 148.73, 140.52, 129.90, 128.30, 127.85, 127.51, 125.88, 110.58,

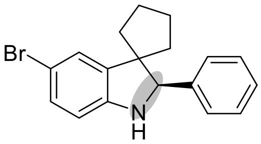

(*R*)-**1s**

110.13, 73.96, 57.45, 39.77, 34.80, 24.57, 24.50. HPLC (Chiralpak IC-H, elute: Hexanes/*i*-PrOH = 97/3, detector: 254 nm, flow rate: 0.5 mL/min, 25 ℃).

(*R*)-1t: (*R*)-2'-phenylspiro[cyclohexane-1,3'-indoline. White solid, 95% yield, 95% *ee*. ^{1}H NMR (400 MHz, $CDCl_3$) δ 7.24 – 7.15 (m, 6H), 7.02 – 6.98 (m, 1H), 6.71 – 6.67 (m, 1H), 6.60 – 6.58 (m, 1H), 4.48(s, 1H), 1.78 –1.60(m,4H), 1.51 – 1.45 (m, 1H), 1.43 – 1.30 (m, 3H),1.16– 1.04(m, 2H). ^{13}C NMR (101 MHz, $CDCl_3$) δ 149.93, 141.14, 137.26, 128.12, 127.95, 127.64, 127.49, 124.32, 118.61, 108.91, 72.98, 49.20, 37.32, 31.81, 25.76, 23.01, 22.18. HPLC (Chiralpak IC-H, elute: Hexanes/*i*-PrOH = 97/3, detector: 254 nm, flow rate: 0.5 mL/min, 25 ℃).

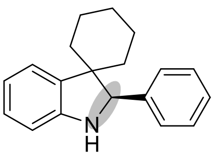

(*R*)-**1t**

(*R*)-1u: (*R*)-5'-fluoro-2'-phenylspiro[cyclohexane-1,3'-indoline. White solid, 93% yield, 94% *ee*.^{1}H NMR (400 MHz, $CDCl_3$) δ 7.23 – 7.19 (q, J = 2.3, 1.8 Hz, 5H), 6.93 – 6.90 (dd, J = 9.0, 2.6 Hz, 1H), 6.73 – 6.68 (m, 1H), 6.52 – 6.49 (dd, J = 8.4, 4.4 Hz, 1H),δ 4.50 (s, 1H), 1.79 – 1.57 (m, 4H), 1.48 – 1.29 (m, 4H), 1.18 – 1.03 (m, 2H). ^{13}C NMR (101 MHz, $CDCl_3$) δ 158.13, 155.79, 145.93, 140.86, 138.99, 138.92, 128.18, 127.89, 127.77, 113.38, 113.15, 112.05, 111.81, 108.87, 108.79, 73.58, 49.45, 36.97, 31.62, 25.62, 22.90, 22.04. ^{19}F NMR (376 MHz, $CDCl_3$) δ -126.06. HPLC (Chiracel OD-H, n-hexane/2-propanol = 90/10, detector: 254 nm, flow rate = 1.0 mL/min, 25 ℃).

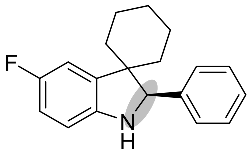

(*R*)-**1u**

(*R*)-1v: (*R*)-5'-chloro-2'-phenylspiro[cyclohexane-1,3'-indoline. White solid, 97% yield, 96% *ee*. ^{1}H NMR (400 MHz, $CDCl_3$) δ 7.21 – 7.18 (dd, J = 8.9, 0.8 Hz, 5H), 7.11 (d, J = 2.1 Hz, 1H),6.98 – 6,94 (d, J = 2.1 Hz, 1H), 6.53 – 6.51(m, 1H), 4.51 (s, 1H), 1.80 – 1.55 (m, 4H), 1.48 – 1.31 (m, 4H), 1.15 – 1.07 (m, 2H). ^{13}C NMR (101 MHz, $CDCl_3$) δ 148.61, 140.71, 139.16, 128.21, 127.83, 127.18, 124.52, 122.99, 109.42, 73.17, 49.49, 37.22, 31.59, 25.60, 22.85, 22.09. HPLC (Chiralpak IC-H, elute: Hexanes/*i*-PrOH = 97/3, detector: 254 nm, flow rate: 0.5 mL/min, 25 ℃).

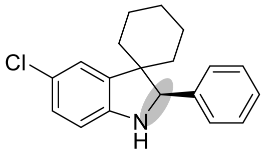

(*R*)-**1v**

(*R*)-1w: (*R*)-5'-bromo-2'-phenylspiro[cyclohexane-1,3'-indoline. White solid, 96% yield, 95% *ee*.^{1}H NMR (400 MHz, $CDCl_3$) δ 7.24 – 7.19(m, 6H), 7.11 – 7.09 (dd, J = 8.2, 2.0 Hz, 1H), 6.49 – 6.47(d, J = 8.1 Hz, 1H), 1.81 – 1.55(m, 4H), 1.48– 1.32 (m, 4H), 1.22 – 1.06 (m, 2H). ^{13}C NMR (101 MHz, $CDCl_3$) δ 149.15, 140.73, 139.61, 130.08, 128.21, 127.83, 127.24, 110.00, 109.95, 73.06, 49.52, 37.29, 31.60, 25.60, 22.85, 22.12. HPLC (Chiralpak IC-H, elute: Hexanes/*i*-PrOH = 97/3, detector: 254 nm, flow rate: 0.5 mL/min, 25 ℃).

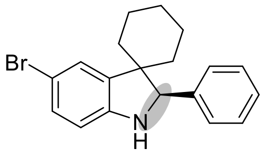

(*R*)-**1w**

(*R*)-1x: (*R*)-6'-bromo-2'-phenylspiro[cyclohexane-1,3'-indoline. White solid, 97% yield, 96% *ee.*^{1}H NMR (400 MHz, $CDCl_3$) δ 7.21 – 7.18 (d, J = 8.5 Hz, 5H), 7.01 – 6.99 (d, J = 7.9 Hz, 1H), 6.80 – 6.78 (dd, J = 7.9, 1.8 Hz, 1H), 6.72 (d, J = 1.8 Hz, 1H), 4.50 (s, 1H), 1.78 – 1.53 (m, 4H), 1.46 – 1.30 (m, 4H), 1.12 – 1.03 (m, 2H). ^{13}C NMR (101 MHz, $CDCl_3$) δ 151.41, 140.59, 136.34, 128.22, 127.83, 125.39, 121.15, 120.99, 111.63, 73.00, 48.90, 37.37, 31.66, 25.62, 22.86, 22.13. HPLC (Chiralpak IC-H, elute: Hexanes/*i*-PrOH = 97/3, detector: 254 nm, flow rate: 0.5 mL/min, 25 ℃).

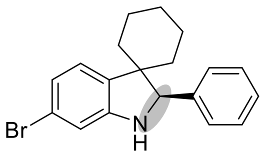

(*R*)-**1x**

(*R*)-1y: (*R*)-2'-phenyl-5'-(trifluoromethyl)spiro[cyclohexane-1,3'-indoline. White solid, 98% yield, 97% *ee.*^{1}H NMR (400 MHz, $CDCl_3$) δ 7.32 (d, J = 1.8 Hz, 1H), 7.27 – 7.16 (m, 6H), 6.56 – 6.54 (d, J = 8.1 Hz, 1H), 4.56 (s, 1H), 1.81 – 1.45 (m, 5H), 1.40 – 1.30 (m, 3H), 1.16 – 1.06 (m, 2H). ^{13}C NMR (101 MHz, $CDCl_3$) δ 152.70, 140.41, 137.39, 128.28, 127.96, 127.78, 125.56, 121.11, 107.51, 72.78, 49.13, 37.63, 31.62, 25.55, 22.77, 22.17. ^{19}F NMR (376 MHz, $CDCl_3$) δ -60.45. HPLC (Chiralpak OD-H, elute: Hexanes/*i*-PrOH = 90/10, detector: 254 nm, flow rate: 1.0 mL/min, 25 ℃).

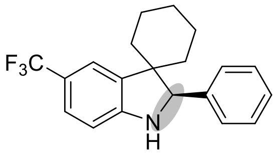

(*R*)-**1y**

(*R*)-1z: (*R*)-2'-phenyl-5'-(trifluoromethoxy)spiro[cyclohexane-1,3'-indoline. White solid, 98% yield, 96% *ee.*^{1}H NMR (400 MHz, $CDCl_3$) δ 7.22 (s, 5H), 7.02 (s, 1H), 6.87 – 6.84 (m, 1H), 6.53 – 6.51 (d, J = 8.4 Hz, 1H), 4.53 (s, 1H), 1.79 – 1.58 (m, 4H), 1.48 – 1.26 (m, 4H), 1.13 – 1.05 (m, 2H). ^{13}C NMR (101 MHz, $CDCl_3$) δ 148.66, 141.57, 140.55, 138.55, 128.21, 127.86, 120.47, 118.24, 108.32, 73.50, 49.31, 37.07, 31.65, 25.56, 22.82, 21.97. ^{19}F NMR (376 MHz, $CDCl_3$) δ -58.31. HPLC (Chiralpak OD-H, elute: Hexanes/*i*-PrOH = 90/10, detector: 254 nm, flow rate: 1.0 mL/min, 25 ℃).

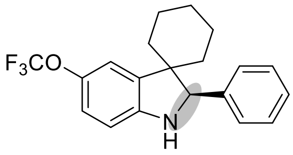

(*R*)-**1z**

(*R*)-1z': (*R*)-5'-methoxy-2'-phenylspiro[cyclohexane-1,3'-indoline. White solid, 97% yield, 96% *ee.*^{1}H NMR (400 MHz, $CDCl_3$) δ 7.24 – 7.18 (m, 5H), 6.82 (d, J = 2.5 Hz, 1H), 6.59 – 6.52 (m, 2H), 4.48 (s, 1H), 3.71 (s, 3H), 1.78 – 1.59 (m, 4H), 1.50 – 1.26 (m, 4H), 1.26 – 1.04 (m, 2H). ^{13}C NMR (101 MHz, $CDCl_3$) δ 153.36, 143.87, 141.32, 139.10, 128.10, 127.94, 127.60, 111.98, 111.60, 109.10, 73.41, 56.02, 49.51, 37.10, 31.66, 25.71, 23.03, 22.16. HPLC (Chiralpak OD-H, elute: Hexanes/*i*-PrOH = 90/10, detector: 254 nm, flow rate: 1.0 mL/min, 25 ℃).

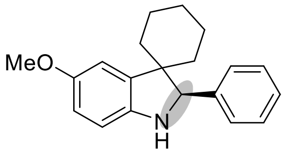

(*R*)-**1z'**

(*S*)-3a: (*S*)-2-phenyl-1,2,3,4-tetrahydroquinoline. White solid, 96% yield, 90% *ee*. ^{1}H NMR (400 MHz, CDCl3) δ 7.34 – 7.15 (m, 5H), 6.95 – 6.91 (m, 2H), 6.64 – 6.60 (m, 1H), 6.51 – 6.48 (dd, J = 8.3, 1.2 Hz, 1H), 4.37 – 4.33 (dd, J = 9.4, 3.3 Hz, 1H), 2.84 – 2.80 (m, 1H), 2.70 – 2.65 (m, 1H), 2.06 – 1.97 (m, 2H). ^{13}C NMR (101 MHz, CDCl3) δ 144.86, 144.77, 129.39, 128.65, 127.52, 126.98, 126.64, 120.95, 117.25, 114.08, 56.31, 31.05, 26.45.HPLC (Chiralpak OD-H, elute: Hexanes/*i*-PrOH = 90/10, detector: 254 nm, flow rate: 1.0 mL/min, 25 ℃).

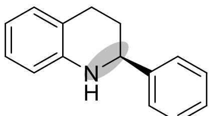

(*S*)-**3a**

(*S*)-3b: (*S*)-2-(4-fluorophenyl)-1,2,3,4-tetrahydroquinoline. White solid, 96% yield, 94% *ee*. ^{1}H NMR (400 MHz, CDCl3) δ 7.31 – 7.28 (m, 2H), 6.99 – 6.93 (m, 4H), 6.63 – 6.59 (m, 1H), 6.50 – 6.48 (d, J = 7.8 Hz, 1H), 4.38 – 4.35f (dd, J = 9.4, 3.2 Hz, 1H), 2.90 – 2.82 (m, 1H), 2.70 – 2.63 (m, 1H), 2.07 – 1.86 (m, 2H). ^{13}C NMR (101 MHz, CDCl3) δ 163.35, 160.91, 144.51, 140.48, 129.36, 128.16, 128.08, 126.97, 120.89, 117.43, 115.48, 115.26, 114.12, 55.62, 31.13, 26.32. ^{19}F NMR (376 MHz, CDCl3) δ -115.26. HPLC (Chiralpak OD-H, elute: Hexanes/*i*-PrOH = 90/10, detector: 254 nm, flow rate: 1.0 mL/min, 25 ℃).

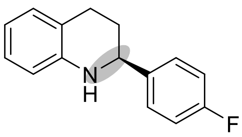

(*S*)-**3b**

(*S*)-3c: (S)-2-(4-(trifluoromethyl)phenyl)-1,2,3,4-tetrahydroquinoline. White solid, 94% yield, 95% *ee*. ^{1}H NMR (400 MHz, CDCl3) δ 7.54 – 7.52 (d, J = 8.1 Hz, 2H), 7.44 – 7.42 (d, J = 8.0 Hz, 2H), 6.98 – 6.93 (m, 2H), 6.63 – 6.60 (m, 1H), 6.52 – 6.50 (d, J = 8.1 Hz, 1H), 4.47 – 4.44 (dd, J = 8.9, 3.4 Hz, 1H), 2.88 – 2.80 (m, 1H), 2.67 – 2.60(m, 1H), 2.10 – 1.87 (m, 2H). ^{13}C NMR (101 MHz, CDCl3) δ 148.91, 144.20, 129.40, 127.08, 126.92, 125.58, 125.54, 122.86, 120.85, 117.65, 114.21, 55.80, 30.86, 25.96. ^{19}F NMR (376 MHz, CDCl3) δ -62.36. HPLC (Chiralpak OD-H, elute: Hexanes/*i*-PrOH = 90/10, detector: 254 nm, flow rate: 1.0 mL/min, 25 ℃).

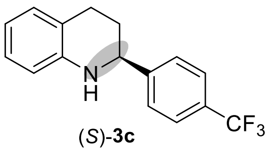

(*S*)-**3c**

(*S*)-3d: (*S*)-4-(1,2,3,4-tetrahydroquinolin-2-yl)benzonitrile. White solid, 96% yield, 97% *ee*. ^{1}H NMR (400 MHz, CDCl3)δ 7.55 – 7.53 (m, 2H), 7.42 – 7.40 (m, 2H), 6.97 – 6.91 (m, 2H), 6.63 – 6.59 (m, 1H), 6.52 – 6.49 (dd, J = 8.0, 1.2 Hz, 1H), 4.46 – 4.43 (dd, J = 8.6, 3.5 Hz, 1H), 2.85 – 2.77 (m, 1H), 2.63 – 2.56 (m, 1H), 2.08 – 2.02 (m, 1H), 1.93 – 1.83 (m, 1H). ^{13}C NMR (101 MHz, CDCl3) δ 150.39, 143.90, 132.47, 129.40, 127.34, 127.15, 120.76, 118.91, 117.81, 114.28, 111.16, 55.75, 30.67, 25.69. HPLC (Chiralpak OD-H, elute: Hexanes/*i*-PrOH = 80/20, detector: 254 nm, flow rate: 1.0 mL/min, 25 ℃).

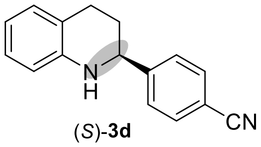

(*S*)-**3d**

(*S*)-3e: (*S*)-2-(3-nitrophenyl)-1,2,3,4-tetrahydroquinoline. White solid, 96% yield, 90% *ee*. ^{1}H NMR (400 MHz, CDCl3) δ 8.19 – 8.18 (t, J = 2.0 Hz, 1H), 8.07 – 8.04 (m, 1H), 7.67 – 7.65 (m, 1H), 7.46 – 7.42 (t, J = 7.9 Hz, 1H), 6.98 – 6.92 (M, 2H), 6.62 (td, J = 7.4, 1.2 Hz, 1H), 6.64 – 6.51 (dd, J = 8.0, 1.2 Hz, 1H), 4.51 – 4.48 (dd, J = 9.0, 3.4 Hz, 1H), 2.89 – 2.81 (m, 1H), 2.67 – 2.60 (m, 1H), 2.11 – 1.88 (m, 2H). ^{13}C NMR (101 MHz, CDCl3) δ 148.51, 147.11, 143.94, 132.84, 129.59, 129.39, 127.15, 122.51, 121.63,

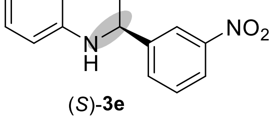

(*S*)-**3e**

120.79, 117.92, 114.43, 55.57, 30.99, 25.94. HPLC (Chiralpak OD-H, elute: Hexanes/*i*-PrOH = 90/10, detector: 254 nm, flow rate: 1.0 mL/min, 25 ℃).

(*S*)-3f: methyl (*S*)-4-(1,2,3,4-tetrahydroquinolin-2-yl)benzoate. White solid, 99% yield, 98% *ee*. ^{1}H NMR (400 MHz, $CDCl_3$) δ 7.94 – 7.92 (m, 2H), 7.39 – 7.37 (d, J = 8.3 Hz, 2H), 6.97 – 6.91 (m, 2H), 6.61 – 6.57 (m, 1H), 6.51 – 6.48 (dd, J = 8.0, 1.2 Hz, 1H), 4.45 – 4.41 (dd, J = 8.9, 3.4 Hz, 1H), 3.83 (s, 3H), 2.87 – 2.79 (m, 1H), 2.66 – 2.59 (m, 1H), 2.08 – 2.02 (m, 1H), 1.95 – 1.85 (m, 1H). ^{13}C NMR (101 MHz, $CDCl_3$) δ 166.97, 150.10, 144.25, 129.95, 129.36, 129.29, 127.04, 126.55, 120.86, 117.52, 114.17, 55.94, 52.15, 30.81, 26.01. HPLC (Chiralpak OD-H, elute: Hexanes/*i*-PrOH = 90/10, detector: 254 nm, flow rate: 1.0 mL/min, 25 ℃).

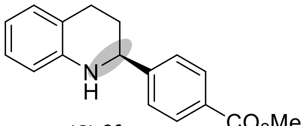

(*S*)-**3f**

(*S*)-3g: (*S*)-2-(p-tolyl)-1,2,3,4-tetrahydroquinoline. White solid, 96% yield, 91% *ee*. ^{1}H NMR (400 MHz, $CDCl_3$) δ 7.28 – 7.26 (d, J = 7.9 Hz, 2H), 7.19 – 7.14 (t, J = 9.4 Hz, 2H), 7.01 – 6.98 (t, J = 7.3 Hz, 2H), 6.66 – 6.62 (m, 1H), 6.52 – 6.50 (dd, J = 8.2, 1.4 Hz, 1H), 4.40 – 4.36 (dd, J = 9.4, 3.3 Hz, 1H), 2.95 – 2.87 (m, 1H), 2.75 – 2.69 (m, 1H), 2.34 (s, 3H), 2.12 – 2.05 (m, 1H), 2.01 – 1.91 (m, 1H). ^{13}C NMR (101 MHz, $CDCl_3$) δ 144.83, 141.86, 137.15, 129.35, 129.30, 126.93, 126.53, 120.94, 117.16, 114.03, 56.06, 31.08, 26.55, 21.18. HPLC (Chiralpak OD-H, elute: Hexanes/*i*-PrOH = 90/10, detector: 254 nm, flow rate: 1.0 mL/min, 25 ℃).

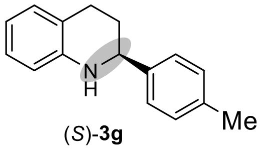

(*S*)-**3g**

(*S*)-3h: (*S*)-2-(m-tolyl)-1,2,3,4-tetrahydroquinoline. White solid, 93% yield, 90% *ee*. ^{1}H NMR (400 MHz, $CDCl_3$) δ 7.13 (m, 3H), 7.02 (d, J = 7.4 Hz, 1H), 6.92 (d, J = 7.4 Hz, 2H), 6.61 – 6.53 (m, 1H), 6.49 – 6.42 (m, 1H), 4.31 (dd, J = 9.6, 3.3 Hz, 1H), 2.84 (m, 1H), 2.66 (m, 1H), 2.28 (s, 3H), 2.02 (m, 1H), 1.90 (m, 1H). ^{13}C NMR (101 MHz, $CDCl_3$) δ 144.72, 138.29, 129.34, 128.53, 128.26, 127.31, 126.94, 123.70, 121.02, 117.25, 114.10, 56.36, 31.06, 26.59, 21.54. HPLC (Chiralpak OD-H, elute: Hexanes/*i*-PrOH = 90/10, detector: 254 nm, flow rate: 1.0 mL/min, 25 ℃).

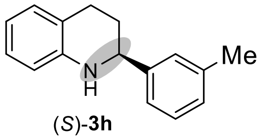

(*S*)-**3h**

(*S*)-3i: (*S*)-2-(4-methoxyphenyl)-1,2,3,4-tetrahydroquinoline. White solid, 95% yield, 92% *ee*. ^{1}H NMR (400 MHz, $CDCl_3$) δ 7.26 – 7.24 (d, J = 8.5 Hz, 2H), 6.95 – 6.91 (p, J = 4.1, 3.3 Hz, 2H), 6.83 – 6.80 (m, 2H), 6.61 – 6.57 (t, J = 7.4 Hz, 1H), 6.47 – 6.45 (m, 1H), 4.32 – 4.29 (dd, J = 9.6, 3.2 Hz, 1H), 3.74 (s, 3H), 2.90 – 2.81 (m, 1H), 2.71 – 2.64 (m, 1H), 2.05 – 1.87 (m, 2H). ^{13}C NMR (101 MHz, $CDCl_3$) δ 158.97, 144.72, 136.81, 129.33, 127.69, 126.89, 120.98, 117.23, 114.08, 113.92, 55.76, 55.35, 31.08, 26.58. HPLC (Chiralpak OD-H, elute: Hexanes/*i*-PrOH = 90/10, detector: 254 nm, flow rate: 1.0 mL/min, 25 ℃).

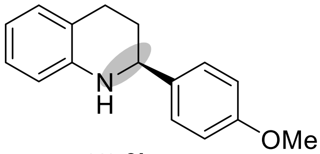

(*S*)-**3i**

(*S*)-3j: (*S*)-2-(4-(methylthio)phenyl)-1,2,3,4-tetrahydroquinoline. White solid, 96% yield, 93% *ee*.

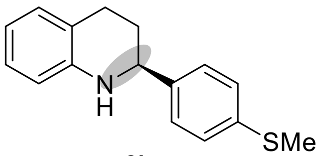

(*S*)-**3j**

^{1}H NMR (400 MHz, $CDCl_3$) δ 7.23 – 7.21 (m, 2H), 7.17 – 7.14 (m, 1H), 6.95 – 6.90 (t, J = 7.6 Hz, 2H), 6.59 – 6.55 (m, 1H), 6.46 – 6.44 (m, 1H), 4.32 – 4.29 (dd, J = 9.3, 3.3 Hz, 1H), 2.87 – 2.78 (m, 1H), 2.67 – 2.61 (m, 1H), 2.40 (s, 3H), 2.04 – 1.97 (m, 1H), 1.92 – 1.83 (m, 1H). ^{13}C NMR (101 MHz, $CDCl_3$) δ 144.60, 141.77, 137.38, 129.35, 127.14, 126.96, 126.89, 120.92, 117.32, 114.09, 55.82, 30.96, 26.36, 16.05. HPLC (Chiralpak OD-H, elute: Hexanes/*i*-PrOH = 90/10, detector: 254 nm, flow rate: 1.0 mL/min, 25 ℃).

(*S*)-3k: (*S*)-2-(4-(trimethylsilyl)phenyl)-1,2,3,4-tetrahydroquinoline. White solid, 95% yield, 91% *ee*.

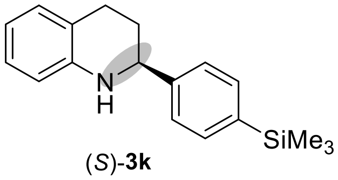

(*S*)-**3k**

^{1}H NMR (400 MHz, $CDCl_3$) δ 7.47 – 7.40 (m, 2H), 7.35 – 7.27 (m, 2H), 6.93 (t, J = 7.1 Hz, 2H), 6.57 (td, J = 7.4, 1.2 Hz, 1H), 6.48 – 6.42 (m, 1H), 4.35 (dd, J = 9.4, 3.3 Hz, 1H), 2.84 (ddd, J = 16.3, 10.7, 5.5 Hz, 1H), 2.66 (dt, J = 16.4, 4.8 Hz, 1H), 2.04 (dddd, J = 13.1, 5.4, 4.4, 3.3 Hz, 1H), 1.92 (dddd, J = 13.0, 10.7, 9.4, 5.1 Hz, 1H), 0.19 (s, 9H). ^{13}C NMR (101 MHz, $CDCl_3$) δ 146.39, 145.72, 140.68, 134.72, 130.38, 127.98, 127.08, 121.98, 118.28, 115.09, 57.33, 31.93, 27.50, -0.00. HPLC (Chiralpak OD-H, elute: Hexanes/*i*-PrOH = 90/10, detector: 254 nm, flow rate: 1.0 mL/min, 25 ℃).

(*S*)-3l: (*S*)-2-([1,1'-biphenyl]-4-yl)-1,2,3,4-tetrahydroquinoline. White solid, 92% yield, 92% *ee*.

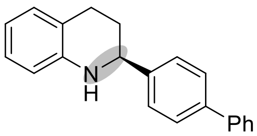

(*S*)-**3l**

^{1}H NMR (400 MHz, $CDCl_3$) δ 7.53 – 7.50 (dd, J = 8.1, 4.3 Hz, 4H), 7.40 – 7.34 (dd, J = 15.3, 7.8 Hz, 4H), 7.29 – 7.25 (t, J = 7.4 Hz, 1H), 6.96 – 6.92 (m, 2H), 6.63 – 6.51 (m, 2H), 4.43 – 4.39 (dd, J = 9.2, 3.3 Hz, 1H), 2.91–2.83 (m, 1H), 2.73–2.64 (m, 1H), 2.13 – 1.94 (m, 2H). ^{13}C NMR (101 MHz, $CDCl_3$) δ 144.58, 143.80, 140.86, 140.45, 129.36, 128.82, 127.34, 127.31, 127.11, 127.04, 126.96, 120.99, 117.34, 114.13, 56.00, 30.94, 26.39.HPLC (Chiralpak OD-H, elute: Hexanes/*i*-PrOH = 90/10, detector: 254 nm, flow rate: 1.0 mL/min, 25 ℃).

(*S*)-3m: (*S*)-2-(thiophen-2-yl)-1,2,3,4-tetrahydroquinoline. White solid, 93% yield, 90% *ee*.

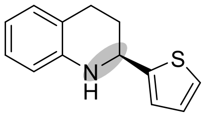

(*S*)-**3m**

^{1}H NMR (400 MHz, $CDCl_3$) δ 7.22 – 7.20 (dd, J = 5.0, 3.0 Hz, 1H), 7.13 – 7.09 (m, 1H), 7.01 – 6.99 (dd, J = 5.0, 1.4 Hz, 1H), 6.94 – 6.89 (t, J = 7.7 Hz, 2H), 6.59 – 6.55(m, 1H), 6.45 – 6.43 (d, J = 7.9 Hz, 1H), 4.48 – 4.45 (dd, J = 9.1, 3.2 Hz, 1H), 2.85 – 2.77 (m, 1H), 2.68 – 2.61 (m, 1H), 2.08 – 1.87 (m, 2H). ^{13}C NMR (101 MHz, $CDCl_3$) δ 146.01, 144.29, 129.36, 126.92, 126.14, 121.03, 120.83, 117.44, 114.20, 52.12, 30.22, 26.21. HPLC (Chiralpak OD-H, elute: Hexanes/*i*-PrOH = 90/10, detector: 254 nm, flow rate: 1.0 mL/min, 25 ℃).

(*S*)-3n: (*S*)-6-fluoro-2-phenyl-1,2,3,4-tetrahydroquinoline. White solid, 91% yield, 87% *ee*.

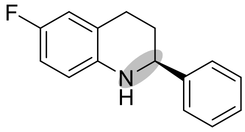

(*S*)-**3n**

^{1}H NMR (400 MHz, $CDCl_3$) δ 7.31 – 7.19 (m, 5H), 6.66 – 6.62 (m, 2H), 6.39 – 6.36 (dd, J = 9.5, 4.8 Hz, 1H), 4.31 – 4.28 (dd, J = 9.5, 3.2 Hz, 1H), 2.87 – 2.79 (m, 1H), 2.66 – 2.60 (m, 1H), 2.05 – 1.99 (m, 1H), 1.94 – 1.84 (m, 1H). ^{13}C NMR

(101 MHz, $CDCl_3$) δ 156.75, 154.41, 144.52, 140.93, 128.64, 127.57, 126.57, 122.27, 122.21, 115.58, 115.37, 114.75, 114.68, 113.54, 113.32, 56.38, 30.71, 26.58. ^{19}F NMR (376 MHz, $CDCl_3$) δ -127.97. HPLC (Chiralpak OD-H, elute: Hexanes/*i*-PrOH = 90/10, detector: 254 nm, flow rate: 1.0 mL/min, 25 ℃).

(*S*)-3o: (*S*)-6-bromo-2-phenyl-1,2,3,4-tetrahydroquinoline. White solid, 93% yield, 91% *ee*. ^{1}H NMR (400 MHz, $CDCl_3$) δ 7.30 – 7.18 (m, 5H), 7.02 – 6.98 (m, 2H), 6.33 – 6.31 (d, J = 8.4 Hz, 1H), 4.34 – 4.31 (dd, J = 9.1, 3.4 Hz, 1H), 2.82 – 2.74 (m, 1H), 2.63 – 2.57 (m, 1H), 2.05 – 1.98 (m, 1H), 1.91 – 1.82 (m, 1H). ^{13}C NMR (101 MHz, $CDCl_3$) δ 144.32, 143.68, 131.73, 129.58, 128.68, 127.63, 126.50, 122.97, 115.43, 108.56, 56.06, 30.40, 26.09. HPLC (Chiralpak AD-H, elute: Hexanes/*i*-PrOH = 90/10, detector: 254 nm, flow rate: 1.0 mL/min, 25 ℃).

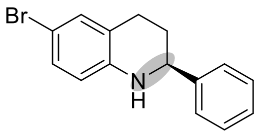

(*S*)-**3o**

(*S*)-3p: (*S*)-6-methyl-2-phenyl-1,2,3,4-tetrahydroquinoline. White solid, 95% yield, 90% *ee*. ^{1}H NMR (400 MHz, $CDCl_3$) δ 7.31 – 7.17 (m, 5H), 6.75 – 6.73 (dd, J = 5.9, 2.3 Hz, 2H), 6.39 – 6.37 (d, J = 8.5 Hz, 1H), 4.32 – 4.29 (dd, J = 9.4, 3.2 Hz, 1H), 2.85 – 2.77 (m, 1H), 2.64 – 2.58 (m, 1H), 2.15 (s, 3H), 2.05 – 1.98 (m, 1H), 1.94 – 1.85 (m, 1H). ^{13}C NMR (101 MHz, $CDCl_3$) δ 144.95, 142.37, 129.89, 128.59, 127.49, 127.44, 126.63, 126.49, 121.01, 114.23, 56.45, 31.19, 26.44, 20.51. HPLC (Chiralpak OD-H, elute: Hexanes/*i*-PrOH = 90/10, detector: 254 nm, flow rate: 1.0 mL/min, 25 ℃).

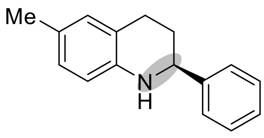

(*S*)-**3p**

(*S*)-3q: (*S*)-6-methoxy-2-phenyl-1,2,3,4-tetrahydroquinoline. White solid, 92% yield, 84% *ee*. ^{1}H NMR (400 MHz, $CDCl_3$) δ 7.32 – 7.17 (m, 5H), 6.56 – 6.52 (m, 2H), 6.42 – 6.40 (d, J = 8.4 Hz, 1H), 4.29 – 4.25 (dd, J = 9.6, 3.1 Hz, 1H), 3.65 (s, 3H), 2.89 – 2.80 (m, 1H), 2.67 – 2.60 (m, 1H), 2.05 – 1.98 (m, 1H), 1.95 – 1.85 (m, 1H). ^{13}C NMR (101 MHz, $CDCl_3$) δ 151.93, 144.85, 138.90, 128.59, 127.45, 126.65, 122.22, 115.25, 114.68, 113.08, 56.62, 55.87, 31.15, 26.87. HPLC (Chiralpak OD-H, elute: Hexanes/*i*-PrOH = 90/10, detector: 254 nm, flow rate: 1.0 mL/min, 25 ℃).

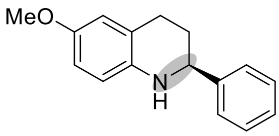

(*S*)-**3q**

(*S*)-3r: methyl (*S*)-4-(6-methoxy-1,2,3,4-tetrahydroquinolin-2-yl)benzoate. White solid, 99% yield, 99% *ee*. ^{1}H NMR (400 MHz, $CDCl_3$) δ 7.95 – 7.93 (m, 2H), 7.40 – 7.38 (d, J = 8.3 Hz, 2H), 6.59 – 6.53 (m, 2H), 6.48 – 6.46 (d, J = 8.5 Hz, 1H), 4.38 – 4.35 (dd, J = 9.3, 3.1 Hz, 1H), 3.84 (s, 3H), 3.67 (s, 3H), 2.89 – 2.81 (m, 1H), 2.66 – 2.60 (m, 1H), 2.07 – 2.01 (m, 1H), 1.95 – 1.86 (m, 1H). ^{13}C NMR (101 MHz, $CDCl_3$) δ 166.98, 152.13, 150.06, 138.28, 129.92, 129.26, 126.60, 122.17, 115.41, 114.63, 113.16, 56.30, 55.83, 52.14, 30.96, 26.49. HPLC (Chiralpak OD-H, elute: Hexanes/*i*-PrOH = 90/10, detector: 254 nm, flow rate: 1.0 mL/min, 25 ℃).

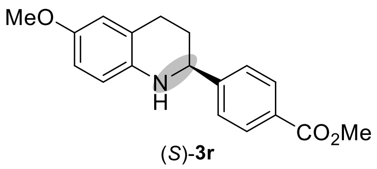

(*S*)-**3r**

Figure S1. ^{1}H–NMR and HPLC spectra of (*R*)-**1a** in the reactions of *rac*-**1a**-d_1 in Table 2

General Note. The ^{1}H–NMR spectra of *rac*-**1a-*d*** (starting material) before reaction *and (R)*-**1a** (chiral products) after deracemization, and the HPLC spectrum of *rac*-**1a**.

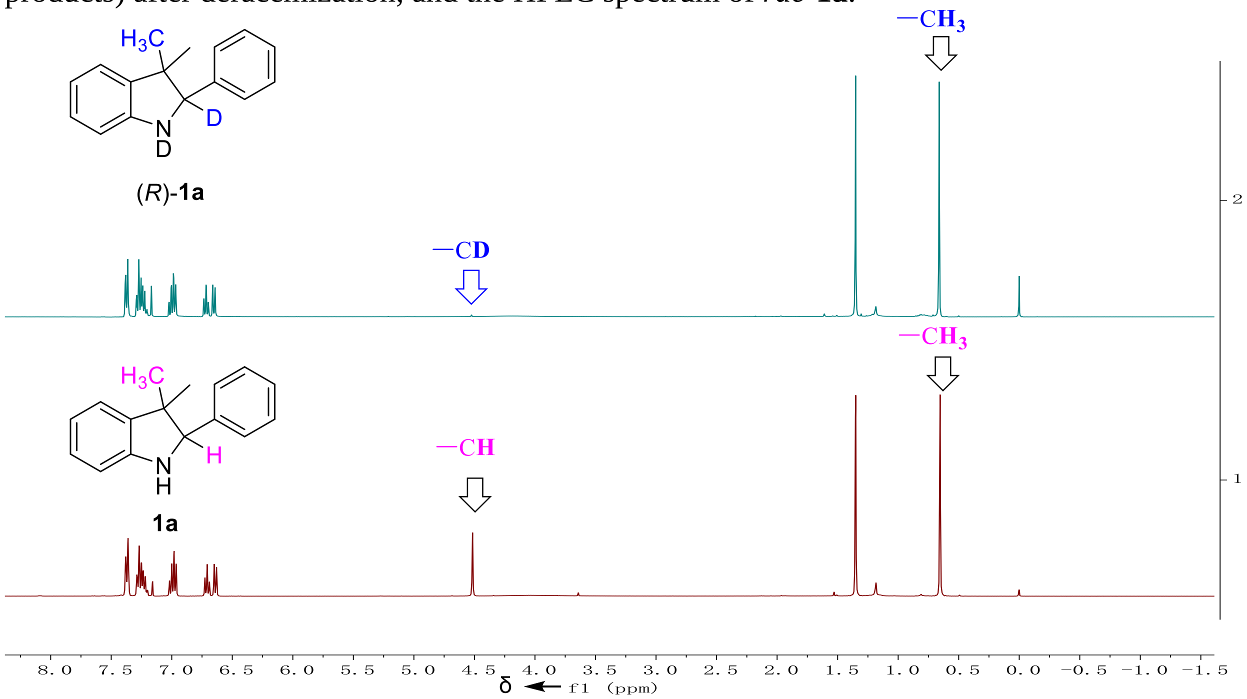

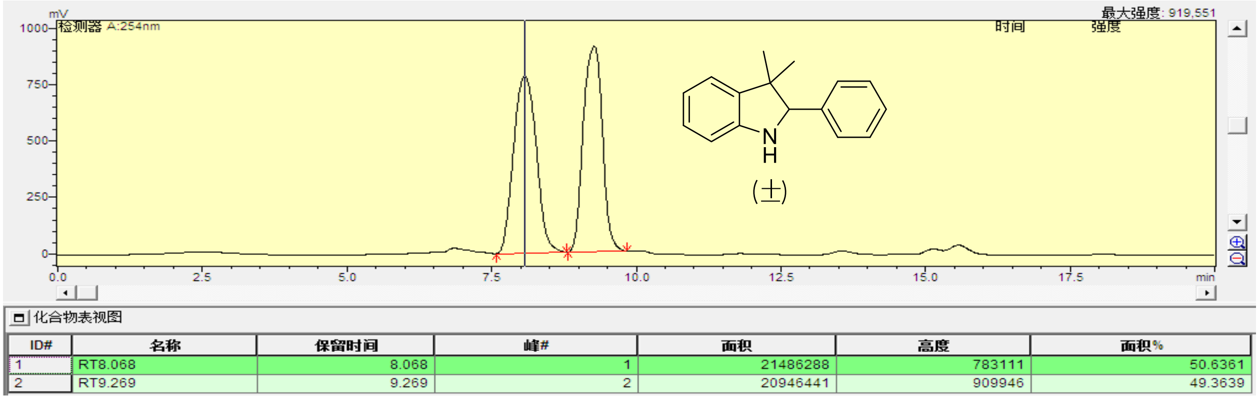

ID#	名称	保留时间	峰#	面积	高度	面积%
1	RT8.068	8.068	1	21486288	783111	50.6361
2	RT9.269	9.269	2	20946441	909946	49.3639

Translation of all characters (Chinese) in the above framework to English is as follows:

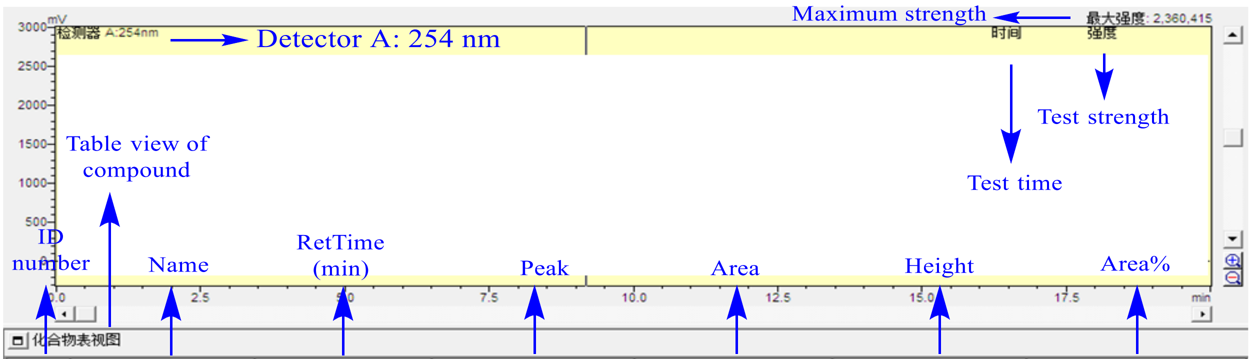

ID#	名称	保留时间	峰#	面积	高度	面积%
1	RT9.190	9.190	1	39625295	2360461	47.0509
2	RT11.859	11.859	2	44592689	2144002	52.9491

Entry 2 in Table 2 for **A** and **CPA-1** as catalysts and HTE-1 as an H-atom transfer reagent in the only aqueous β-cyclodextrin emulsion.

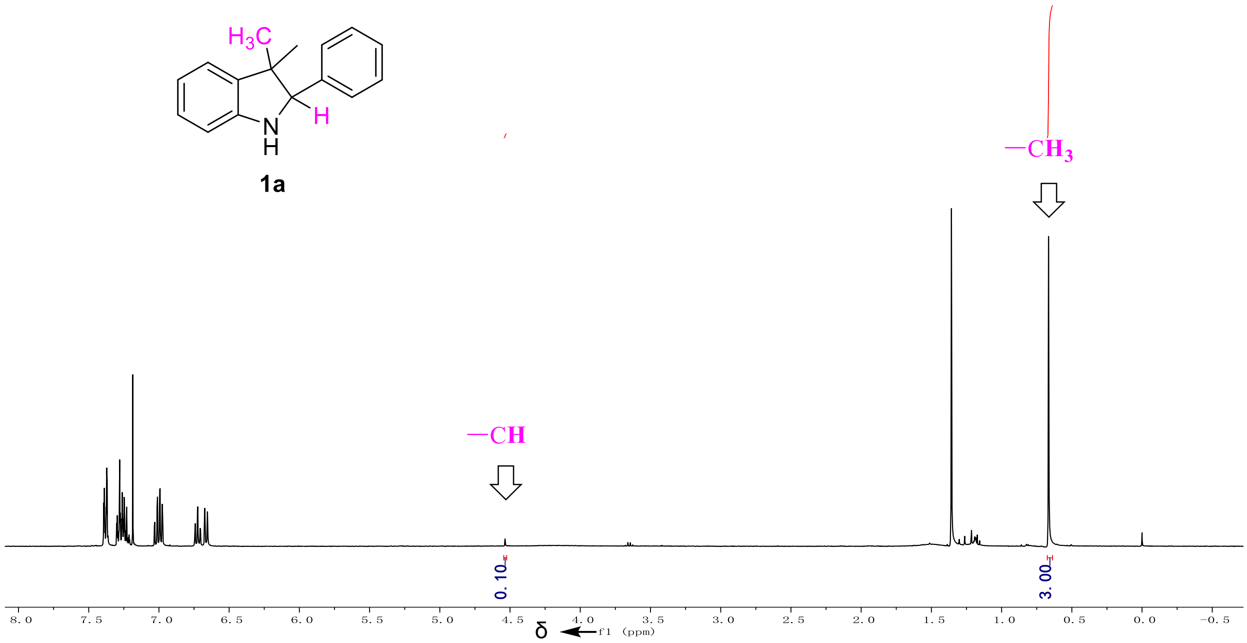

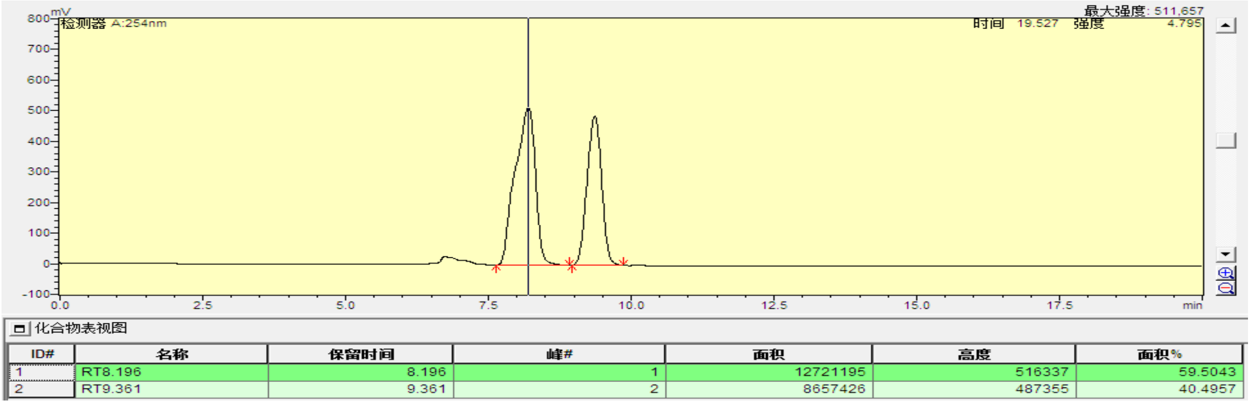

ID#	名称	保留时间	峰#	面积	高度	面积%
1	RT8.196	8.196	1	12721195	516337	59.5043
2	RT9.361	9.361	2	8657426	487355	40.4957

Translation of all characters (Chinese) in the above framework to English is as follows:

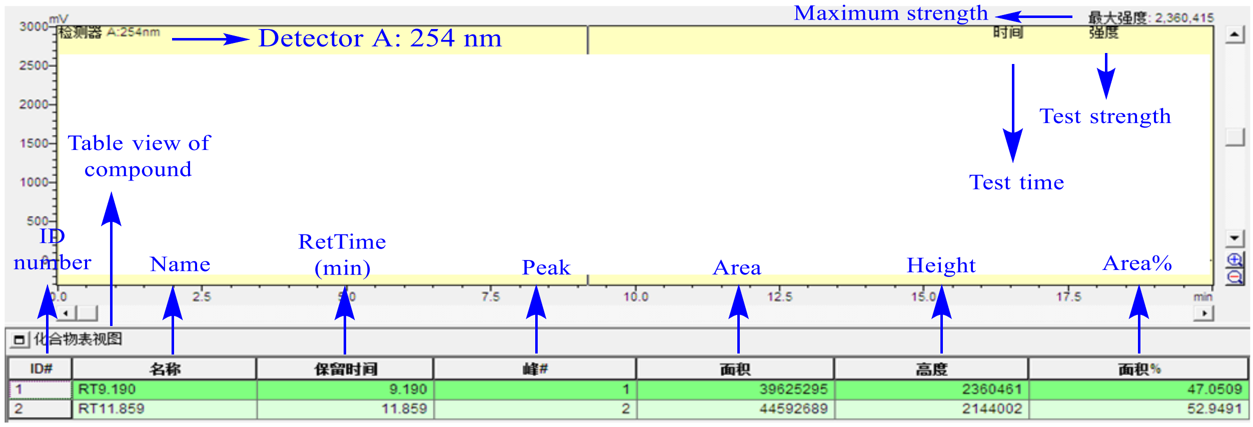

ID#	名称	保留时间	峰#	面积	高度	面积%
1	RT9.190	9.190	1	39625295	2360461	47.0509
2	RT11.859	11.859	2	44592689	2144002	52.9491

Entry 3 in Table 2 for **A** and **CPA-1** as catalysts and HTE-1 as an H-atom transfer reagent in the toluene/aqueous β-cyclodextrin emulsion (v/v = 1/1) cosolvent.

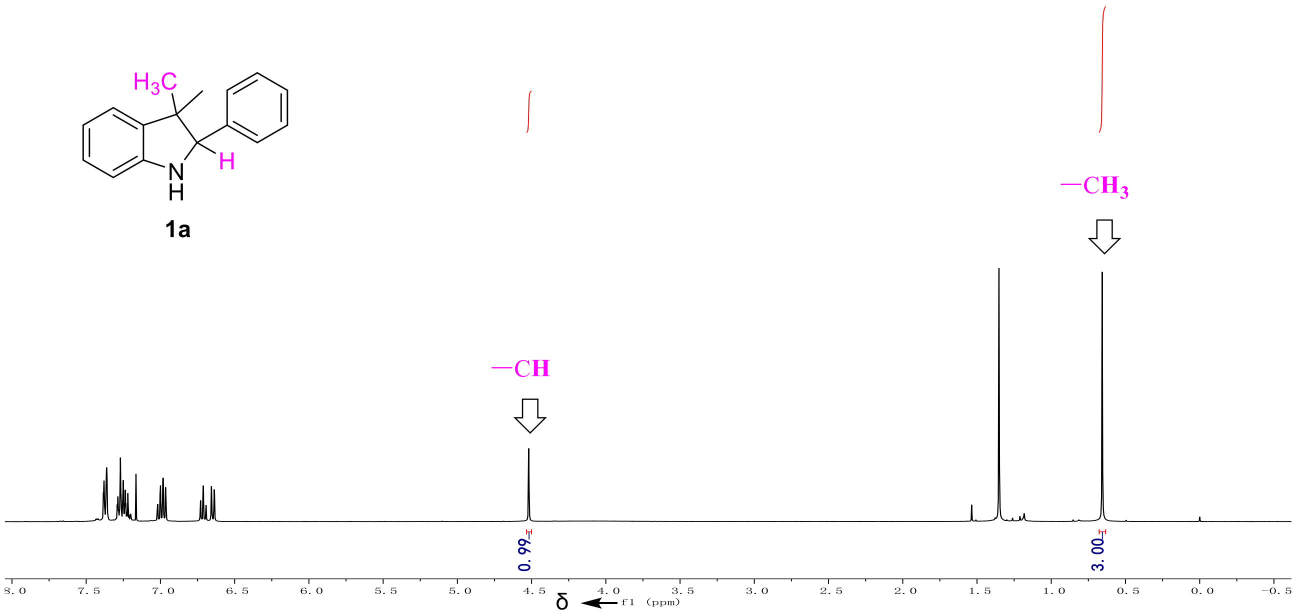

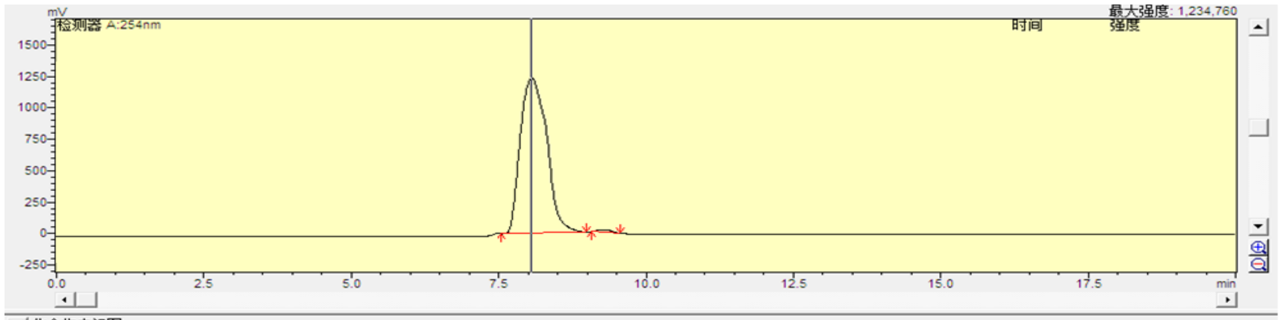

ID#	名称	保留时间	峰#	面积	高度	面积%
1	RT8.057	8.057	1	38492491	1232030	99.0999
2	RT9.289	9.289	2	349599	21559	0.9001

<u>Translation of all characters (Chinese) in the above framework to English is as follows:</u>

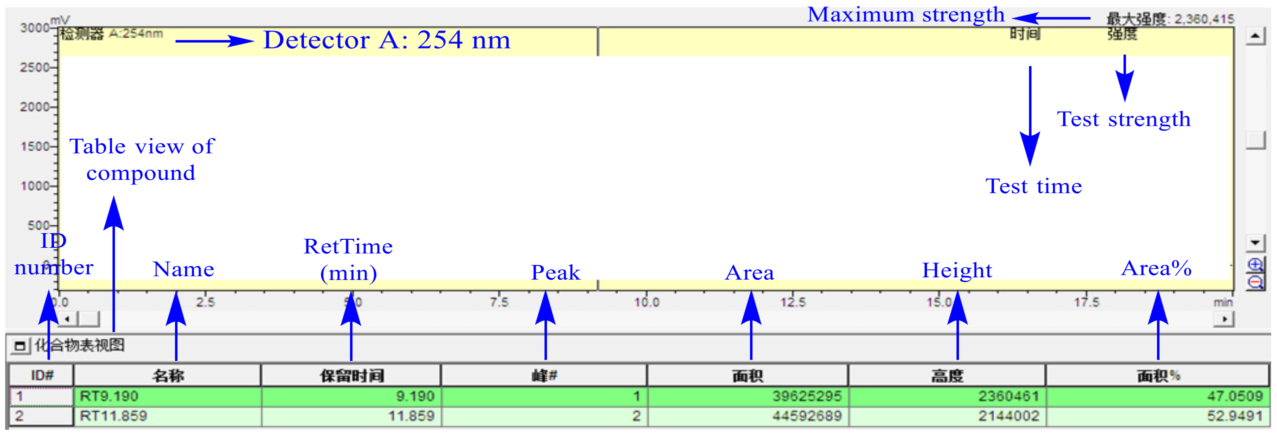

ID#	名称	保留时间	峰#	面积	高度	面积%
1	RT9.190	9.190	1	39625295	2360461	47.0509
2	RT11.859	11.859	2	44592689	2144002	52.9491

Entry 4 in Table 2 for **A** and **CPA-1** as catalysts and HTE-2 as an H-atom transfer reagent in the toluene/aqueous β-cyclodextrin emulsion (v/v = 1/1) cosolvent.

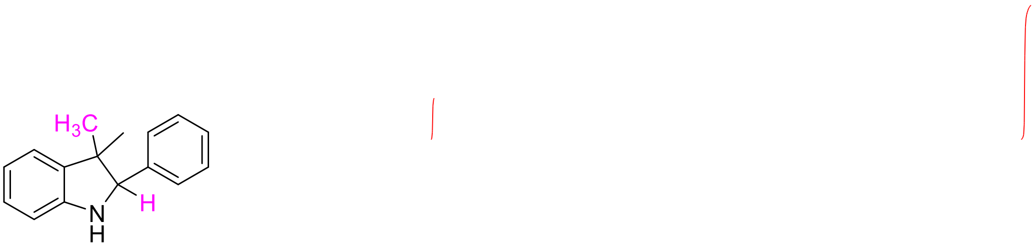

1a

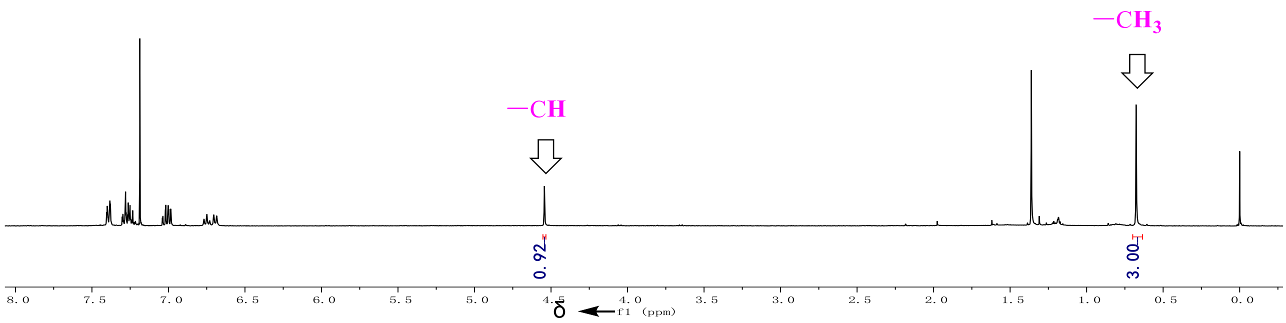

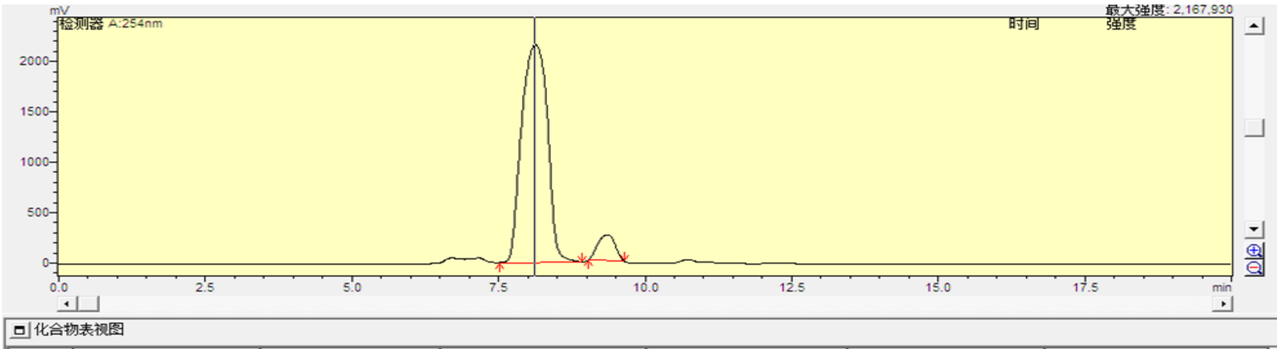

ID#	名称	保留时间	峰#	面积	高度	面积%
1	RT8.124	8.124	1	66192508	2159771	92.6418
2	RT9.356	9.356	2	5257398	255175	7.3582

Translation of all characters (Chinese) in the above framework to English is as follows:

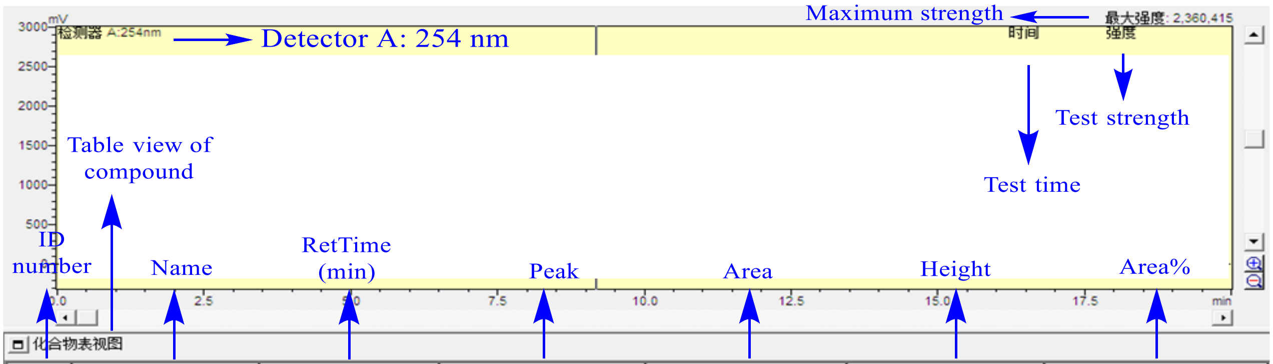

ID#	名称	保留时间	峰#	面积	高度	面积%
1	RT9.190	9.190	1	39625295	2360461	47.0509
2	RT11.859	11.859	2	44592689	2144002	52.9491

Entry 5 in Table 2 for **A** and **CPA-1** as catalysts and HTE-3 as an H-atom transfer reagent in the toluene/aqueous β-cyclodextrin emulsion (v/v = 1/1) cosolvent.

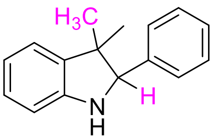

1a

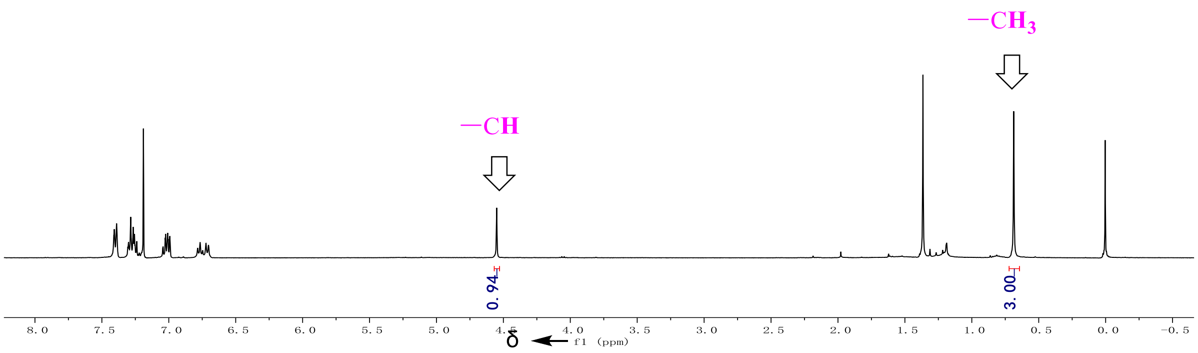

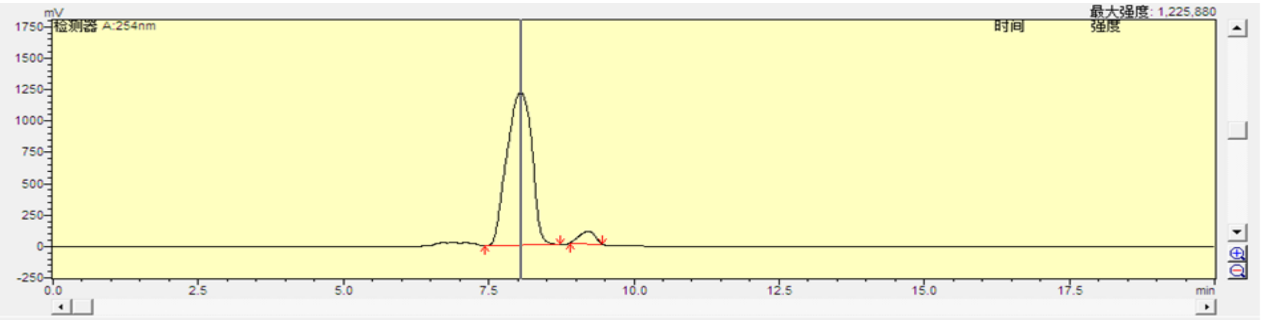

ID#	名称	保留时间	峰#	面积	高度	面积%
1	RT8.048	8.048	1	36463027	1215966	95.2008
2	RT9.223	9.223	2	1838147	98355	4.7992

Translation of all characters (Chinese) in the above framework to English is as follows:

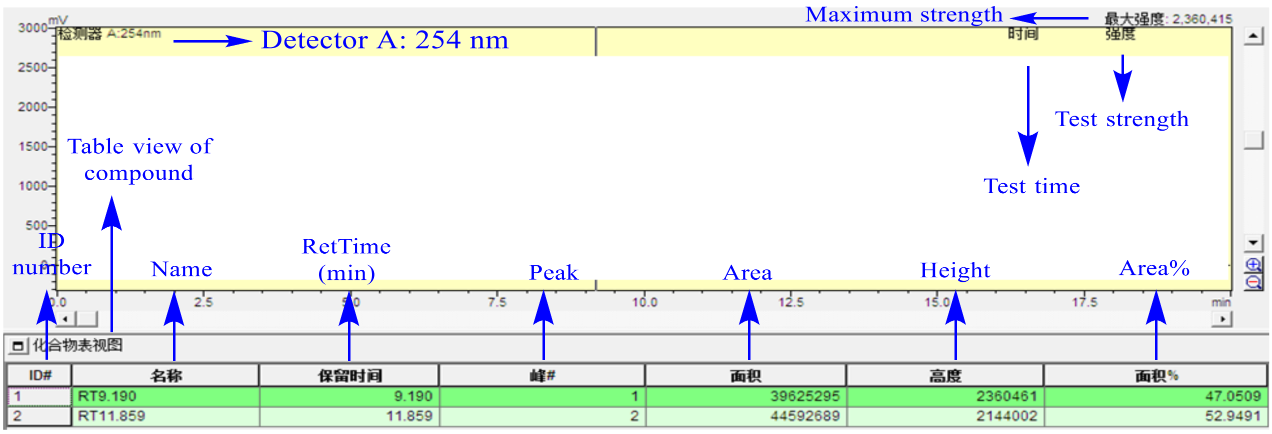

ID#	名称	保留时间	峰#	面积	高度	面积%
1	RT9.190	9.190	1	39625295	2360461	47.0509
2	RT11.859	11.859	2	44592689	2144002	52.9491

Entry 6 in Table 2 for **A** and **CPA-1** as catalysts and HTE-4 as a H-atom transfer reagent in the toluene/aqueous β-cyclodextrin emulsion (v/v = 1/1) cosolvent.

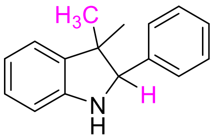

1a

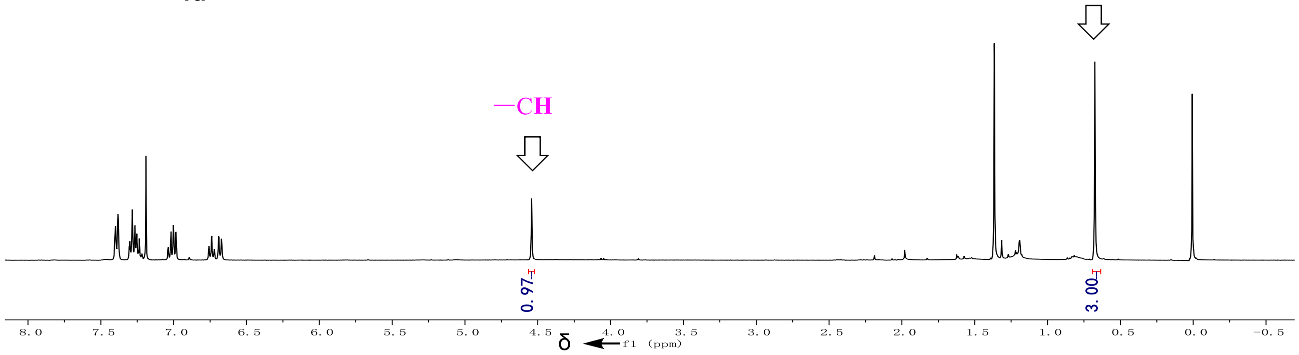

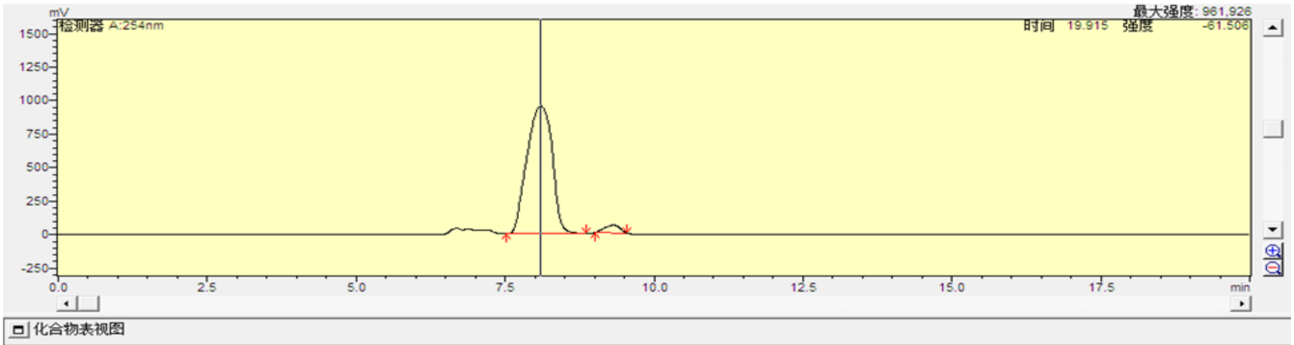

ID#	名称	保留时间	峰#	面积	高度	面积%
1	RT8.093	8.093	1	28100807	954229	96.5511
2	RT9.315	9.315	2	1003796	56281	3.4489

Translation of all characters (Chinese) in the above framework to English is as follows:

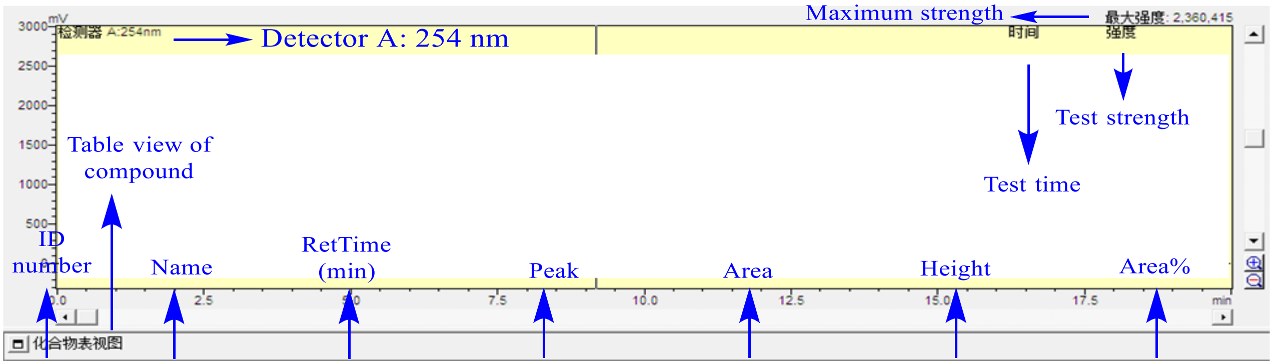

ID#	名称	保留时间	峰#	面积	高度	面积%
1	RT9.190	9.190	1	39625295	2360461	47.0509
2	RT11.859	11.859	2	44592689	2144002	52.9491

Entry 7 in Table 2 for **A** and **CPA-1** as catalysts and HTE-1 as an H-atom transfer reagent in the toluene/aqueous β-cyclodextrin emulsion (v/v = 3/2) cosolvent.

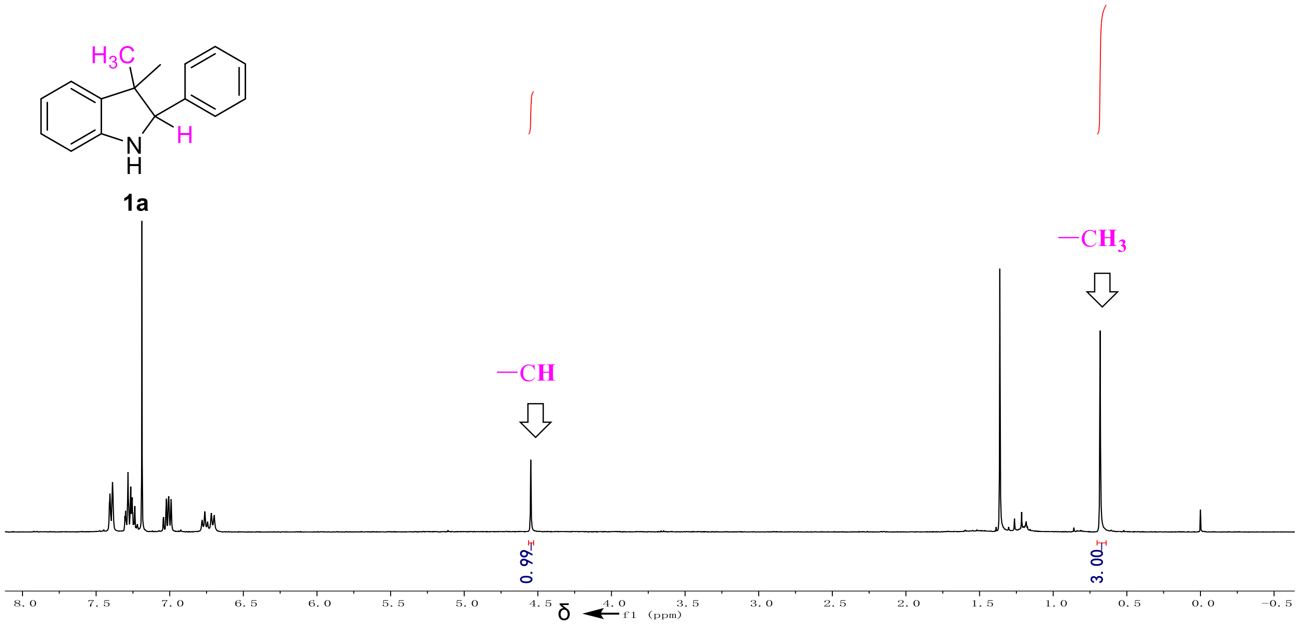

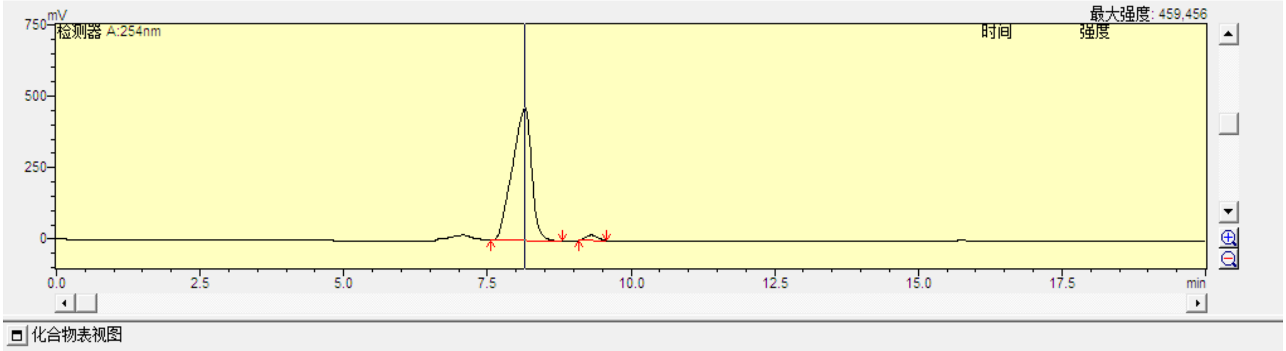

ID#	名称	保留时间	峰#	面积	高度	面积%
1	RT8.149	8.149	1	10757138	465121	97.5526
2	RT9.306	9.306	2	269880	18545	2.4474

<u>Translation of all characters (Chinese) in the above framework to English is as follows:</u>

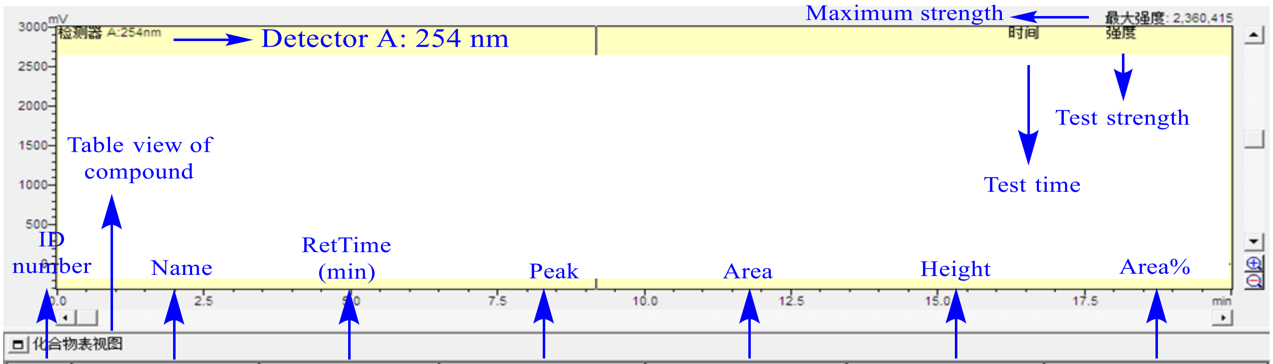

ID#	名称	保留时间	峰#	面积	高度	面积%
1	RT9.190	9.190	1	39625295	2360461	47.0509
2	RT11.859	11.859	2	44592689	2144002	52.9491

Entry 8 in Table 2 for **A** and **CPA-1** as catalysts and HTE-1 as an H-atom transfer reagent in the toluene/aqueous β-cyclodextrin emulsion (v/v = 2/3) cosolvent.

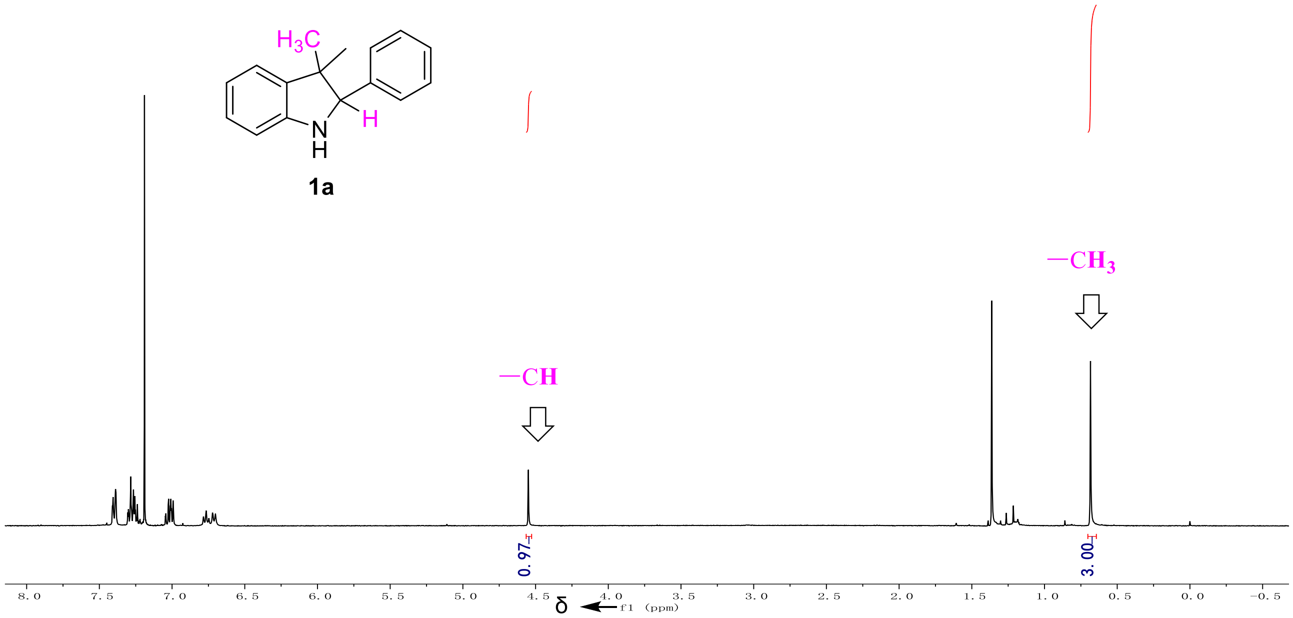

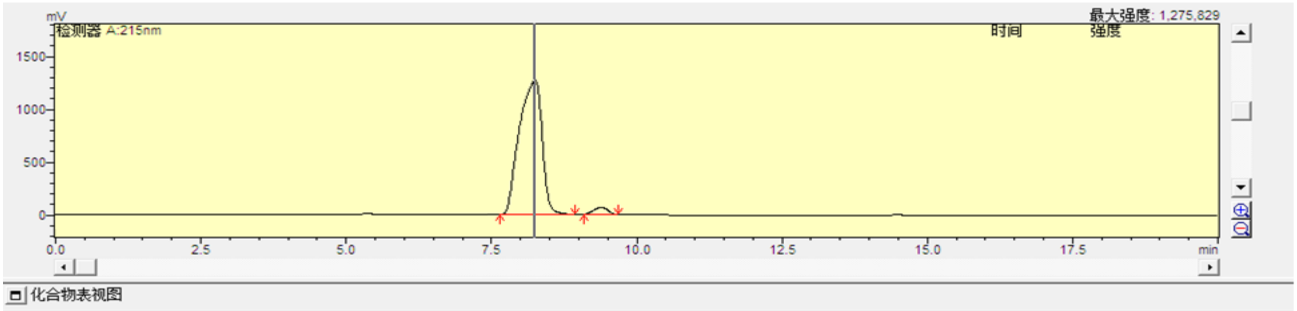

ID#	名称	保留时间	峰#	面积	高度	面积%
1	RT8.244	8.244	1	34636215	1271761	96.7986
2	RT9.387	9.387	2	1145507	67760	3.2014

Translation of all characters (Chinese) in the above framework to English is as follows:

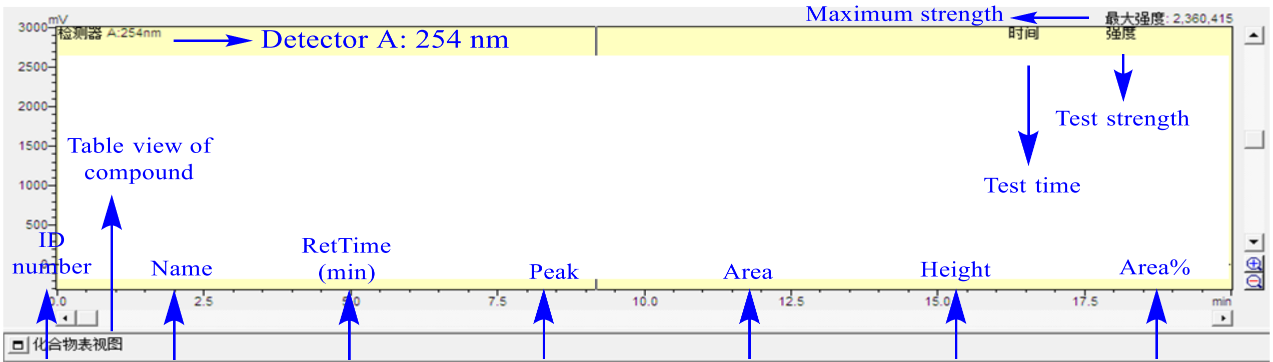

ID#	名称	保留时间	峰#	面积	高度	面积%
1	RT9.190	9.190	1	39625295	2360461	47.0509
2	RT11.859	11.859	2	44592689	2144002	52.9491

Entry 9 in Table 2 for **A** and **CPA-2** as catalysts and HTE-1 as an H-atom transfer reagent in the toluene/aqueous β-cyclodextrin emulsion (v/v = 1/1) cosolvent.

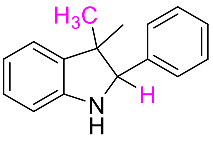

1a

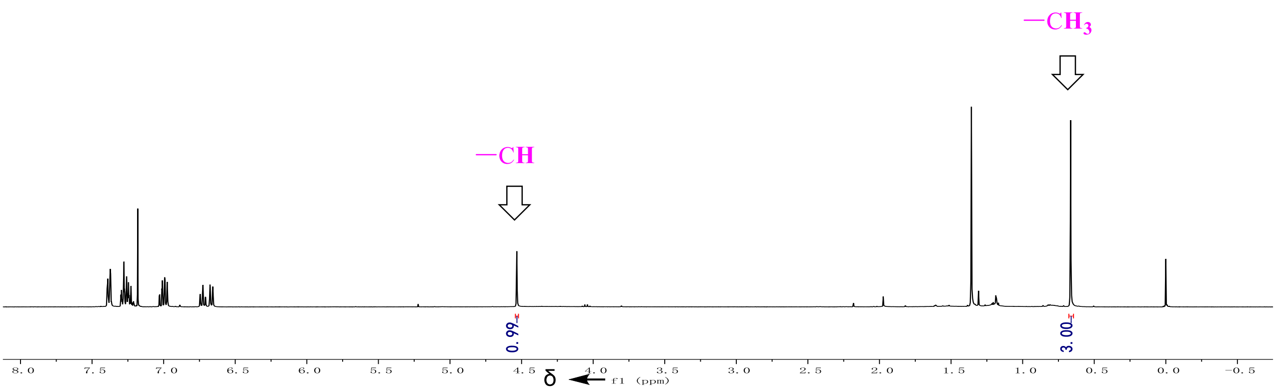

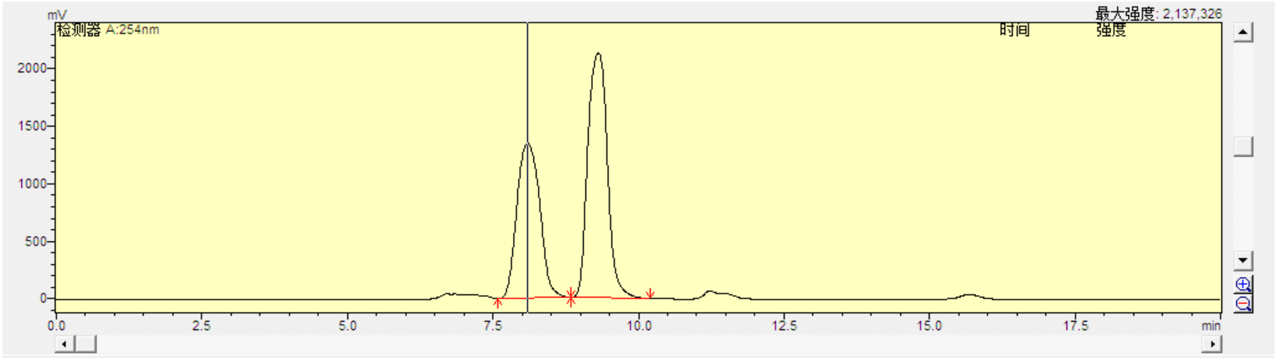

□ 化合物表视图

ID#	名称	保留时间	峰#	面积	高度	面积%
1	RT8.092	8.092	1	36744189	1344270	42.5101
2	RT9.309	9.309	2	49692211	2128473	57.4899

Translation of all characters (Chinese) in the above framework to English is as follows:

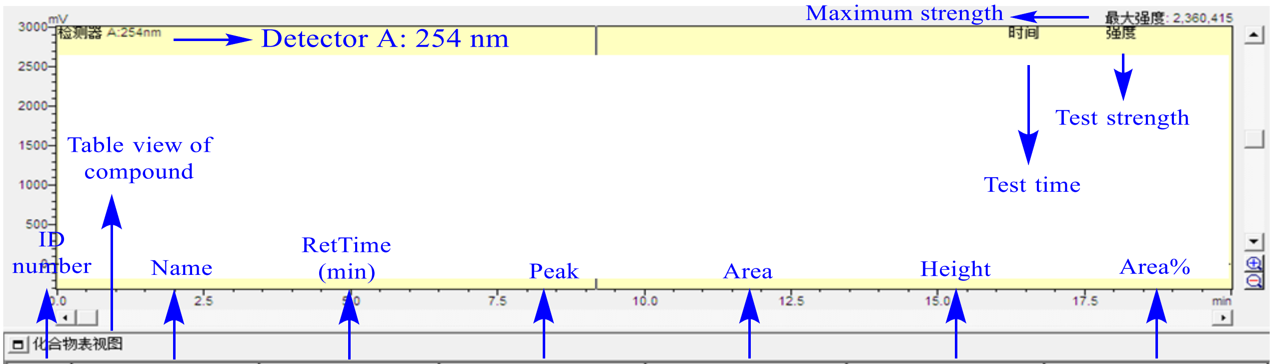

ID#	名称	保留时间	峰#	面积	高度	面积%
1	RT9.190	9.190	1	39625295	2360461	47.0509
2	RT11.859	11.859	2	44592689	2144002	52.9491

Entry 10 in Table 2 for **A** and **CPA-3** as catalysts and HTE-1 as an H-atom transfer reagent in the toluene/aqueous β-cyclodextrin emulsion (v/v = 1/1) cosolvent

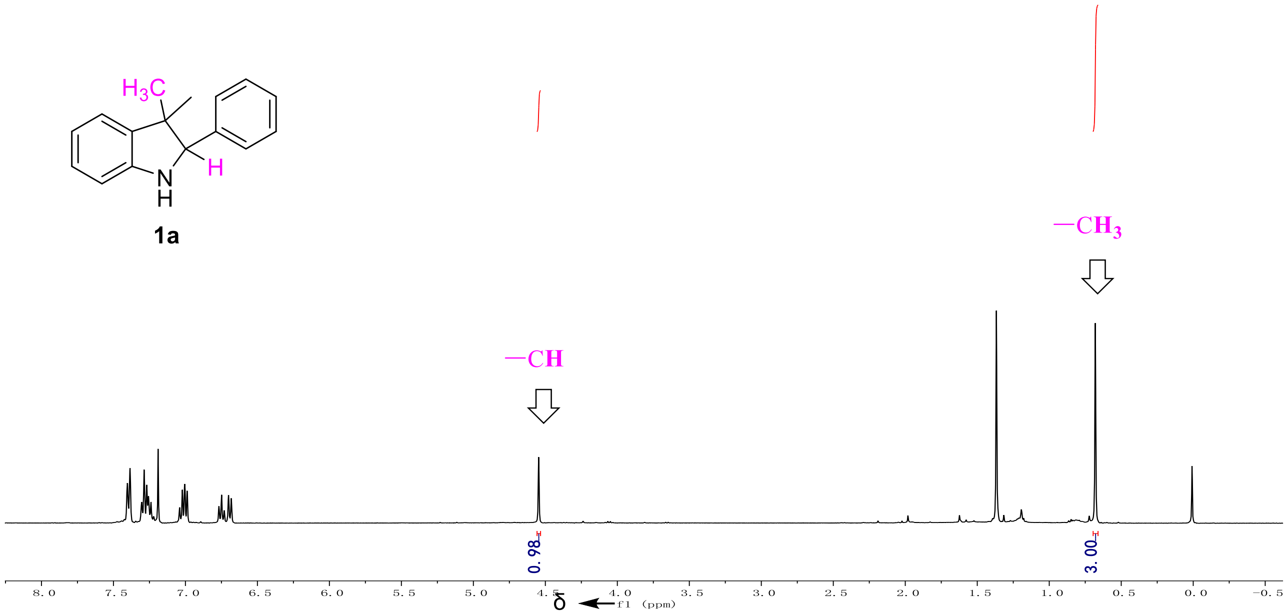

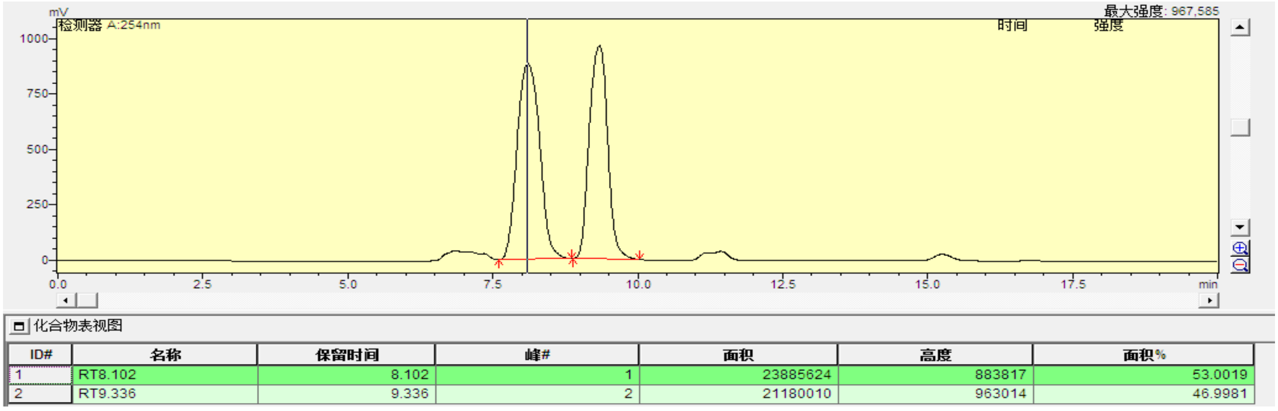

ID#	名称	保留时间	峰#	面积	高度	面积%
1	RT8.102	8.102	1	23885624	883817	53.0019
2	RT9.336	9.336	2	21180010	963014	46.9981

Translation of all characters (Chinese) in the above framework to English is as follows:

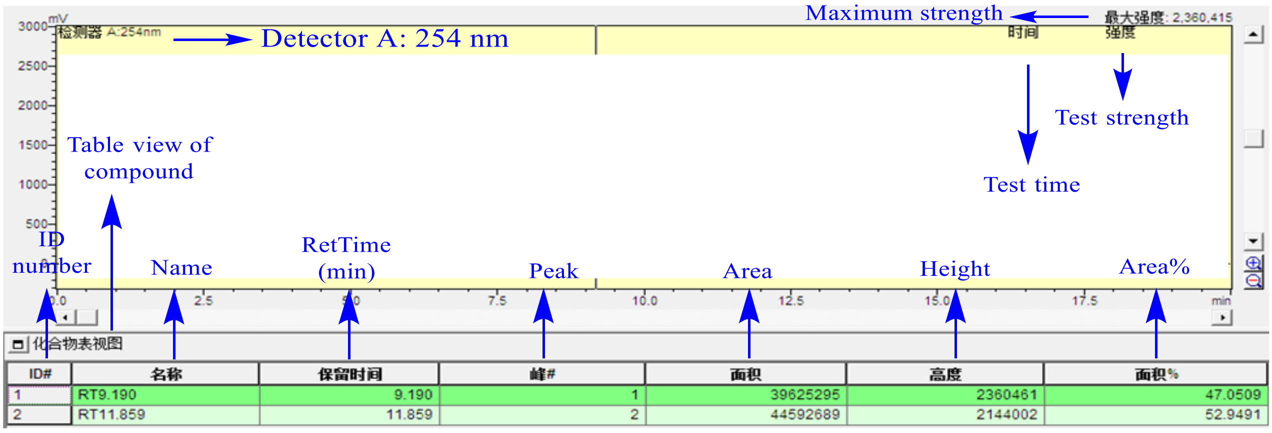

ID#	名称	保留时间	峰#	面积	高度	面积%
1	RT9.190	9.190	1	39625295	2360461	47.0509
2	RT11.859	11.859	2	44592689	2144002	52.9491

Figure S2. GC analysis of H_2 in the single-step oxidations of *rac*-**1a** in toluene.

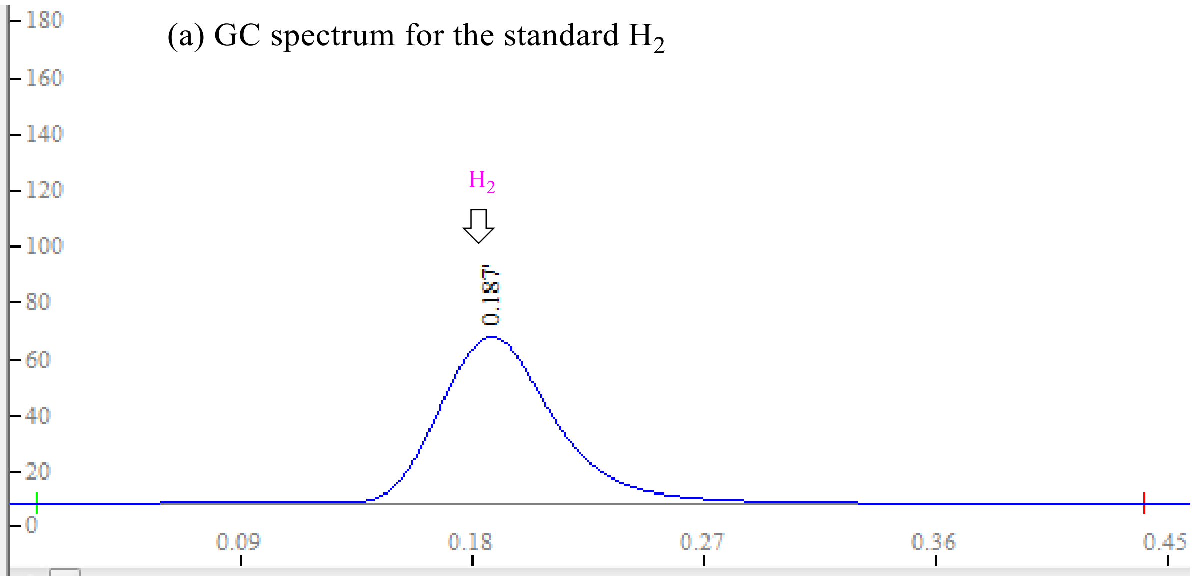

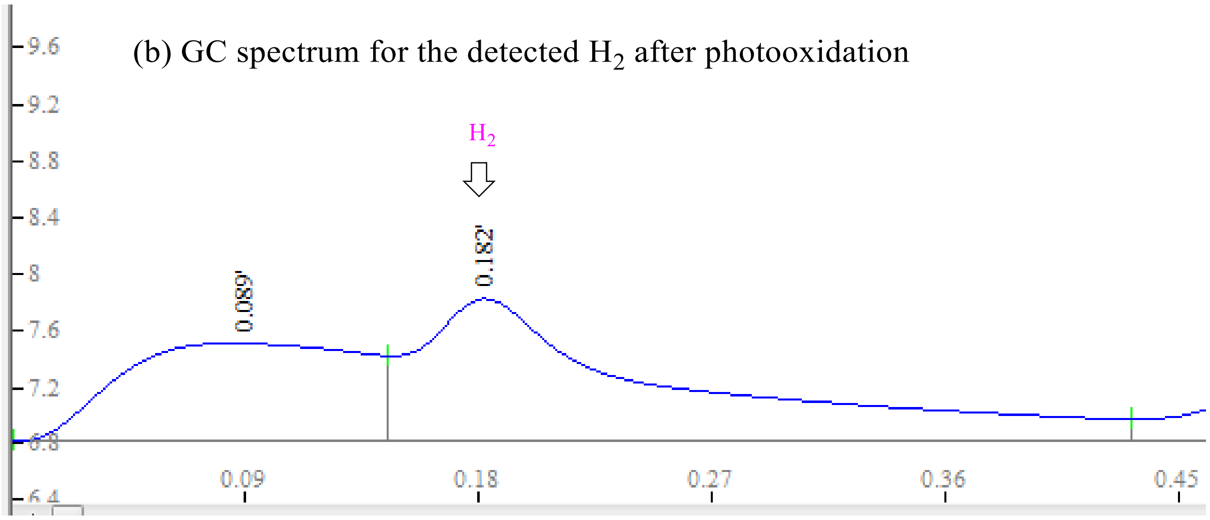

Figure S3. ^{1}H-NMR spectra of **2a**, CPA-1, and **2a**+CPA (mole ratio = 1:1) in CDCl3.

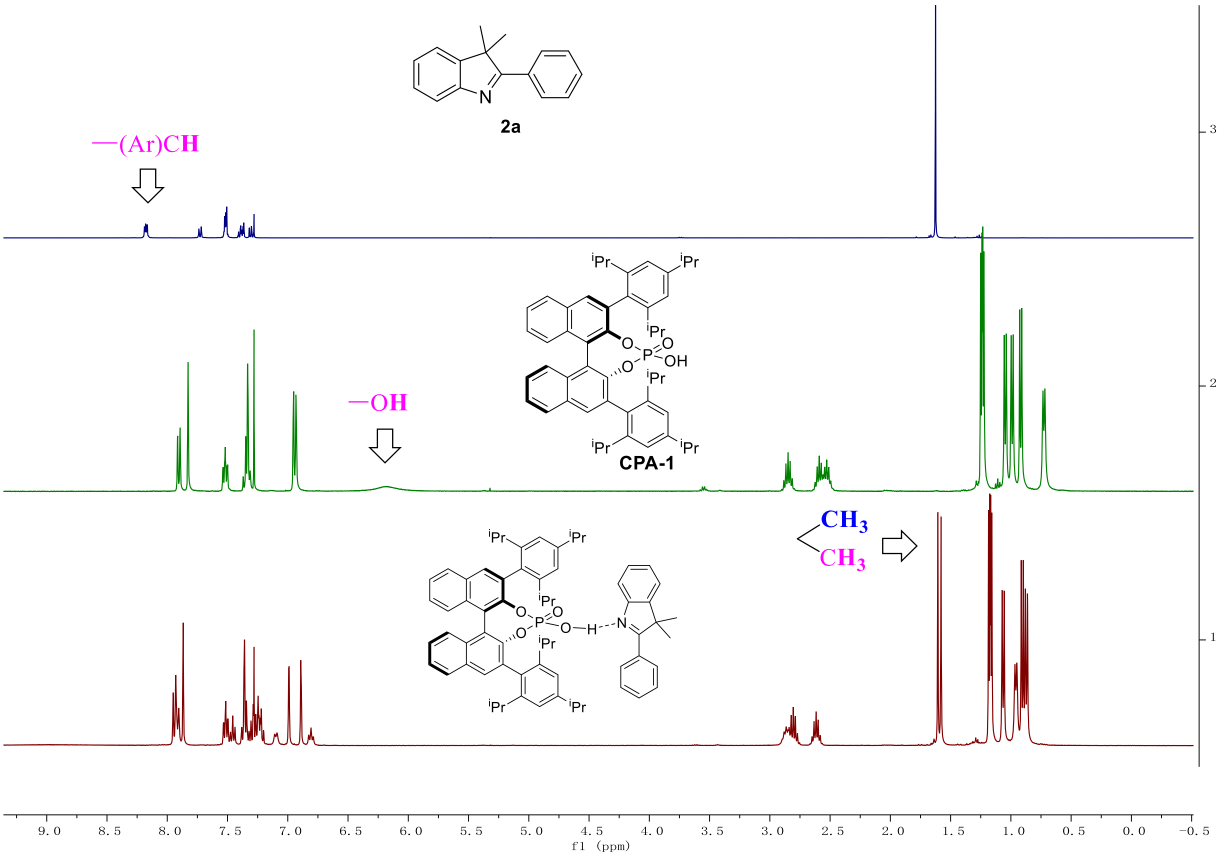

Figure S4. HPLC analyses for chiral products.

(*R*)-1a：**(R)-3,3-dimethyl-2-phenylindoline**：(HPLC: Chiracel IC-H, detected at 254 nm, eluent: n-hexane/2-propanol = 97/03, flow rate = 0.5mL/min, 25 ℃).

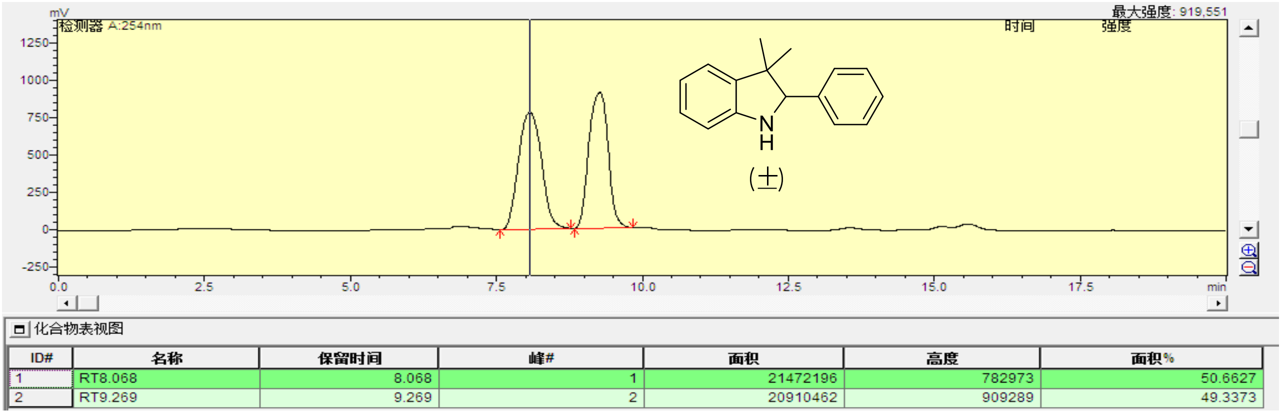

ID#	名称	保留时间	峰#	面积	高度	面积%
1	RT8.068	8.068	1	21472196	782973	50.6627
2	RT9.269	9.269	2	20910462	909289	49.3373

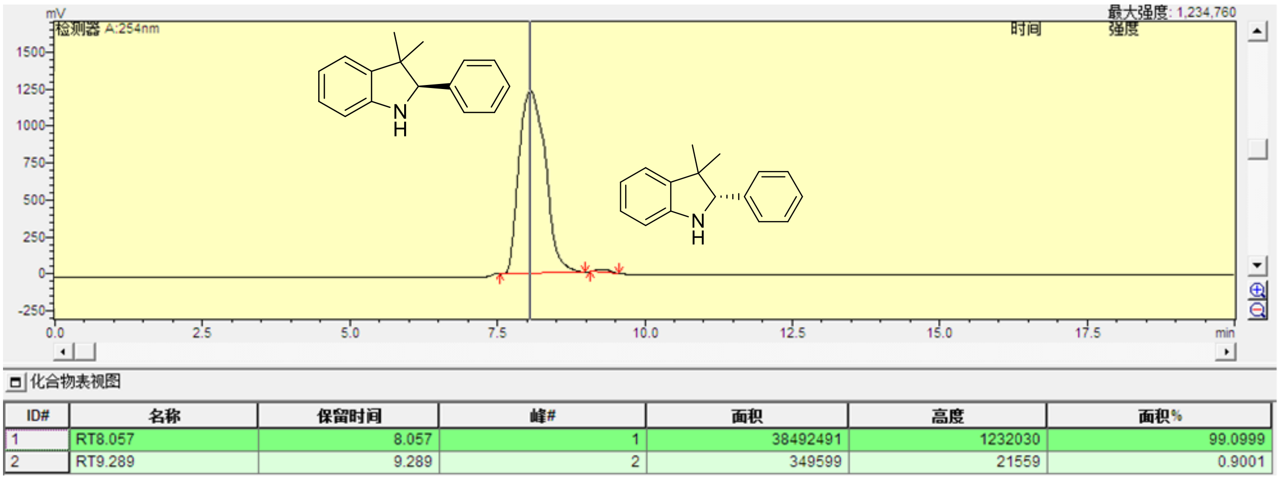

ID#	名称	保留时间	峰#	面积	高度	面积%
1	RT8.057	8.057	1	38492491	1232030	99.0999
2	RT9.289	9.289	2	349599	21559	0.9001

Translation of all characters (Chinese) in the above two frameworks to English is as follows:

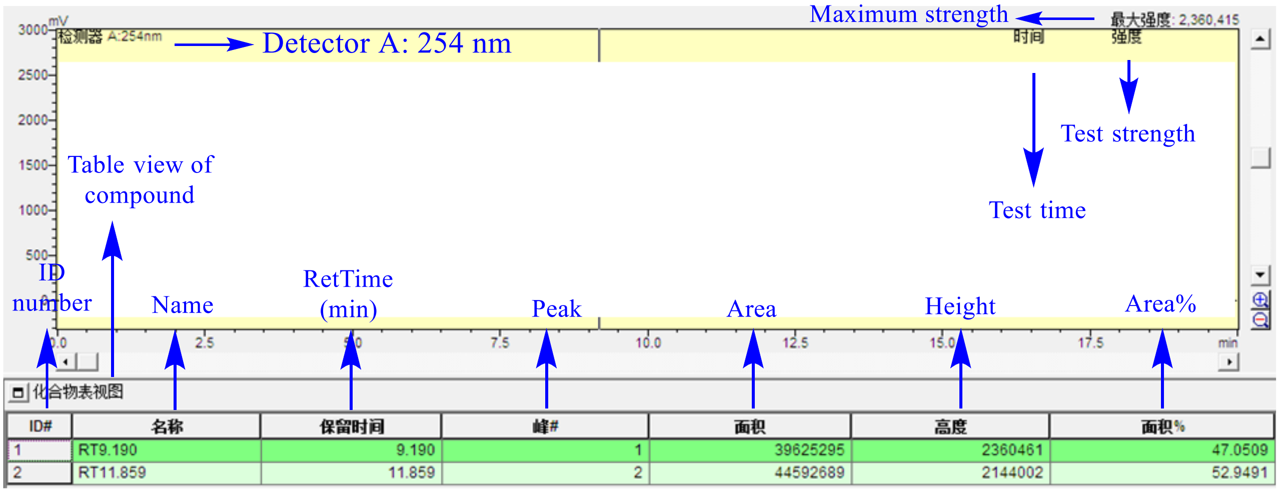

ID#	名称	保留时间	峰#	面积	高度	面积%
1	RT9.190	9.190	1	39625295	2360461	47.0509
2	RT11.859	11.859	2	44592689	2144002	52.9491

(*R*)-1b: (R)- 5-fluoro-3,3-dimethyl-2-phenylindoline: (HPLC: Chiracel IC-H, detected at 254 nm, eluent: n-hexane/2-propanol = 97/03, flow rate = 1.0mL/min, 25 ℃).

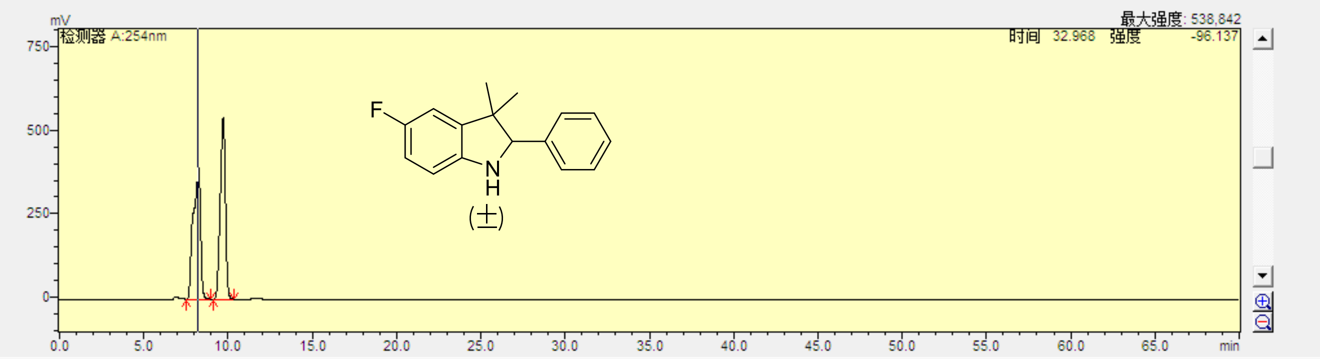

化合物表视图

ID#	名称	保留时间	峰#	面积	高度	面积%
1	RT8.241	8.241	1	11799282	391090	50.0964
2	RT9.711	9.711	2	11753857	544709	49.9036

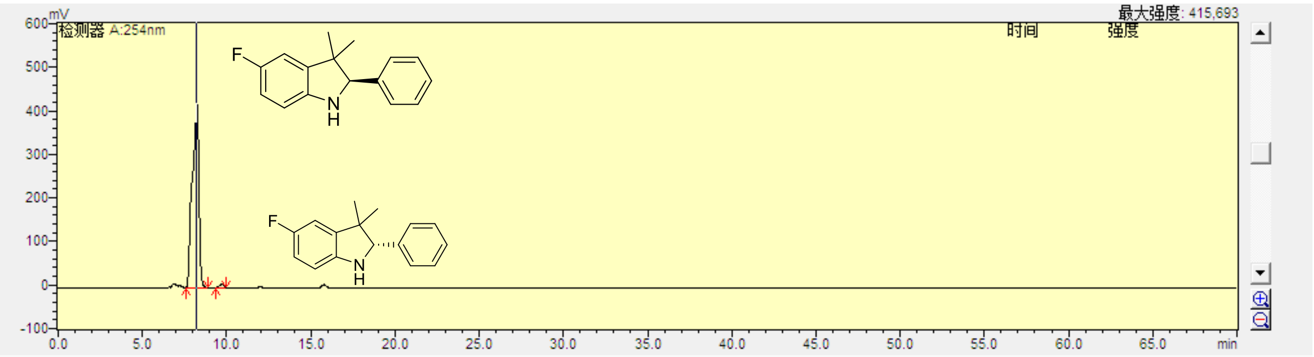

化合物表视图

ID#	名称	保留时间	峰#	面积	高度	面积%
1	RT8.237	8.237	1	11586867	421594	98.7384
2	RT9.708	9.708	2	148046	8229	1.2616

<u>Translation of all characters (Chinese) in the above two frameworks to English is as follows:</u>

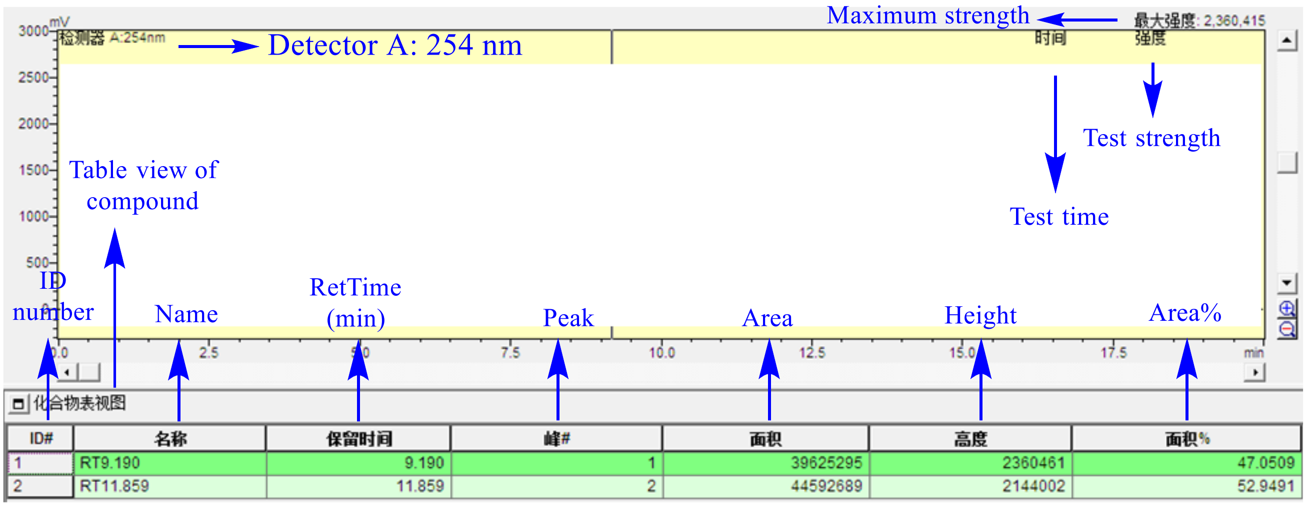

ID#	名称	保留时间	峰#	面积	高度	面积%
1	RT9.190	9.190	1	39625295	2360461	47.0509
2	RT11.859	11.859	2	44592689	2144002	52.9491

(***R***)**-1c: (R)-4-chloro-3,3-dimethyl-2-phenylindoline:** (HPLC: Chiracel OD-H, detected at 254 nm, eluent: n-hexane/2-propanol = 90/10, flow rate = 1.0mL/min, 25 ℃).

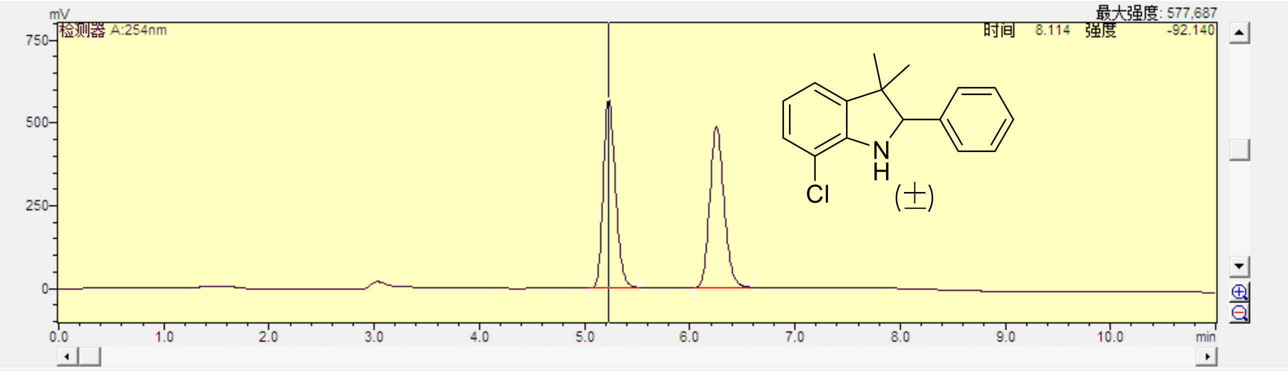

化合物表视图

ID#	名称	保留时间	峰#	面积	高度	面积%
1	RT5.226	5.226	1	4522419	575523	49.8532
2	RT6.248	6.248	2	4549059	487650	50.1468

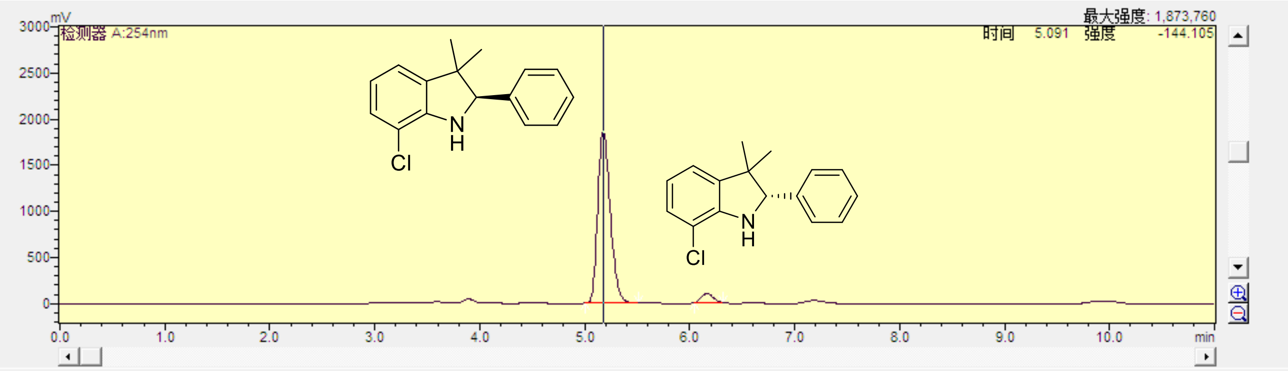

化合物表视图

ID#	名称	保留时间	峰#	面积	高度	面积%
1	RT5.174	5.174	1	15515716	1870529	95.3608
2	RT6.159	6.159	2	754828	95783	4.6392

Translation of all characters (Chinese) in the above two frameworks to English is as follows:

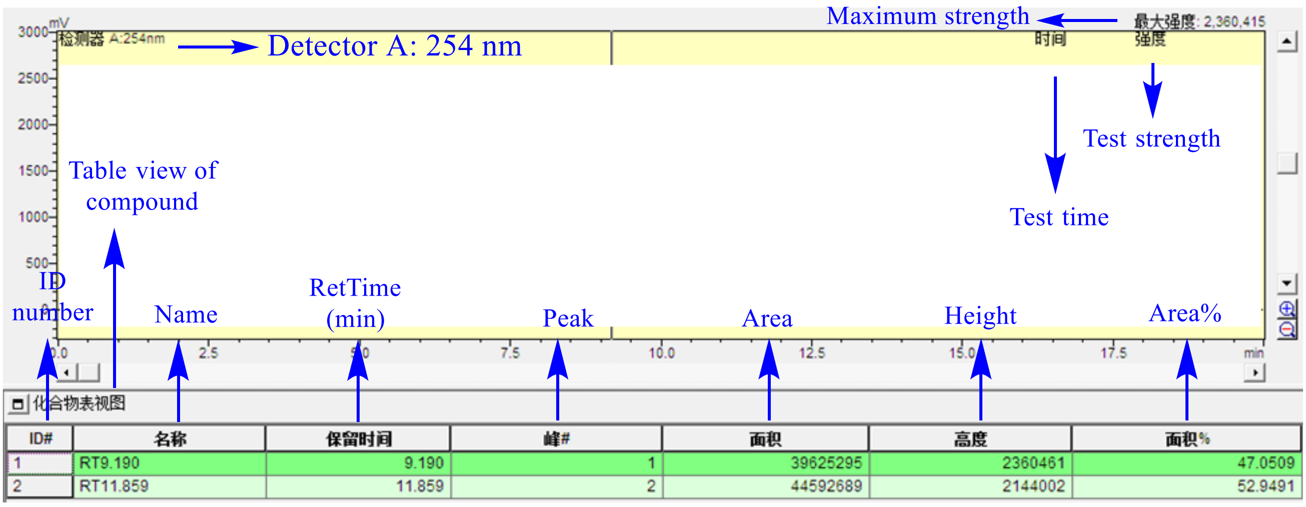

ID#	名称	保留时间	峰#	面积	高度	面积%
1	RT9.190	9.190	1	39625295	2360461	47.0509
2	RT11.859	11.859	2	44592689	2144002	52.9491

(*R*)-1d：(R)-5-chloro-3,3-dimethyl-2-phenylindoline：(HPLC: Chiracel IC-H, detected at 254 nm, eluent: n-hexane/2-propanol = 97/03, flow rate = 0.5mL/min, 25 ℃).

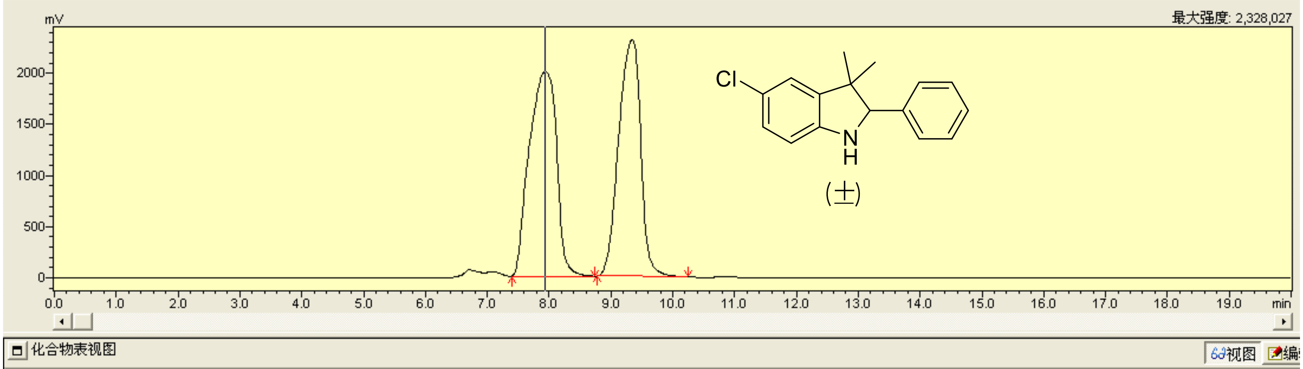

ID#	名称	保留时间	峰#	面积	高度	面积%
1	RT7.934	7.934	1	61411888	2001564	51.6924
2	RT9.342	9.342	2	57390692	2314347	48.3076

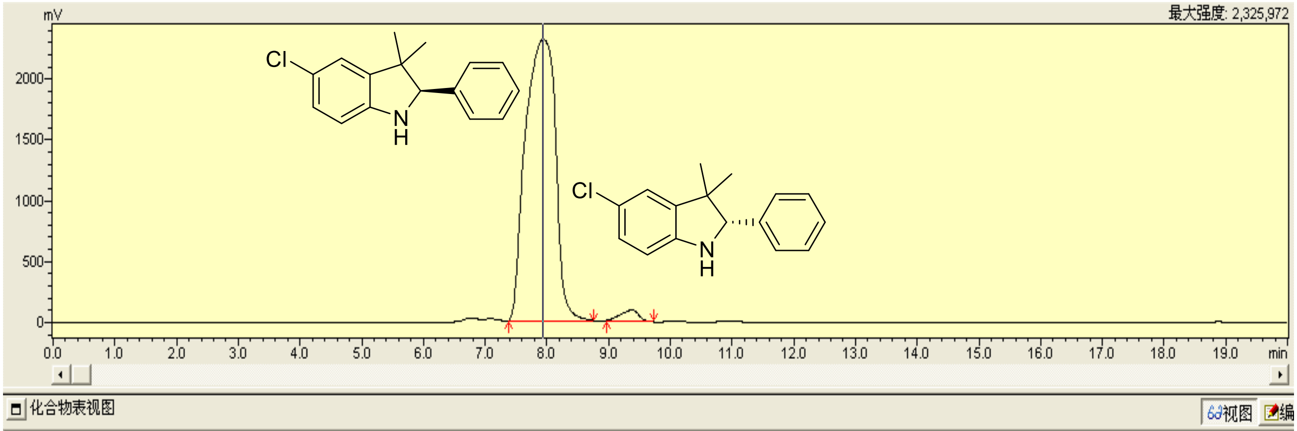

ID#	名称	保留时间	峰#	面积	高度	面积%
1	RT7.937	7.937	1	78607652	2312133	97.6684
2	RT9.372	9.372	2	1876564	93540	2.3316

Translation of all characters (Chinese) in the above two frameworks to English is as follows:

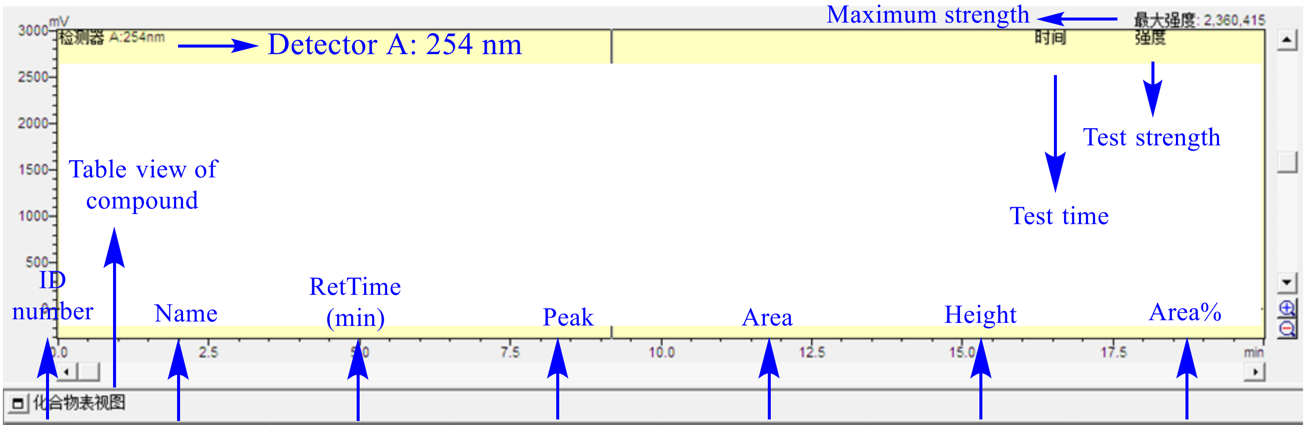

ID#	名称	保留时间	峰#	面积	高度	面积%
1	RT9.190	9.190	1	39625295	2360461	47.0509
2	RT11.859	11.859	2	44592689	2144002	52.9491

(*R*)-1e: (R)-5-bromo-3,3-dimethyl-2-phenylindoline： (HPLC: Chiracel IC-H, detected at 254 nm, eluent: n-hexane/2-propanol = 97/03, flow rate = 0.5mL/min, 25 ℃).

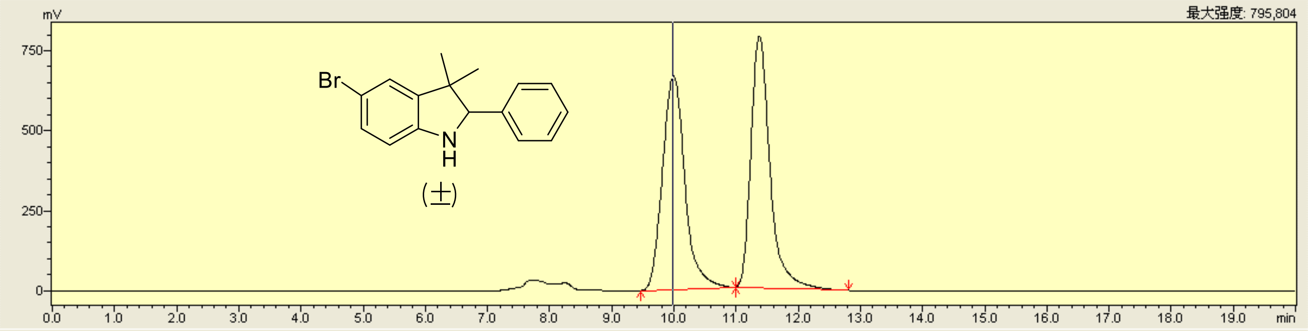

化合物表视图

ID#	名称	保留时间	峰#	面积	高度	面积%
1	RT9.989	9.989	1	16687284	665777	49.9165
2	RT11.370	11.370	2	16743118	786539	50.0835

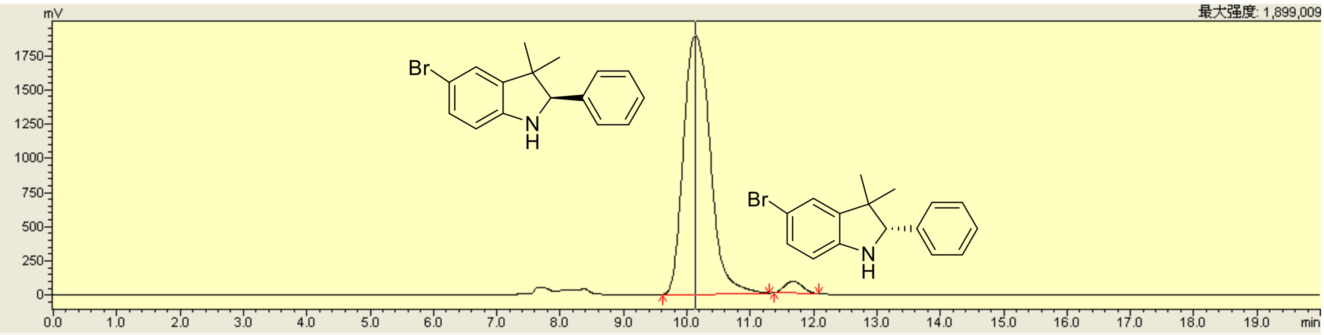

化合物表视图

ID#	名称	保留时间	峰#	面积	高度	面积%
1	RT10.131	10.131	1	55330977	1890223	97.0191
2	RT11.672	11.672	2	1700025	85998	2.9809

<u>Translation of all characters (Chinese) in the above two frameworks to English is as follows:</u>

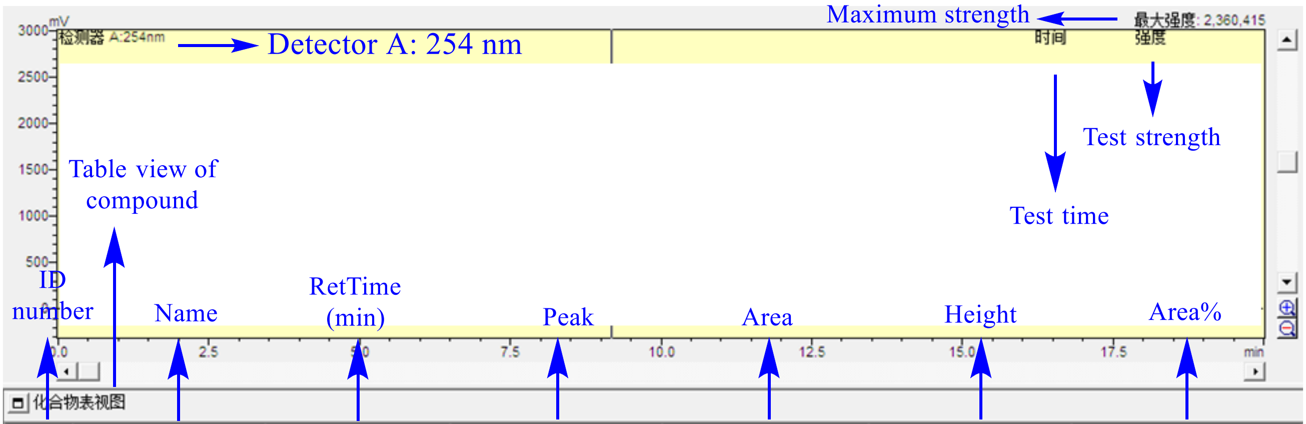

ID#	名称	保留时间	峰#	面积	高度	面积%
1	RT9.190	9.190	1	39625295	2360461	47.0509
2	RT11.859	11.859	2	44592689	2144002	52.9491

(*R*)-1f：(R)-6-bromo-3,3-dimethyl-2-phenylindoline：(HPLC: Chiracel IC-H, detected at 254 nm, eluent: n-hexane/2-propanol = 97/03, flow rate = 0.5mL/min, 25 ℃).

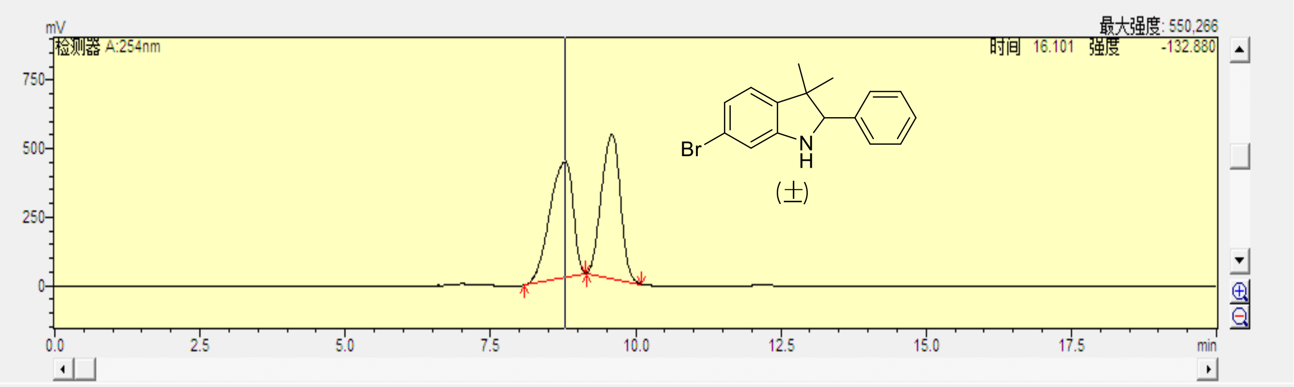

化合物表视图

ID#	名称	保留时间	峰#	面积	高度	面积%
1	RT8.784	8.784	1	11798880	422225	49.8946
2	RT9.590	9.590	2	11848737	522376	50.1054

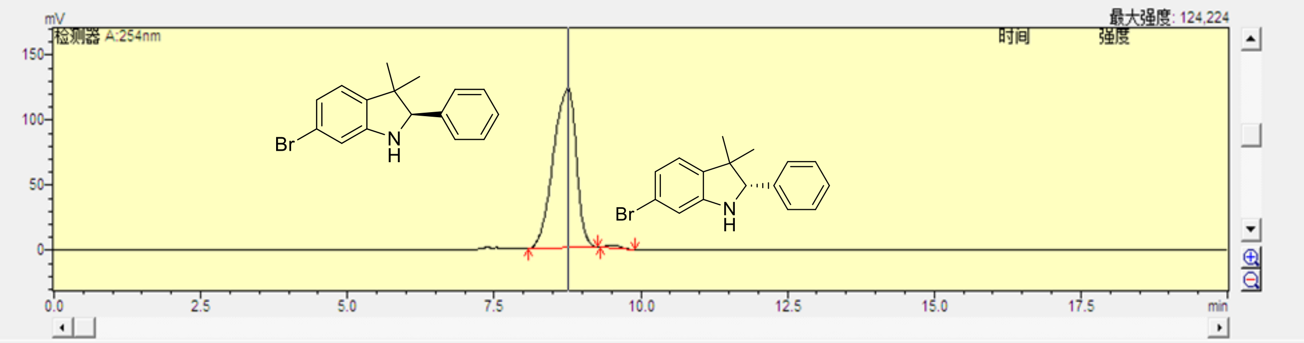

化合物表视图

ID#	名称	保留时间	峰#	面积	高度	面积%
1	RT8.757	8.757	1	3348219	122292	98.9519
2	RT9.520	9.520	2	35466	2061	1.0481

<u>**Translation of all characters (Chinese) in the above two frameworks to English is as follows:**</u>

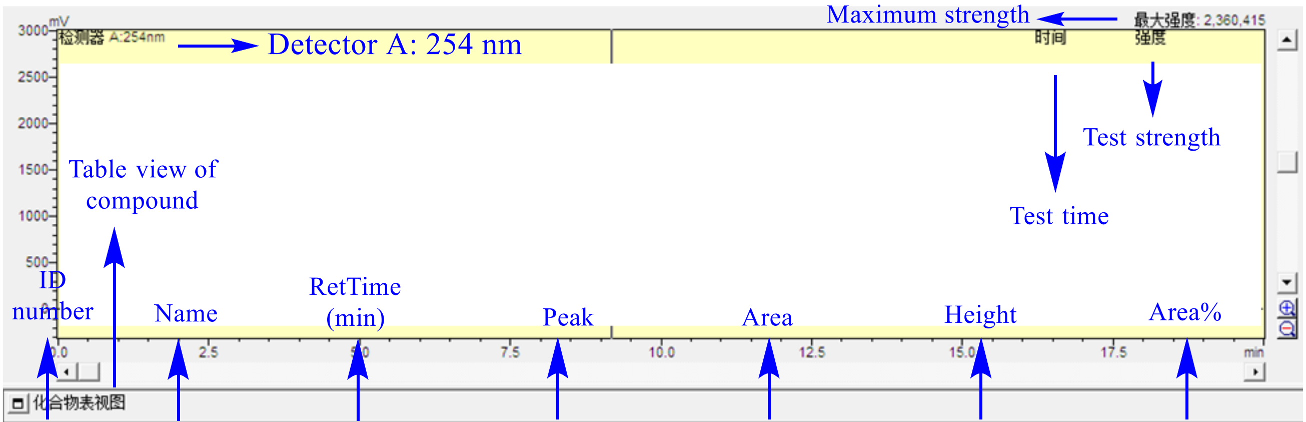

化合物表视图

ID#	名称	保留时间	峰#	面积	高度	面积%
1	RT9.190	9.190	1	39625295	2360461	47.0509
2	RT11.859	11.859	2	44592689	2144002	52.9491

(*R*)-1g: (*R*)-3,3-dimethyl-2-phenyl-5-(trifluoromethyl)indoline： (HPLC: Chiracel IC-H, detected at 254 nm, eluent: n-hexane/2-propanol = 97/03, flow rate = 0.5mL/min, 25 ℃).

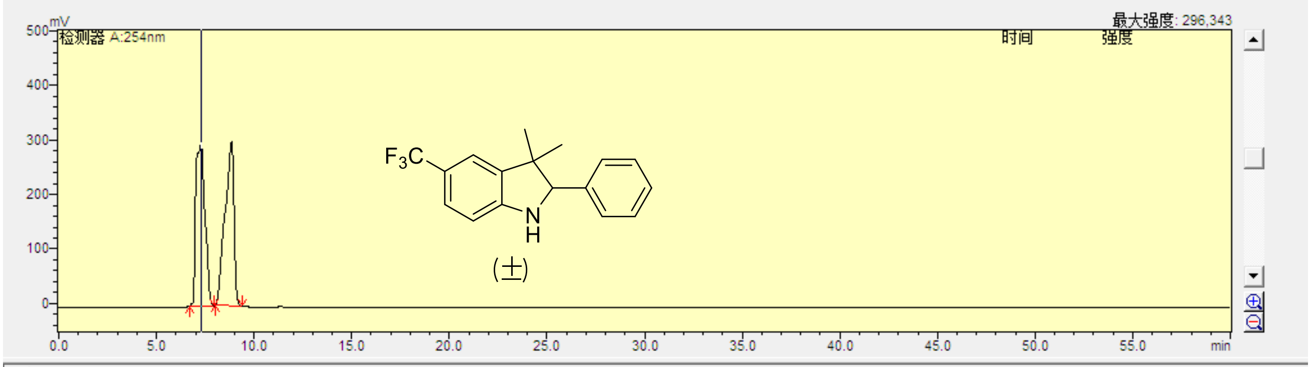

化合物表视图

ID#	名称	保留时间	峰#	面积	高度	面积%
1	RT7.285	7.285	1	9798099	298697	50.3604
2	RT8.841	8.841	2	9657861	301061	49.6396

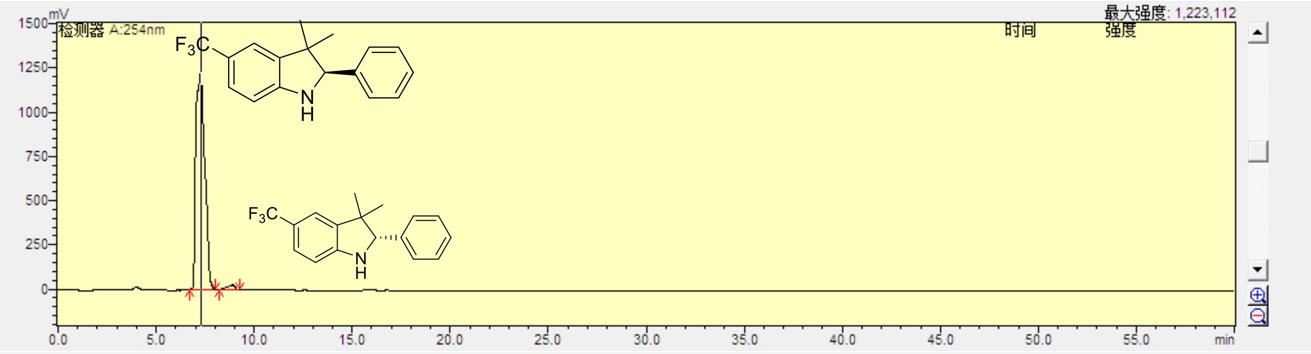

化合物表视图

ID#	名称	保留时间	峰#	面积	高度	面积%
1	RT7.274	7.274	1	39276416	1227343	98.0328
2	RT8.880	8.880	2	788130	27505	1.9672

<u>**Translation of all characters (Chinese) in the above two frameworks to English is as follows:**</u>

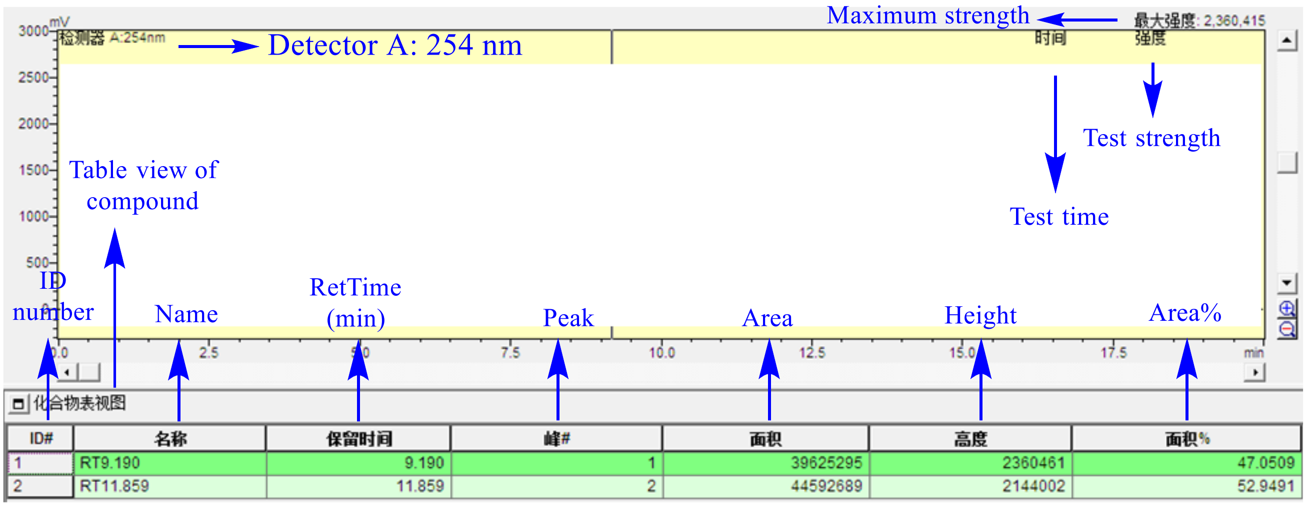

化合物表视图

ID#	名称	保留时间	峰#	面积	高度	面积%
1	RT9.190	9.190	1	39625295	2360461	47.0509
2	RT11.859	11.859	2	44592689	2144002	52.9491

(*R*)-1h：(R)-3,3,5-trimethyl-2-phenylindoline： (HPLC: Chiracel IC-H, detected at 254 nm, eluent: n-hexane/2-propanol = 97/03, flow rate = 0.5mL/min, 25 ℃).

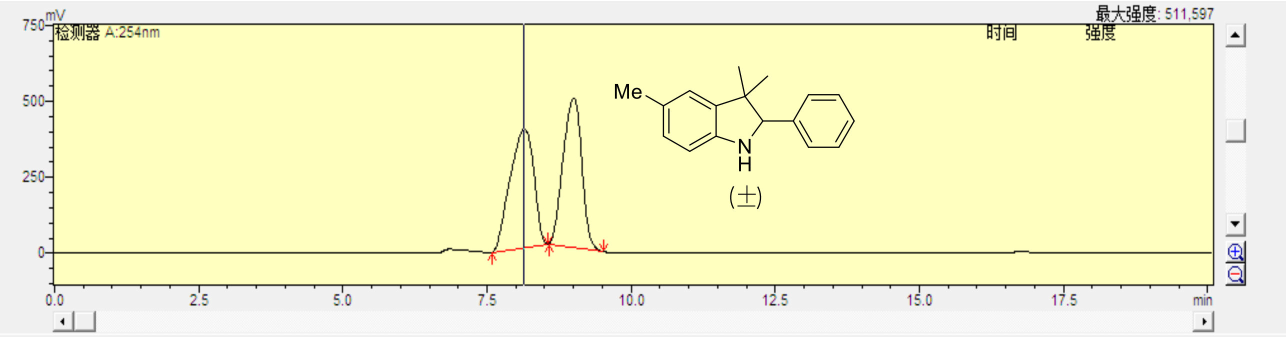

化合物表视图

ID#	名称	保留时间	峰#	面积	高度	面积%
1	RT8.142	8.142	1	11169657	390352	50.1560
2	RT9.004	9.004	2	11100162	492300	49.8440

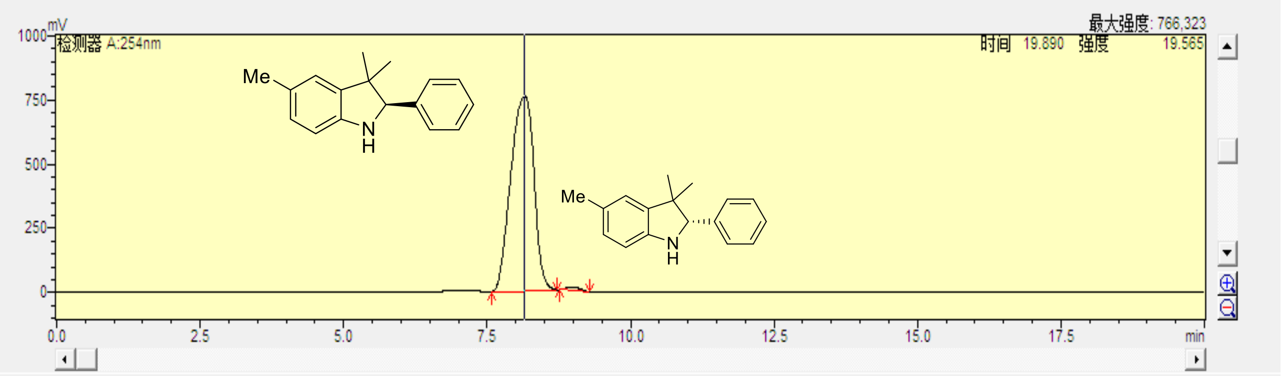

化合物表视图

ID#	名称	保留时间	峰#	面积	高度	面积%
1	RT8.151	8.151	1	21319263	759381	98.8550
2	RT8.990	8.990	2	246935	15113	1.1450

Translation of all characters (Chinese) in the above two frameworks to English is as follows:

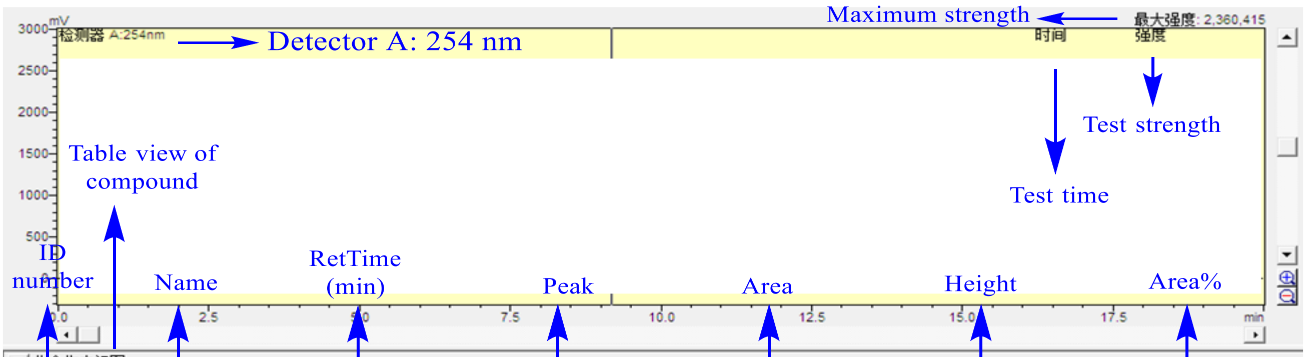

ID#	名称	保留时间	峰#	面积	高度	面积%
1	RT9.190	9.190	1	39625295	2360461	47.0509
2	RT11.859	11.859	2	44592689	2144002	52.9491

(*R*)-1i: (R)- 5-methoxy-3,3-dimethyl-2-phenylindoline: (HPLC: Chiracel OD-H, detected at 254 nm, eluent: n-hexane/2-propanol = 90/10, flow rate = 1.0mL/min, 25 ℃).

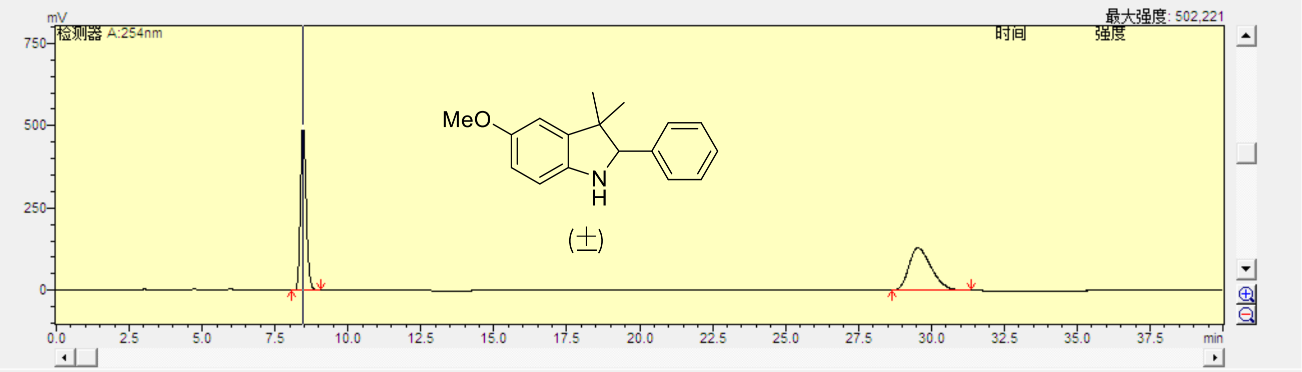

化合物表视图

ID#	名称	保留时间	峰#	面积	高度	面积%
1	RT8.453	8.453	1	6864757	502373	49.7339
2	RT29.541	29.541	2	6938218	128732	50.2661

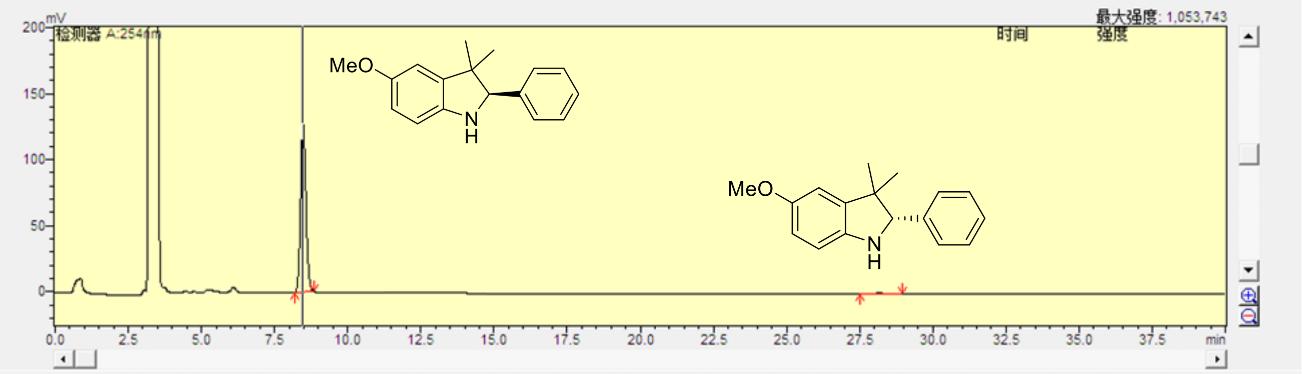

化合物表视图

ID#	名称	保留时间	峰#	面积	高度	面积%
1	RT8.470	8.470	1	1636322	127317	98.9250
2	RT28.156	28.156	2	17782	425	1.0750

<u>Translation of all characters (Chinese) in the above two frameworks to English is as follows:</u>

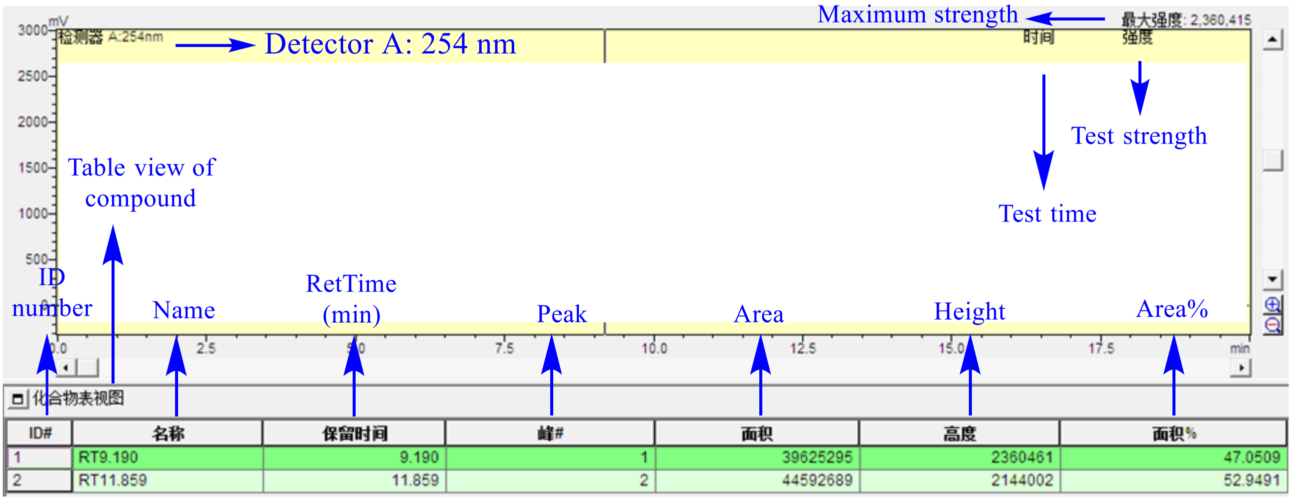

(*R*)-1j: (R)- 5-(benzyloxy)-3,3-dimethyl-2-phenylindoline：(HPLC: Chiracel IC-H, detected at 254 nm, eluent: n-hexane/2-propanol = 90/10, flow rate = 1mL/min, 25 ℃).

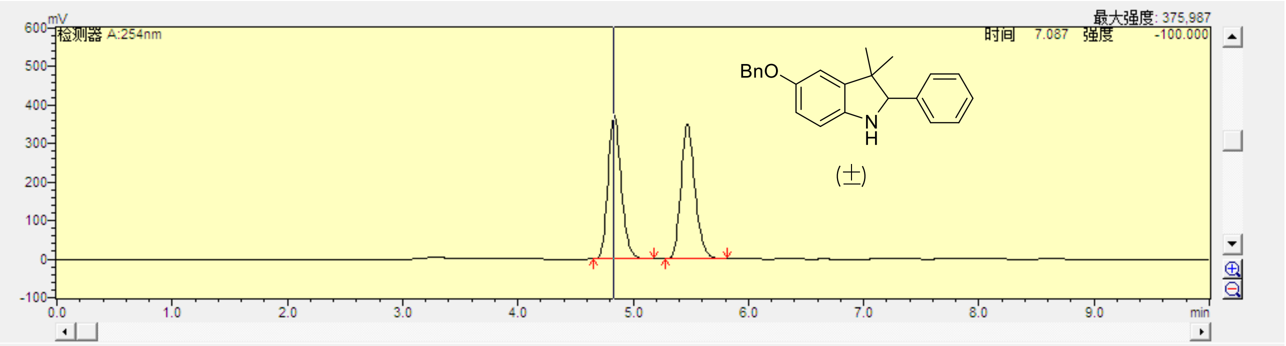

化合物表视图

ID#	名称	保留时间	峰#	面积	高度	面积%
1	RT4.831	4.831	1	2968949	375484	49.8775
2	RT5.466	5.466	2	2983533	349373	50.1225

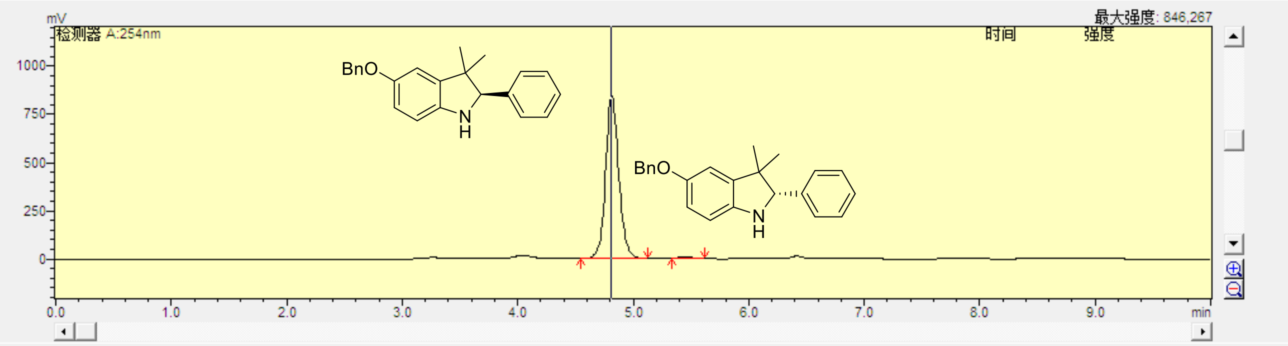

化合物表视图

ID#	名称	保留时间	峰#	面积	高度	面积%
1	RT4.810	4.810	1	6668500	844886	98.7211
2	RT5.440	5.440	2	86386	11354	1.2789

<u>Translation of all characters (Chinese) in the above two frameworks to English is as follows:</u>

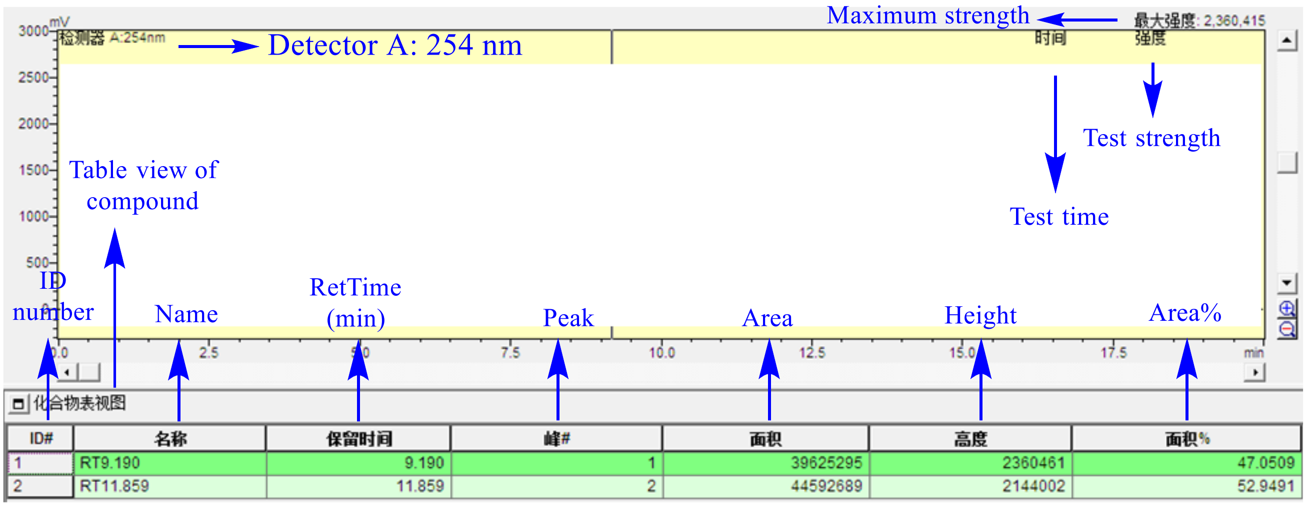

化合物表视图

ID#	名称	保留时间	峰#	面积	高度	面积%
1	RT9.190	9.190	1	39625295	2360461	47.0509
2	RT11.859	11.859	2	44592689	2144002	52.9491

(*R*)-1k: (R)- 3,3-dimethyl-2-phenyl-5-(trifluoromethoxy)indoline : (HPLC: Chiracel IC-H, detected at 254 nm, eluent: n-hexane/2-propanol = 97/03, flow rate = 0.5mL/min, 25 ℃).

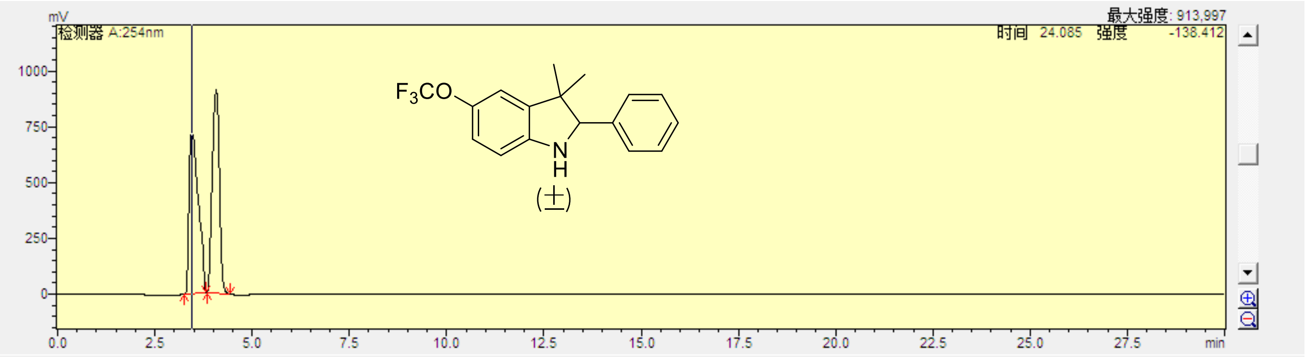

化合物表视图

ID#	名称	保留时间	峰#	面积	高度	面积%
1	RT3.446	3.446	1	11883671	714454	50.9607
2	RT4.068	4.068	2	11435626	910904	49.0393

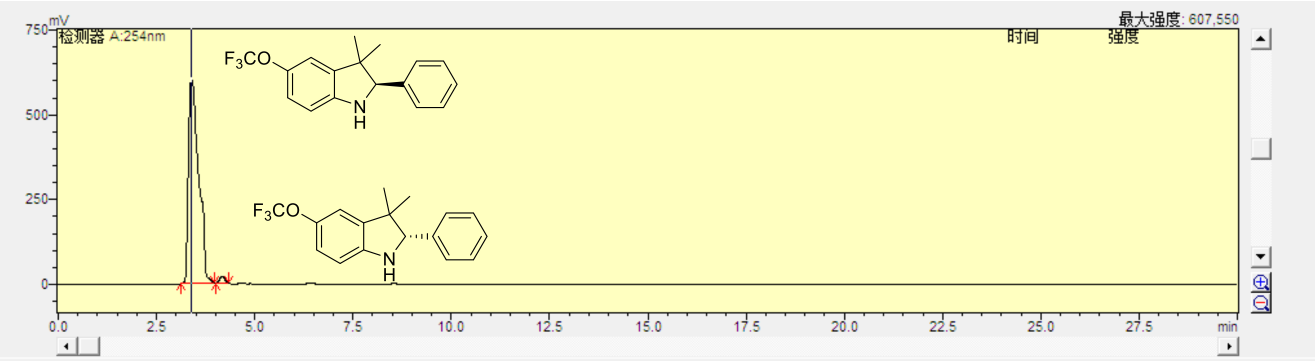

化合物表视图

ID#	名称	保留时间	峰#	面积	高度	面积%
1	RT3.384	3.384	1	10836474	604851	98.3595
2	RT4.170	4.170	2	180742	19872	1.6405

<u>Translation of all characters (Chinese) in the above two frameworks to English is as follows:</u>

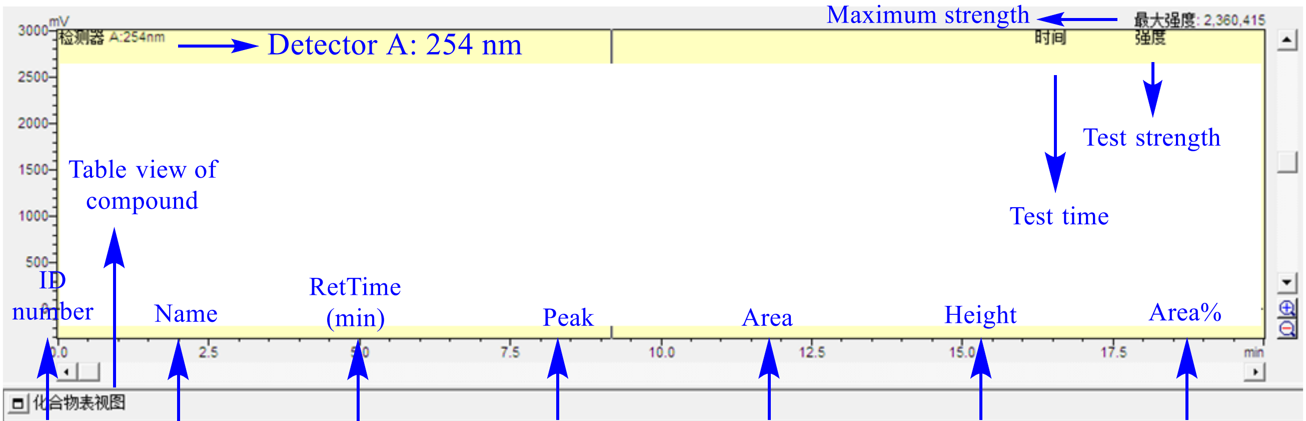

化合物表视图

ID#	名称	保留时间	峰#	面积	高度	面积%
1	RT9.190	9.190	1	39625295	2360461	47.0509
2	RT11.859	11.859	2	44592689	2144002	52.9491

(*R*)-1l: (R)-1,1-dimethyl-2-phenyl-2,3-dihydro-1H-benzo[e]indole.: (HPLC: Chiracel OD-H, detected at 254 nm, eluent: n-hexane/2-propanol = 90/10, flow rate = 1.0mL/min, 25 ℃).

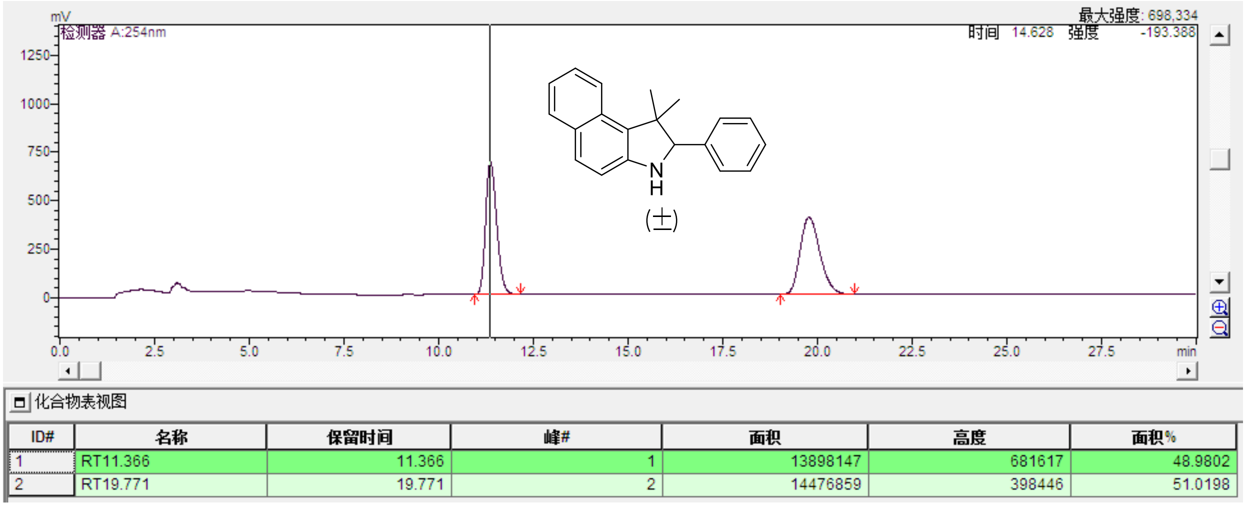

ID#	名称	保留时间	峰#	面积	高度	面积%
1	RT11.366	11.366	1	13898147	681617	48.9802
2	RT19.771	19.771	2	14476859	398446	51.0198

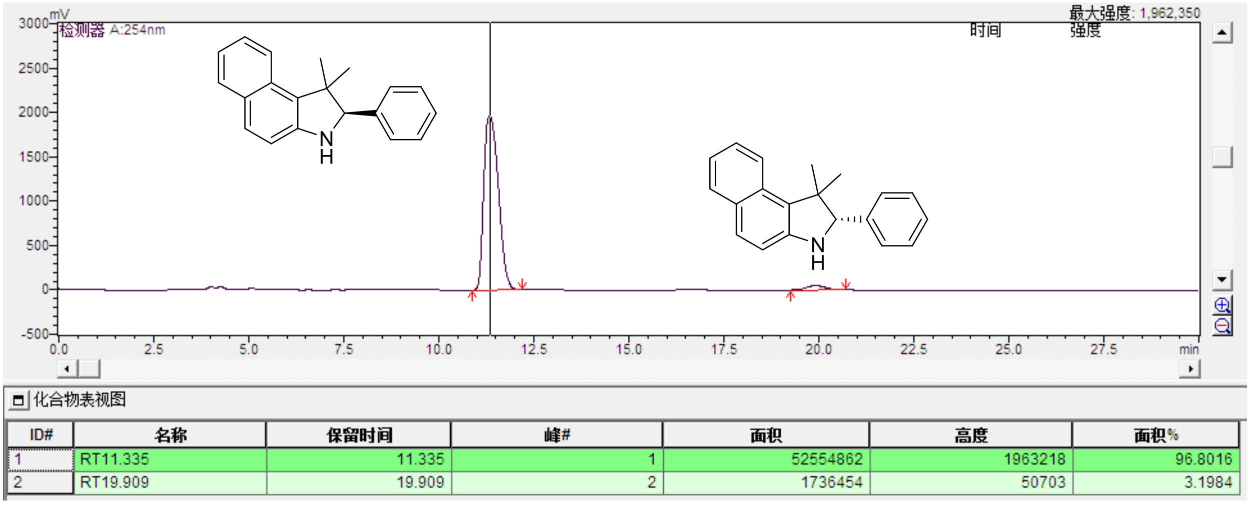

ID#	名称	保留时间	峰#	面积	高度	面积%
1	RT11.335	11.335	1	52554862	1963218	96.8016
2	RT19.909	19.909	2	1736454	50703	3.1984

<u>**Translation of all characters (Chinese) in the above two frameworks to English is as follows:**</u>

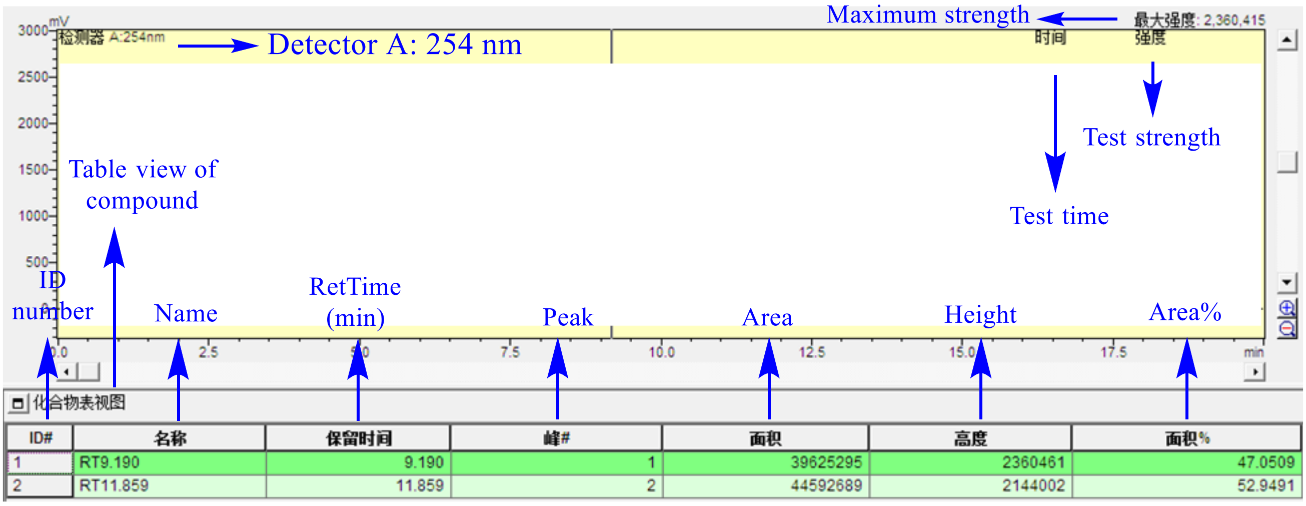

ID#	名称	保留时间	峰#	面积	高度	面积%
1	RT9.190	9.190	1	39625295	2360461	47.0509
2	RT11.859	11.859	2	44592689	2144002	52.9491

(*R*)-1m: **(R)-2-(4-chlorophenyl)-3,3-dimethylindoline**: (HPLC: Chiracel IC-H, detected at 254 nm, eluent: n-hexane/2-propanol = 97/03, flow rate = 0.5mL/min, 25 ℃).

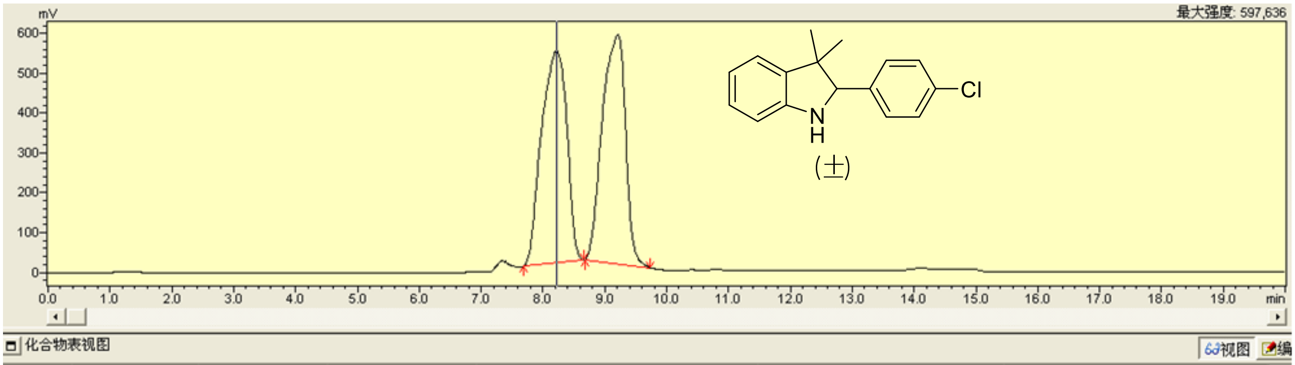

ID#	名称	保留时间	峰#	面积	高度	面积%
1	RT8.217	8.217	1	15173278	532917	50.3787
2	RT9.209	9.209	2	14945189	575449	49.6213

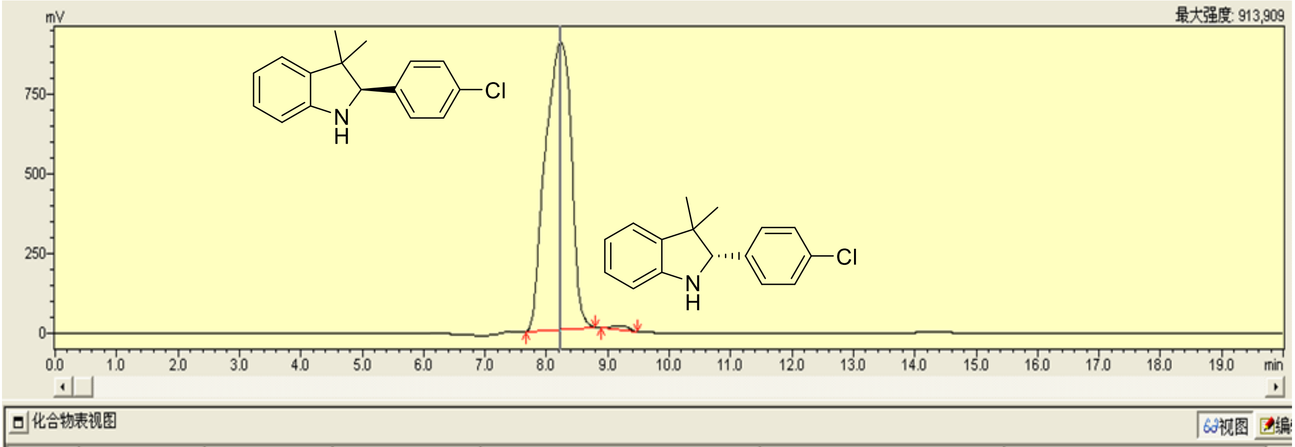

ID#	名称	保留时间	峰#	面积	高度	面积%
1	RT8.229	8.229	1	26616117	901895	99.0553
2	RT9.227	9.227	2	253834	13919	0.9447

Translation of all characters (Chinese) in the above two frameworks to English is as follows:

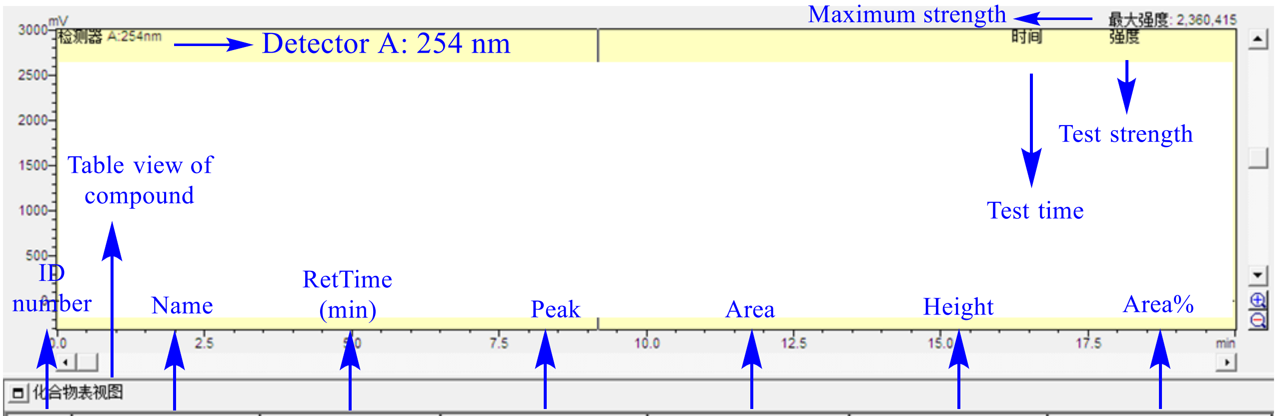

ID#	名称	保留时间	峰#	面积	高度	面积%
1	RT9.190	9.190	1	39625295	2360461	47.0509
2	RT11.859	11.859	2	44592689	2144002	52.9491

(*R*)-1n：(R)-2-(4-bromophenyl)-3,3-dimethylindoline.：(HPLC: Chiracel IC-H, detected at 254 nm, eluent: n-hexane/2-propanol = 97/03, flow rate = 0.5mL/min, 25 ℃).

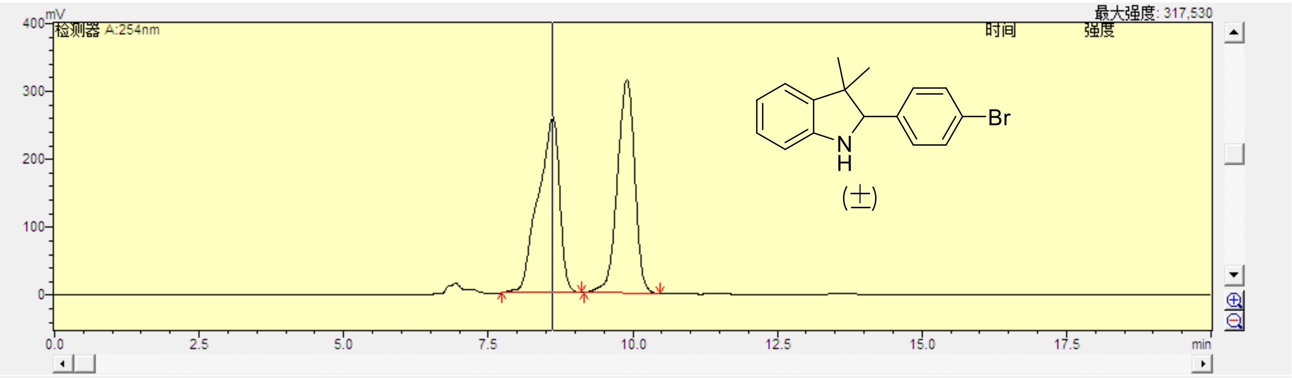

化合物表视图

ID#	名称	保留时间	峰#	面积	高度	面积%
1	RT8.615	8.615	1	6658710	258817	50.2694
2	RT9.896	9.896	2	6587327	314502	49.7306

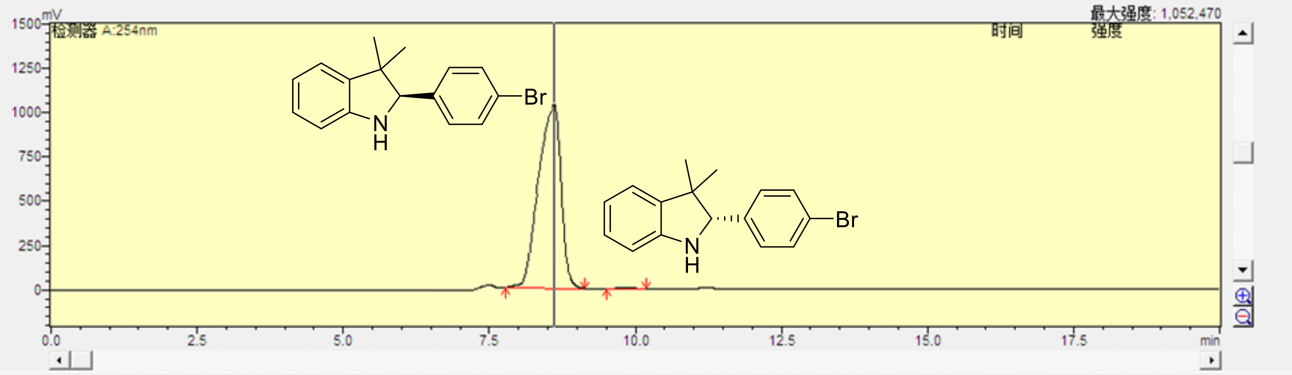

化合物表视图

ID#	名称	保留时间	峰#	面积	高度	面积%
1	RT8.602	8.602	1	29071067	1042101	99.4583
2	RT9.862	9.862	2	158330	8446	0.5417

<u>Translation of all characters (Chinese) in the above two frameworks to English is as follows:</u>

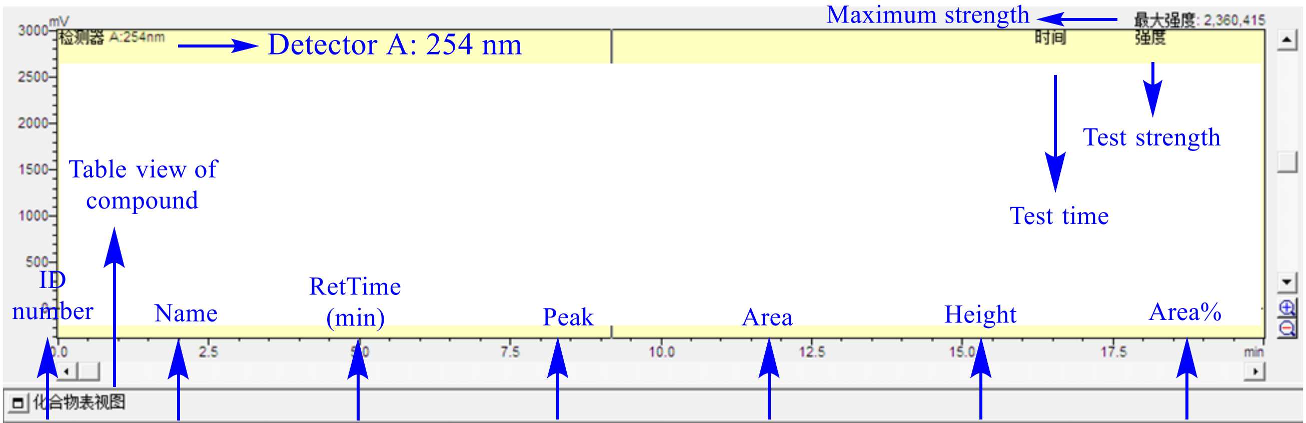

ID#	名称	保留时间	峰#	面积	高度	面积%
1	RT9.190	9.190	1	39625295	2360461	47.0509
2	RT11.859	11.859	2	44592689	2144002	52.9491

(*R*)-1o: (R)-2-(4-ethylphenyl)-3,3-dimethylindoline.: (HPLC: Chiracel OD-H, detected at 254 nm, eluent: n-hexane/2-propanol = 90/10, flow rate = 1.0mL/min, 25 ℃).

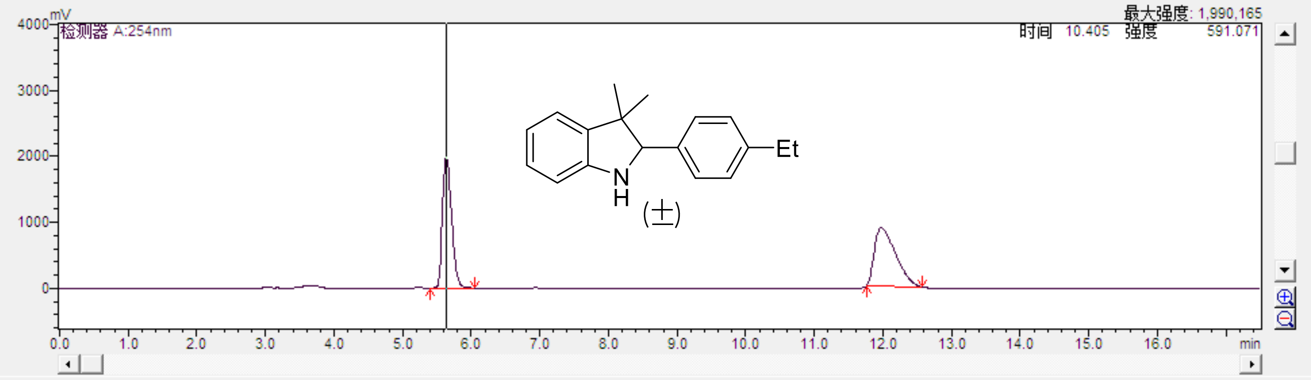

化合物表视图

ID#	名称	保留时间	峰#	面积	高度	面积%
1	RT5.631	5.631	1	18452352	1987028	49.0783
2	RT11.973	11.973	2	19145449	889423	50.9217

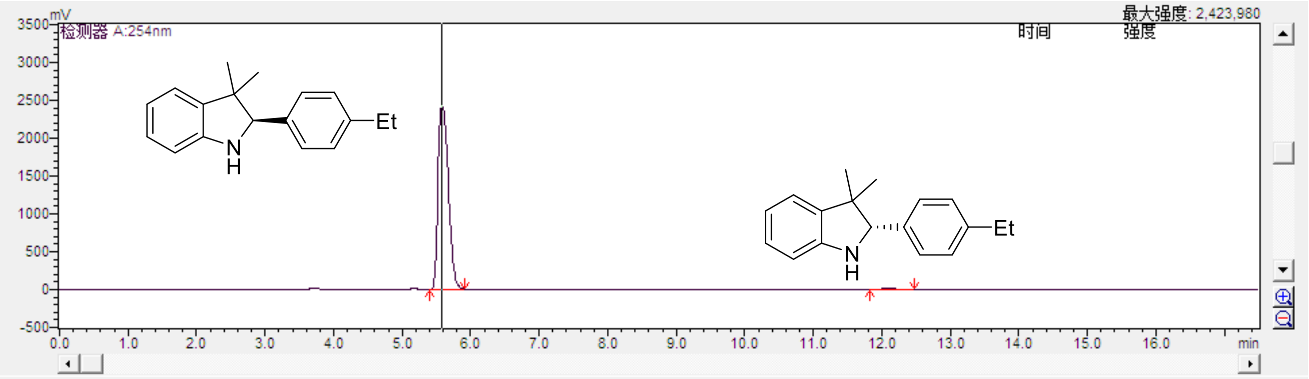

化合物表视图

ID#	名称	保留时间	峰#	面积	高度	面积%
1	RT5.580	5.580	1	25635427	2418893	99.0411
2	RT12.101	12.101	2	248208	14065	0.9589

Translation of all characters (Chinese) in the above two frameworks to English is as follows:

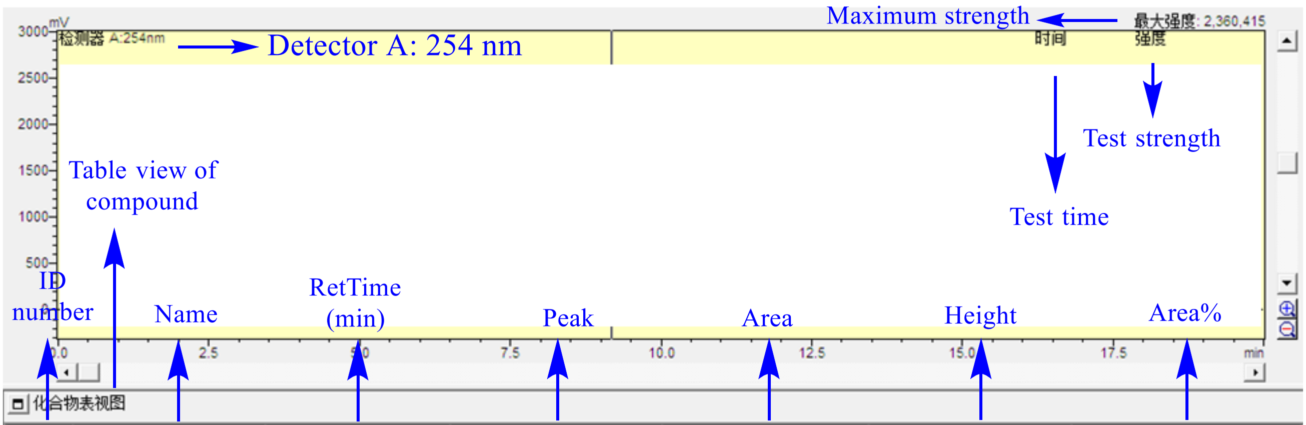

ID#	名称	保留时间	峰#	面积	高度	面积%
1	RT9.190	9.190	1	39625295	2360461	47.0509
2	RT11.859	11.859	2	44592689	2144002	52.9491

(*R*)-1p: (R)-2-(4-methoxyphenyl)-3,3-dimethylindoline.: (HPLC: Chiracel OD-H, detected at 254 nm, eluent: n-hexane/2-propanol = 90/10, flow rate = 1.0mL/min, 25 ℃).

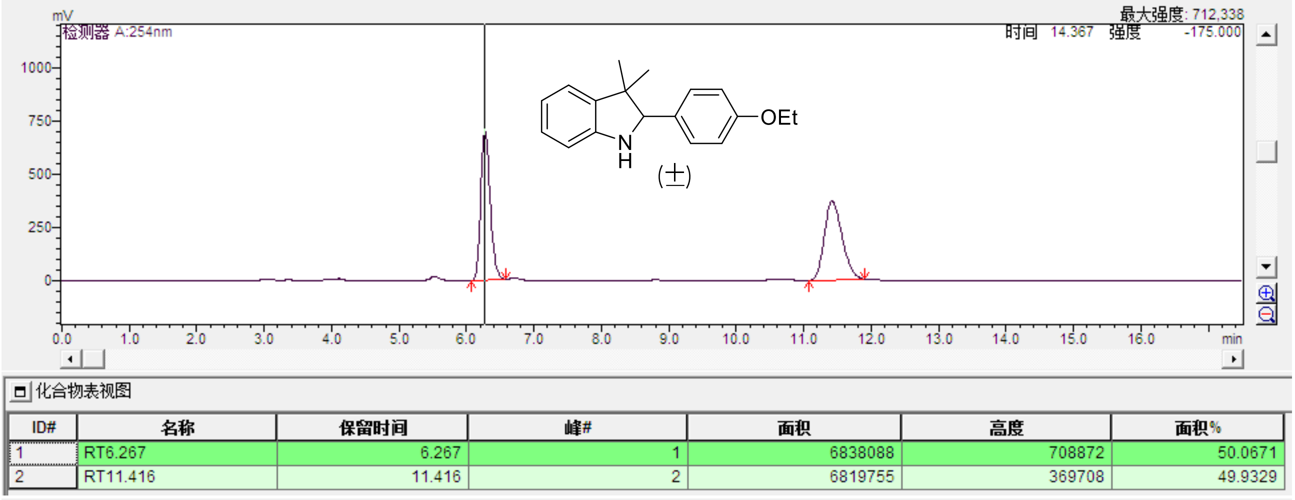

ID#	名称	保留时间	峰#	面积	高度	面积%
1	RT6.267	6.267	1	6838088	708872	50.0671
2	RT11.416	11.416	2	6819755	369708	49.9329

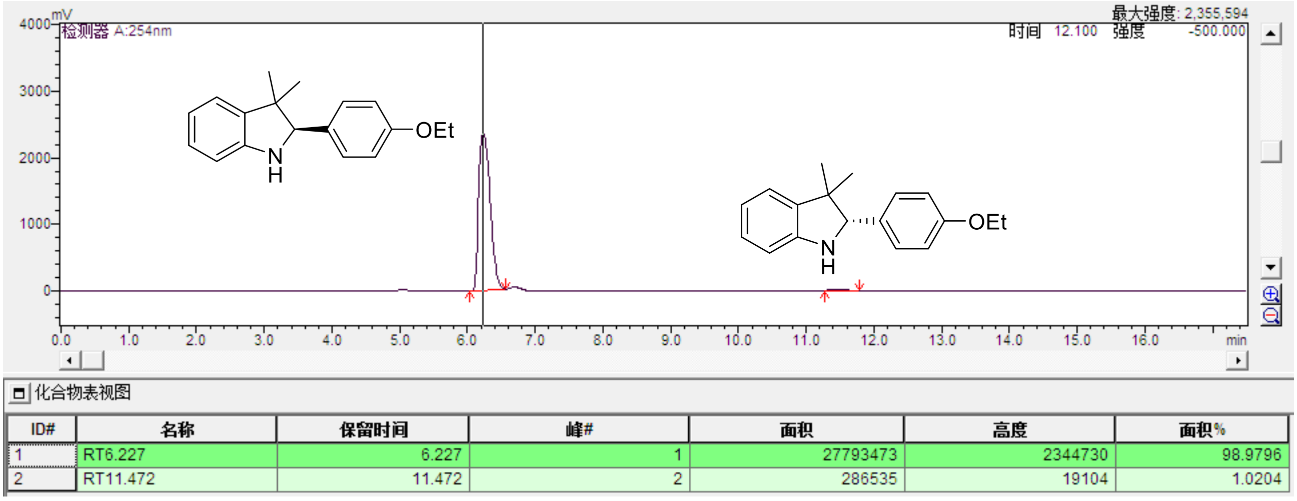

ID#	名称	保留时间	峰#	面积	高度	面积%
1	RT6.227	6.227	1	27793473	2344730	98.9796
2	RT11.472	11.472	2	286535	19104	1.0204

Translation of all characters (Chinese) in the above two frameworks to English is as follows:

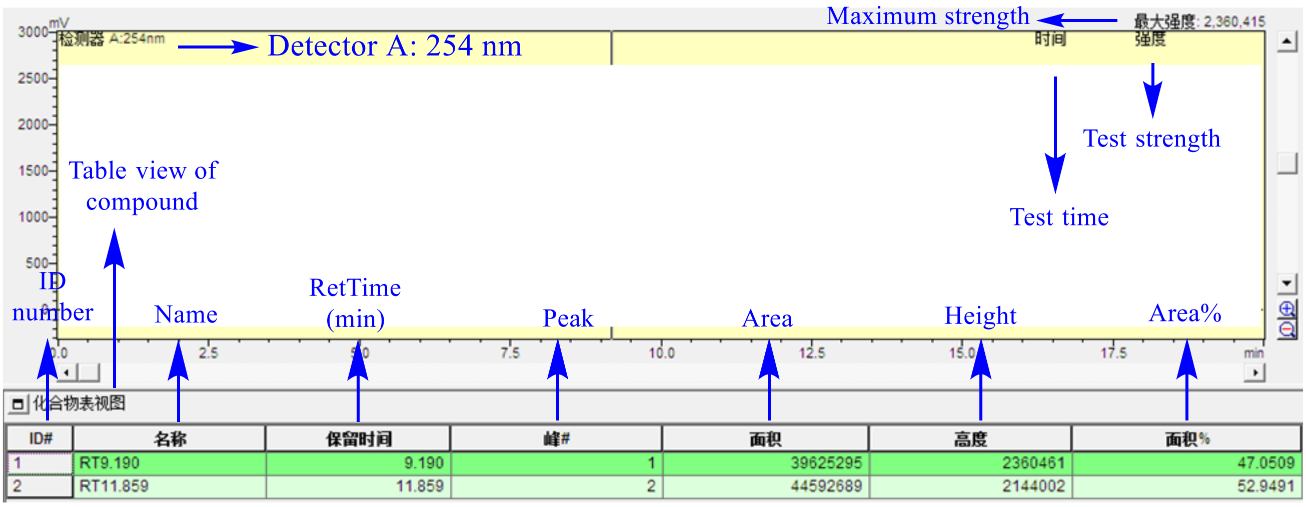

ID#	名称	保留时间	峰#	面积	高度	面积%
1	RT9.190	9.190	1	39625295	2360461	47.0509
2	RT11.859	11.859	2	44592689	2144002	52.9491

(*R*)-1q: (R)-2'-phenylspiro[cyclopentane-1,3'-indoline]: (HPLC: Chiracel IC-H, detected at 254 nm, eluent: n-hexane/2-propanol = 97/03, flow rate = 0.5mL/min, 25 ℃).

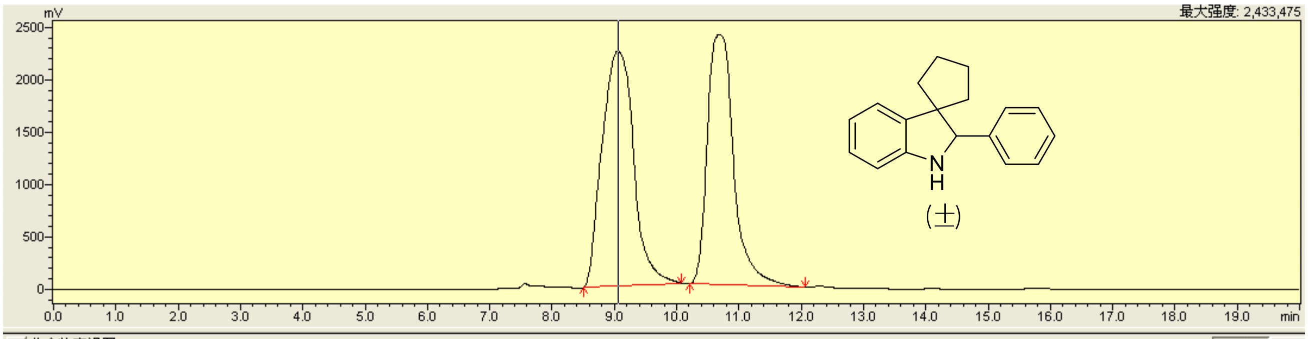

ID#	名称	保留时间	峰#	面积	高度	面积%
1	RT9. 054	9. 054	1	78946065	2241454	52. 8666
2	RT10. 679	10. 679	2	70384745	2390560	47. 1334

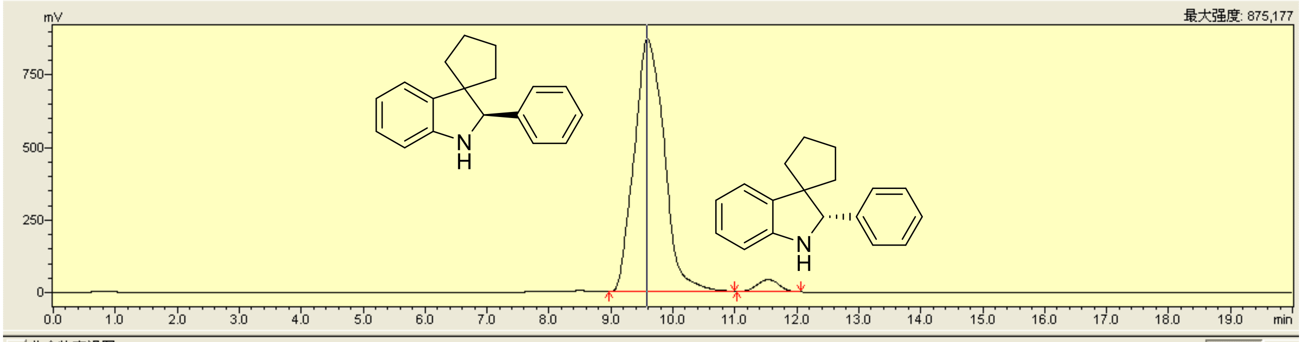

ID#	名称	保留时间	峰#	面积	高度	面积%
1	RT9. 587	9. 587	1	29329244	872071	96. 6515
2	RT11. 542	11. 542	2	1016107	40882	3. 3485

<u>Translation of all characters (Chinese) in the above two frameworks to English is as follows:</u>

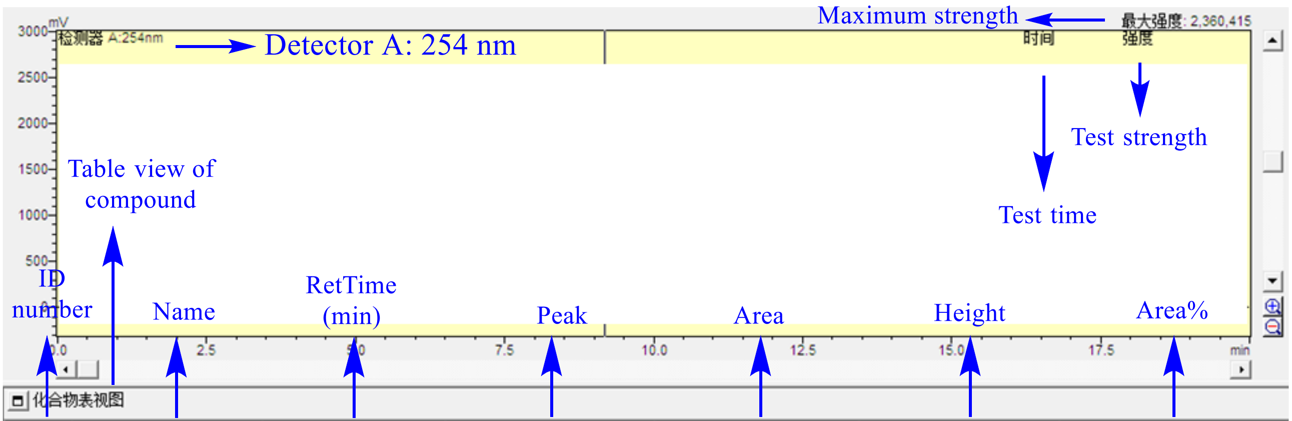

ID#	名称	保留时间	峰#	面积	高度	面积%
1	RT9.190	9.190	1	39625295	2360461	47.0509
2	RT11.859	11.859	2	44592689	2144002	52.9491

(***R***)**-1r: (R)-5'-fluoro-2'-phenylspiro[cyclopentane-1,3'-indoline].:** (HPLC: Chiracel IC-H, detected at 254 nm, eluent: n-hexane/2-propanol = 97/03, flow rate = 0.5mL/min, 25 ℃).

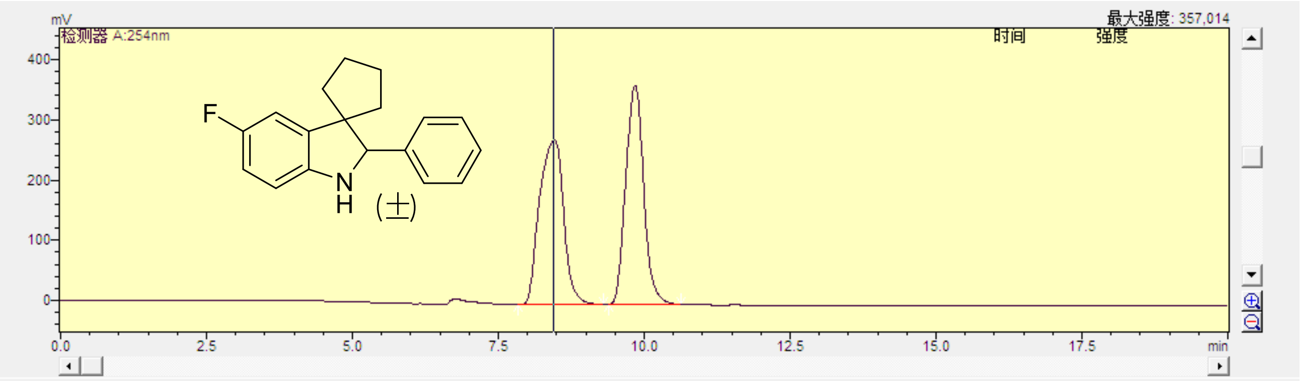

化合物表视图

ID#	名称	保留时间	峰#	面积	高度	面积%
1	RT8.454	8.454	1	7863056	273474	50.1922
2	RT9.843	9.843	2	7802848	363629	49.8078

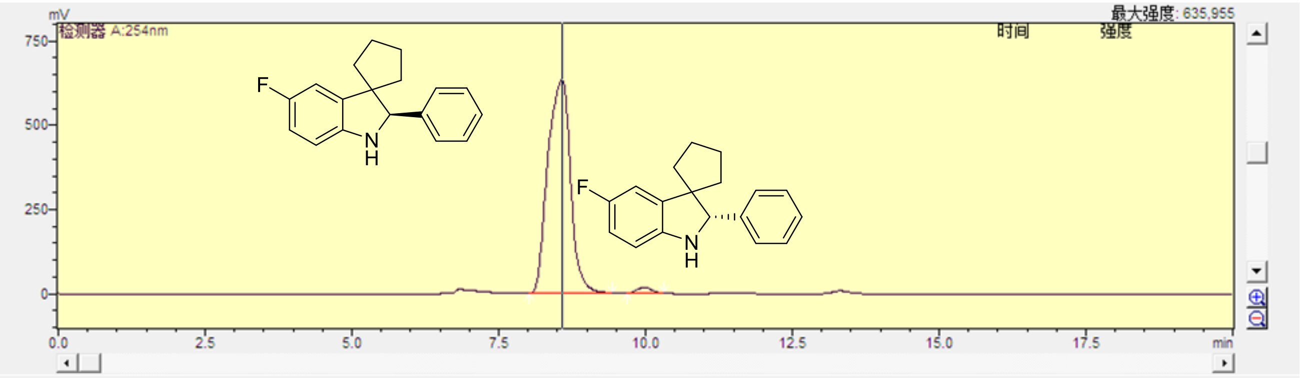

化合物表视图

ID#	名称	保留时间	峰#	面积	高度	面积%
1	RT8.573	8.573	1	17002995	634590	98.2230
2	RT9.980	9.980	2	307602	17199	1.7770

Translation of all characters (Chinese) in the above two frameworks to English is as follows:

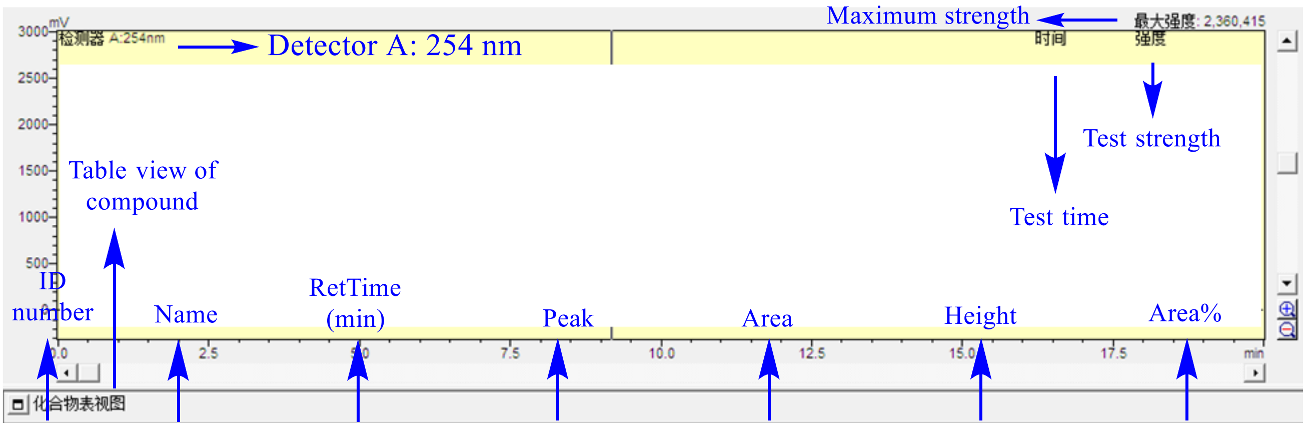

ID#	名称	保留时间	峰#	面积	高度	面积%
1	RT9.190	9.190	1	39625295	2360461	47.0509
2	RT11.859	11.859	2	44592689	2144002	52.9491

(*R*)-1s：(R)-5'-bromo-2'-phenylspiro[cyclopentane-1,3'-indoline]： (HPLC: Chiracel IC-H, detected at 254 nm, eluent: n-hexane/2-propanol = 97/03, flow rate = 0.5mL/min, 25 ℃).

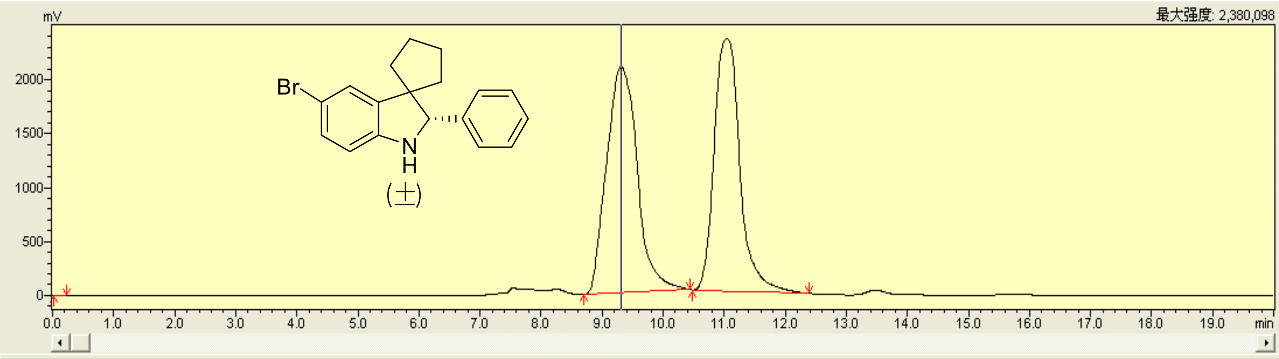

化合物表视图

ID#	名称	保留时间	峰#	面积	高度	面积%
1	RT9.309	9.309	2	73329697	2094631	51.6957
2	RT11.037	11.037	3	68517902	2337460	48.3035

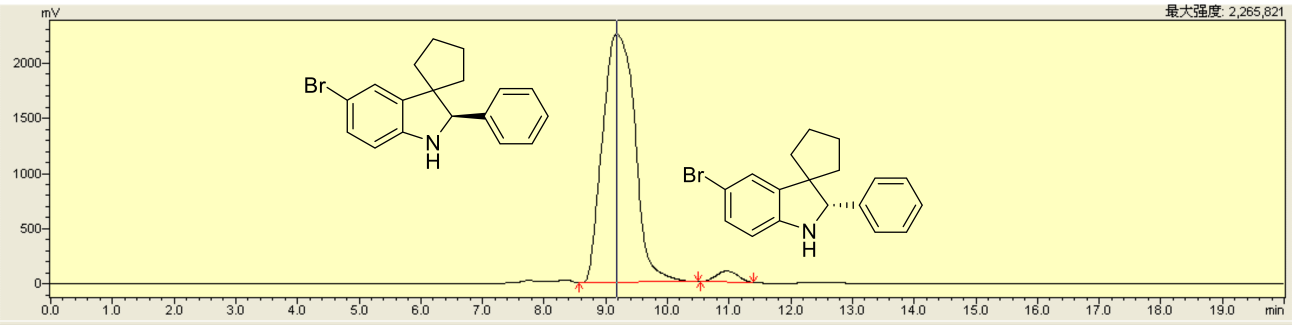

化合物表视图

ID#	名称	保留时间	峰#	面积	高度	面积%
1	RT9.176	9.176	1	82567733	2254128	97.1283
2	RT10.956	10.956	2	2441214	99257	2.8717

<u>Translation of all characters (Chinese) in the above two frameworks to English is as follows:</u>

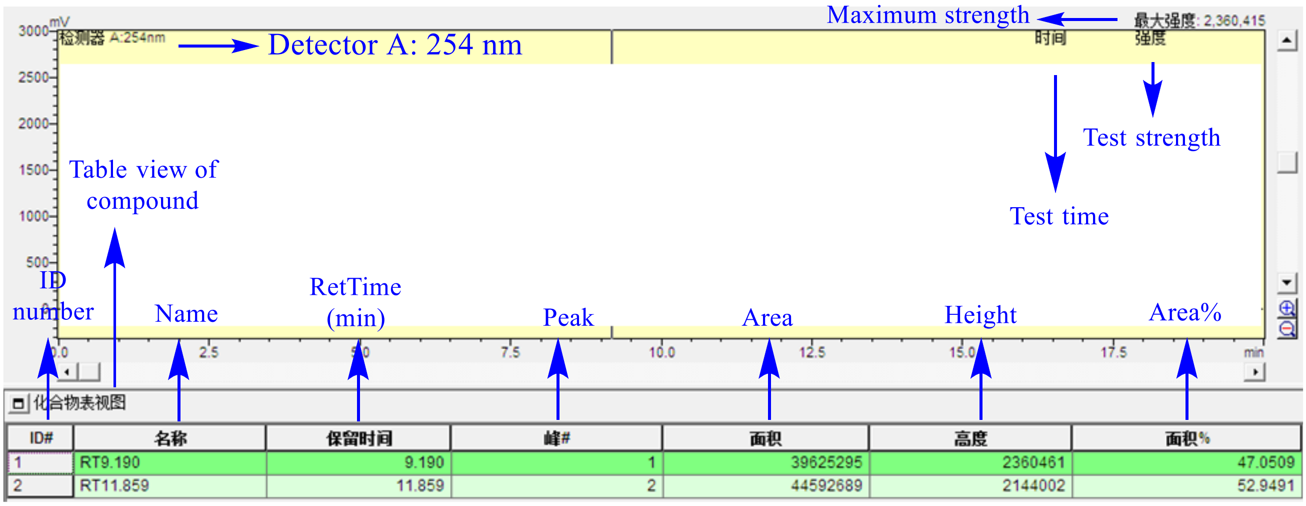

ID#	名称	保留时间	峰#	面积	高度	面积%
1	RT9.190	9.190	1	39625295	2360461	47.0509
2	RT11.859	11.859	2	44592689	2144002	52.9491

(*R*)-1t: (R)-2'-phenylspiro[cyclohexane-1,3'-indoline]： (HPLC: Chiracel IC-H, detected at 254 nm, eluent: n-hexane/2-propanol = 97/03, flow rate = 0.5mL/min, 25 ℃).

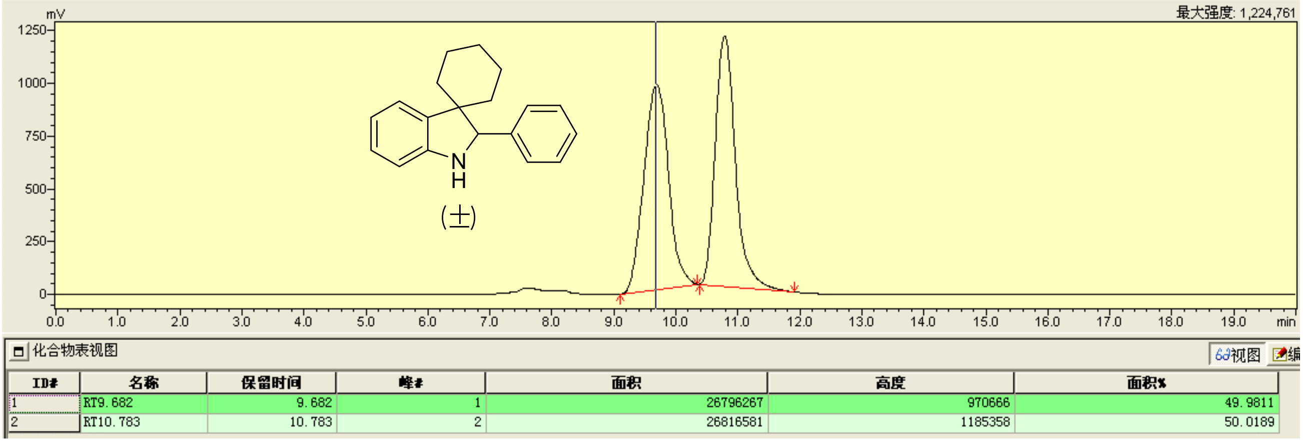

ID#	名称	保留时间	峰#	面积	高度	面积%
1	RT9.682	9.682	1	26796267	970666	49.9811
2	RT10.783	10.783	2	26816581	1185358	50.0189

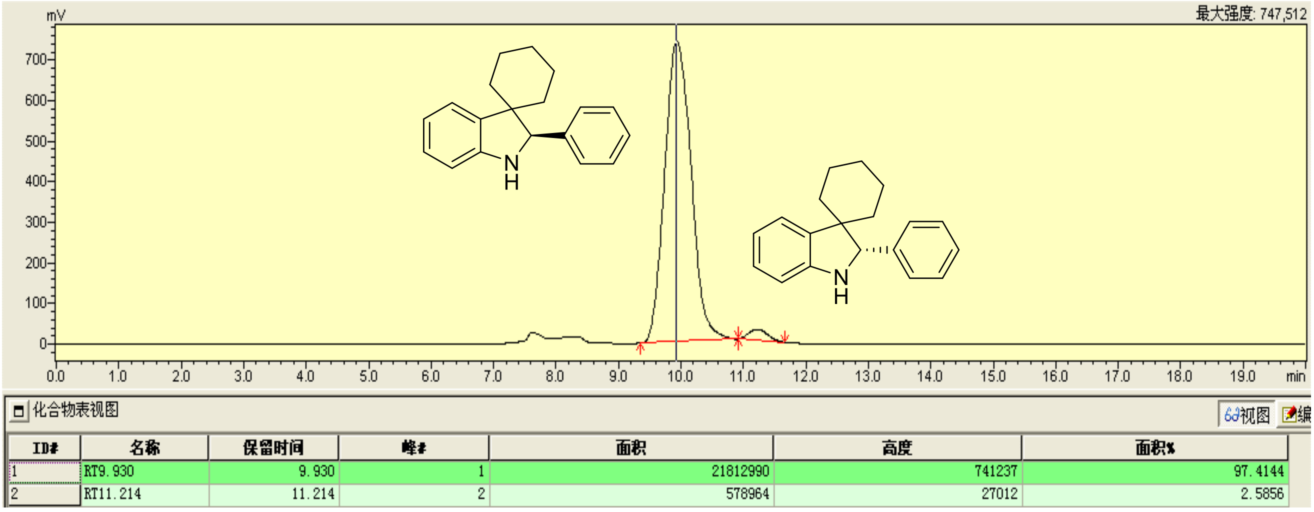

ID#	名称	保留时间	峰#	面积	高度	面积%
1	RT9.930	9.930	1	21812990	741237	97.4144
2	RT11.214	11.214	2	578964	27012	2.5856

Translation of all characters (Chinese) in the above two frameworks to English is as follows:

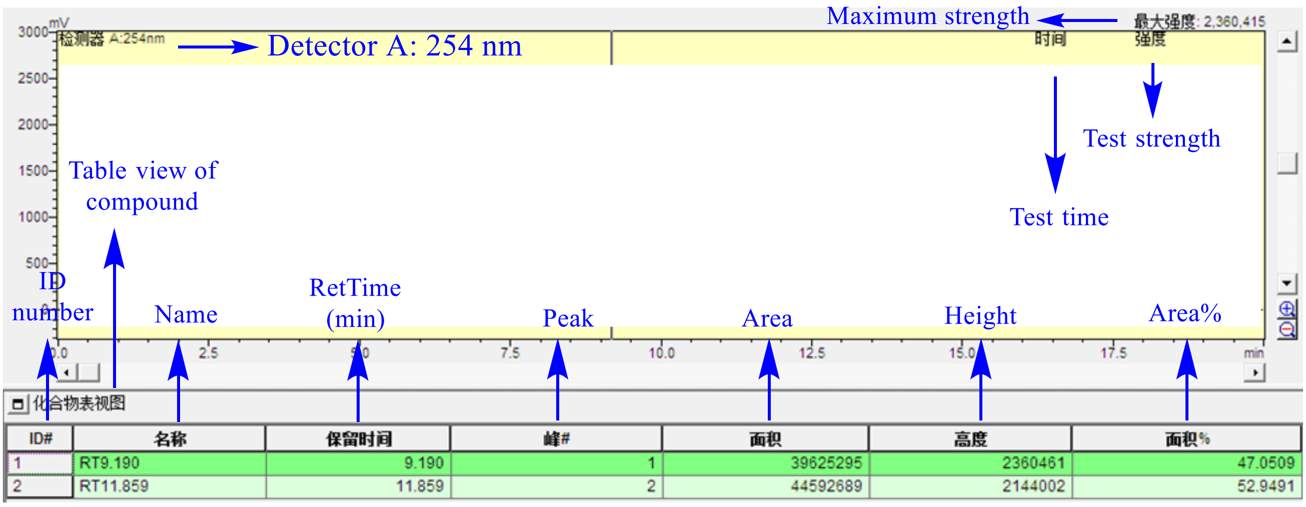

ID#	名称	保留时间	峰#	面积	高度	面积%
1	RT9.190	9.190	1	39625295	2360461	47.0509
2	RT11.859	11.859	2	44592689	2144002	52.9491

(*R*)-1u: (R)-5'-fluoro-2'-phenylspiro[cyclohexane-1,3'-indoline]: (HPLC: Chiracel IC-H, detected at 254 nm, eluent: n-hexane/2-propanol = 97/03, flow rate = 0.5mL/min, 25 ℃).

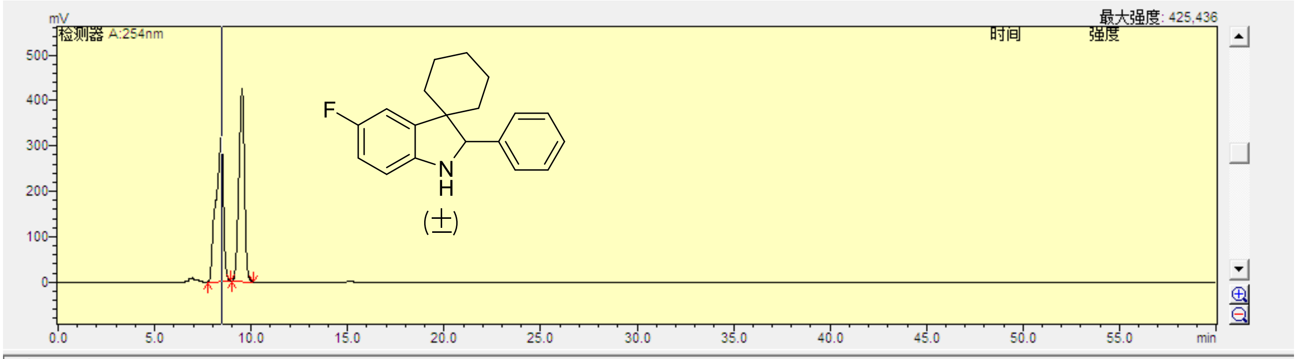

化合物表视图

ID#	名称	保留时间	峰#	面积	高度	面积%
1	RT8.451	8.451	1	8677755	317631	50.2074
2	RT9.527	9.527	2	8606045	424114	49.7926

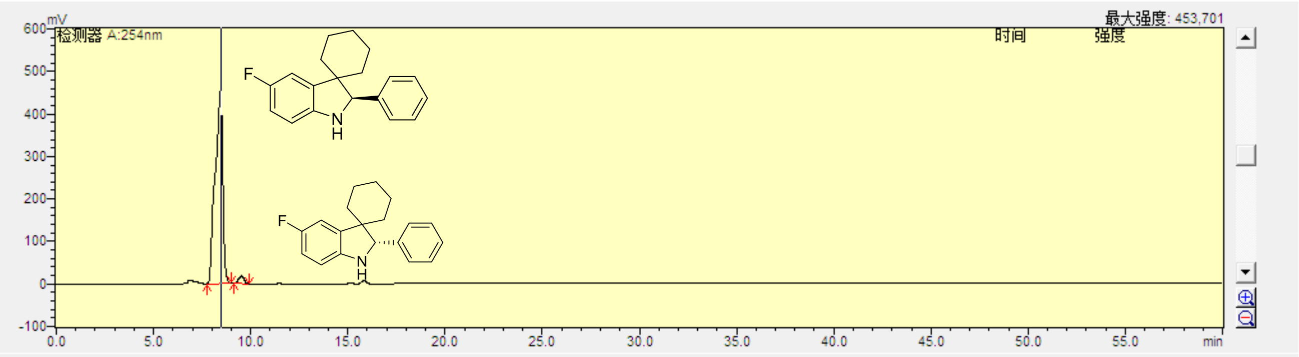

化合物表视图

ID#	名称	保留时间	峰#	面积	高度	面积%
1	RT8.445	8.445	1	12365353	452623	97.2848
2	RT9.532	9.532	2	345111	17990	2.7152

<u>**Translation of all characters (Chinese) in the above two frameworks to English is as follows:**</u>

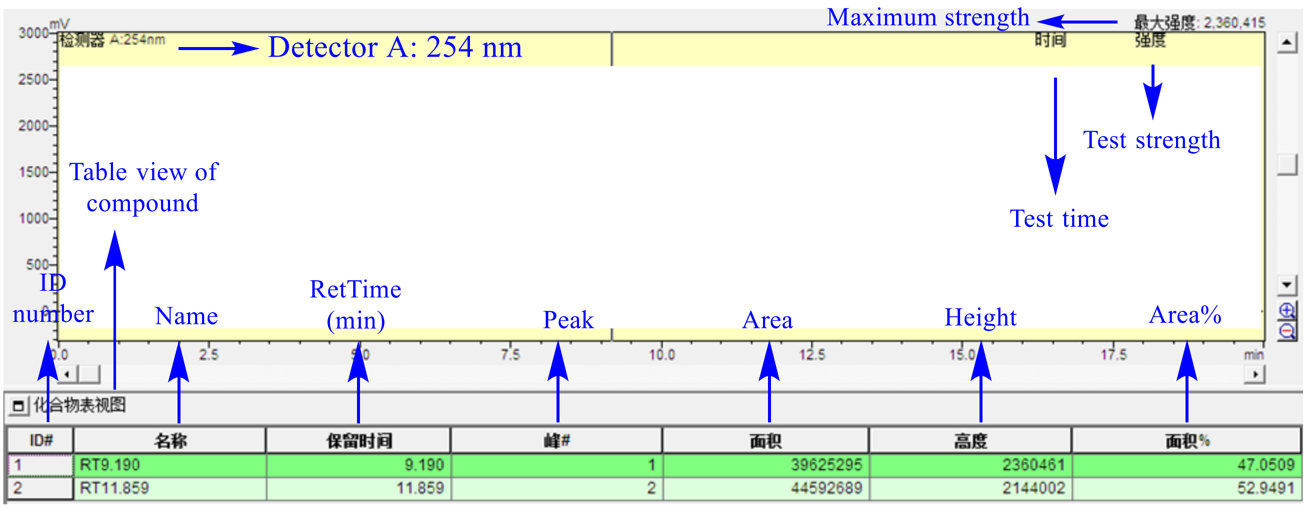

化合物表视图

ID#	名称	保留时间	峰#	面积	高度	面积%
1	RT9.190	9.190	1	39625295	2360461	47.0509
2	RT11.859	11.859	2	44592689	2144002	52.9491

(***R***)**-1v：(R)-5'-chloro-2'-phenylspiro[cyclohexane-1,3'-indoline]：**(HPLC: Chiracel IC-H, detected at 254 nm, eluent: n-hexane/2-propanol = 97/03, flow rate = 0.5mL/min, 25 ℃).

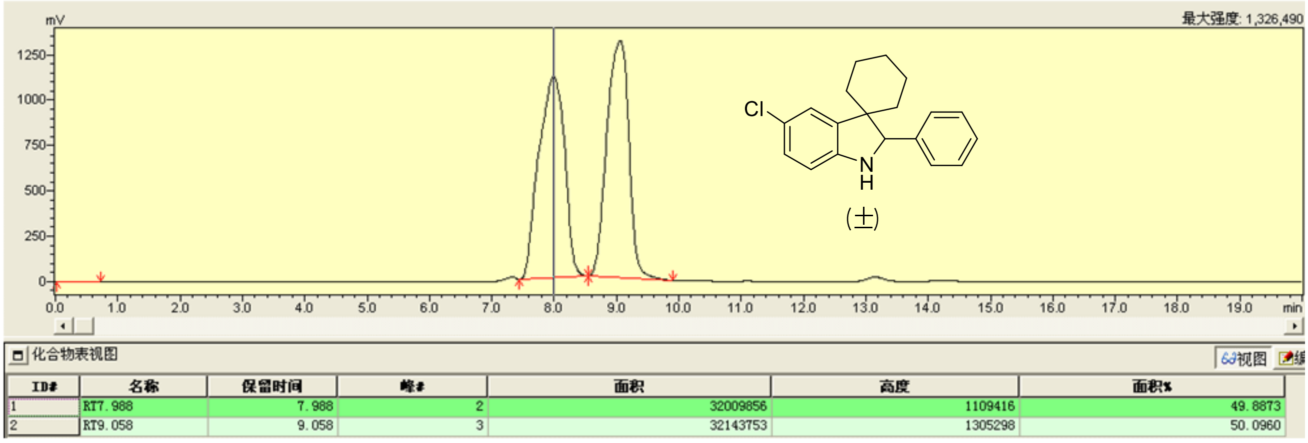

ID#	名称	保留时间	峰#	面积	高度	面积%
1	RT7.988	7.988	2	32009856	1109416	49.8873
2	RT9.058	9.058	3	32143753	1305298	50.0960

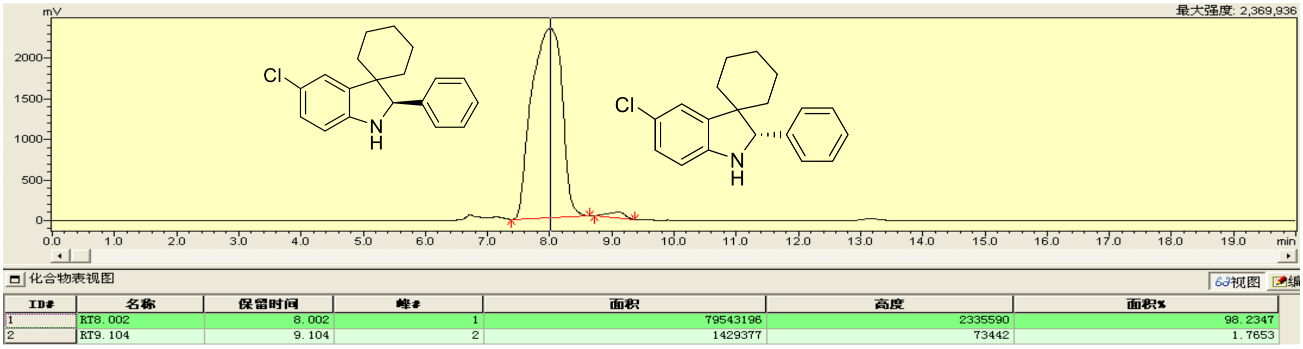

ID#	名称	保留时间	峰#	面积	高度	面积%
1	RT8.002	8.002	1	79543196	2335590	98.2347
2	RT9.104	9.104	2	1429377	73442	1.7653

Translation of all characters (Chinese) in the above two frameworks to English is as follows:

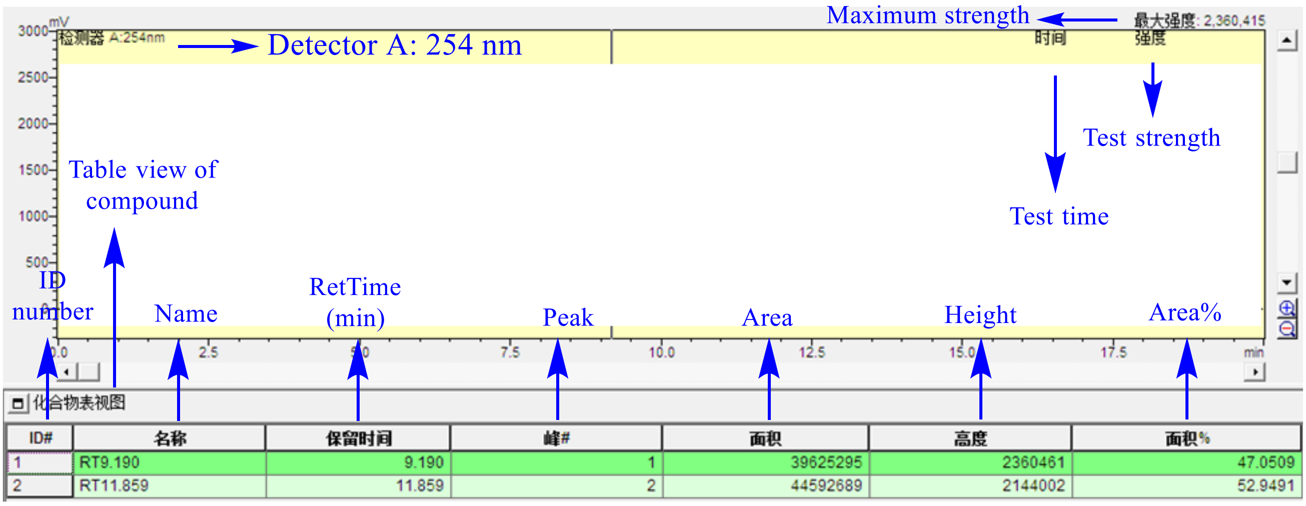

ID#	名称	保留时间	峰#	面积	高度	面积%
1	RT9.190	9.190	1	39625295	2360461	47.0509
2	RT11.859	11.859	2	44592689	2144002	52.9491

(*R*)-1w: (R)-5'-bromo-2'-phenylspiro[cyclohexane-1,3'-indoline]: (HPLC: Chiracel IC-H, detected at 254 nm, eluent: n-hexane/2-propanol = 97/03, flow rate = 0.5mL/min, 25 ℃).

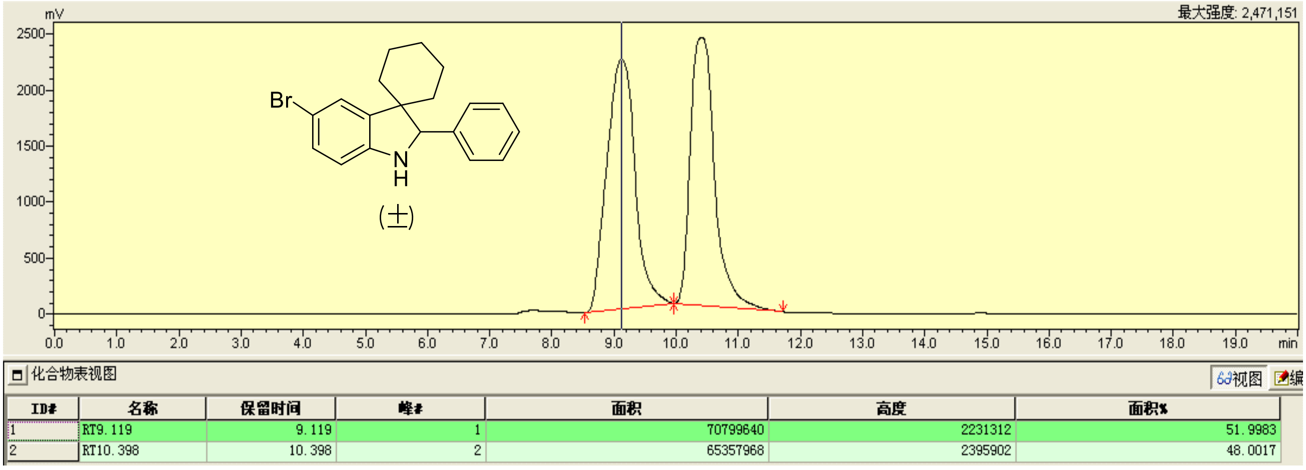

ID#	名称	保留时间	峰#	面积	高度	面积%
1	RT9.119	9.119	1	70799640	2231312	51.9983
2	RT10.398	10.398	2	65357968	2395902	48.0017

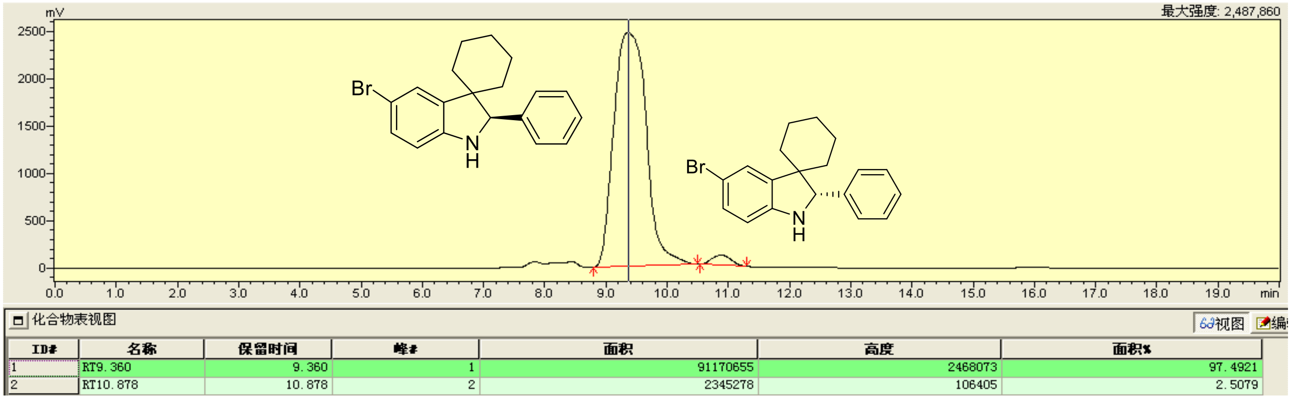

ID#	名称	保留时间	峰#	面积	高度	面积%
1	RT9.360	9.360	1	91170655	2468073	97.4921
2	RT10.878	10.878	2	2345278	106405	2.5079

<u>Translation of all characters (Chinese) in the above two frameworks to English is as follows:</u>

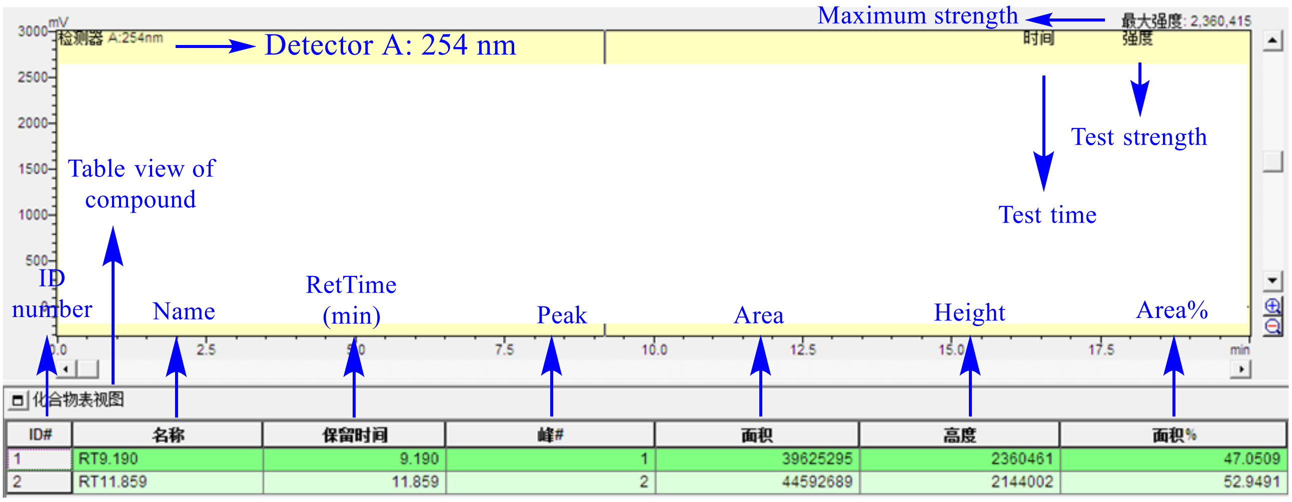

ID#	名称	保留时间	峰#	面积	高度	面积%
1	RT9.190	9.190	1	39625295	2360461	47.0509
2	RT11.859	11.859	2	44592689	2144002	52.9491

(*R*)-1x: (R)-6'-bromo-2'-phenylspiro[cyclohexane-1,3'-indoline].: (HPLC: Chiracel IC-H, detected at 254 nm, eluent: n-hexane/2-propanol = 97/03, flow rate = 0.5mL/min, 25 ℃).

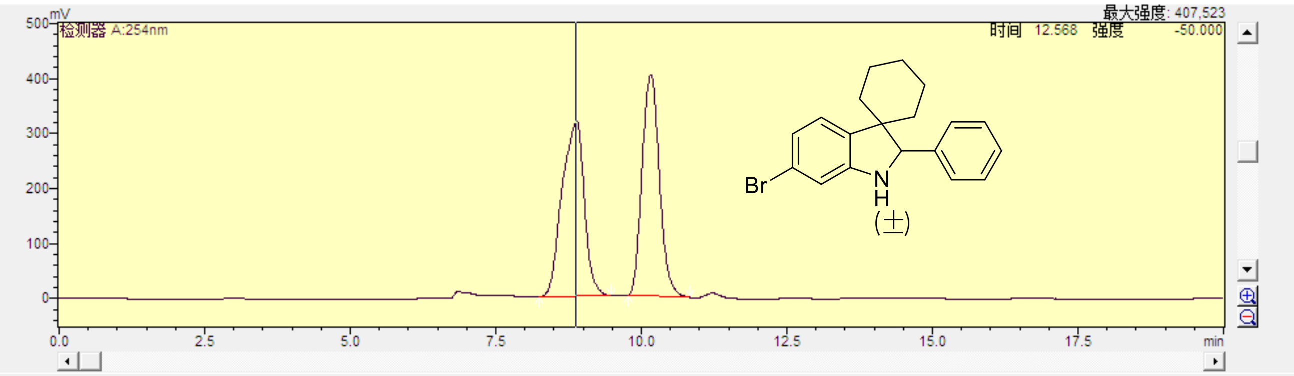

化合物表视图

ID#	名称	保留时间	峰#	面积	高度	面积%
1	RT8.882	8.882	1	8467066	319196	50.1902
2	RT10.159	10.159	2	8402887	404009	49.8098

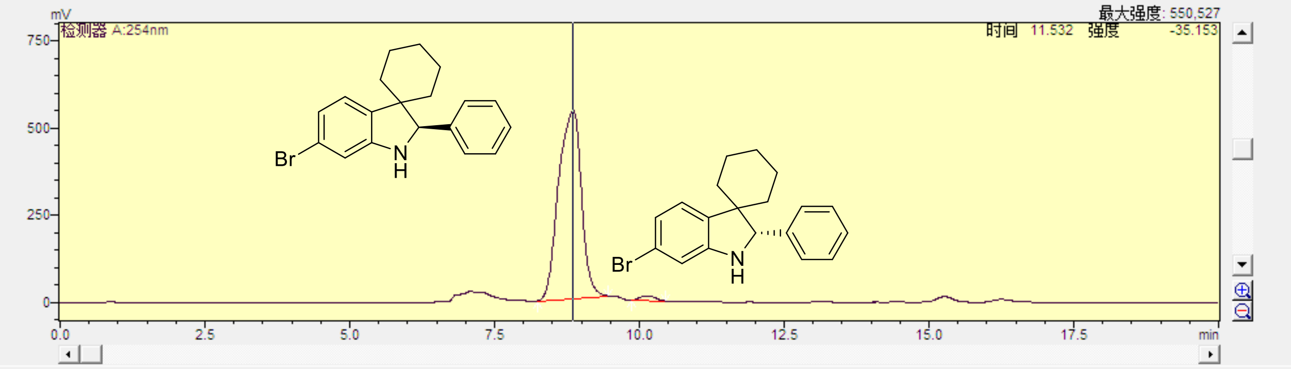

化合物表视图

ID#	名称	保留时间	峰#	面积	高度	面积%
1	RT8.853	8.853	1	14813765	539588	98.1593
2	RT10.141	10.141	2	277785	14863	1.8407

Translation of all characters (Chinese) in the above two frameworks to English is as follows:

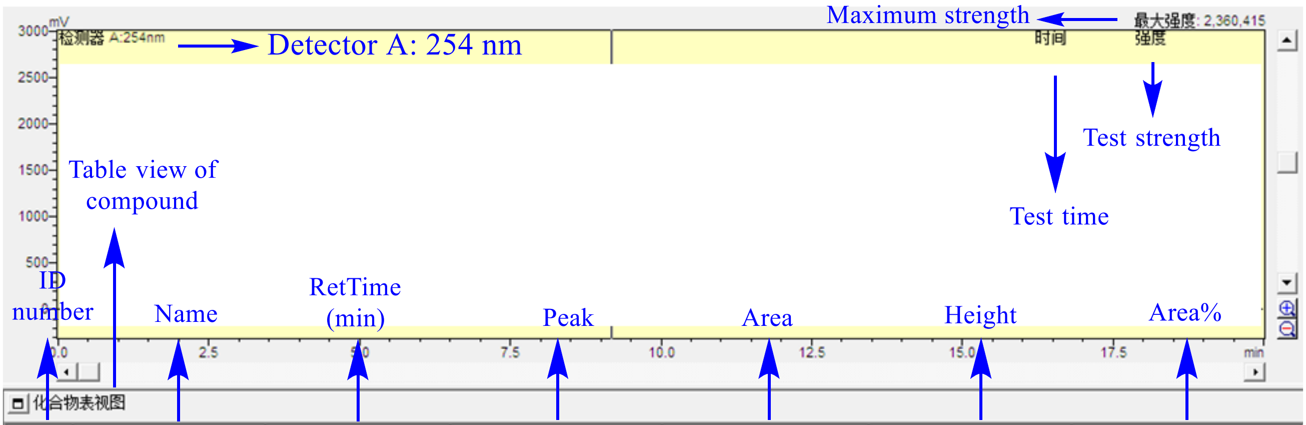

ID#	名称	保留时间	峰#	面积	高度	面积%
1	RT9.190	9.190	1	39625295	2360461	47.0509
2	RT11.859	11.859	2	44592689	2144002	52.9491

(*R*)-1y: (R)-2'-phenyl-5'-(trifluoromethyl)spiro[cyclohexane-1,3'-indoline].: (HPLC: Chiracel OD-H, detected at 254 nm, eluent: n-hexane/2-propanol = 90/10, flow rate = 1.0mL/min, 25 ℃).

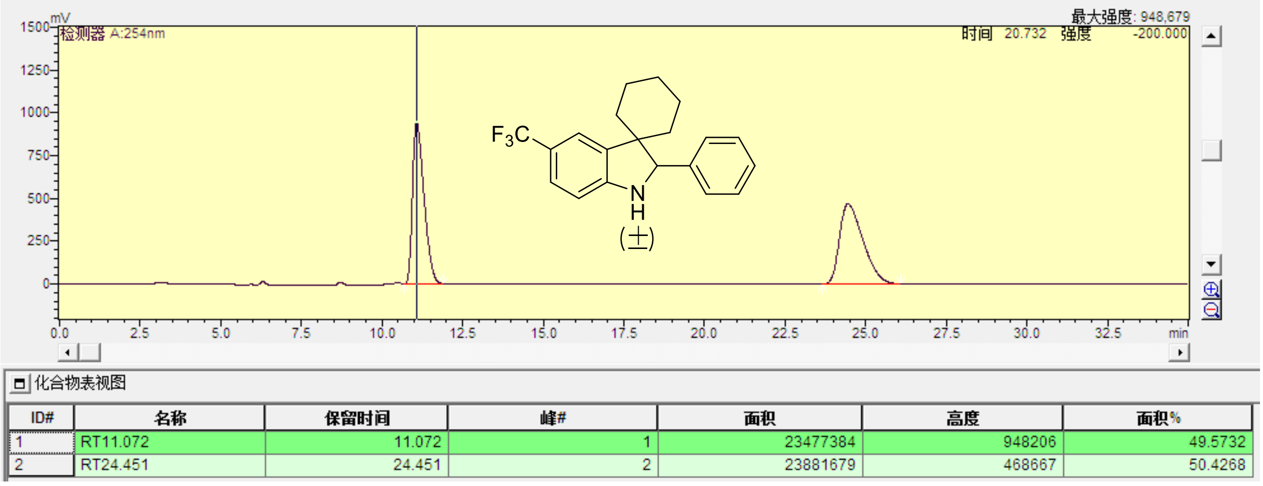

ID#	名称	保留时间	峰#	面积	高度	面积%
1	RT11.072	11.072	1	23477384	948206	49.5732
2	RT24.451	24.451	2	23881679	468667	50.4268

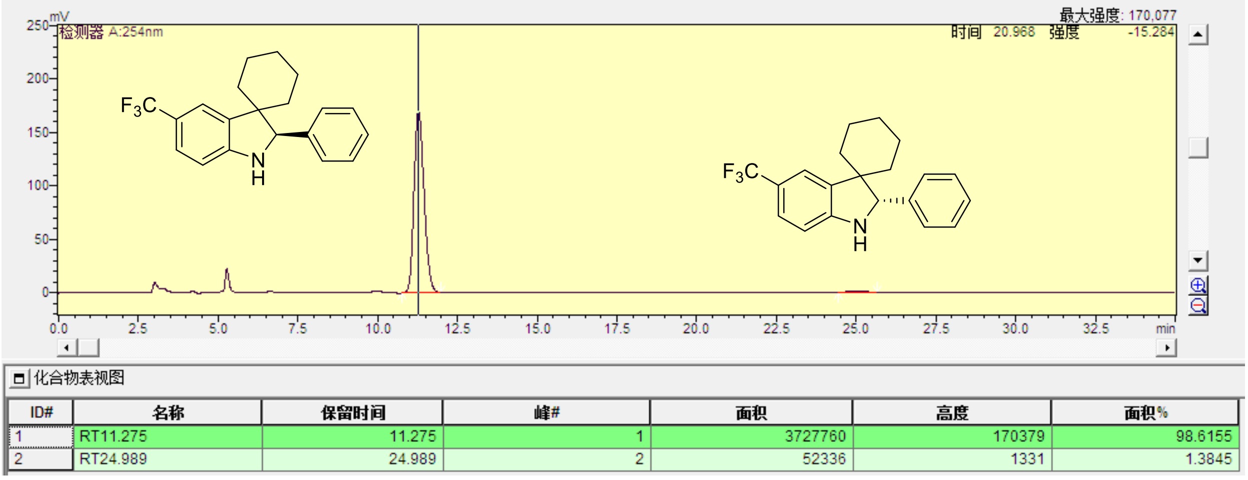

ID#	名称	保留时间	峰#	面积	高度	面积%
1	RT11.275	11.275	1	3727760	170379	98.6155
2	RT24.989	24.989	2	52336	1331	1.3845

<u>Translation of all characters (Chinese) in the above two frameworks to English is as follows:</u>

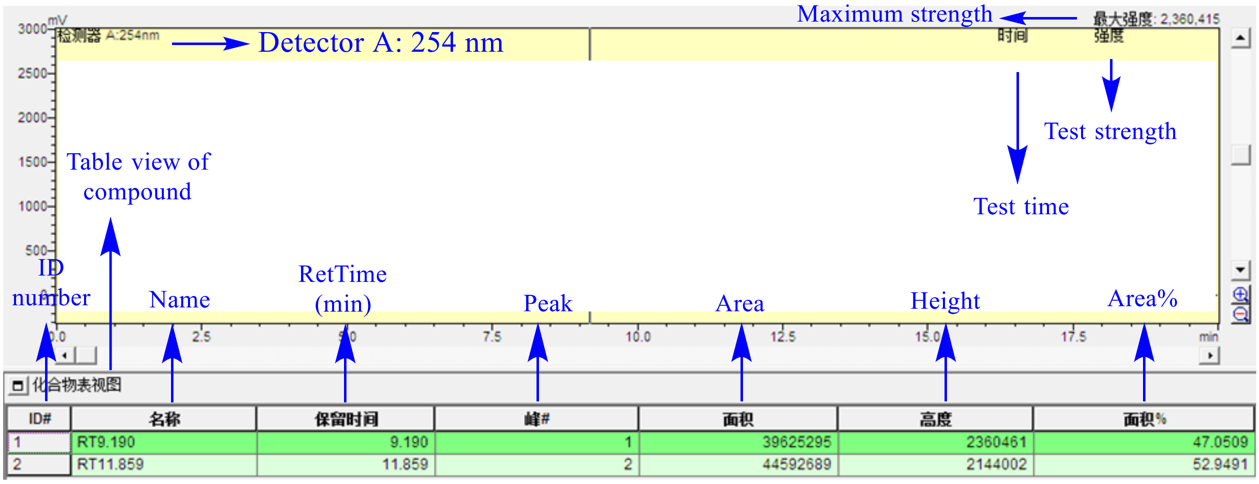

ID#	名称	保留时间	峰#	面积	高度	面积%
1	RT9.190	9.190	1	39625295	2360461	47.0509
2	RT11.859	11.859	2	44592689	2144002	52.9491

(*R*)-1z: (R)-2'-phenyl-5'-(trifluoromethoxy)spiro[cyclohexane-1,3'-indoline].: (HPLC: Chiracel OD-H, detected at 254 nm, eluent: n-hexane/2-propanol = 90/10, flow rate = 1.0mL/min, 25 ℃).

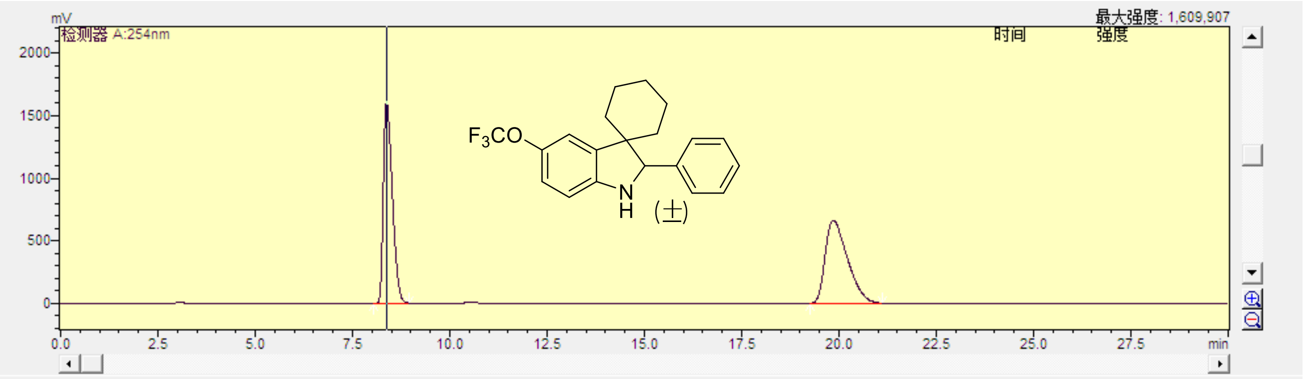

化合物表视图

ID#	名称	保留时间	峰#	面积	高度	面积%
1	RT8.374	8.374	1	25520515	1608437	49.2882
2	RT19.849	19.849	2	26257605	660549	50.7118

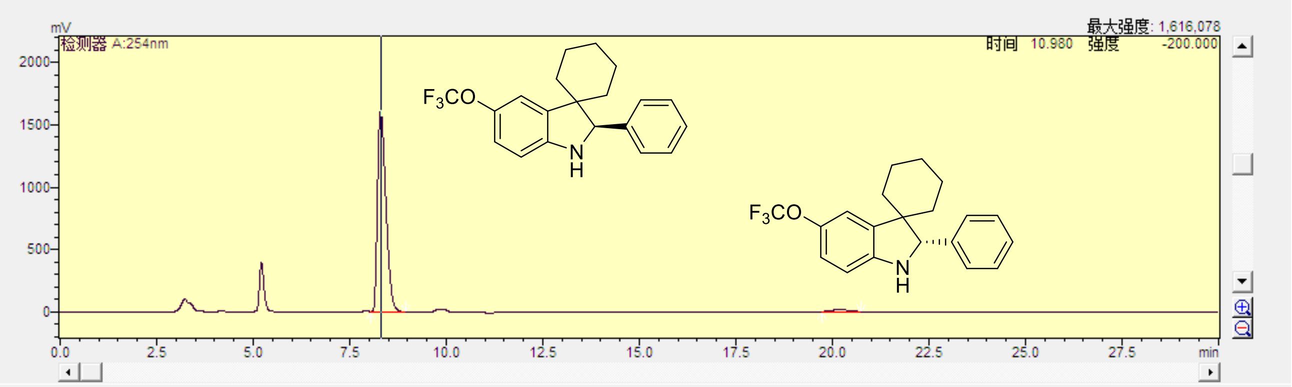

化合物表视图

ID#	名称	保留时间	峰#	面积	高度	面积%
1	RT8.298	8.298	1	24120239	1612582	97.9188
2	RT20.183	20.183	2	512651	16143	2.0812

__Translation of all characters (Chinese) in the above two frameworks to English is as follows:__

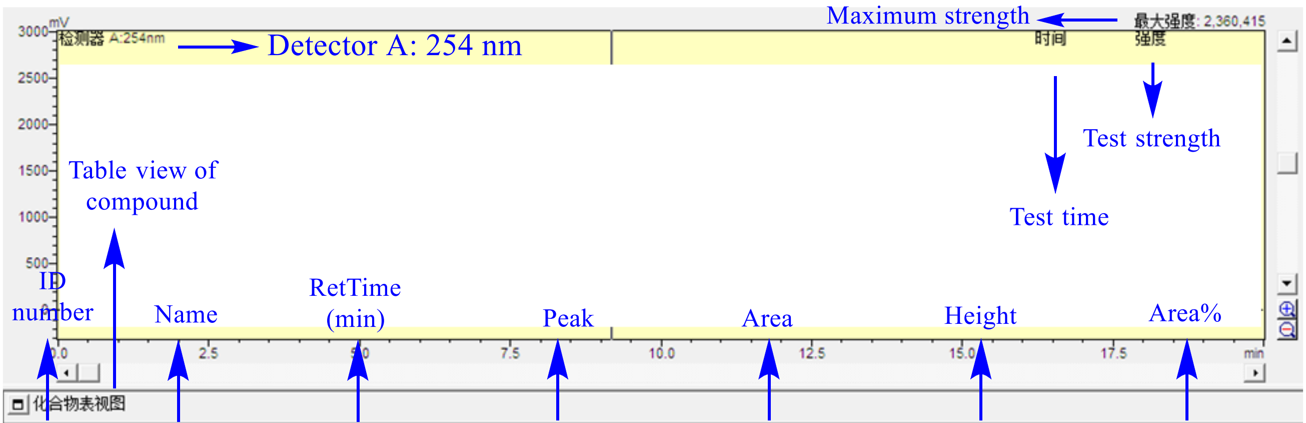

化合物表视图

ID#	名称	保留时间	峰#	面积	高度	面积%
1	RT9.190	9.190	1	39625295	2360461	47.0509
2	RT11.859	11.859	2	44592689	2144002	52.9491

(*R*)-1z': (R)- 5'-methoxy-2'-phenylspiro[cyclohexane-1,3'-indoline]: (HPLC: Chiracel OD-H, detected at 254 nm, eluent: n-hexane/2-propanol = 90/10, flow rate = 1.0mL/min, 25 ℃).

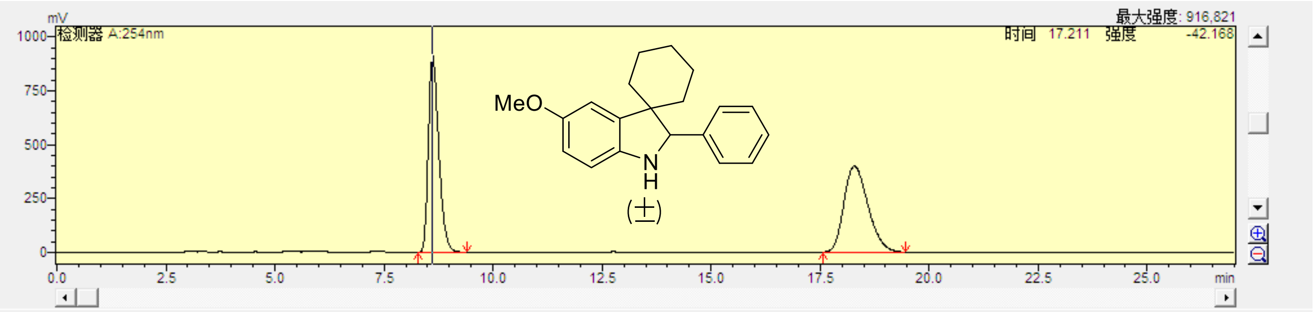

化合物表视图

ID#	名称	保留时间	峰#	面积	高度	面积%
1	RT8.612	8.612	1	15228187	915696	49.7585
2	RT18.283	18.283	2	15375993	400170	50.2415

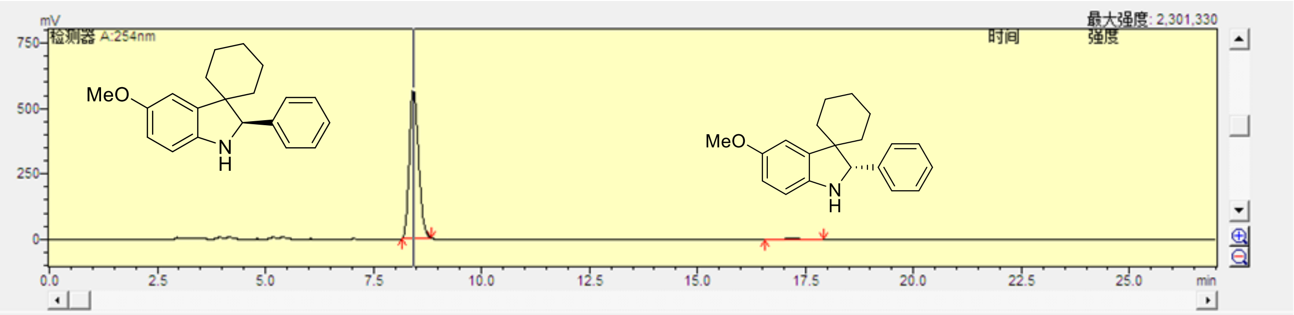

化合物表视图

ID#	名称	保留时间	峰#	面积	高度	面积%
1	RT8.423	8.423	1	8534571	572818	98.3296
2	RT17.193	17.193	2	144986	4523	1.6704

<u>**Translation of all characters (Chinese) in the above two frameworks to English is as follows:**</u>

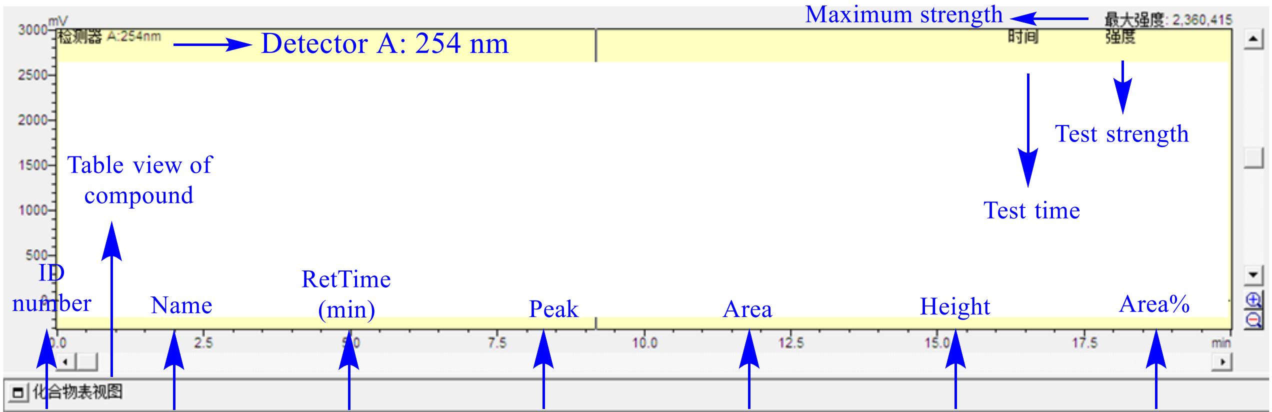

ID#	名称	保留时间	峰#	面积	高度	面积%
1	RT9.190	9.190	1	39625295	2360461	47.0509
2	RT11.859	11.859	2	44592689	2144002	52.9491

(*S*)-3a: (S)-2-phenyl-1,2,3,4-tetrahydroquinoline: (HPLC: Chiracel OD-H, detected at 254 nm, eluent: n-hexane/2-propanol = 90/10, flow rate = 1mL/min, 25 ℃).

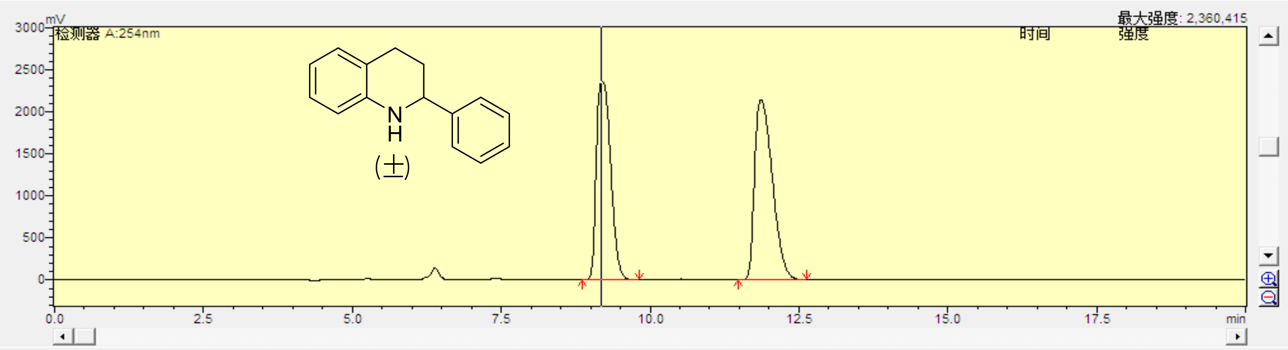

化合物表视图

ID#	名称	保留时间	峰#	面积	高度	面积%
1	RT9.190	9.190	1	39625295	2360461	47.0509
2	RT11.859	11.859	2	44592689	2144002	52.9491

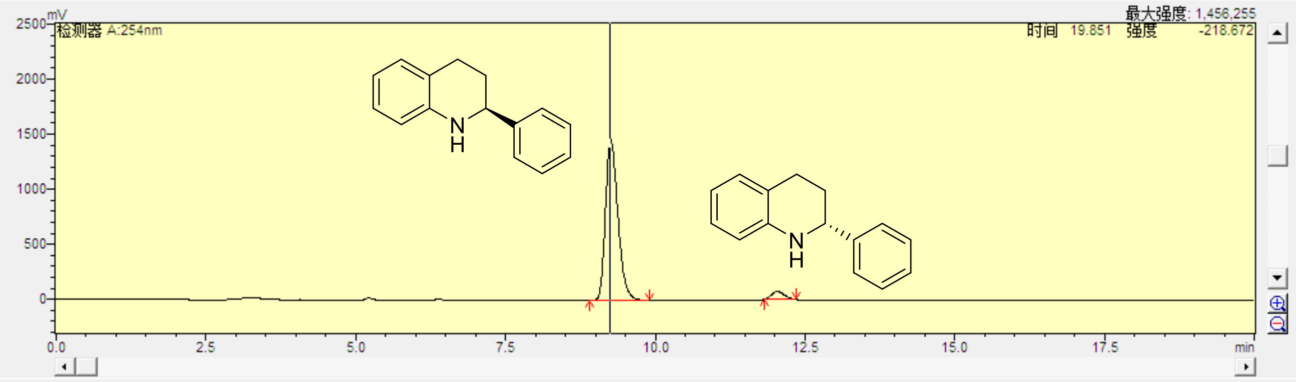

化合物表视图

ID#	名称	保留时间	峰#	面积	高度	面积%
1	RT9.254	9.254	1	21071970	1465261	95.1329
2	RT12.037	12.037	2	1078069	71744	4.8671

<u>Translation of all characters (Chinese) in the above two frameworks to English is as follows:</u>

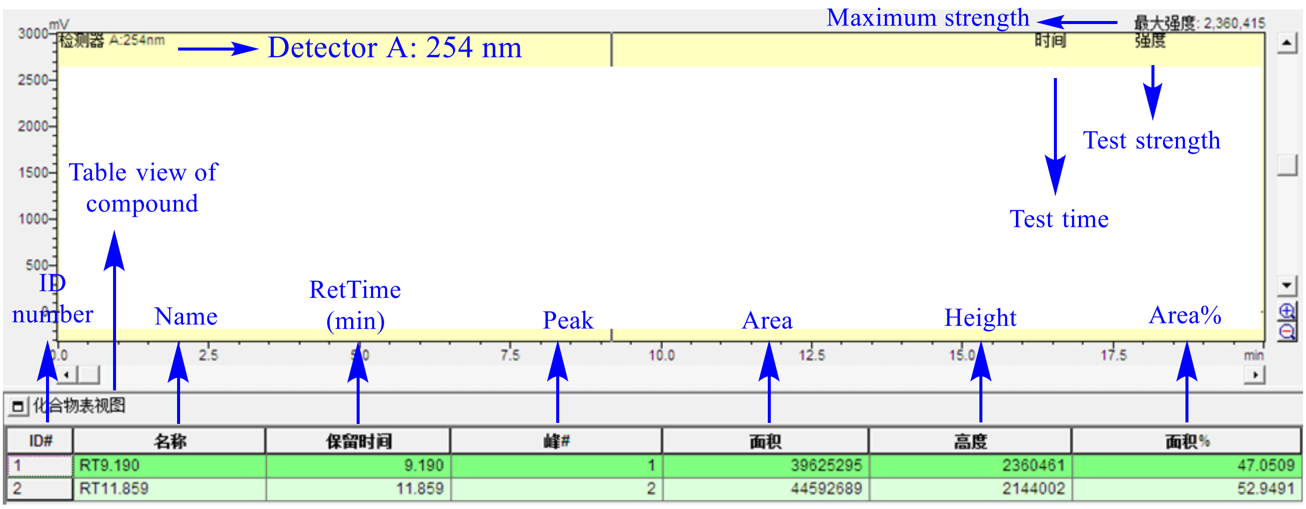

(*S*)-3b: (S)-2-(4-fluorophenyl)-1,2,3,4-tetrahydroquinoline: (HPLC: Chiracel OD-H, detected at 254 nm, eluent: n-hexane/2-propanol = 90/10, flow rate = 1mL/min, 25 ℃).

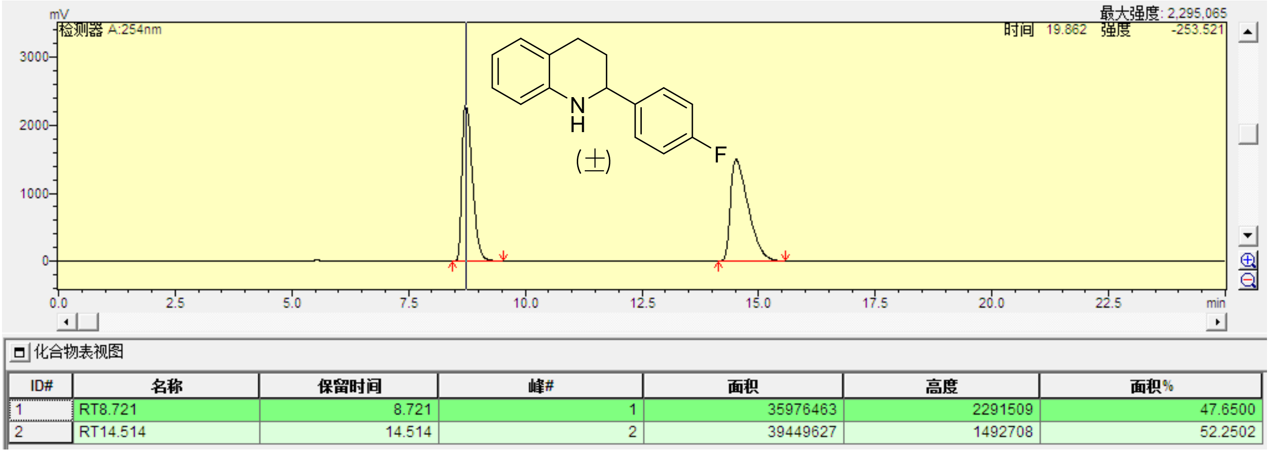

ID#	名称	保留时间	峰#	面积	高度	面积%
1	RT8.721	8.721	1	35976463	2291509	47.6500
2	RT14.514	14.514	2	39449627	1492708	52.2502

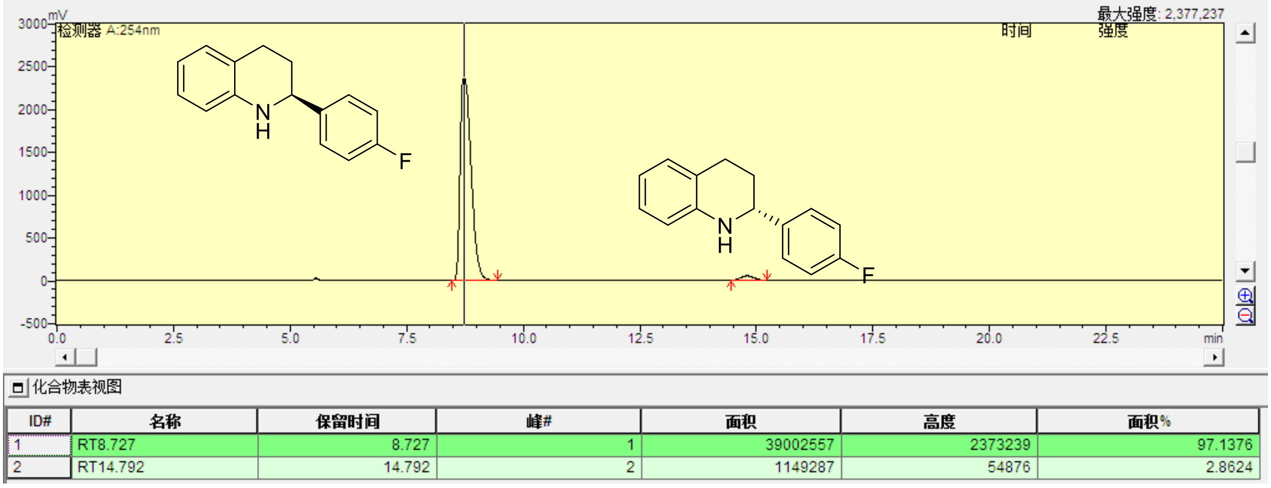

ID#	名称	保留时间	峰#	面积	高度	面积%
1	RT8.727	8.727	1	39002557	2373239	97.1376
2	RT14.792	14.792	2	1149287	54876	2.8624

<u>Translation of all characters (Chinese) in the above two frameworks to English is as follows:</u>

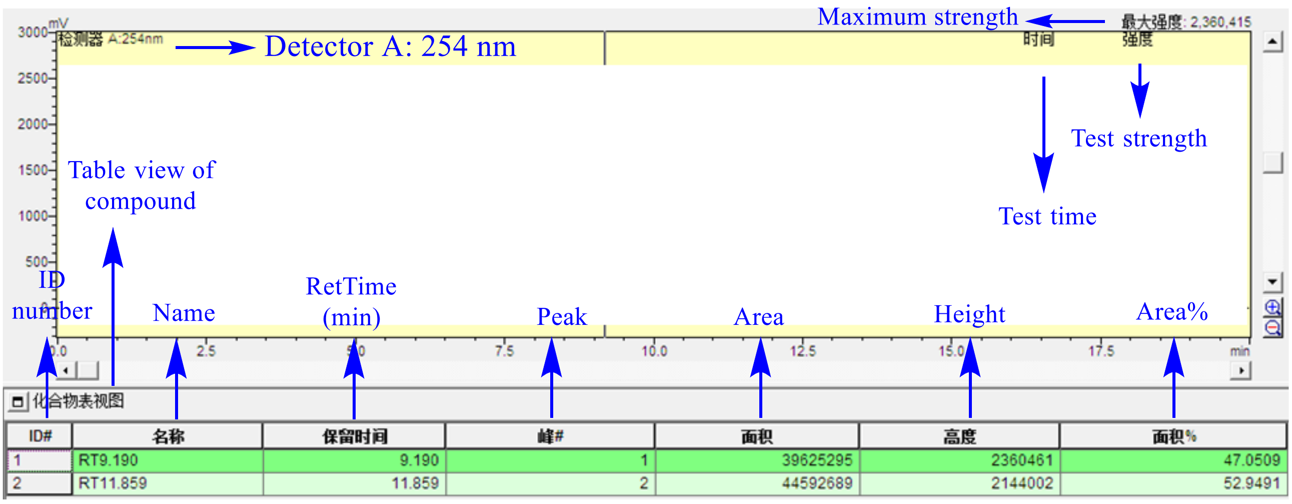

ID#	名称	保留时间	峰#	面积	高度	面积%
1	RT9.190	9.190	1	39625295	2360461	47.0509
2	RT11.859	11.859	2	44592689	2144002	52.9491

(*S*)-3c: (S)-2-(4-(trifluoromethyl)phenyl)-1,2,3,4-tetrahydroquinoline: (HPLC: Chiracel OD-H, detected at 254 nm, eluent: n-hexane/2-propanol = 90/10, flow rate = 1mL/min, 25 ℃).

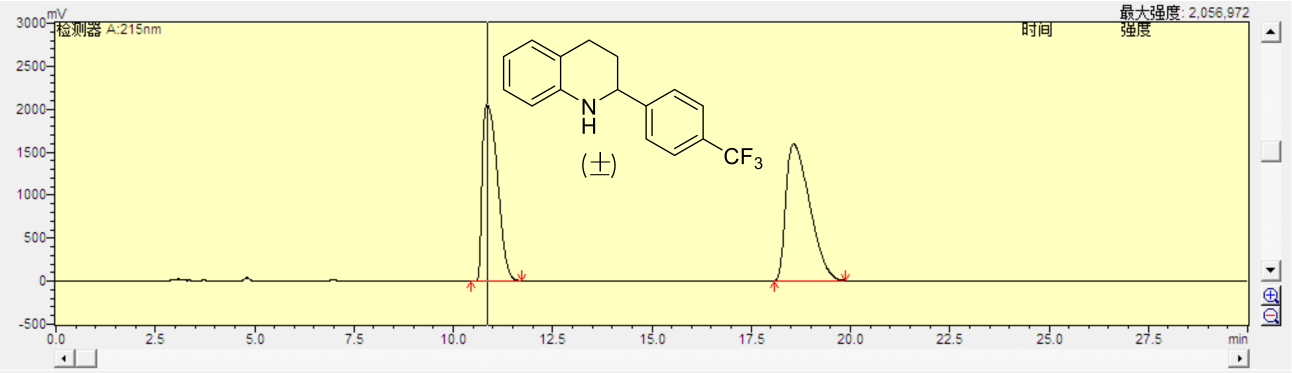

化合物表视图

ID#	名称	保留时间	峰#	面积	高度	面积%
1	RT10.848	10.848	1	54659397	2054311	45.2190
2	RT18.569	18.569	2	66217677	1589640	54.7810

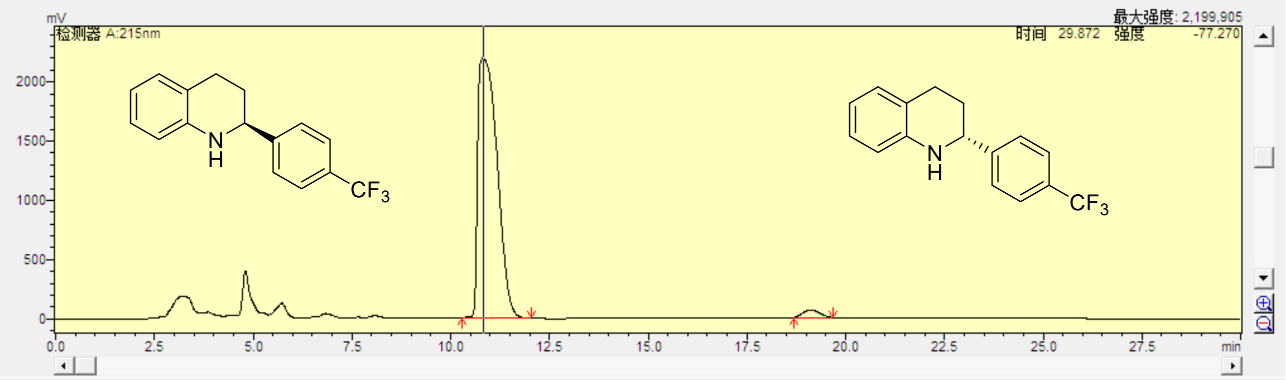

化合物表视图

ID#	名称	保留时间	峰#	面积	高度	面积%
1	RT10.818	10.818	1	74898433	2191904	97.4214
2	RT19.113	19.113	2	1982435	65963	2.5786

<u>**Translation of all characters (Chinese) in the above two frameworks to English is as follows:**</u>

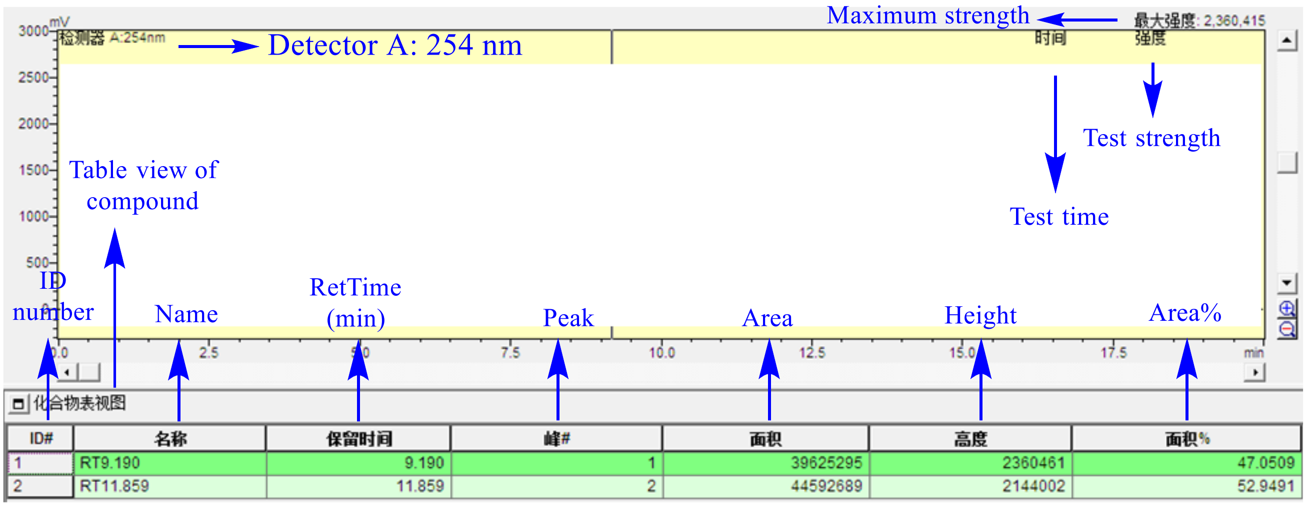

化合物表视图

ID#	名称	保留时间	峰#	面积	高度	面积%
1	RT9.190	9.190	1	39625295	2360461	47.0509
2	RT11.859	11.859	2	44592689	2144002	52.9491

(*S*)-3d: (S)-4-(1,2,3,4-tetrahydroquinolin-2-yl)benzonitrile: (HPLC: Chiracel OD-H, detected at 254 nm, eluent: n-hexane/2-propanol = 80/20, flow rate = 1mL/min, 25 ℃).

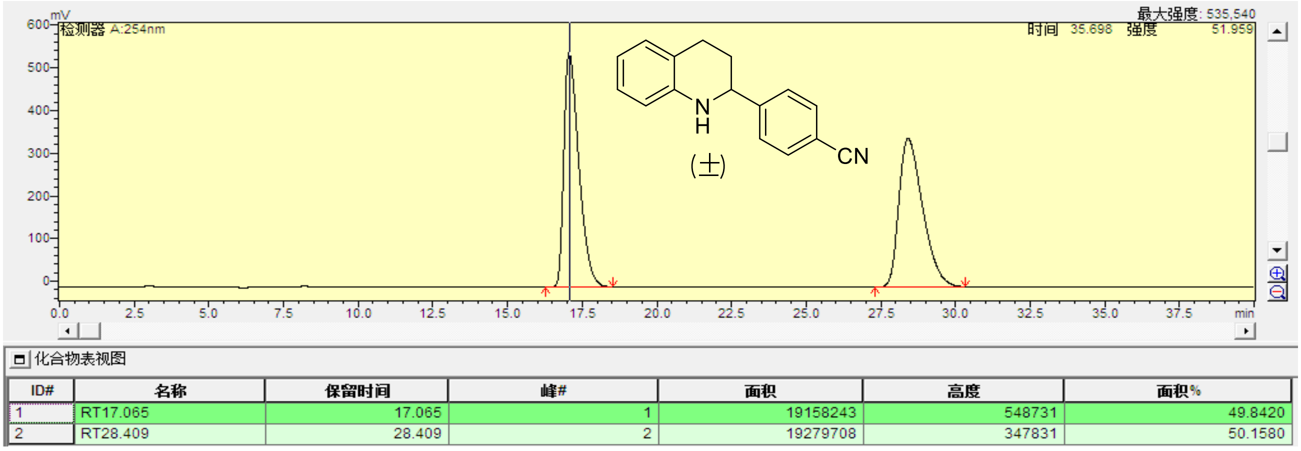

ID#	名称	保留时间	峰#	面积	高度	面积%
1	RT17.065	17.065	1	19158243	548731	49.8420
2	RT28.409	28.409	2	19279708	347831	50.1580

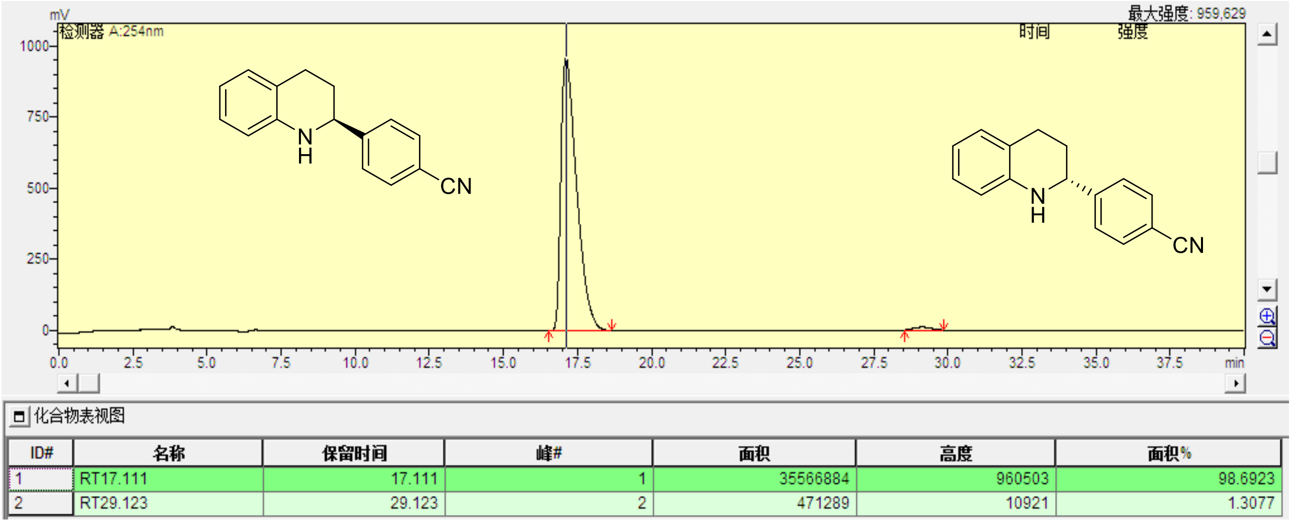

ID#	名称	保留时间	峰#	面积	高度	面积%
1	RT17.111	17.111	1	35566884	960503	98.6923
2	RT29.123	29.123	2	471289	10921	1.3077

<u>Translation of all characters (Chinese) in the above two frameworks to English is as follows:</u>

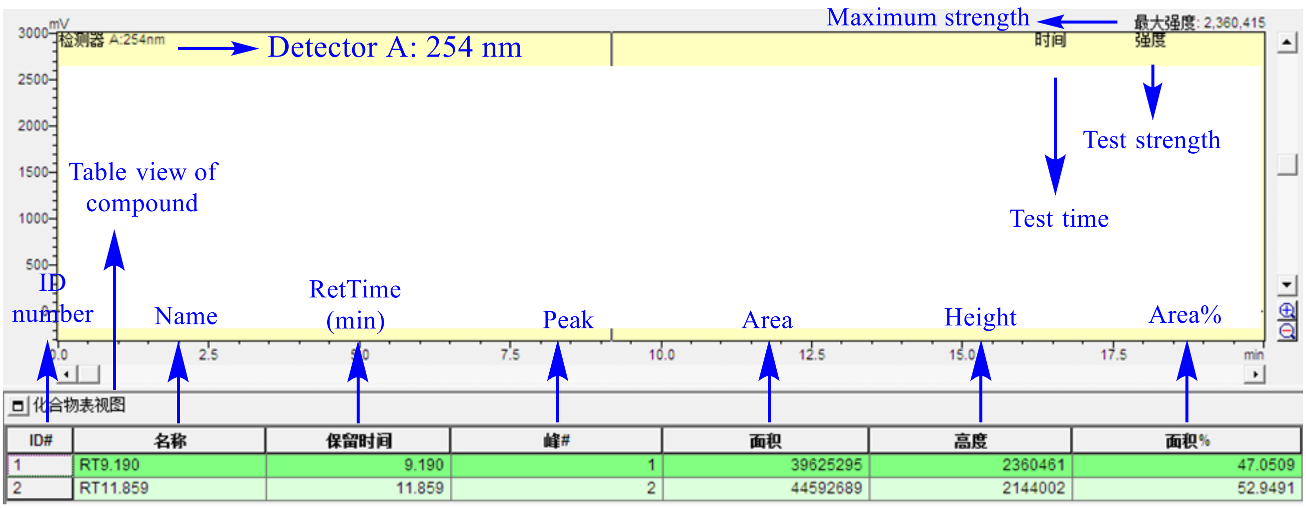

ID#	名称	保留时间	峰#	面积	高度	面积%
1	RT9.190	9.190	1	39625295	2360461	47.0509
2	RT11.859	11.859	2	44592689	2144002	52.9491

(*S*)-3e: (S)-2-(3-nitrophenyl)-1,2,3,4-tetrahydroquinoline: (HPLC: Chiracel OD-H, detected at 254 nm, eluent: n-hexane/2-propanol = 90/10, flow rate = 1mL/min, 25 ℃).

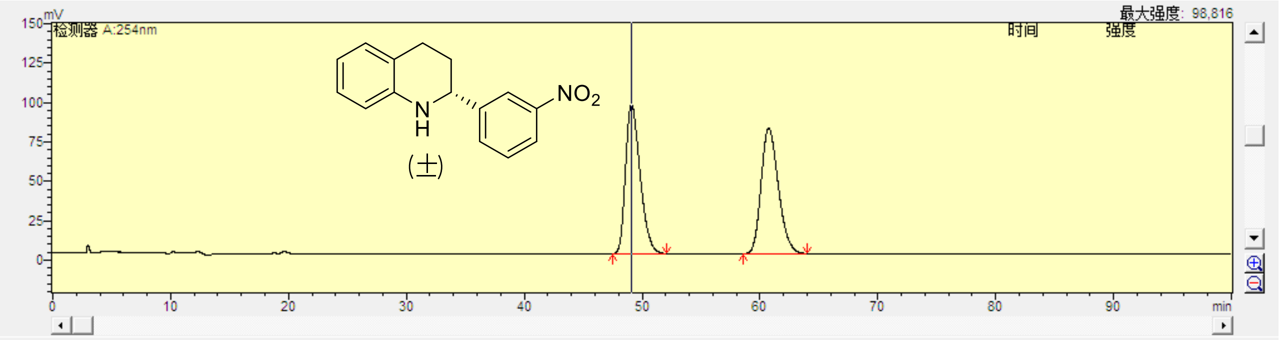

化合物表视图

ID#	名称	保留时间	峰#	面积	高度	面积%
1	RT49.096	49.096	1	8126420	94666	50.0018
2	RT60.735	60.735	2	8125836	79746	49.9982

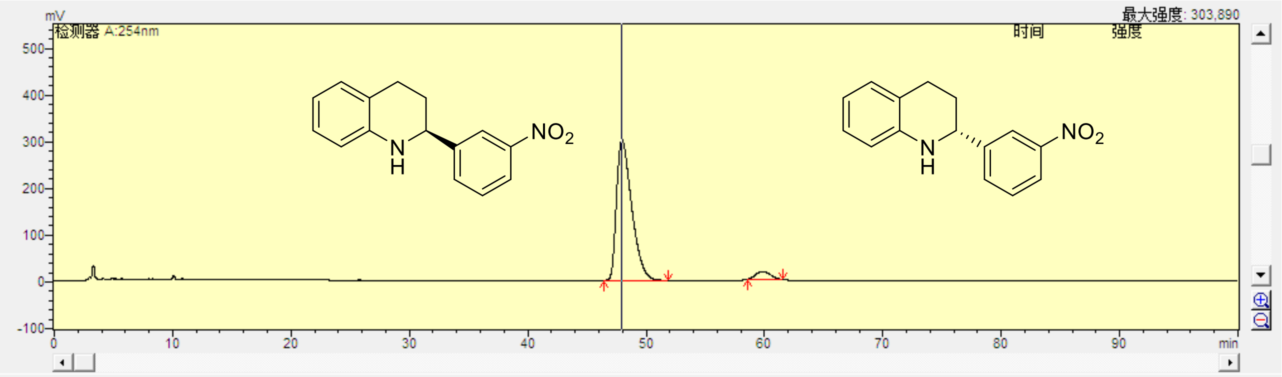

化合物表视图

ID#	名称	保留时间	峰#	面积	高度	面积%
1	RT47.956	47.956	1	26762608	300406	94.7812
2	RT59.835	59.835	2	1473603	16814	5.2188

Translation of all characters (Chinese) in the above two frameworks to English is as follows:

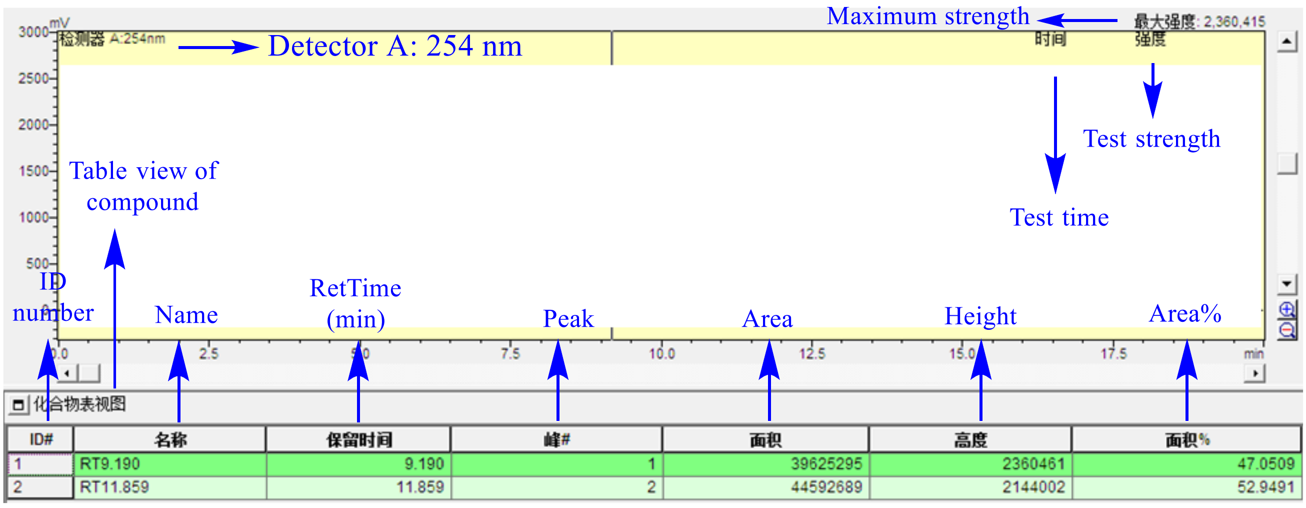

化合物表视图

ID#	名称	保留时间	峰#	面积	高度	面积%
1	RT9.190	9.190	1	39625295	2360461	47.0509
2	RT11.859	11.859	2	44592689	2144002	52.9491

(*S*)-3f: methyl (S)-4-(1,2,3,4-tetrahydroquinolin-2-yl)benzoate.: (HPLC: Chiracel OD-H, detected at 254 nm, eluent: n-hexane/2-propanol = 90/10, flow rate = 1.0mL/min, 25 ℃).

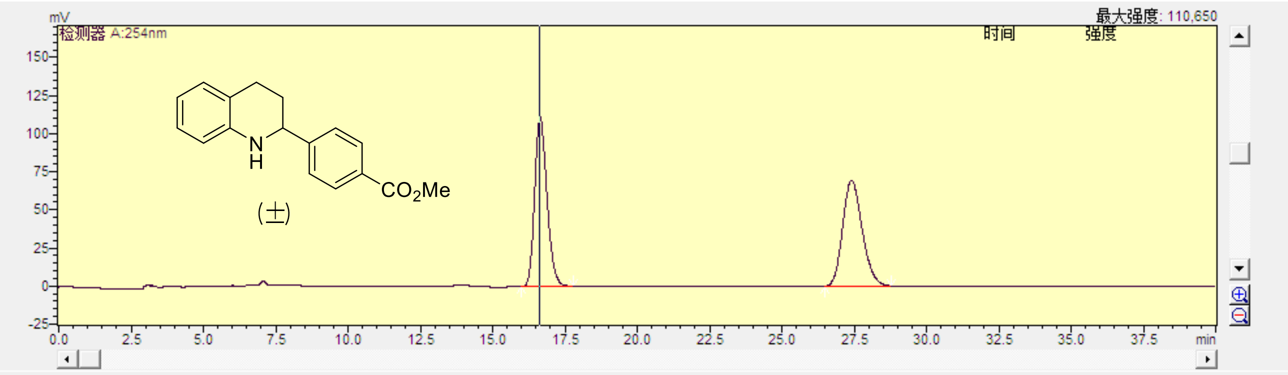

化合物表视图

ID#	名称	保留时间	峰#	面积	高度	面积%
1	RT16.641	16.641	1	3271803	110804	50.1150
2	RT27.405	27.405	2	3256788	69497	49.8850

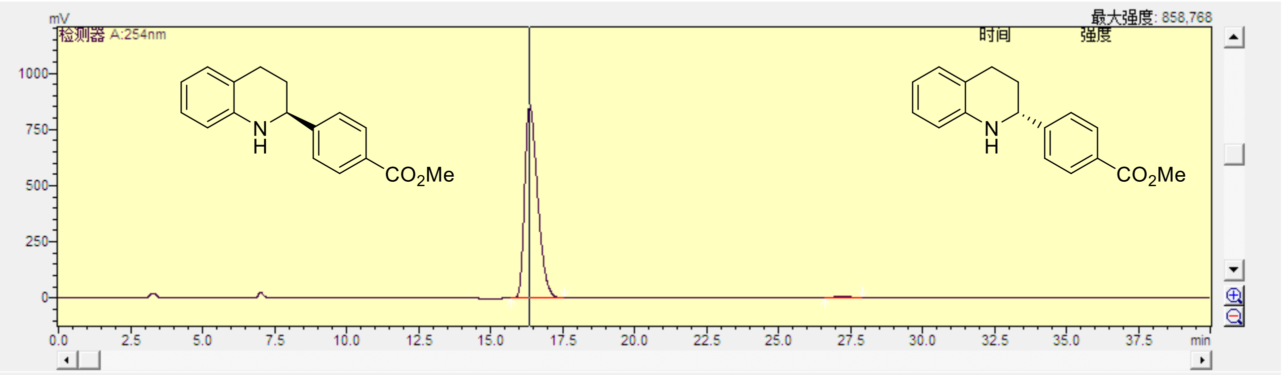

化合物表视图

ID#	名称	保留时间	峰#	面积	高度	面积%
1	RT16.366	16.366	1	26766476	858838	99.0611
2	RT27.195	27.195	2	253705	6361	0.9389

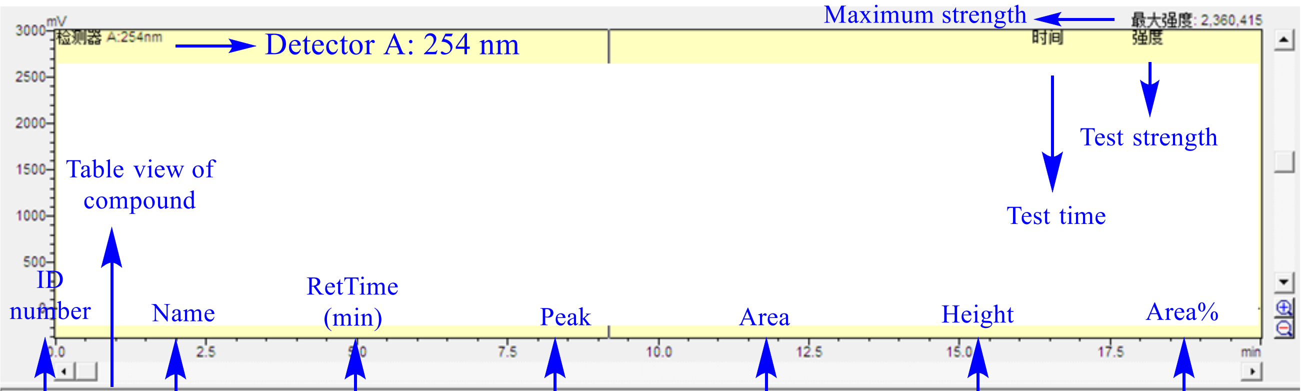

化合物表视图

ID#	名称	保留时间	峰#	面积	高度	面积%
1	RT9.190	9.190	1	39625295	2360461	47.0509
2	RT11.859	11.859	2	44592689	2144002	52.9491

(*S*)-3g: (S)-2-(p-tolyl)-1,2,3,4-tetrahydroquinoline: (HPLC: Chiracel OD-H, detected at 254 nm, eluent: n-hexane/2-propanol = 90/10, flow rate = 1mL/min, 25 ℃).

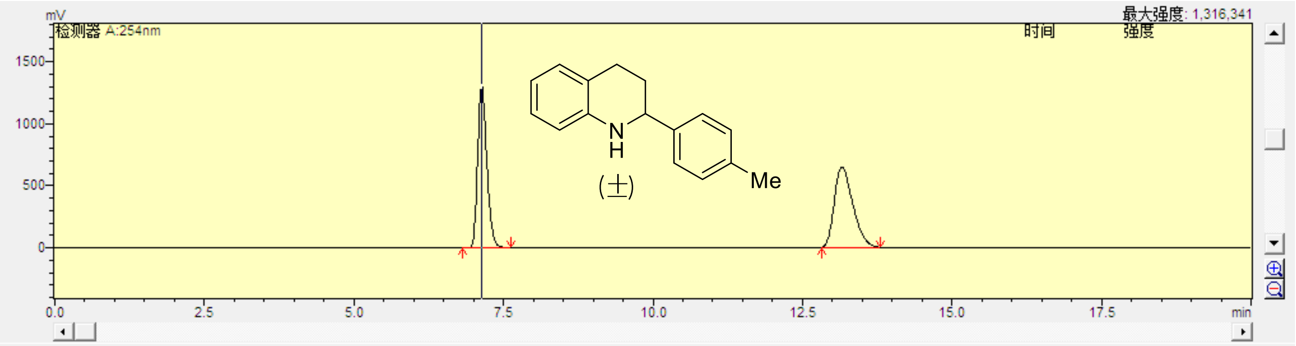

化合物表视图

ID#	名称	保留时间	峰#	面积	高度	面积%
1	RT7.132	7.132	1	14089864	1317752	50.0993
2	RT13.154	13.154	2	14034007	647553	49.9007

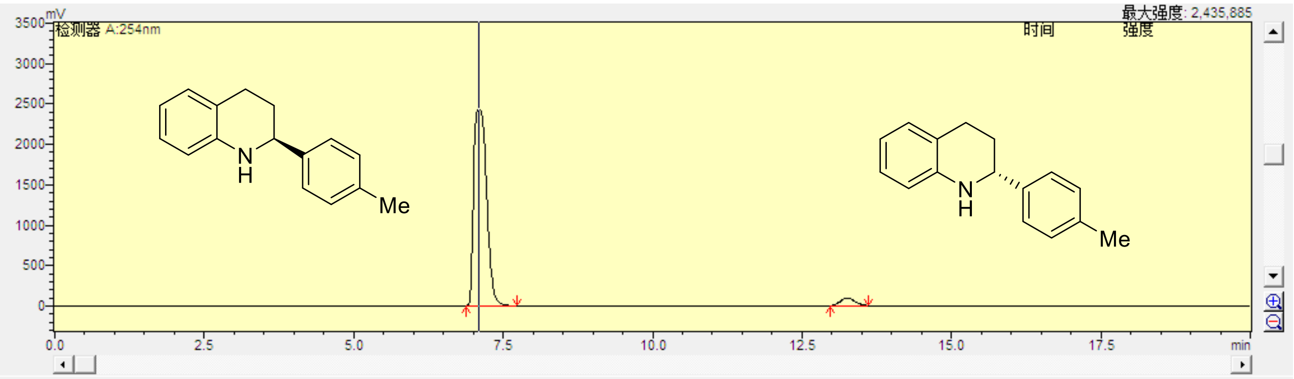

化合物表视图

ID#	名称	保留时间	峰#	面积	高度	面积%
1	RT7.087	7.087	1	36645117	2435607	95.6481
2	RT13.251	13.251	2	1667328	96781	4.3519

<u>Translation of all characters (Chinese) in the above two frameworks to English is as follows:</u>

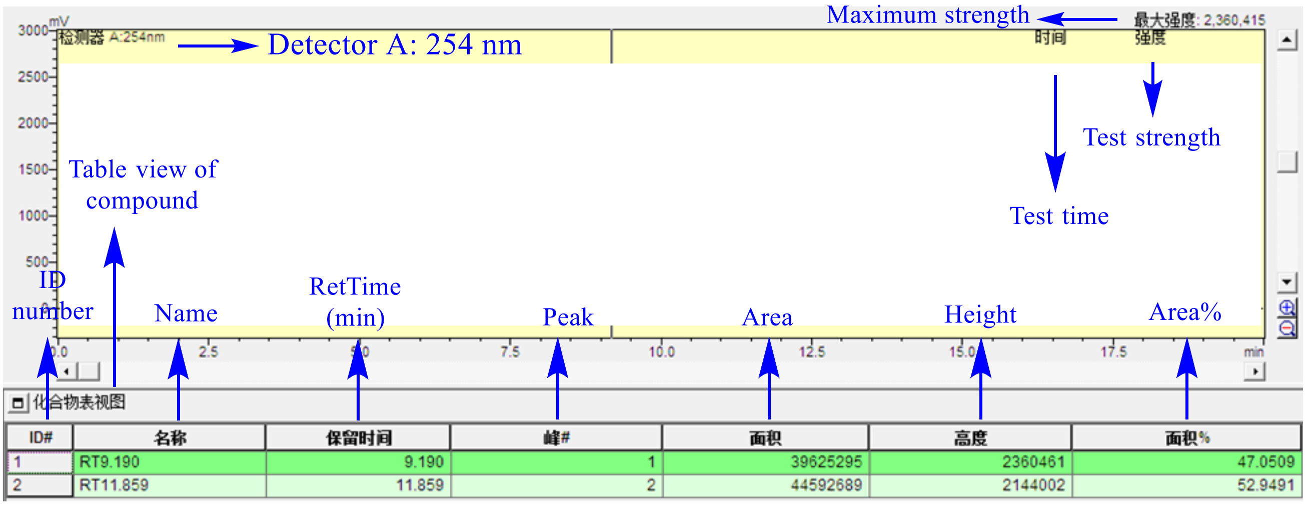

ID#	名称	保留时间	峰#	面积	高度	面积%
1	RT9.190	9.190	1	39625295	2360461	47.0509
2	RT11.859	11.859	2	44592689	2144002	52.9491

(*S*)-3h: (S)-2-(m-tolyl)-1,2,3,4-tetrahydroquinoline: (HPLC: Chiracel OD-H, detected at 254 nm, eluent: n-hexane/2-propanol = 90/10, flow rate = 1mL/min, 25 ℃).

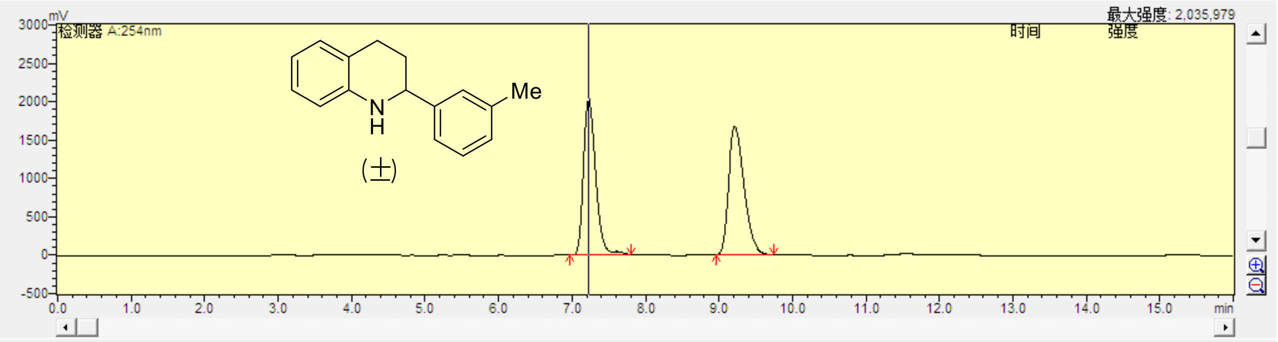

ID#	名称	保留时间	峰#	面积	高度	面积%
1	RT7.223	7.223	1	24018538	2033401	49.3728
2	RT9.214	9.214	2	24628739	1672524	50.6272

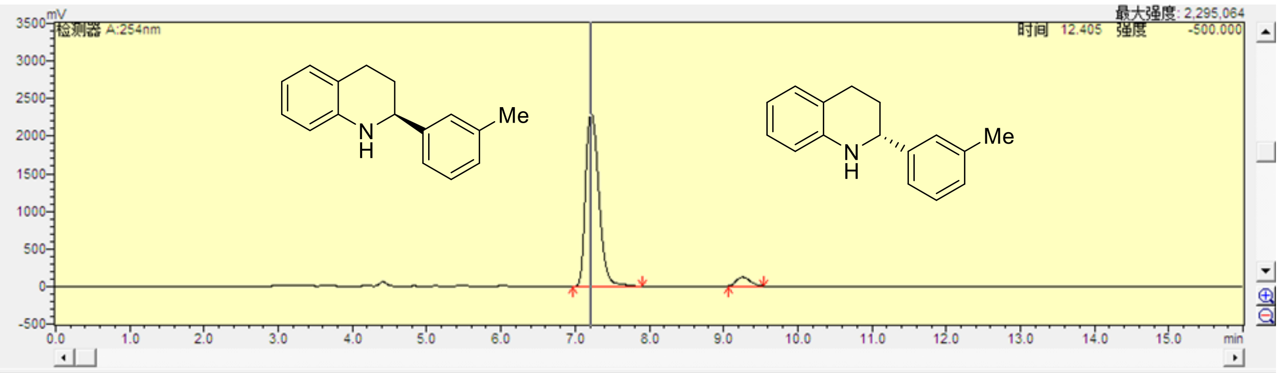

ID#	名称	保留时间	峰#	面积	高度	面积%
1	RT7.213	7.213	1	29375483	2292808	95.0167
2	RT9.255	9.255	2	1540635	119026	4.9833

<u>**Translation of all characters (Chinese) in the above two frameworks to English is as follows:**</u>

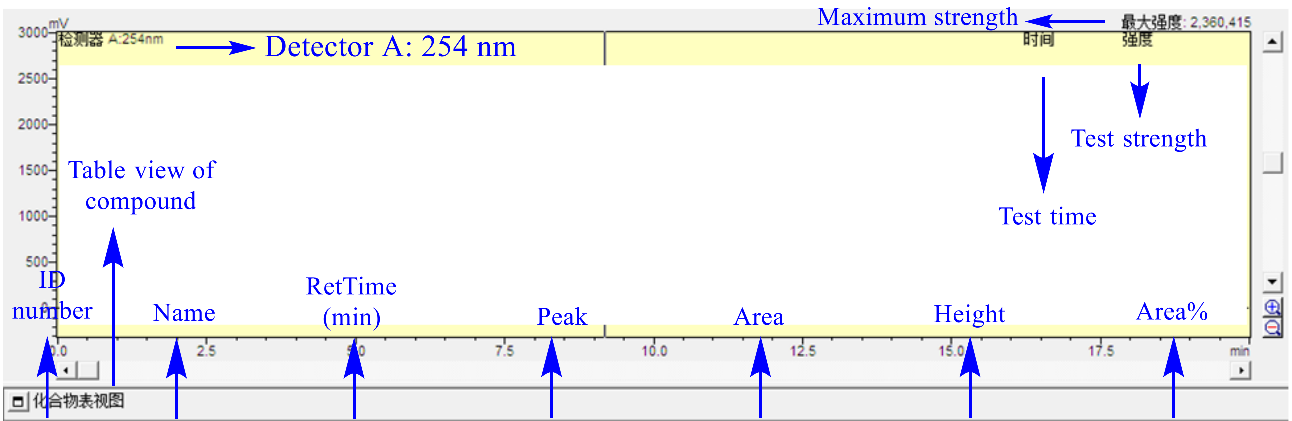

ID#	名称	保留时间	峰#	面积	高度	面积%
1	RT9.190	9.190	1	39625295	2360461	47.0509
2	RT11.859	11.859	2	44592689	2144002	52.9491

(*S*)-3i: (S)-2-(4-methoxyphenyl)-1,2,3,4-tetrahydroquinoline: (HPLC: Chiracel OD-H, detected at 254 nm, eluent: n-hexane/2-propanol = 90/10, flow rate = 1mL/min, 25 ℃).

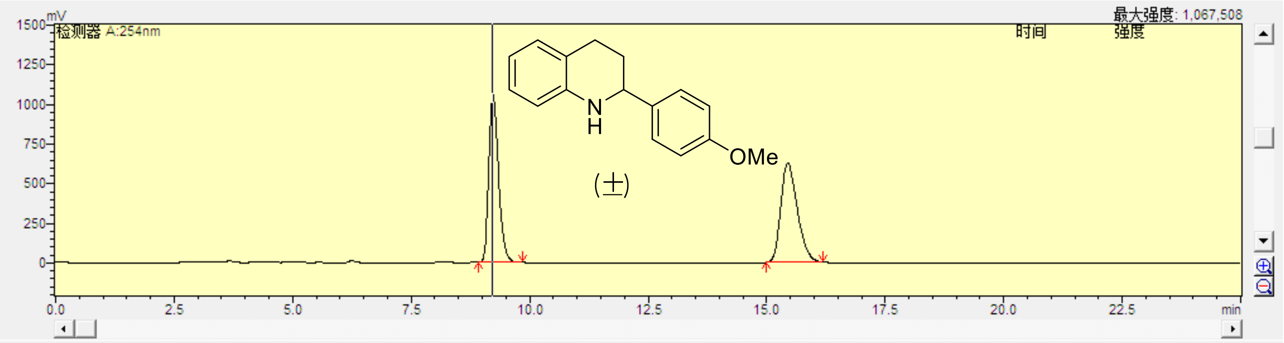

□ 化合物表视图

ID#	名称	保留时间	峰#	面积	高度	面积%
1	RT9.222	9.222	1	15186047	1063229	49.9242
2	RT15.442	15.442	2	15232185	624860	50.0758

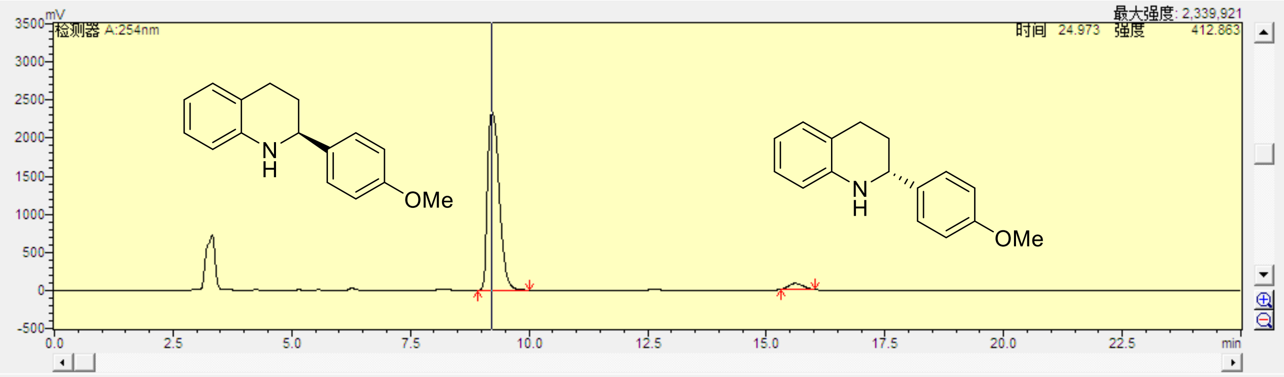

□ 化合物表视图

ID#	名称	保留时间	峰#	面积	高度	面积%
1	RT9.218	9.218	1	40114723	2336032	95.9625
2	RT15.607	15.607	2	1687786	79513	4.0375

Translation of all characters (Chinese) in the above two frameworks to English is as follows:

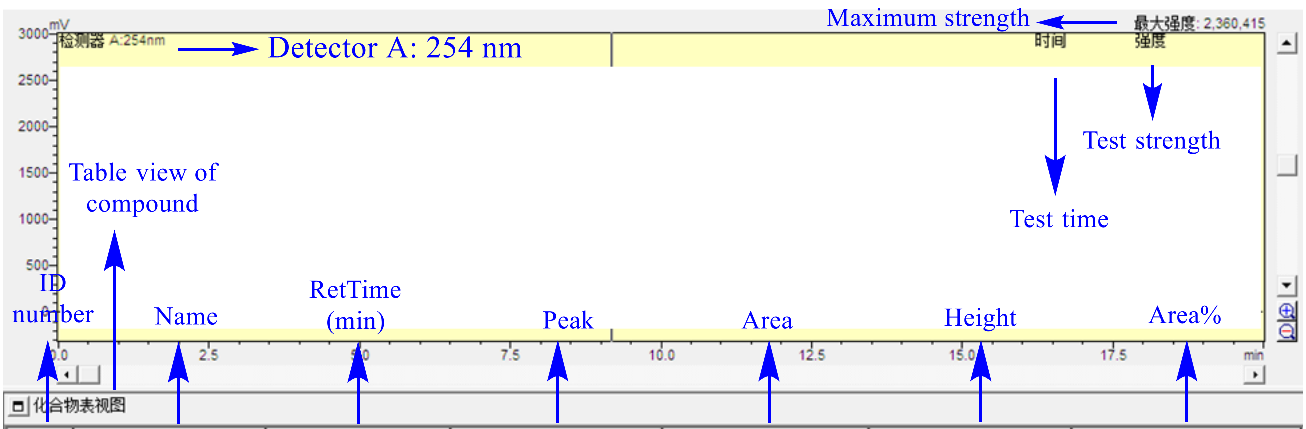

□ 化合物表视图

ID#	名称	保留时间	峰#	面积	高度	面积%
1	RT9.190	9.190	1	39625295	2360461	47.0509
2	RT11.859	11.859	2	44592689	2144002	52.9491

(*S*)-3j: (S)-2-(4-(methylthio)phenyl)-1,2,3,4-tetrahydroquinoline.: (HPLC: Chiracel OD-H, detected at 254 nm, eluent: n-hexane/2-propanol = 90/10, flow rate = 1.0mL/min, 25 ℃).

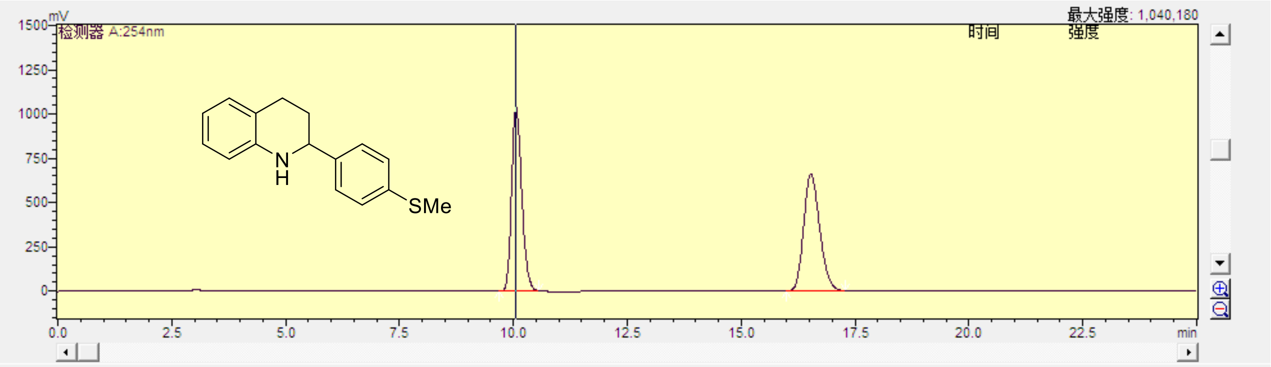

化合物表视图

ID#	名称	保留时间	峰#	面积	高度	面积%
1	RT10.056	10.056	1	16081924	1039121	49.8381
2	RT16.526	16.526	2	16186419	658779	50.1619

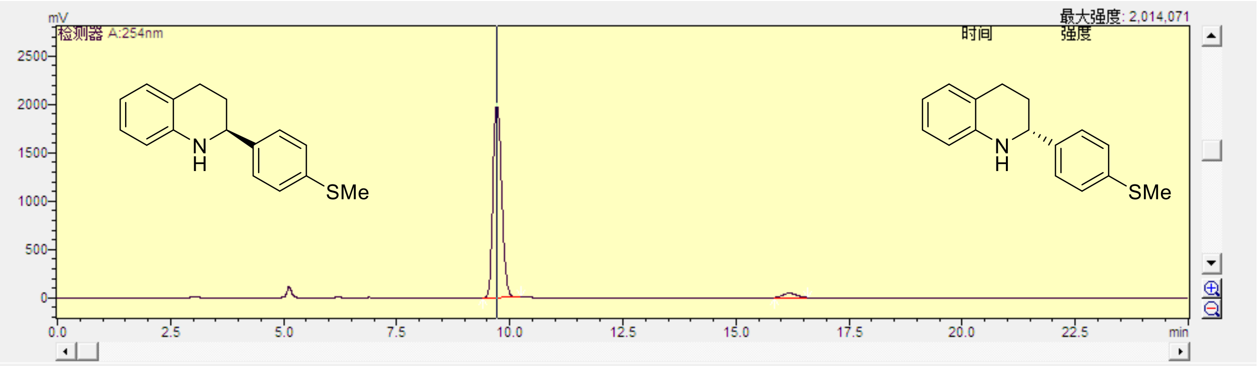

化合物表视图

ID#	名称	保留时间	峰#	面积	高度	面积%
1	RT9.710	9.710	1	26787727	2009737	96.5955
2	RT16.183	16.183	2	944125	44081	3.4045

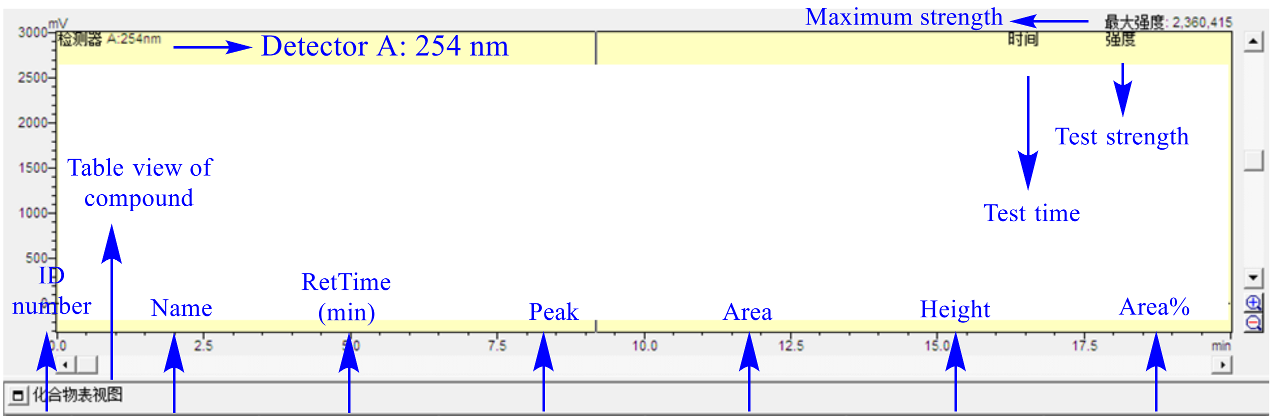

化合物表视图

ID#	名称	保留时间	峰#	面积	高度	面积%
1	RT9.190	9.190	1	39625295	2360461	47.0509
2	RT11.859	11.859	2	44592689	2144002	52.9491

(*S*)-3k: (S)-2-(4-(trimethylsilyl)phenyl)-1,2,3,4-tetrahydroquinoline.: (HPLC: Chiracel OD-H, detected at 254 nm, eluent: n-hexane/2-propanol = 90/10, flow rate = 1.0mL/min, 25 ℃).

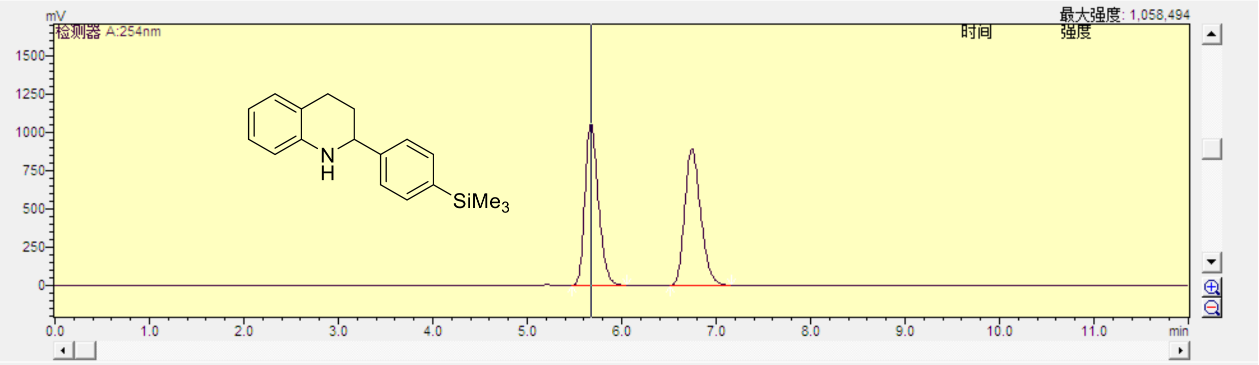

化合物表视图

ID#	名称	保留时间	峰#	面积	高度	面积%
1	RT5.670	5.670	1	10447843	1057592	49.9166
2	RT6.738	6.738	2	10482758	891578	50.0834

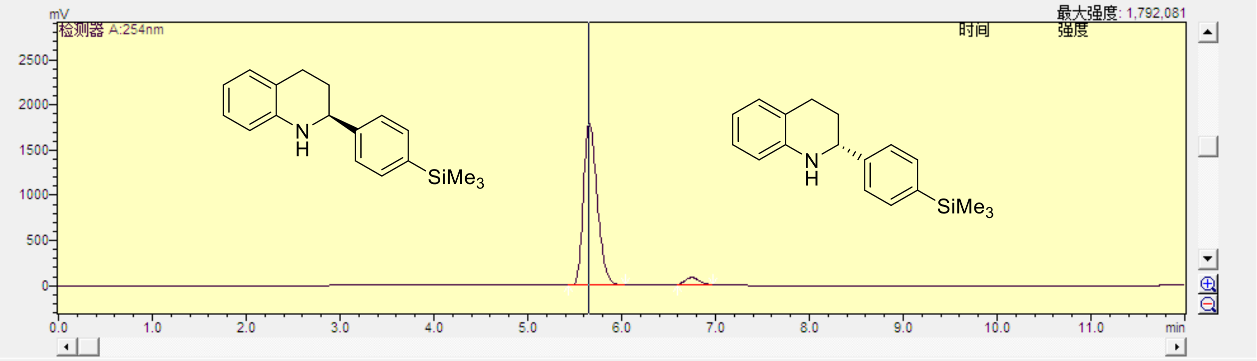

化合物表视图

ID#	名称	保留时间	峰#	面积	高度	面积%
1	RT5.653	5.653	1	18407935	1788702	95.5358
2	RT6.742	6.742	2	860160	80960	4.4642

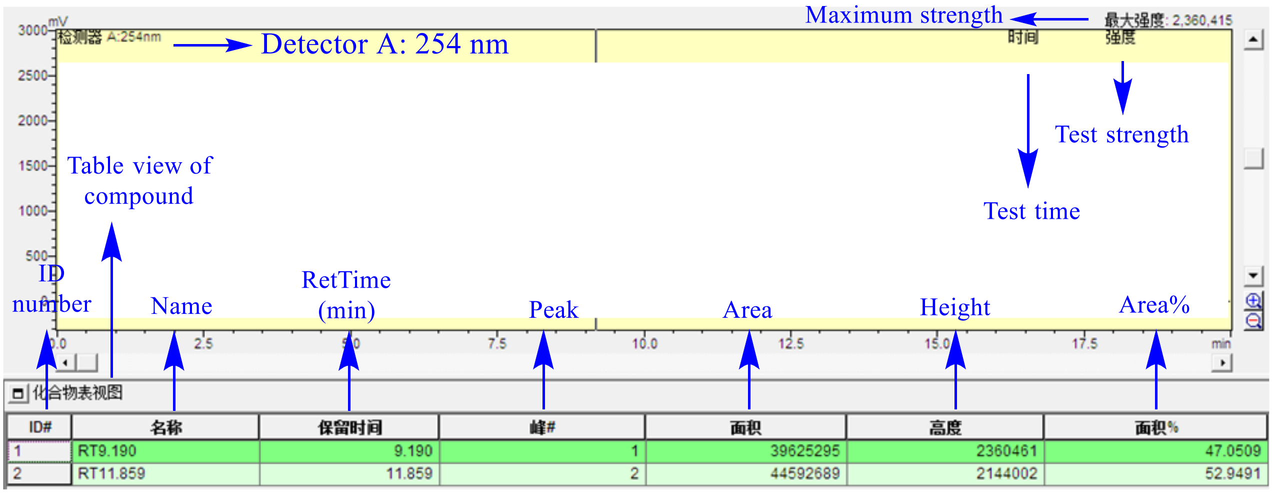

化合物表视图

ID#	名称	保留时间	峰#	面积	高度	面积%
1	RT9.190	9.190	1	39625295	2360461	47.0509
2	RT11.859	11.859	2	44592689	2144002	52.9491

(*S*)-3l: (S)-2-([1,1'-biphenyl]-4-yl)-1,2,3,4-tetrahydroquinoline: (HPLC: Chiracel OD-H, detected at 254 nm, eluent: n-hexane/2-propanol = 90/10, flow rate = 1mL/min, 25 ℃).

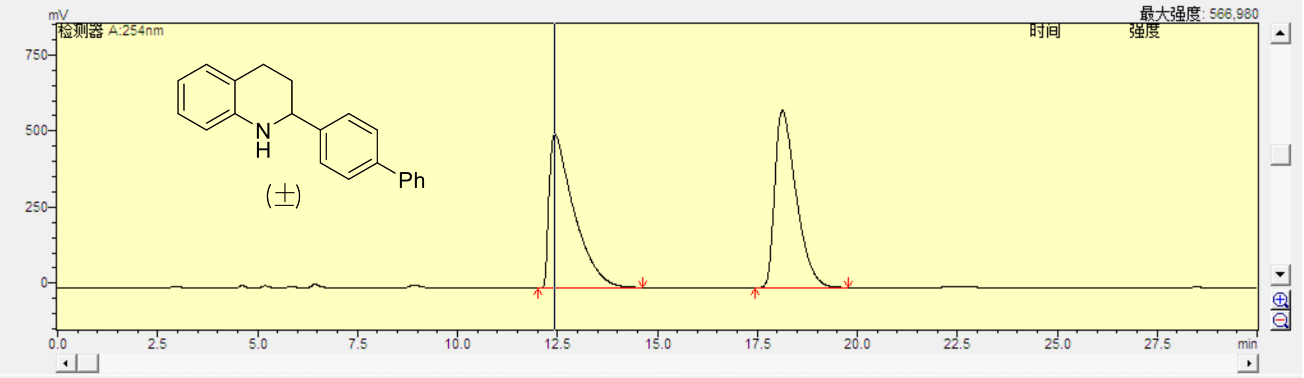

化合物表视图

ID#	名称	保留时间	峰#	面积	高度	面积%
1	RT12.434	12.434	1	21718532	504085	50.0504
2	RT18.125	18.125	2	21674799	581184	49.9496

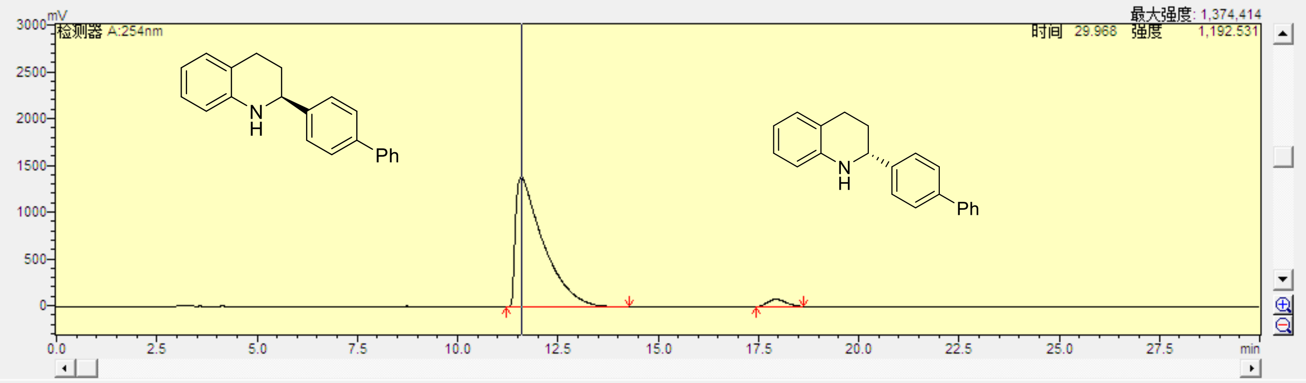

化合物表视图

ID#	名称	保留时间	峰#	面积	高度	面积%
1	RT11.585	11.585	1	66741422	1387207	96.3509
2	RT17.933	17.933	2	2527672	78766	3.6491

<u>Translation of all characters (Chinese) in the above two frameworks to English is as follows:</u>

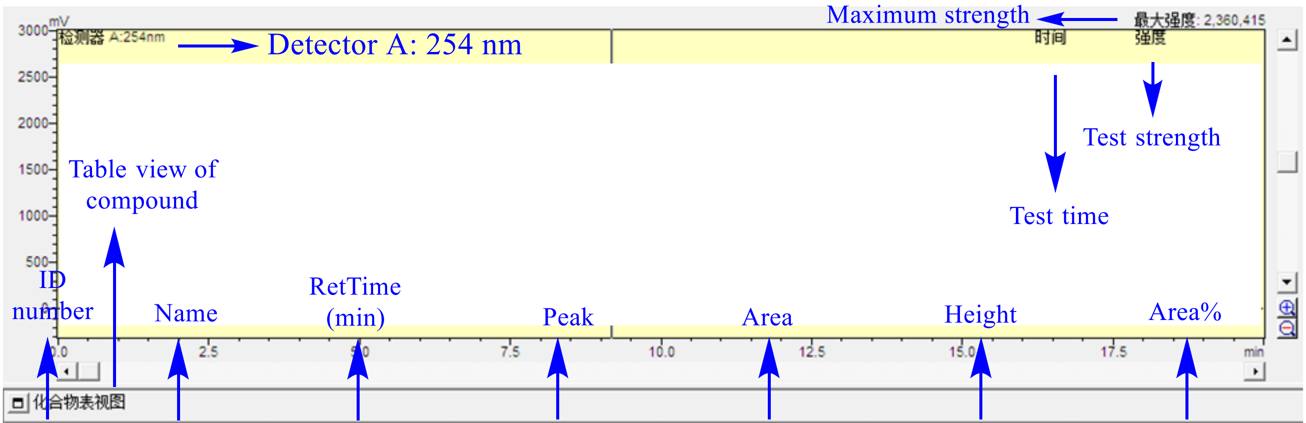

ID#	名称	保留时间	峰#	面积	高度	面积%
1	RT9.190	9.190	1	39625295	2360461	47.0509
2	RT11.859	11.859	2	44592689	2144002	52.9491

(*S*)-3m: (S)-2-(thiophen-2-yl)-1,2,3,4-tetrahydroquinoline: (HPLC: Chiracel OD-H, detected at 254 nm, eluent: n-hexane/2-propanol = 90/10, flow rate = 1mL/min, 25 ℃).

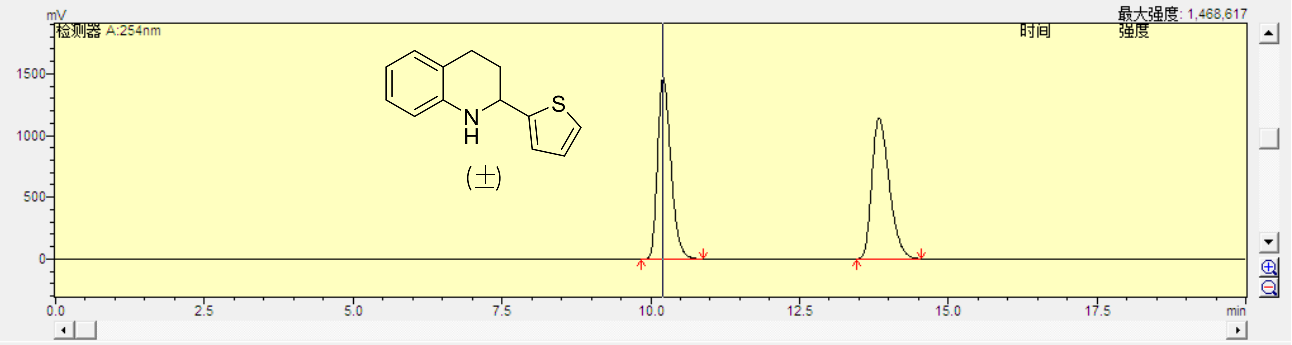

化合物表视图

ID#	名称	保留时间	峰#	面积	高度	面积%
1	RT10.207	10.207	1	22999065	1470004	49.6323
2	RT13.833	13.833	2	23331671	1143757	50.3500

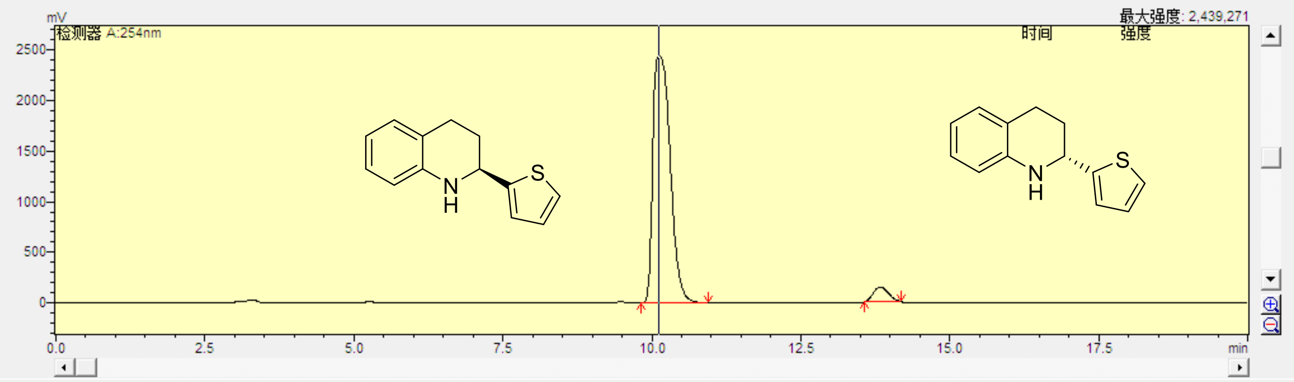

化合物表视图

ID#	名称	保留时间	峰#	面积	高度	面积%
1	RT10.124	10.124	1	49645320	2439358	95.0869
2	RT13.832	13.832	2	2565130	146895	4.9131

Translation of all characters (Chinese) in the above two frameworks to English is as follows:

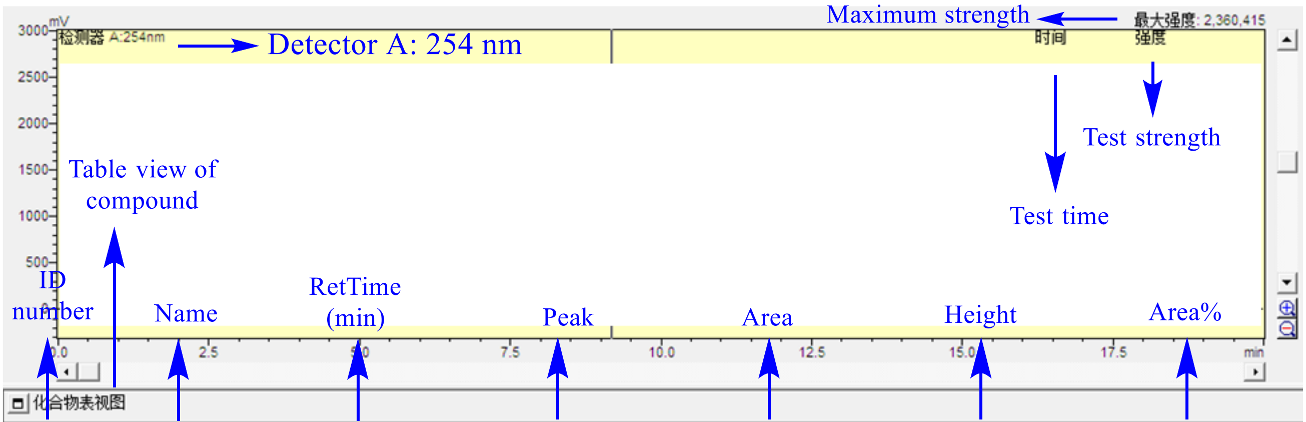

ID#	名称	保留时间	峰#	面积	高度	面积%
1	RT9.190	9.190	1	39625295	2360461	47.0509
2	RT11.859	11.859	2	44592689	2144002	52.9491

(*S*)-3n: (S)-6-fluoro-2-phenyl-1,2,3,4-tetrahydroquinoline.: (HPLC: Chiracel OD-H, detected at 254 nm, eluent: n-hexane/2-propanol = 90/10, flow rate = 1.0mL/min, 25 ℃).

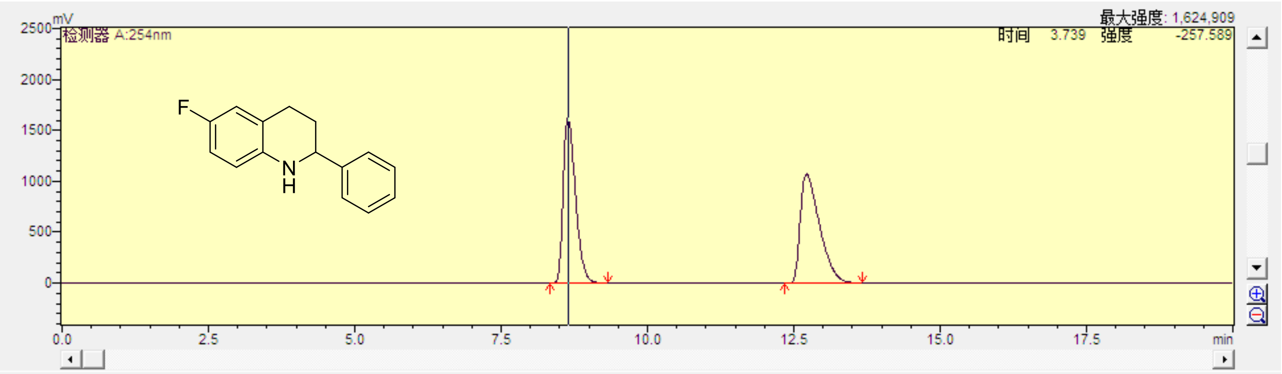

化合物表视图

ID#	名称	保留时间	峰#	面积	高度	面积%
1	RT8.638	8.638	1	23840889	1625642	49.2654
2	RT12.714	12.714	2	24551892	1071054	50.7346

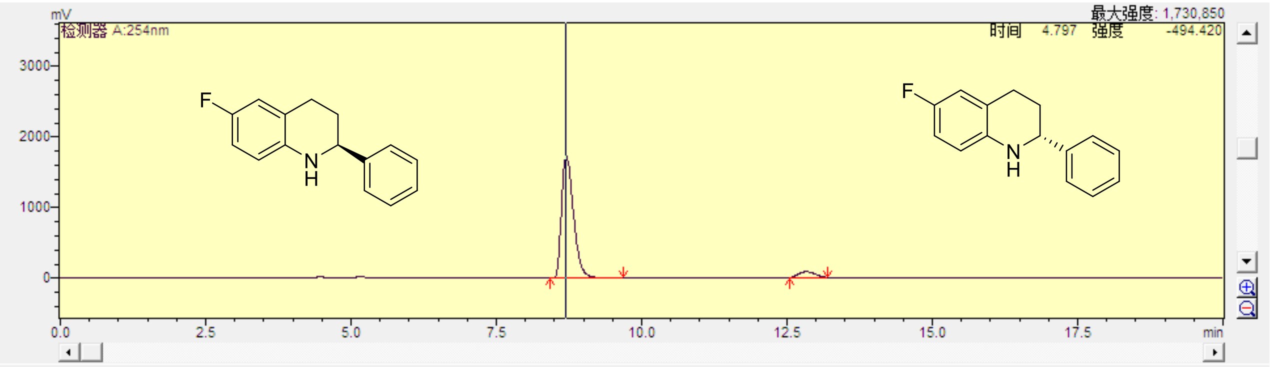

化合物表视图

ID#	名称	保留时间	峰#	面积	高度	面积%
1	RT8.699	8.699	1	24930235	1730211	93.6247
2	RT12.825	12.825	2	1697605	83896	6.3753

<u>Translation of all characters (Chinese) in the above two frameworks to English is as follows:</u>

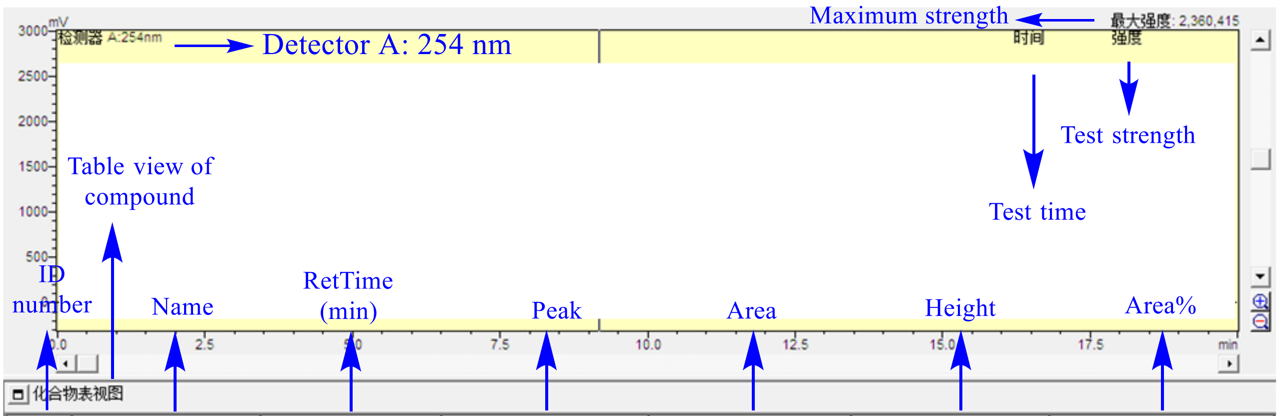

ID#	名称	保留时间	峰#	面积	高度	面积%
1	RT9.190	9.190	1	39625295	2360461	47.0509
2	RT11.859	11.859	2	44592689	2144002	52.9491

(*S*)-3o: (S)-6-bromo-2-phenyl-1,2,3,4-tetrahydroquinoline.: (HPLC: Chiracel OD-H, detected at 254 nm, eluent: n-hexane/2-propanol = 90/10, flow rate = 1.0mL/min, 25 ℃).

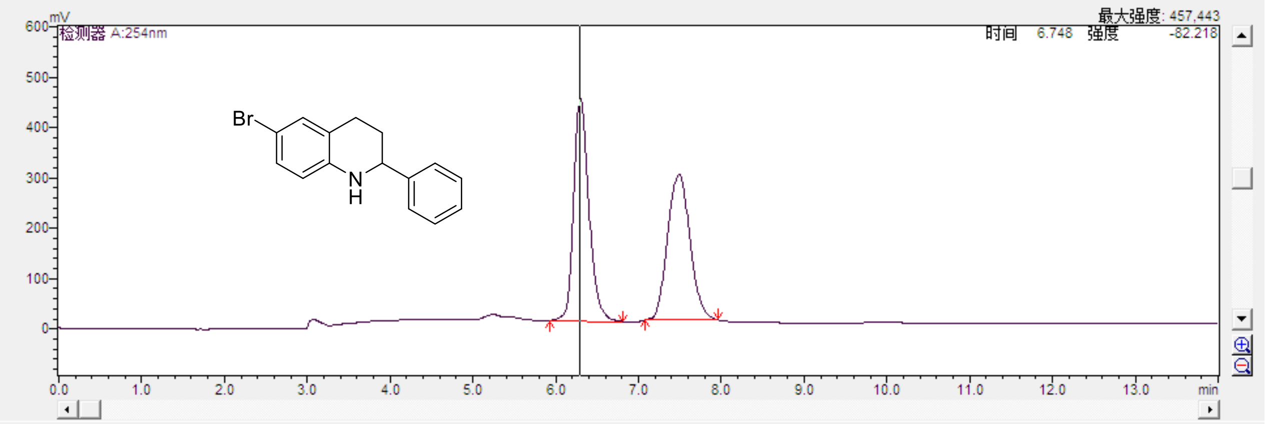

化合物表视图

ID#	名称	保留时间	峰#	面积	高度	面积%
1	RT6.295	6.295	1	5666579	441585	50.9246
2	RT7.487	7.487	2	5460801	289395	49.0754

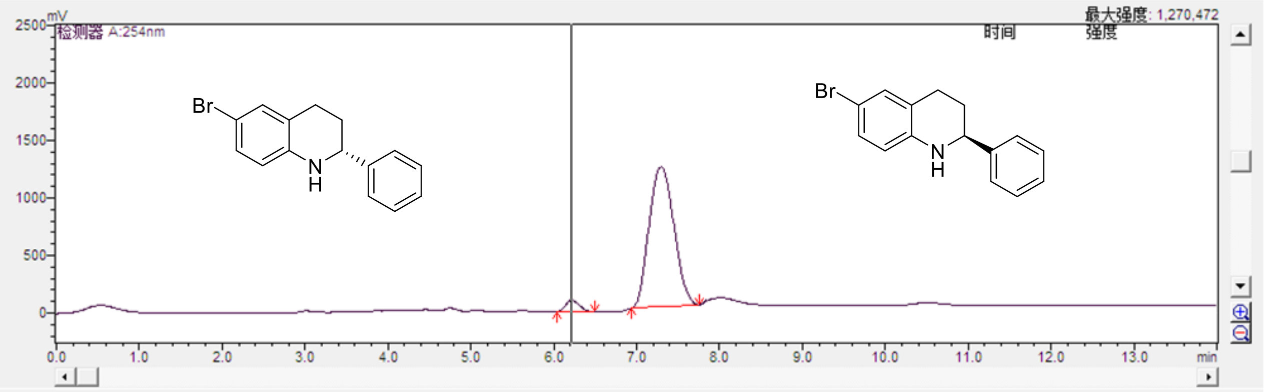

化合物表视图

ID#	名称	保留时间	峰#	面积	高度	面积%
1	RT6.212	6.212	1	1214048	103748	4.5626
2	RT7.292	7.292	2	25394423	1216021	95.4374

Translation of all characters (Chinese) in the above two frameworks to English is as follows:

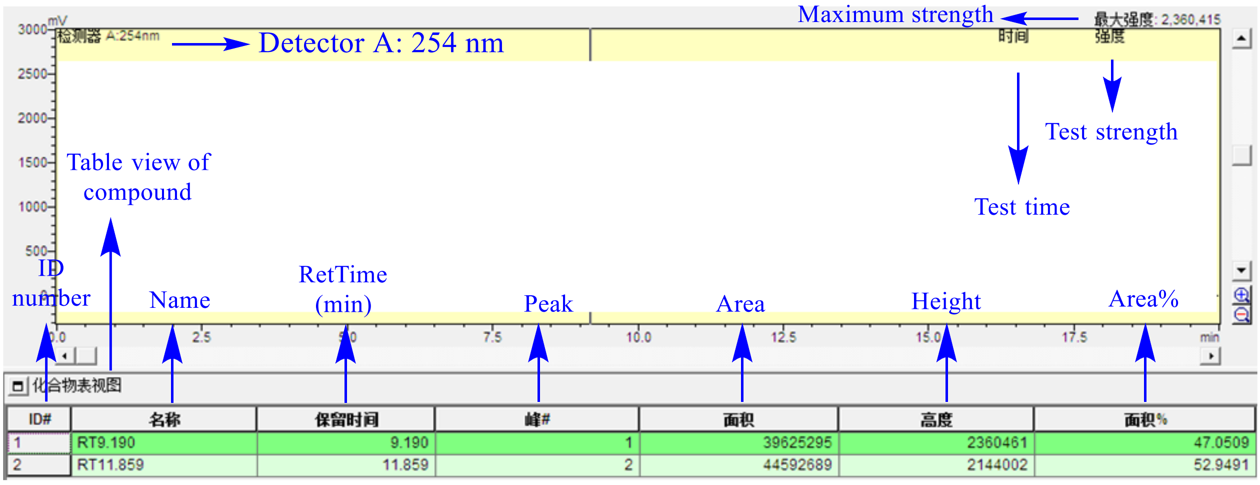

ID#	名称	保留时间	峰#	面积	高度	面积%
1	RT9.190	9.190	1	39625295	2360461	47.0509
2	RT11.859	11.859	2	44592689	2144002	52.9491

(*S*)-3p: (S)-6-methyl-2-phenyl-1,2,3,4-tetrahydroquinoline.: (HPLC: Chiracel OD-H, detected at 254 nm, eluent: n-hexane/2-propanol = 90/10, flow rate = 1.0mL/min, 25 ℃).

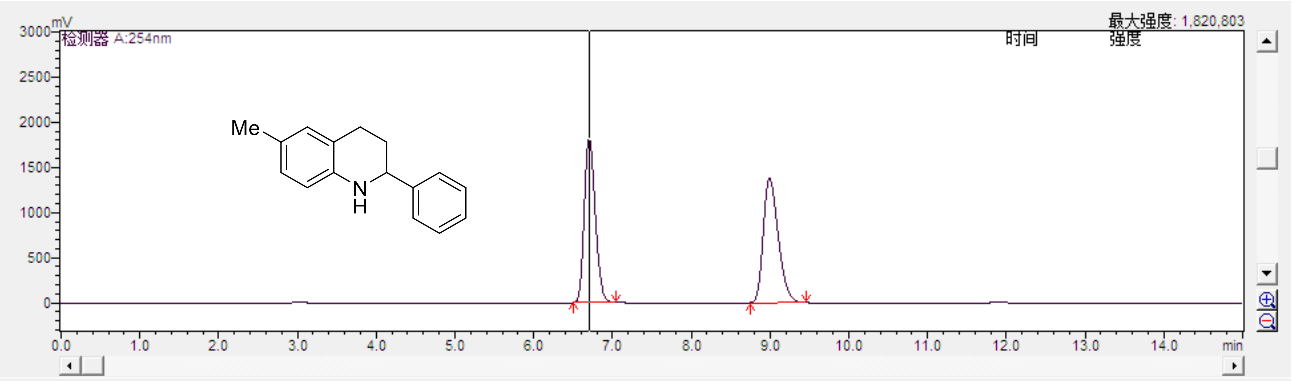

化合物表视图

ID#	名称	保留时间	峰#	面积	高度	面积%
1	RT6.698	6.698	1	17546716	1817844	49.3413
2	RT8.990	8.990	2	18015190	1373378	50.6587

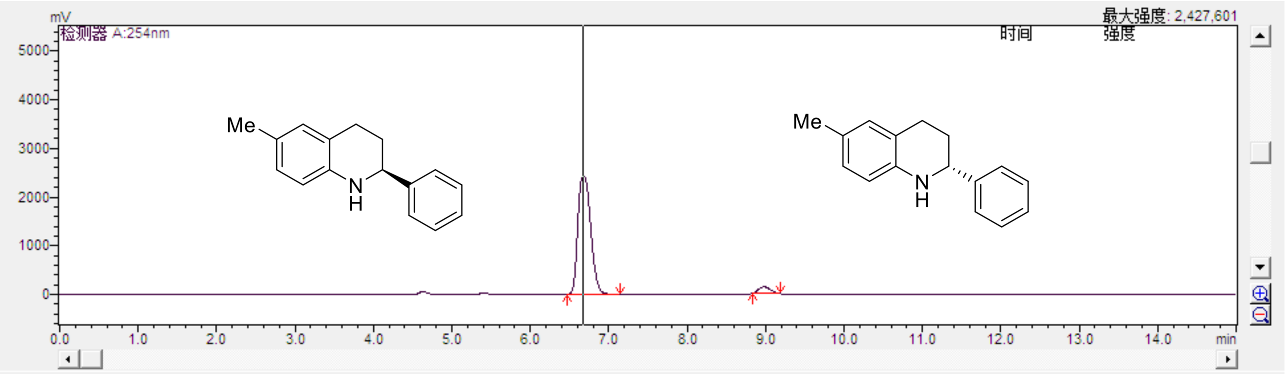

化合物表视图

ID#	名称	保留时间	峰#	面积	高度	面积%
1	RT6.674	6.674	1	27393811	2424607	94.9441
2	RT8.976	8.976	2	1458768	136599	5.0559

<u>Translation of all characters (Chinese) in the above two frameworks to English is as follows:</u>

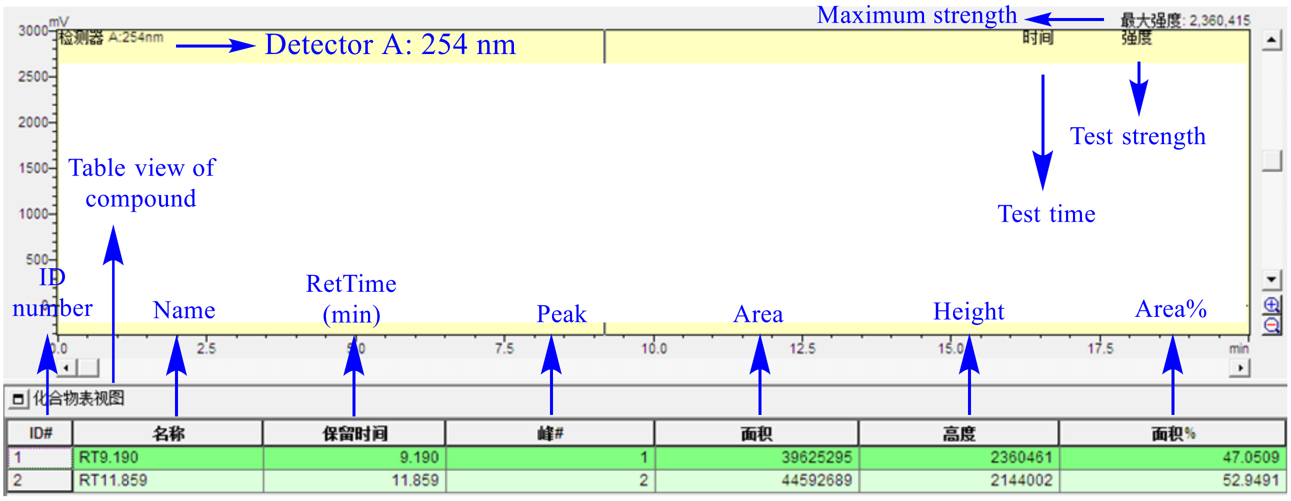

ID#	名称	保留时间	峰#	面积	高度	面积%
1	RT9.190	9.190	1	39625295	2360461	47.0509
2	RT11.859	11.859	2	44592689	2144002	52.9491

(*S*)-3q: (S)-6-methoxy-2-phenyl-1,2,3,4-tetrahydroquinoline.: (HPLC: Chiracel OD-H, detected at 254 nm, eluent: n-hexane/2-propanol = 90/10, flow rate = 1.0mL/min, 25 ℃).

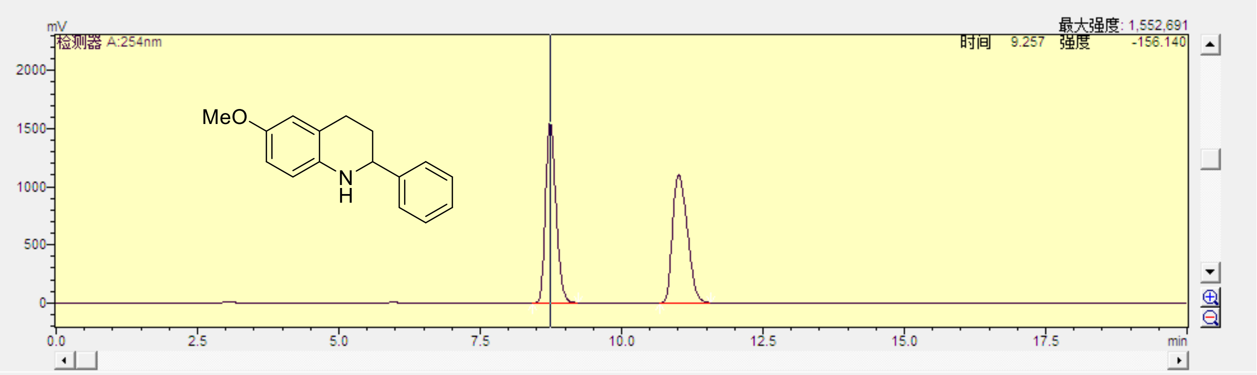

化合物表视图

ID#	名称	保留时间	峰#	面积	高度	面积%
1	RT8.732	8.732	1	20048620	1552324	49.8493
2	RT11.005	11.005	2	20169823	1100662	50.1507

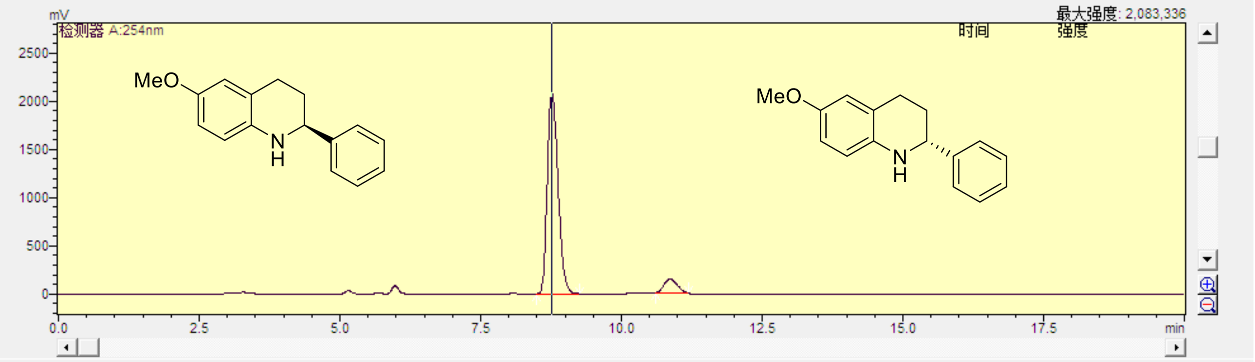

化合物表视图

ID#	名称	保留时间	峰#	面积	高度	面积%
1	RT8.762	8.762	1	27823837	2080359	91.8935
2	RT10.862	10.862	2	2454526	145817	8.1065

<u>Translation of all characters (Chinese) in the above two frameworks to English is as follows:</u>

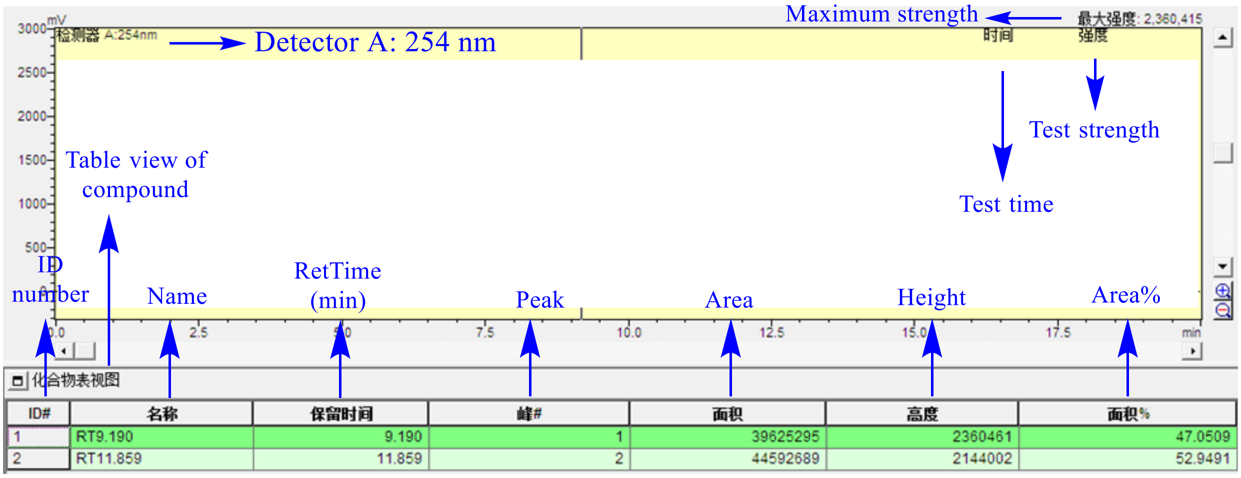

化合物表视图

ID#	名称	保留时间	峰#	面积	高度	面积%
1	RT9.190	9.190	1	39625295	2360461	47.0509
2	RT11.859	11.859	2	44592689	2144002	52.9491

(*S*)-3r: methyl (S)-4-(6-methoxy-1,2,3,4-tetrahydroquinolin-2-yl)benzoate.: (HPLC: Chiracel OD-H, detected at 254 nm, eluent: n-hexane/2-propanol = 90/10, flow rate = 1.0mL/min, 25 ℃).

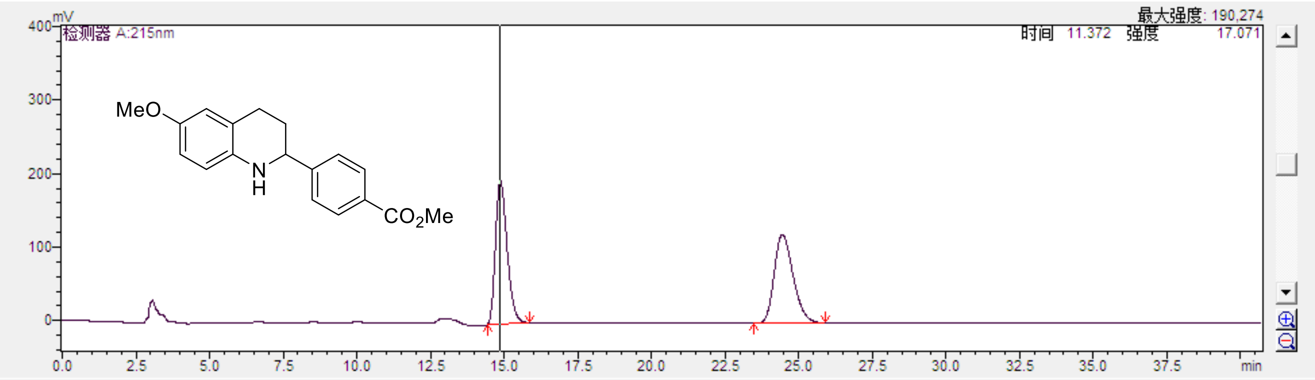

化合物表视图

ID#	名称	保留时间	峰#	面积	高度	面积%
1	RT14.874	14.874	1	5347358	194826	49.7455
2	RT24.444	24.444	2	5402079	120800	50.2545

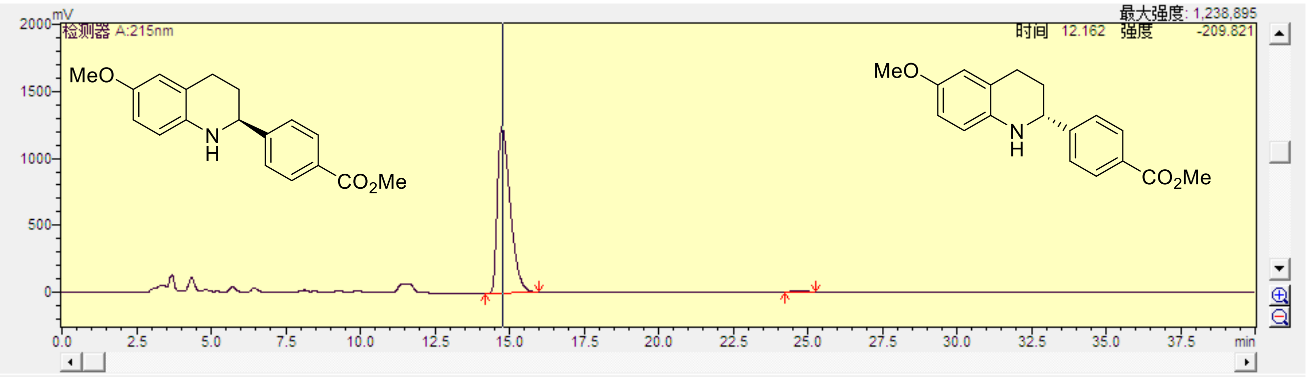

化合物表视图

ID#	名称	保留时间	峰#	面积	高度	面积%
1	RT14.752	14.752	1	37350783	1245582	99.2543
2	RT24.723	24.723	2	280624	8157	0.7457

<u>Translation of all characters (Chinese) in the above two frameworks to English is as follows:</u>

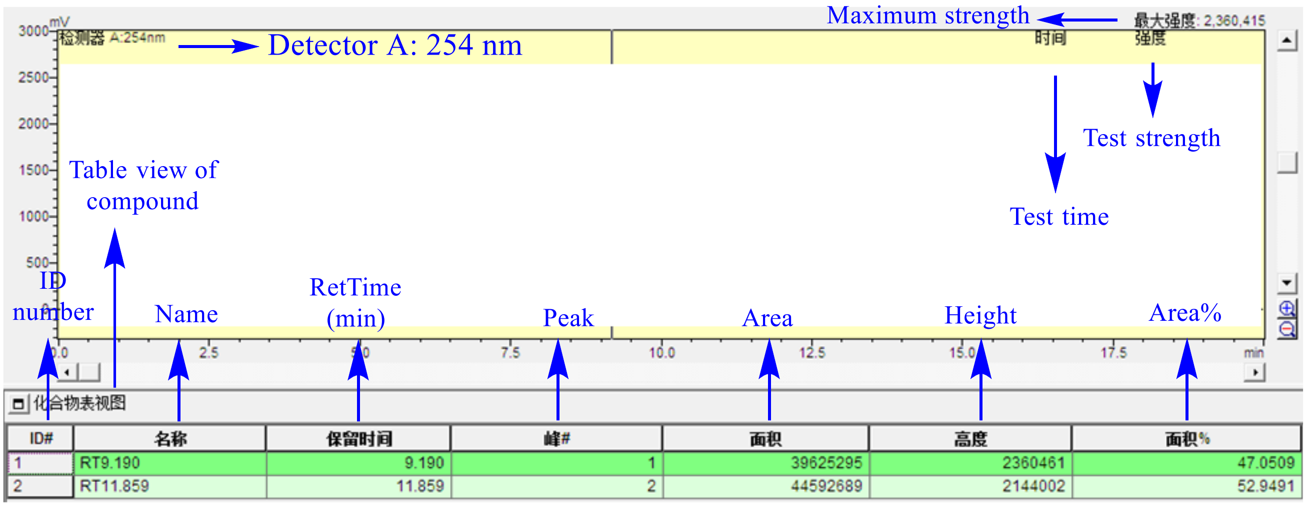

化合物表视图

ID#	名称	保留时间	峰#	面积	高度	面积%
1	RT9.190	9.190	1	39625295	2360461	47.0509
2	RT11.859	11.859	2	44592689	2144002	52.9491

Figure S5. Characterization of chiral products (The ^{1}H NMR and ^{13}C NMR spectra of all chiral products).

(*R*)-1a: (R)-3,3-dimethyl-2-phenylindoline.

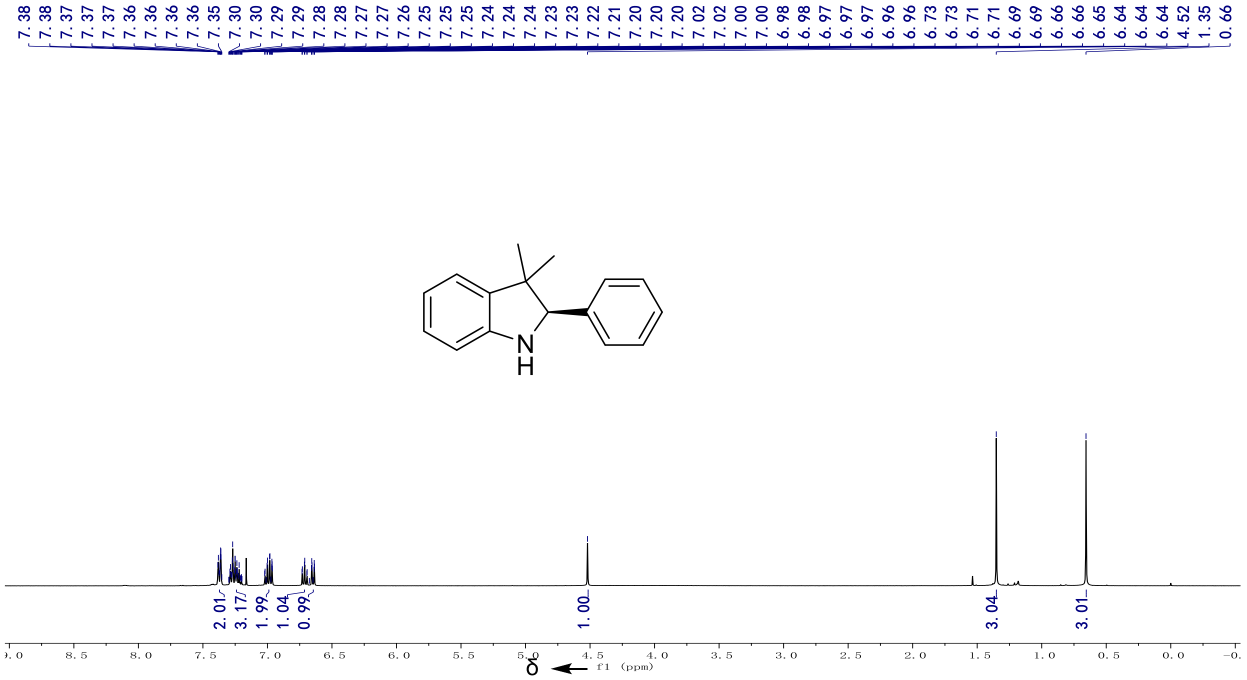

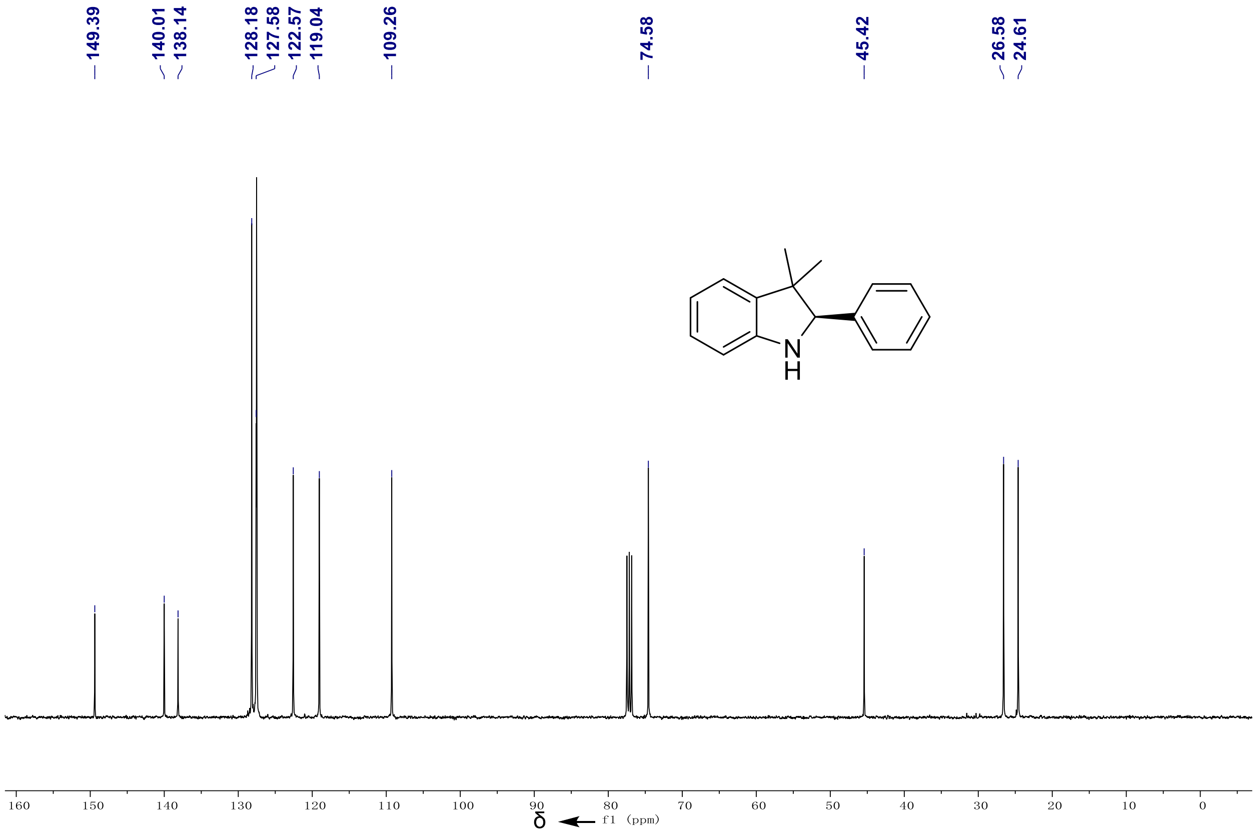

(*R*)-1b: (R)- 5-fluoro-3,3-dimethyl-2-phenylindoline.

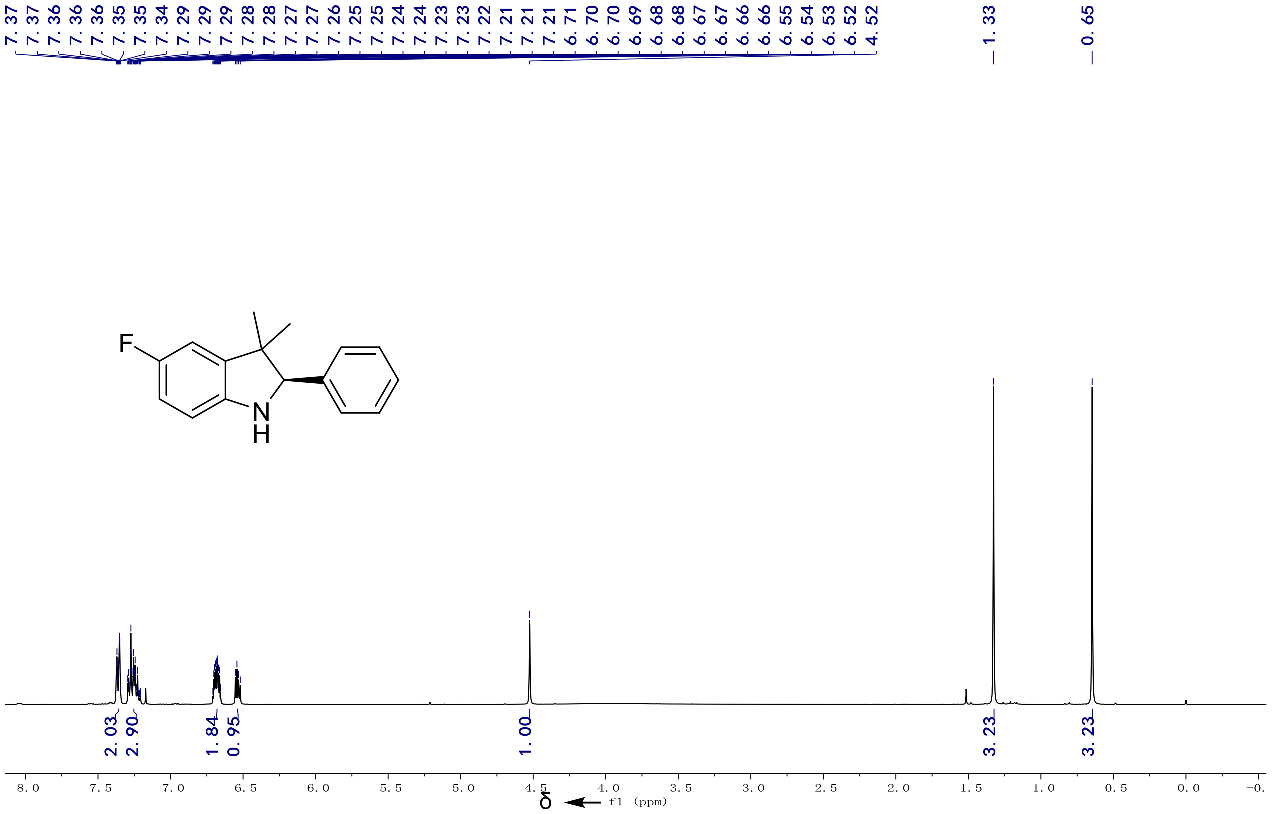

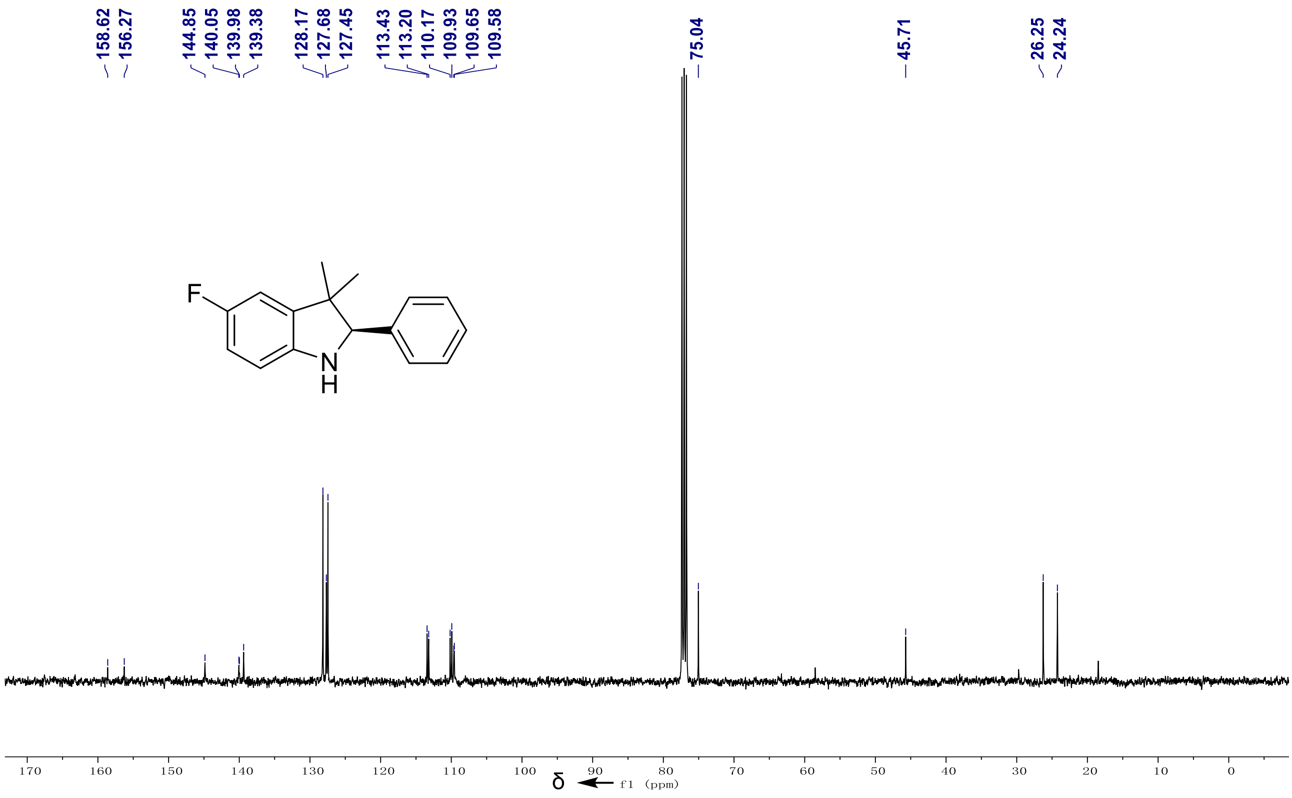

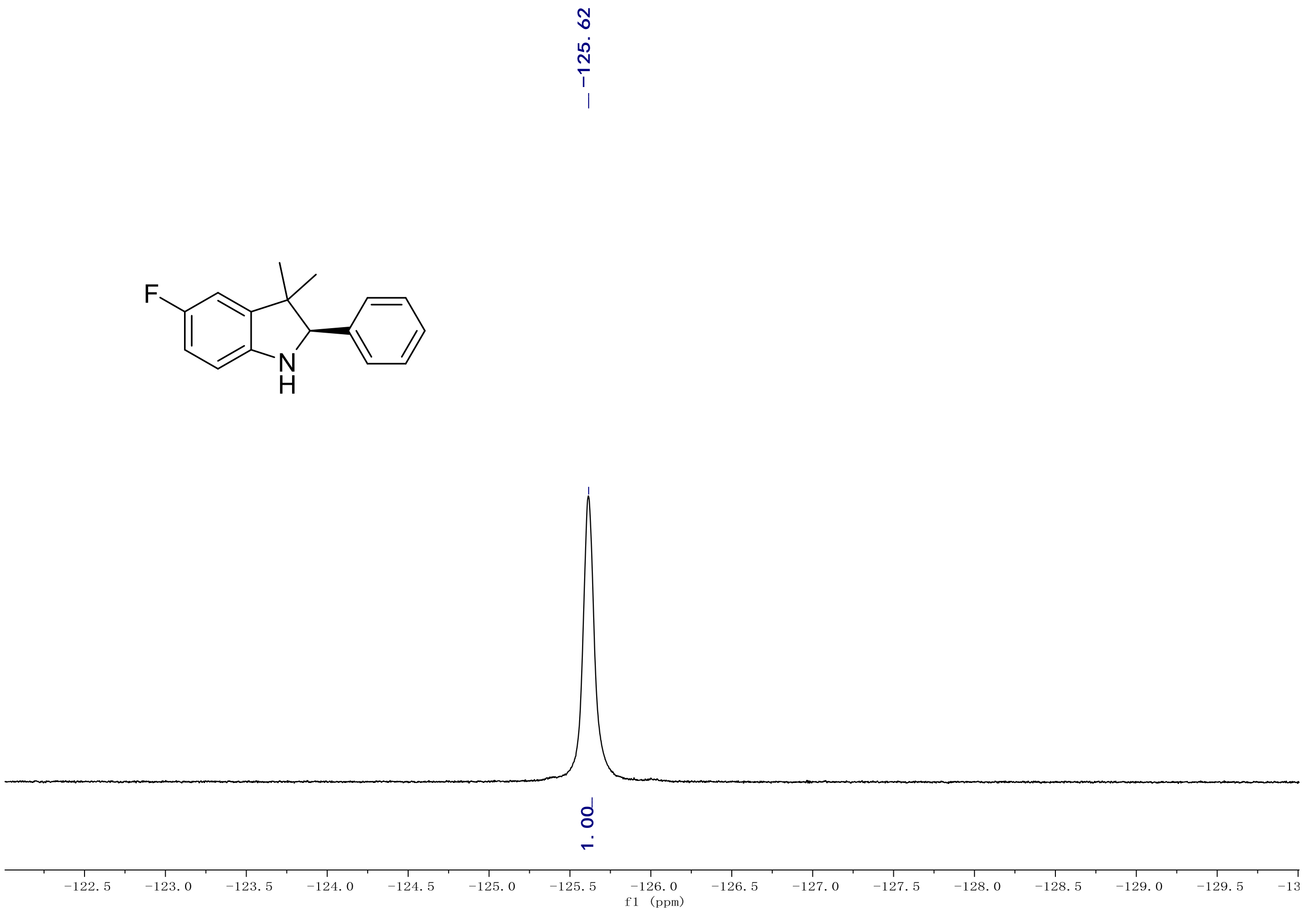

-125.62
F
N
H
1.00
-122.5 -123.0 -123.5 -124.0 -124.5 -125.0 -125.5 -126.0 -126.5 -127.0 -127.5 -128.0 -128.5 -129.0 -129.5 -13
f1 (ppm)

(*R*)-1c：(R)-4-chloro-3,3-dimethyl-2-phenylindoline

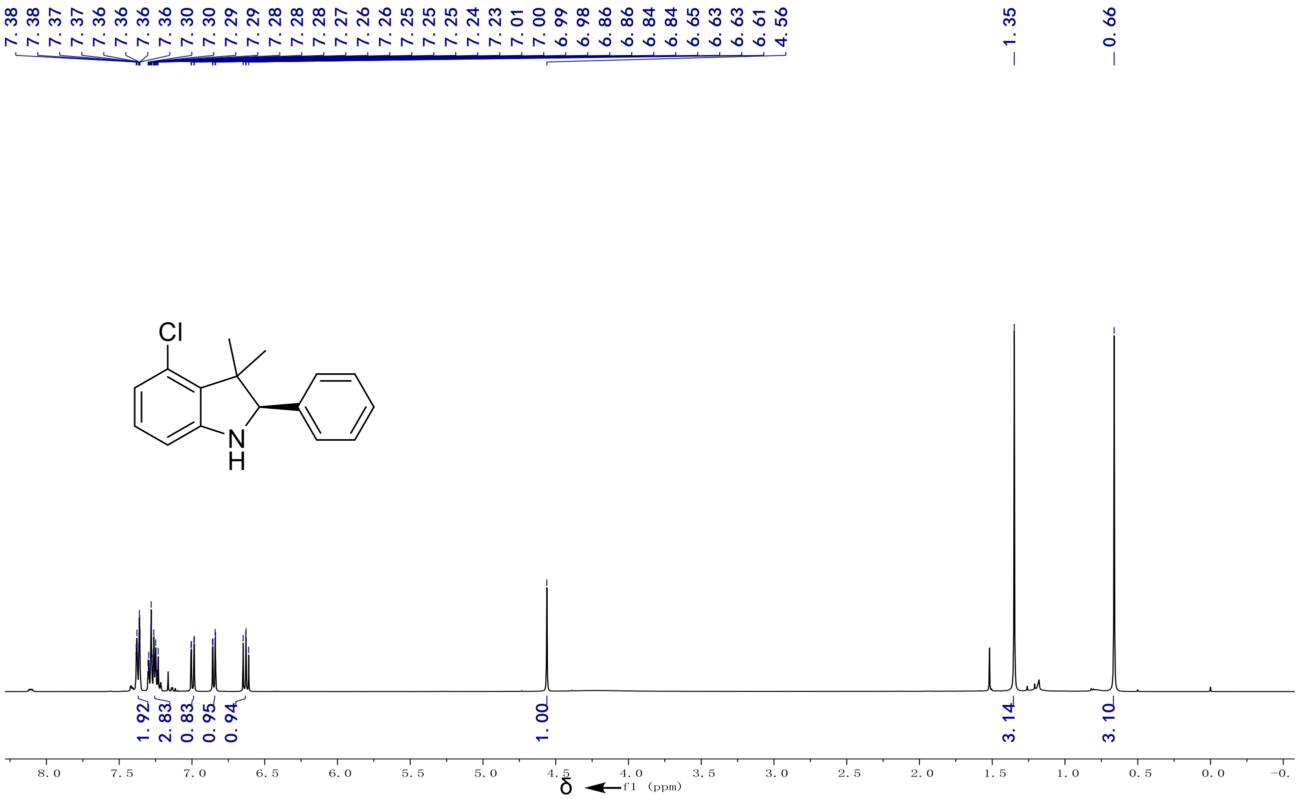

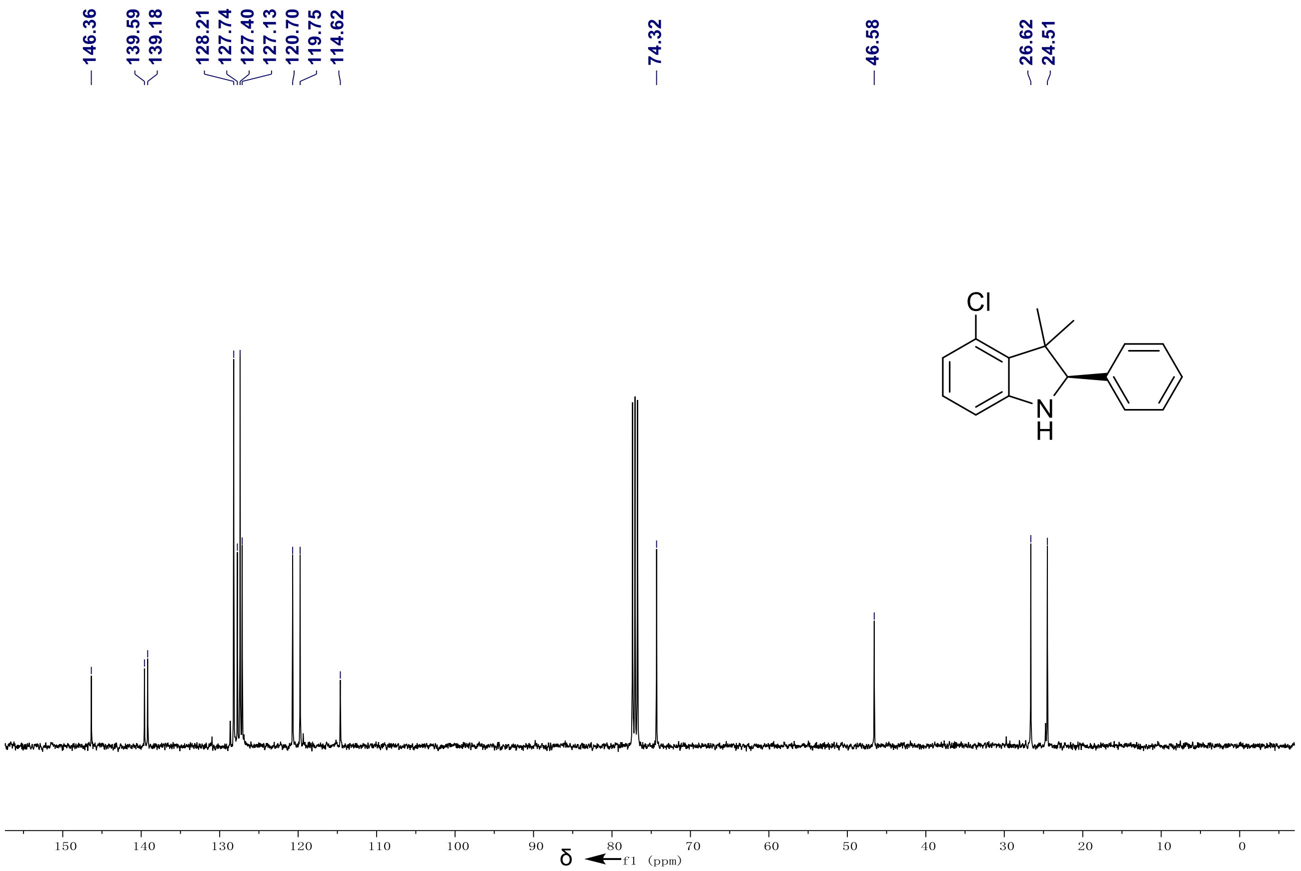

(*R*)-1d: (R)-5-chloro-3,3-dimethyl-2-phenylindoline.

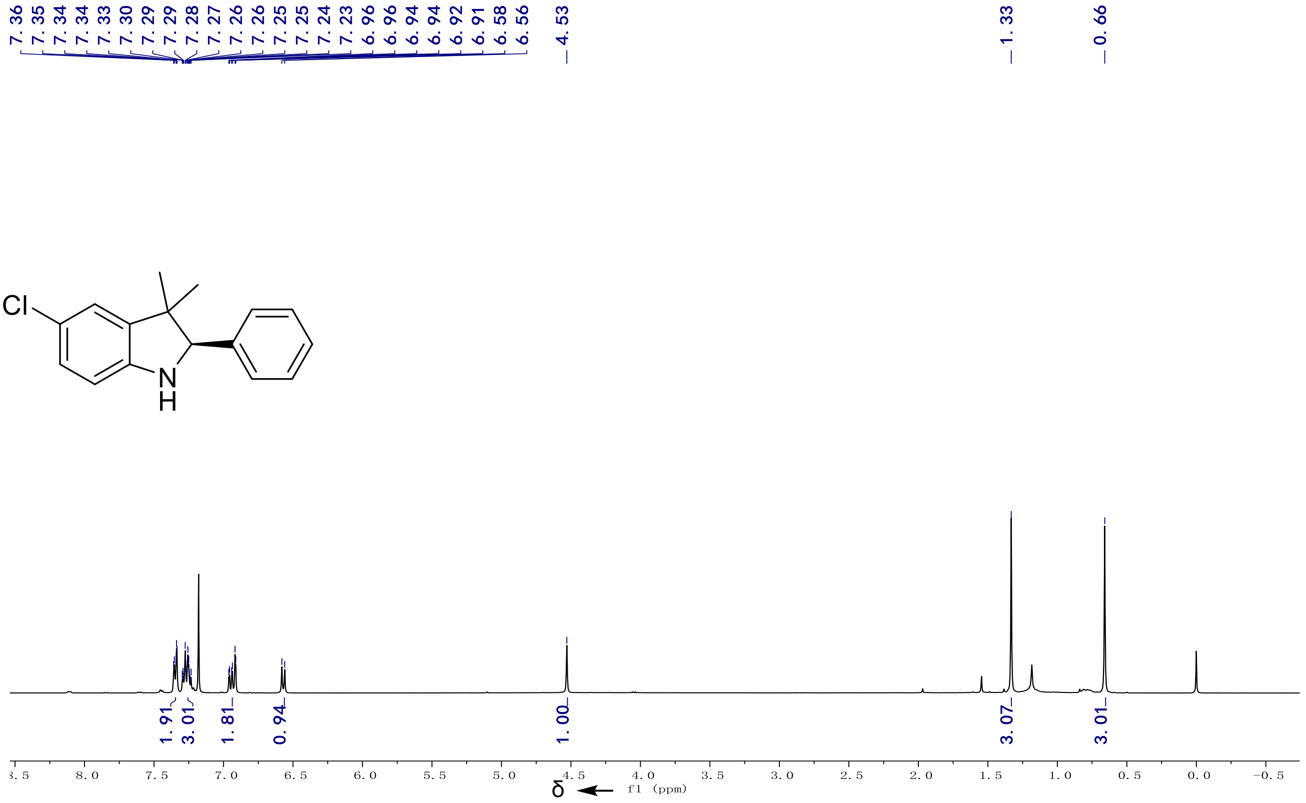

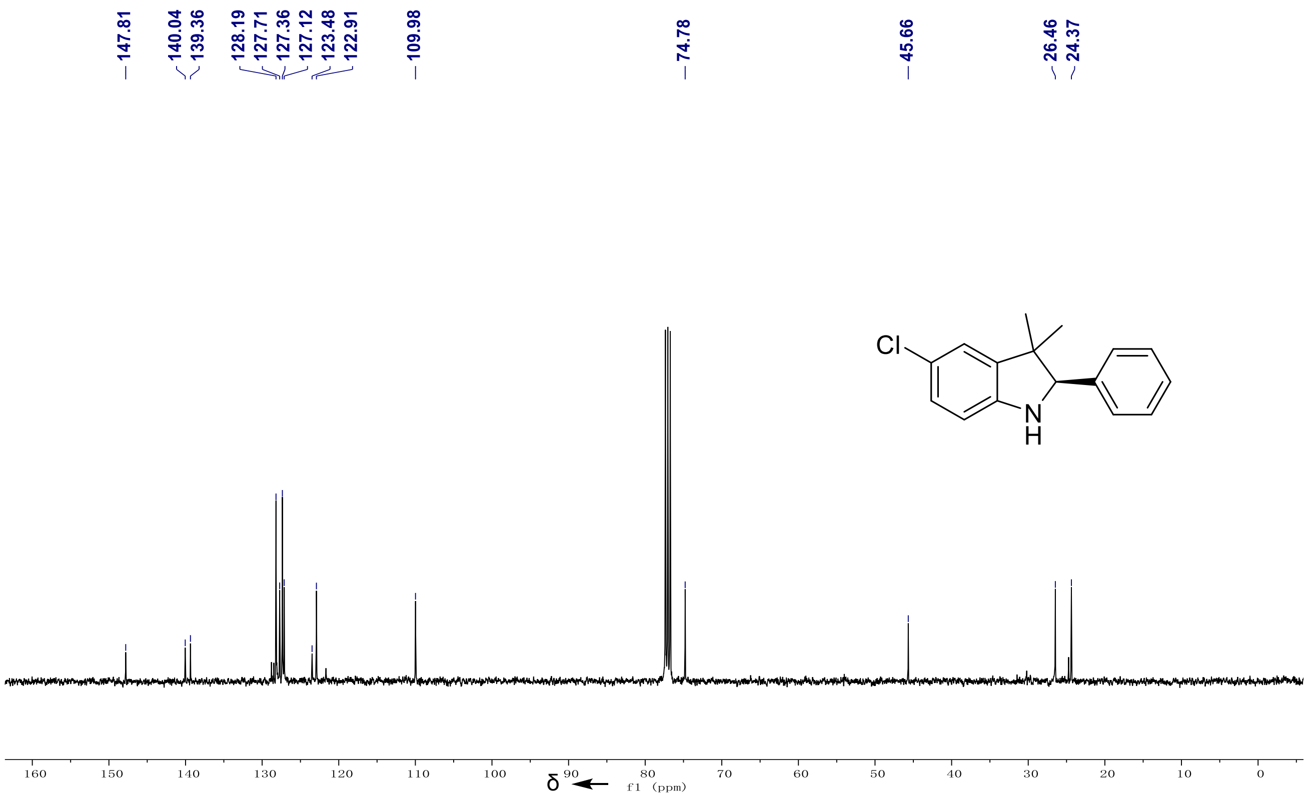

(*R*)-1e: (R)-5-bromo-3,3-dimethyl-2-phenylindolin.

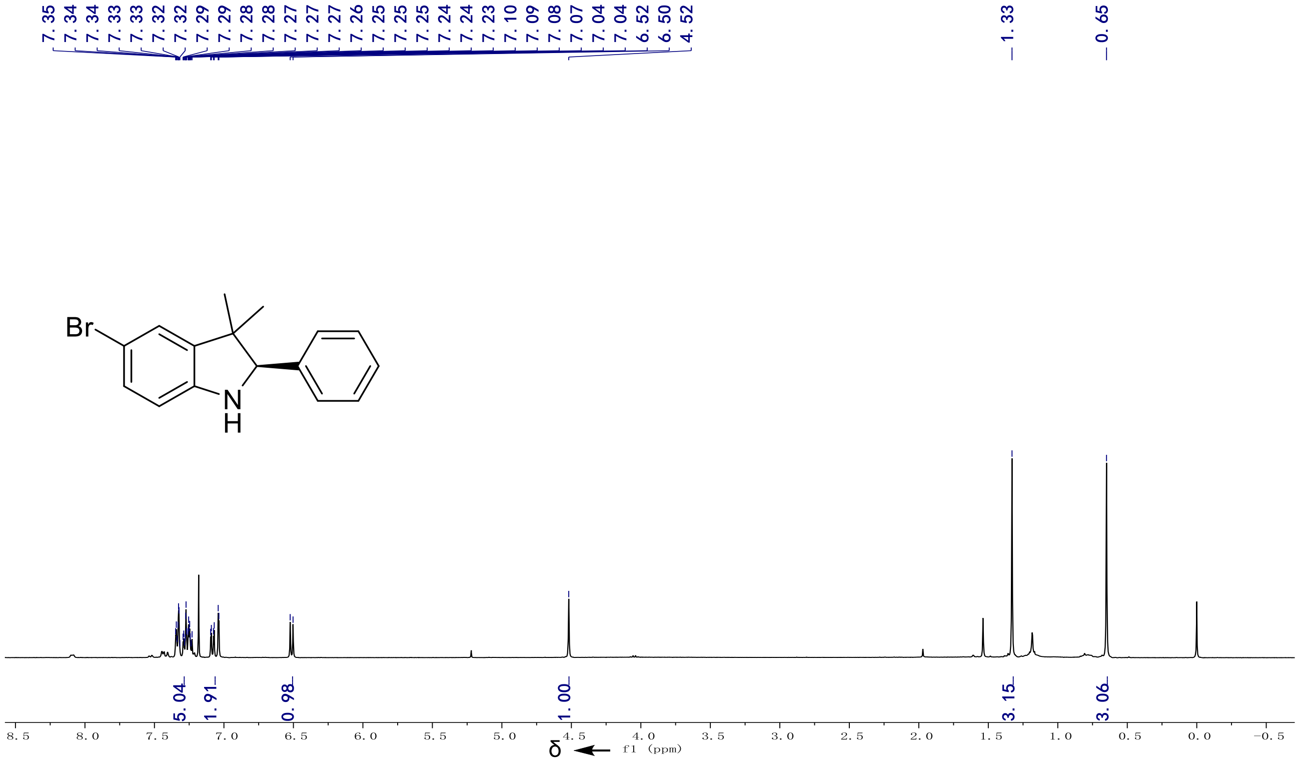

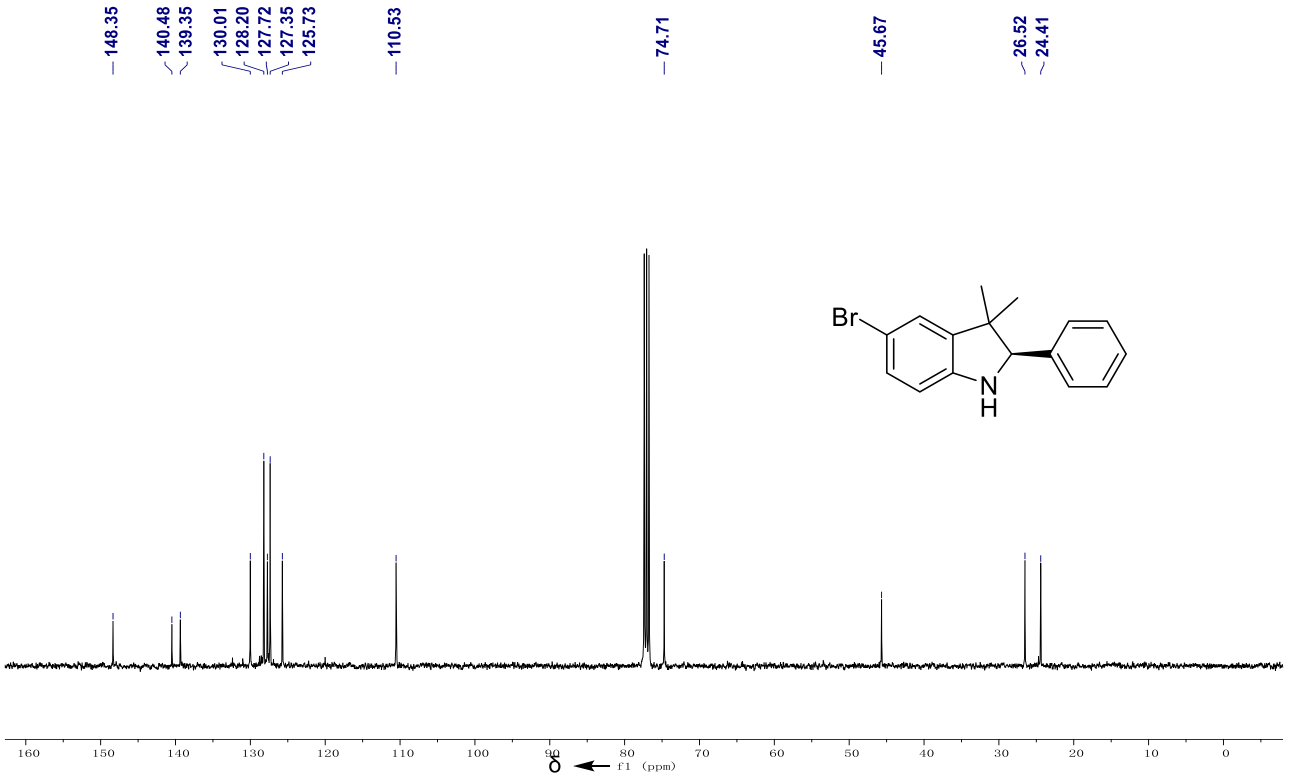

(*R*)-1f: (R)-6-broo-3,3-dimethyl-2-phenylindoline.

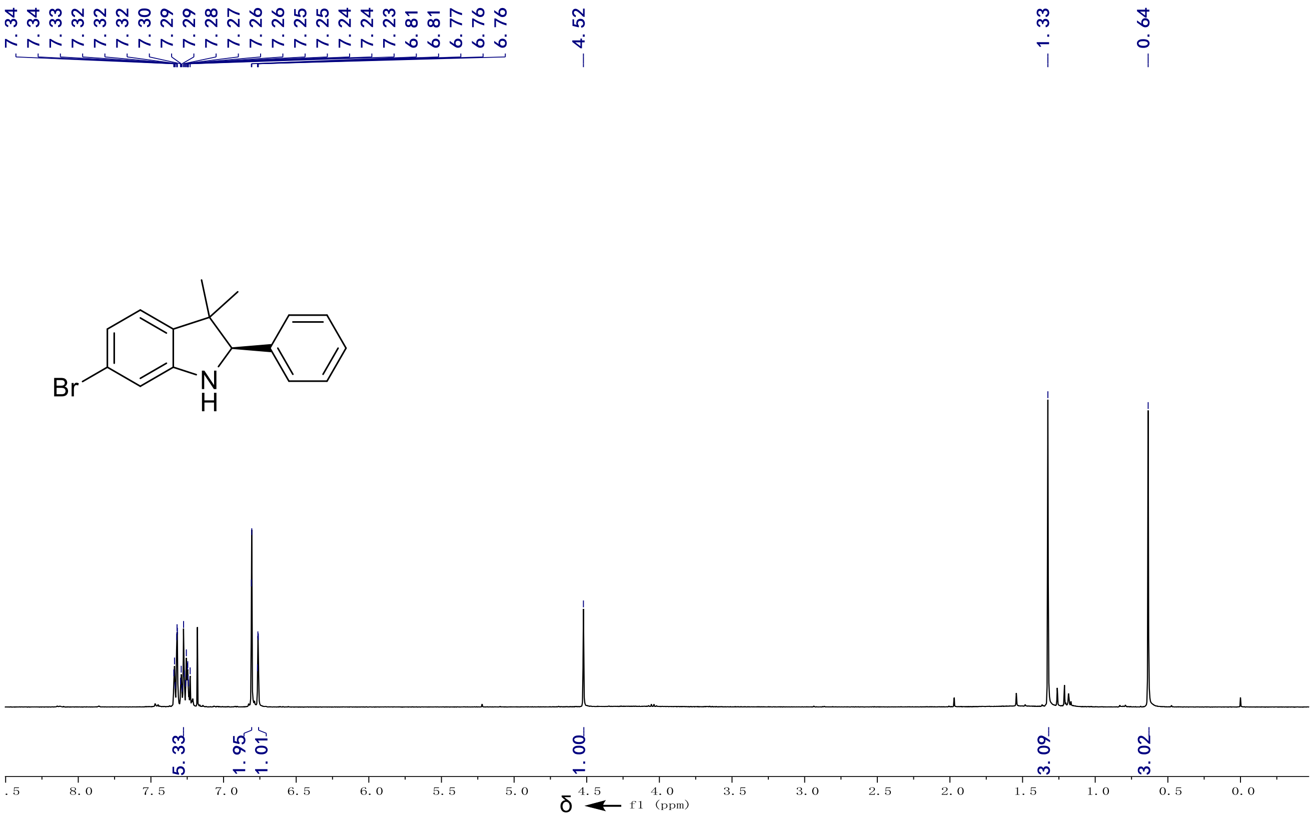

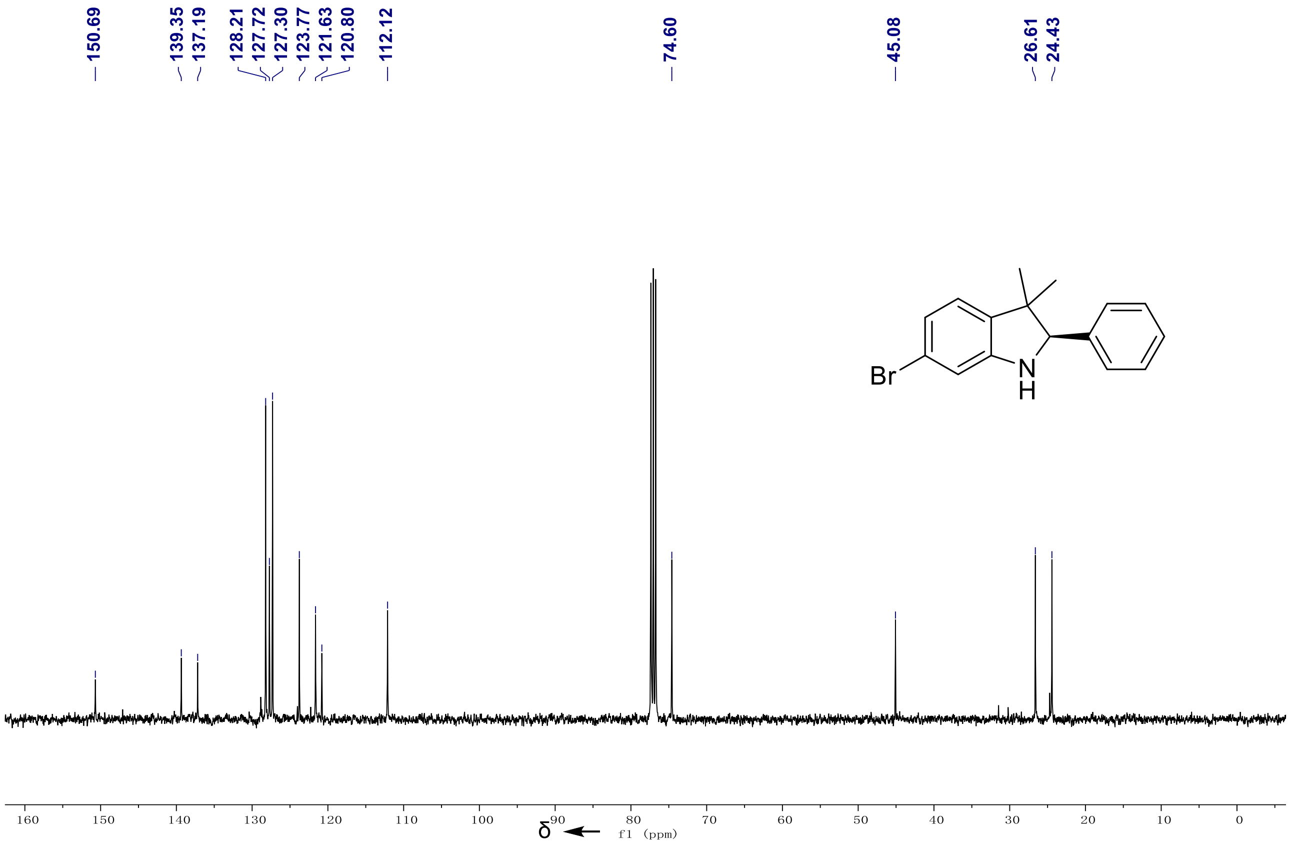

(*R*)-1g: (R)- 3,3-dimethyl-2-phenyl-5-(trifluoromethyl)indoline.

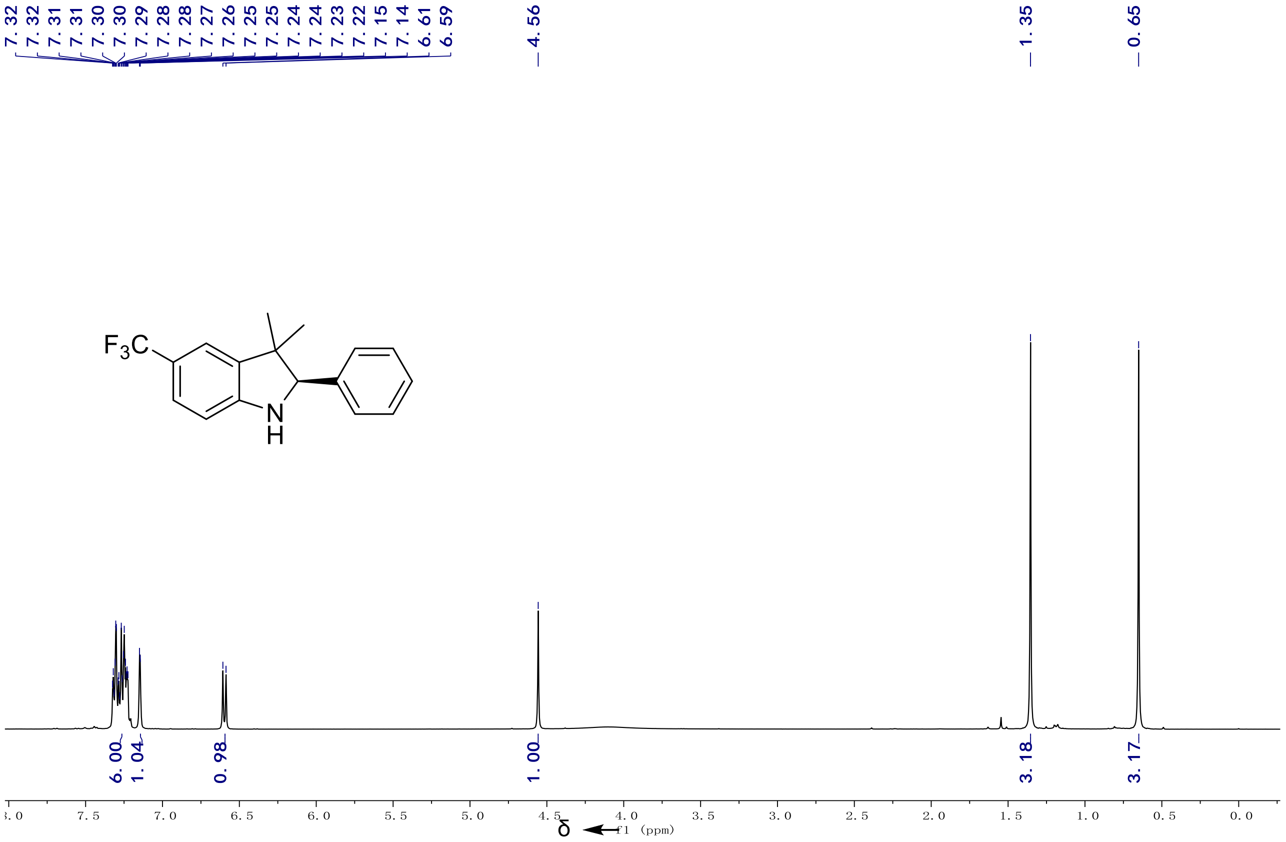

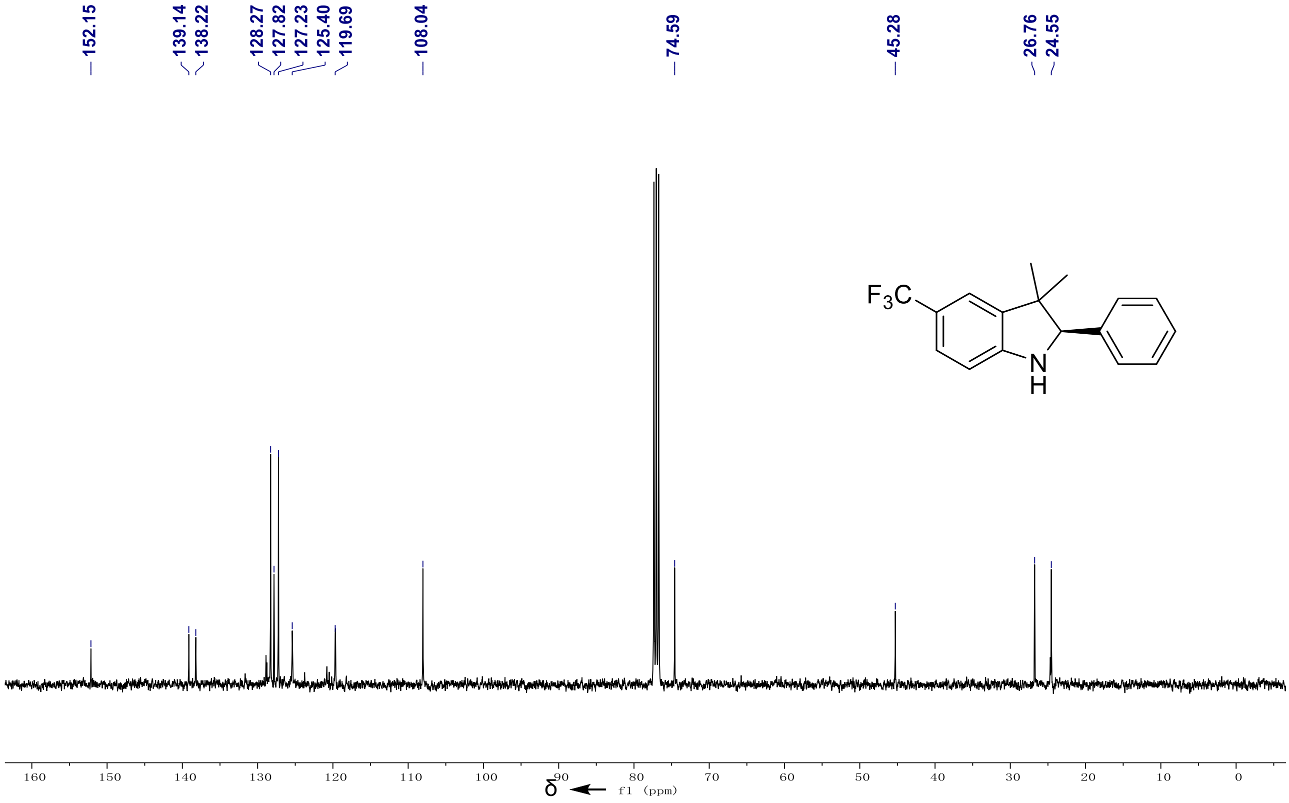

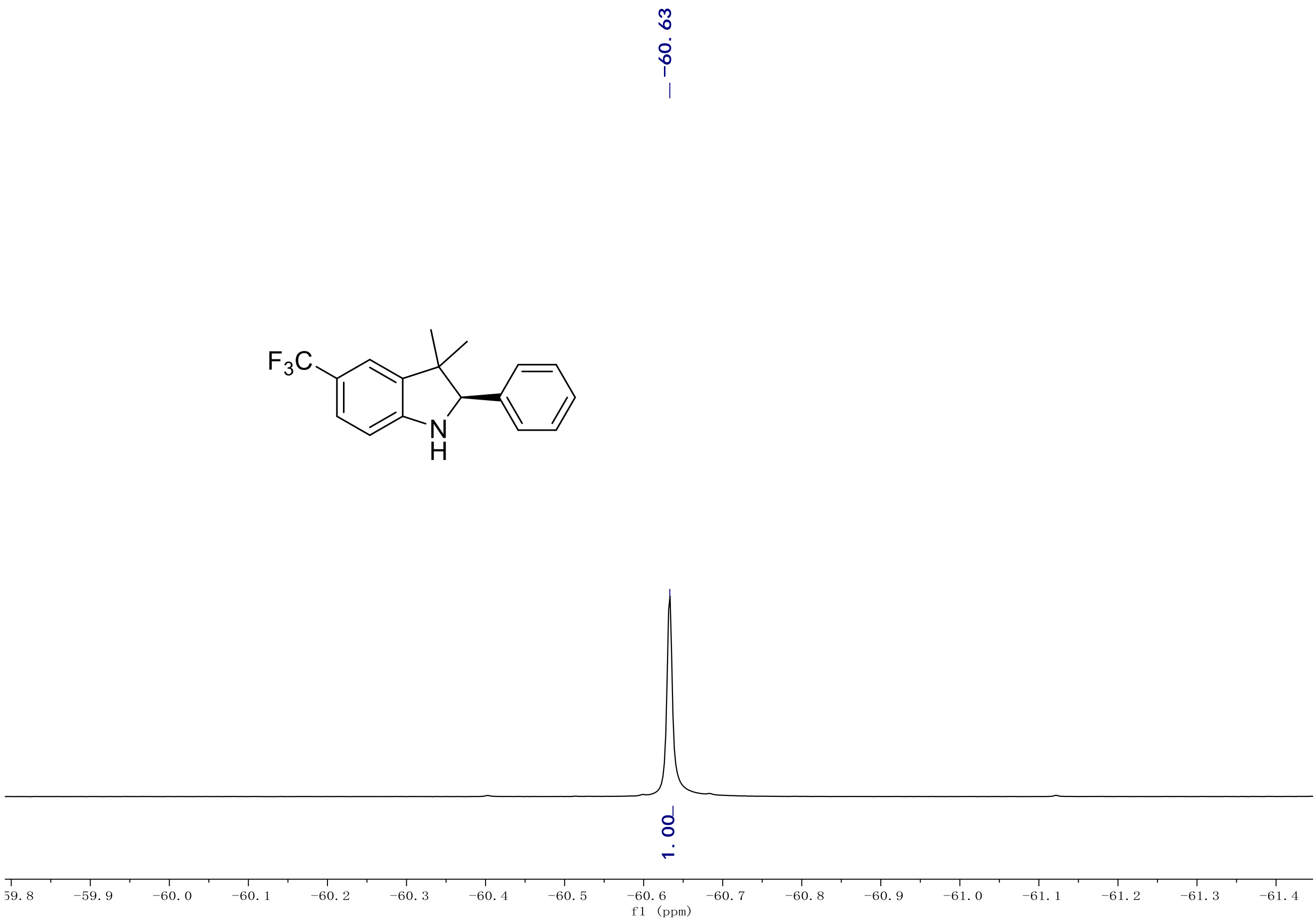

—60.63
F3C
N
H
1.00
59.8
−59.9
−60.0
−60.1
−60.2
−60.3
−60.4
−60.5
−60.6
−60.7
−60.8
−60.9
−61.0
−61.1
−61.2
−61.3
−61.4
f1 (ppm)

(*R*)-1h: (R)-3,3,5-trimethyl-2-phenylindoline.

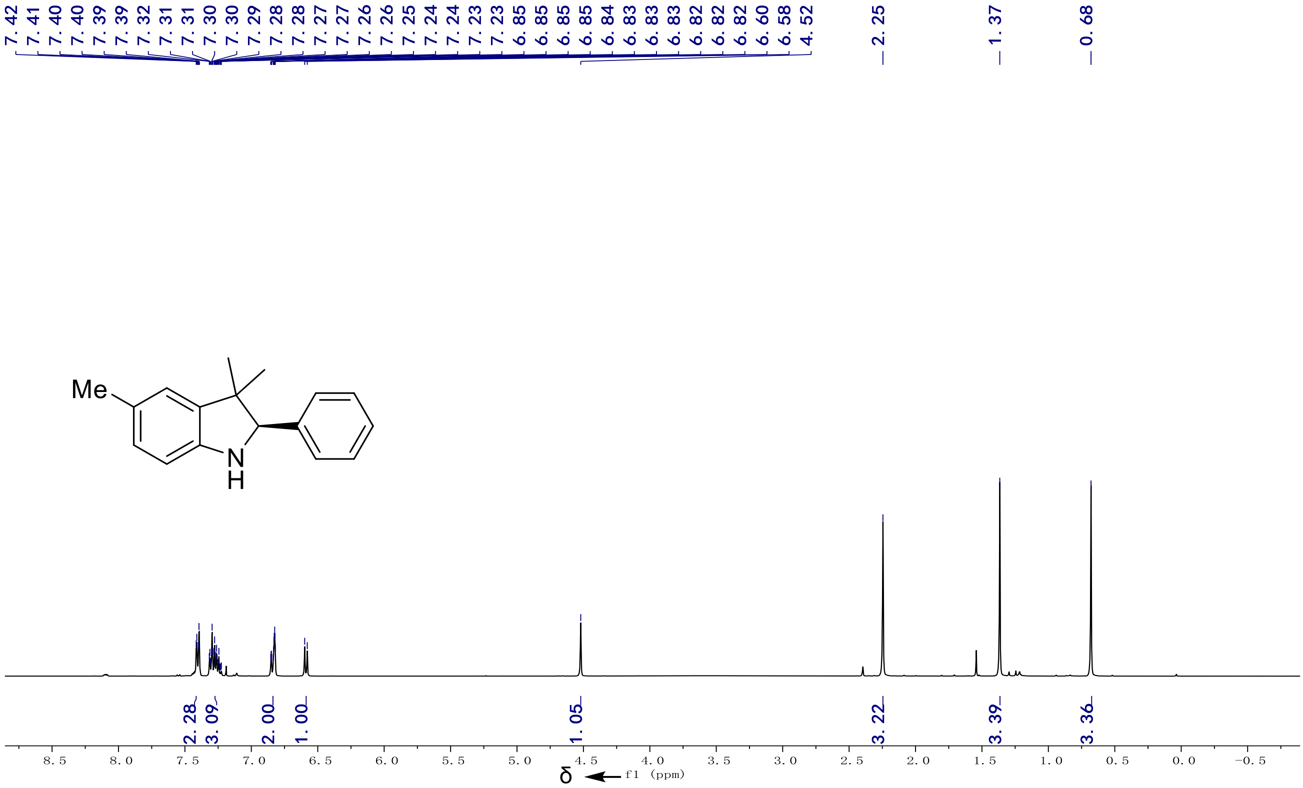

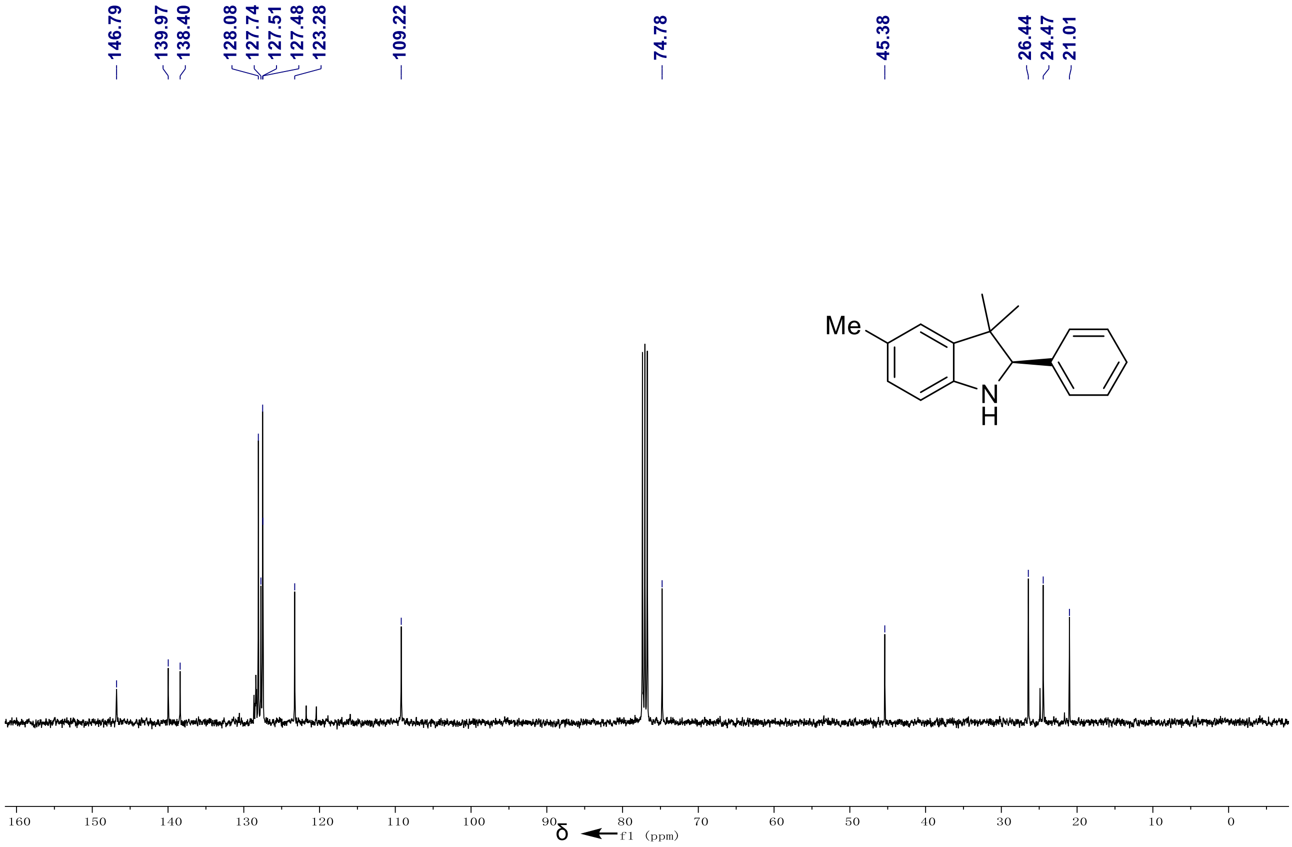

(*R*)-1i: (R)- 5-methoxy-3,3-dimethyl-2-phenylindoline.

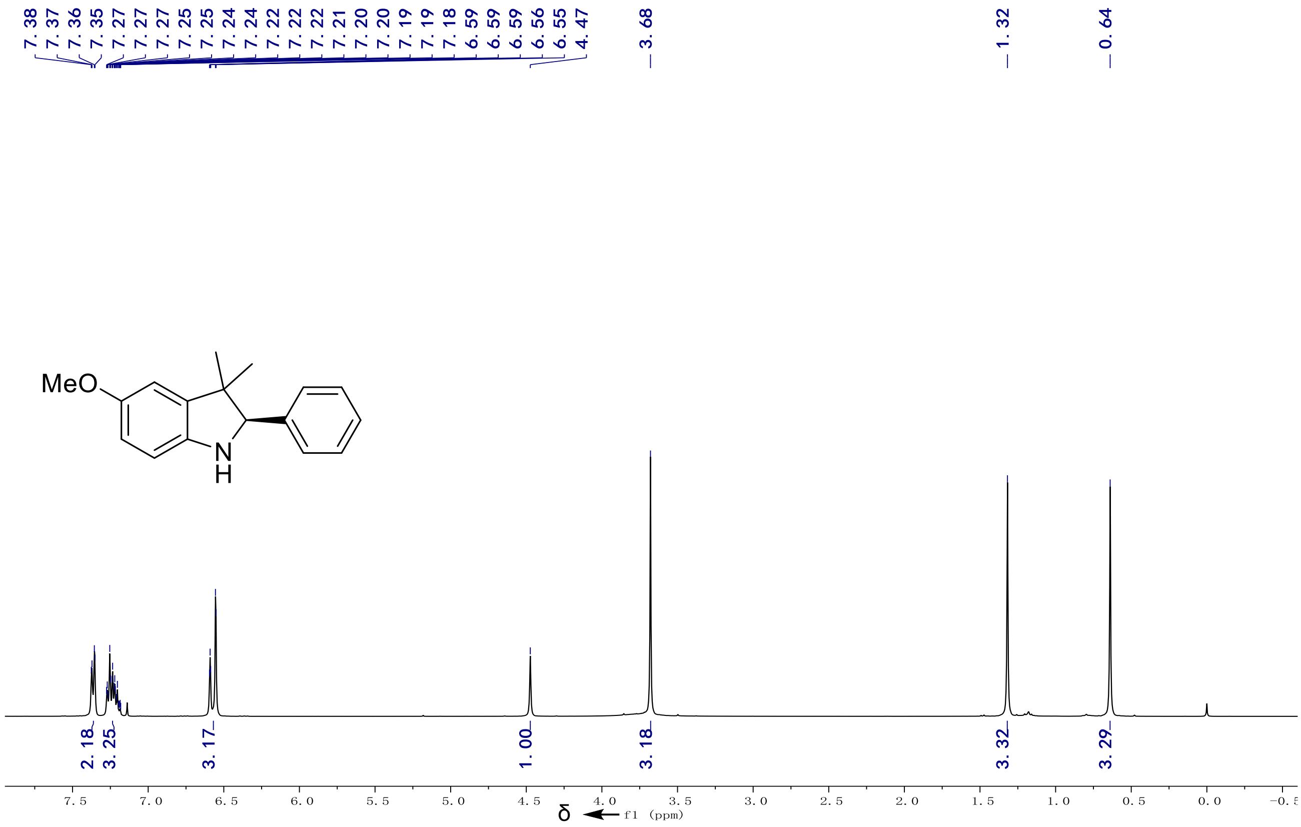

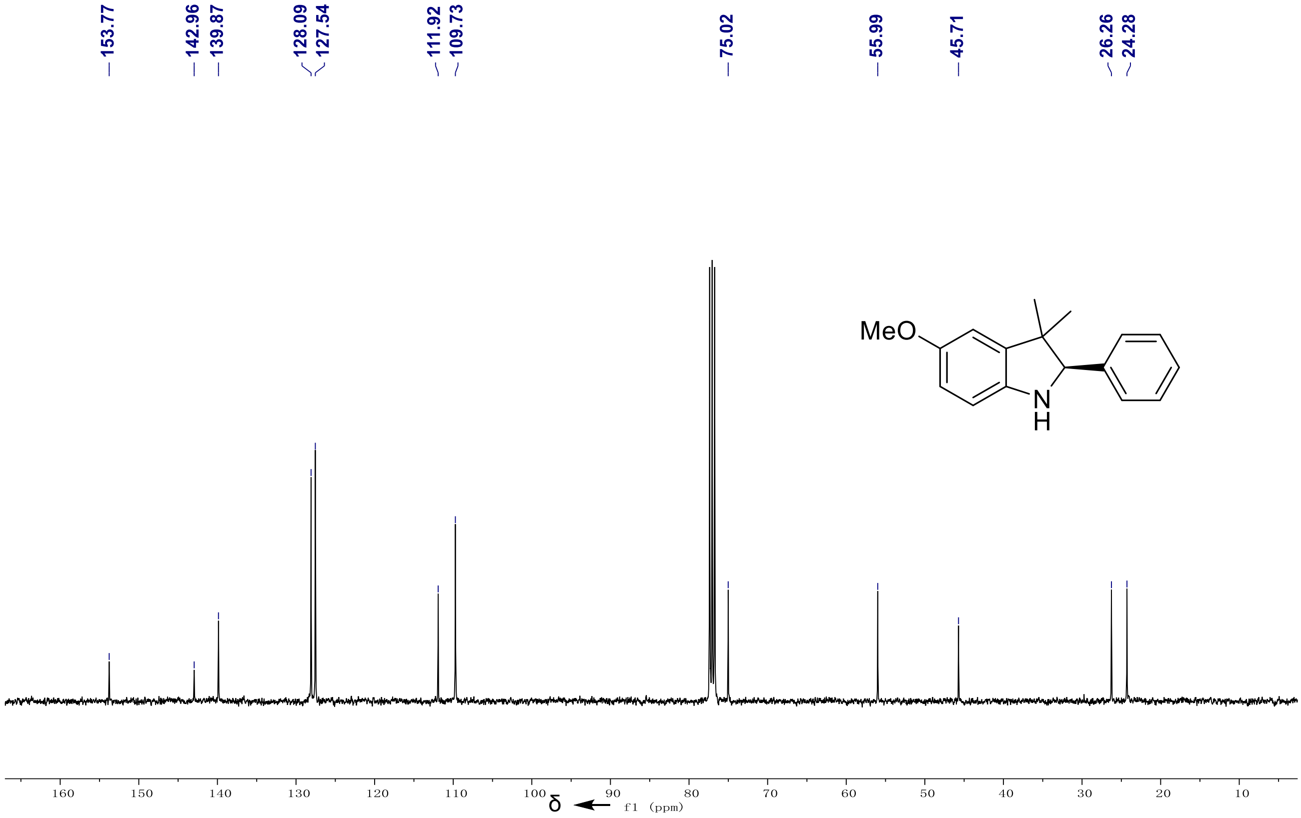

(*R*)-1j: (R)- 5-(benzyloxy)-3,3-dimethyl-2-phenylindoline.

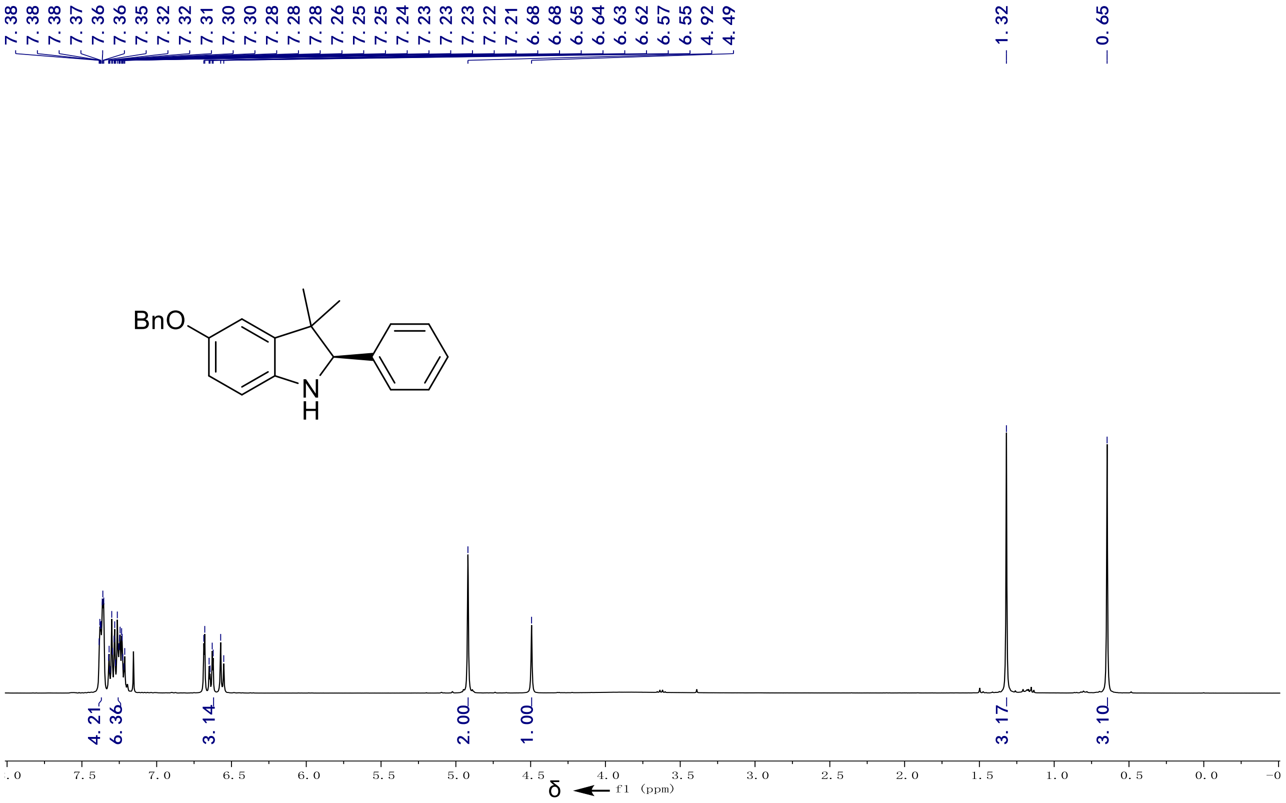

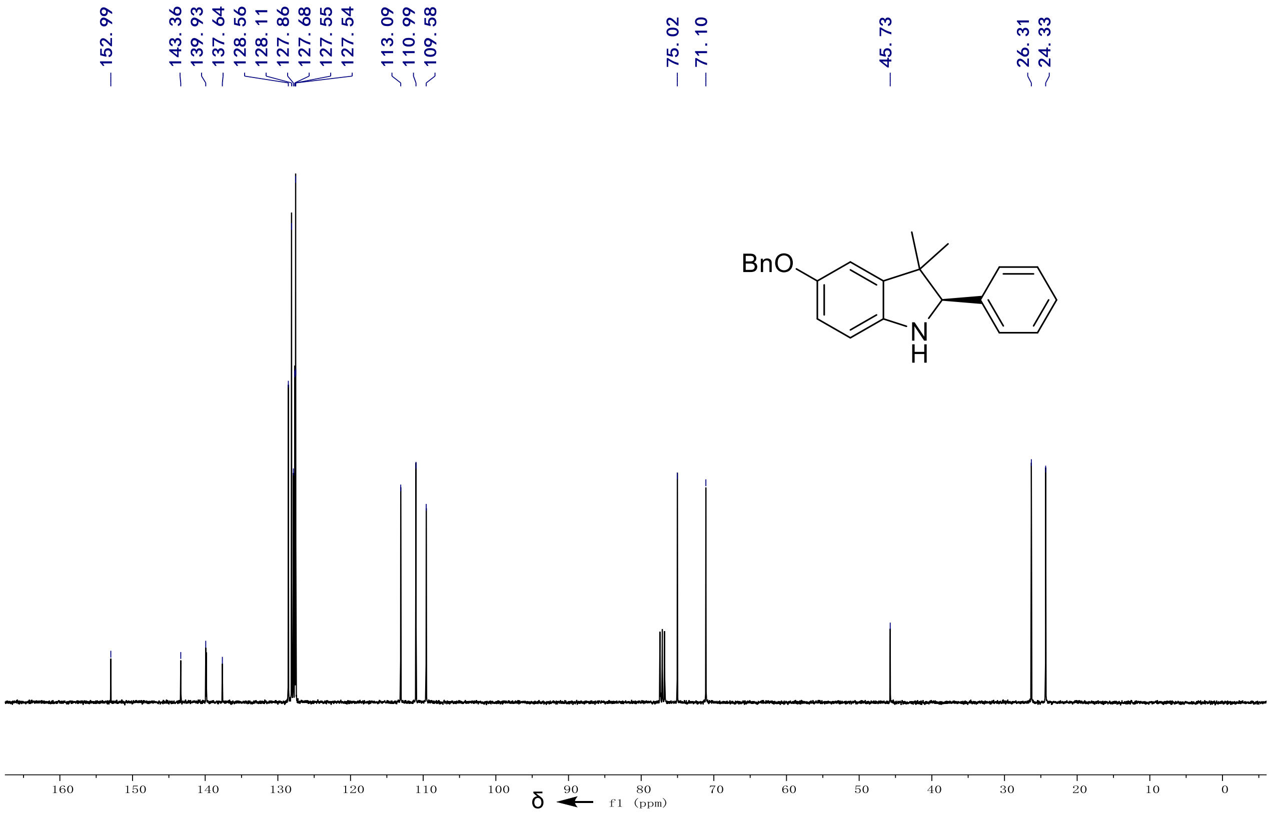

(*R*)-1k: (R)- 3,3-dimethyl-2-phenyl-5-(trifluoromethoxy)indoline.

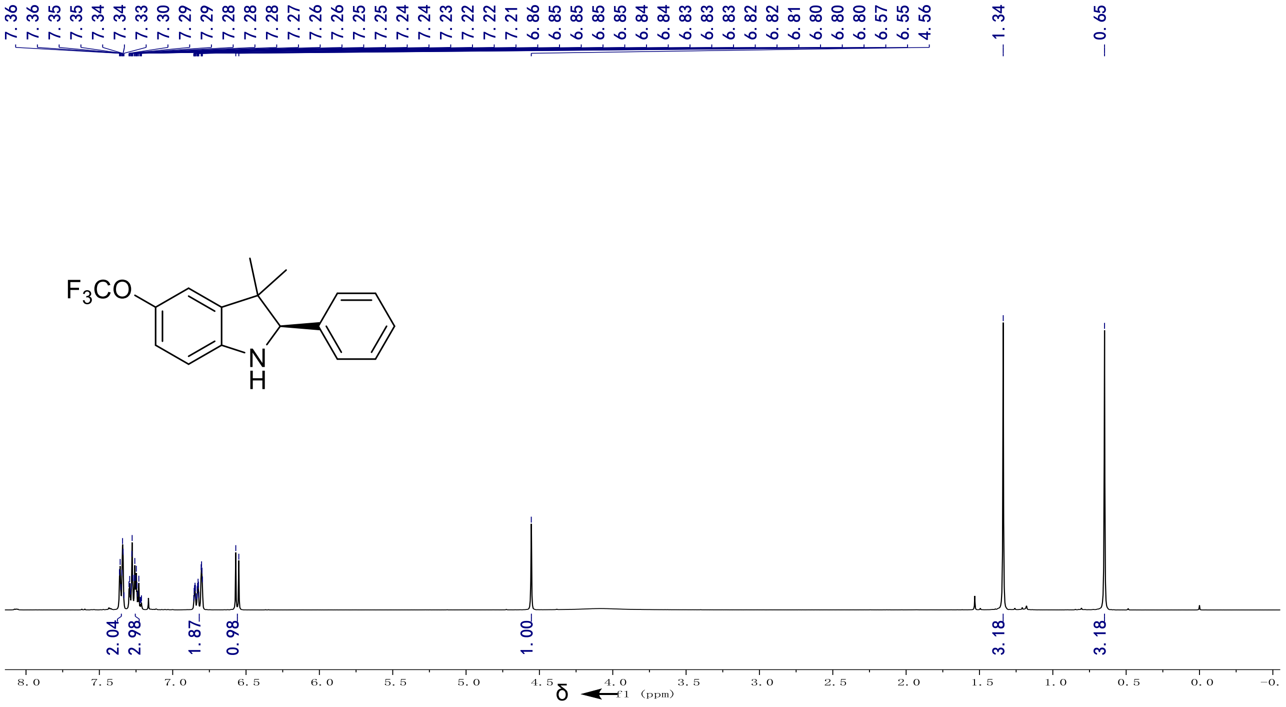

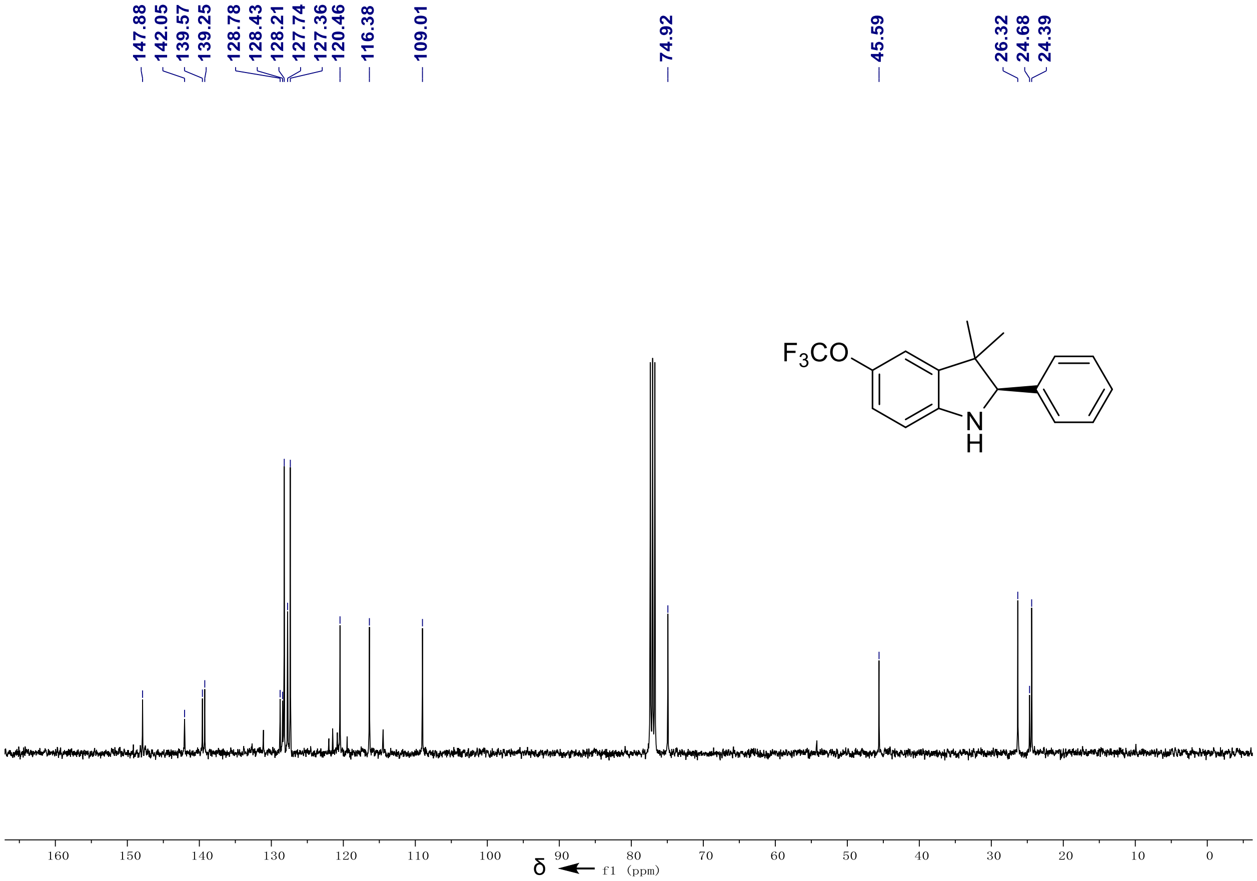

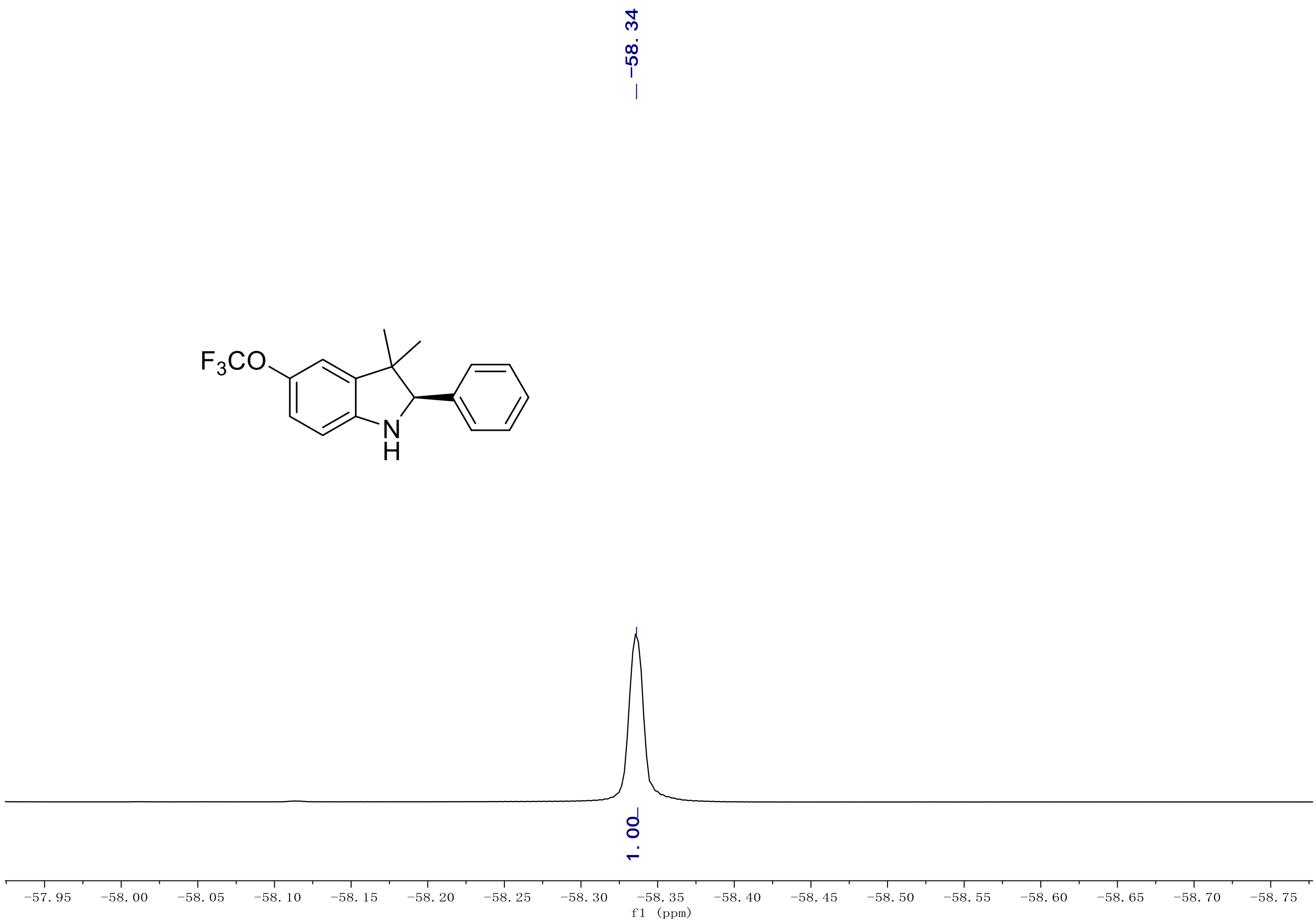

−58.34
F3CO
N
H
1.00
−57.95 −58.00 −58.05 −58.10 −58.15 −58.20 −58.25 −58.30 −58.35 −58.40 −58.45 −58.50 −58.55 −58.60 −58.65 −58.70 −58.75
f1 (ppm)

(*R*)-1l:(R)-1,1-dimethyl-2-phenyl-2,3-dihydro-1H-benzo[e]indole

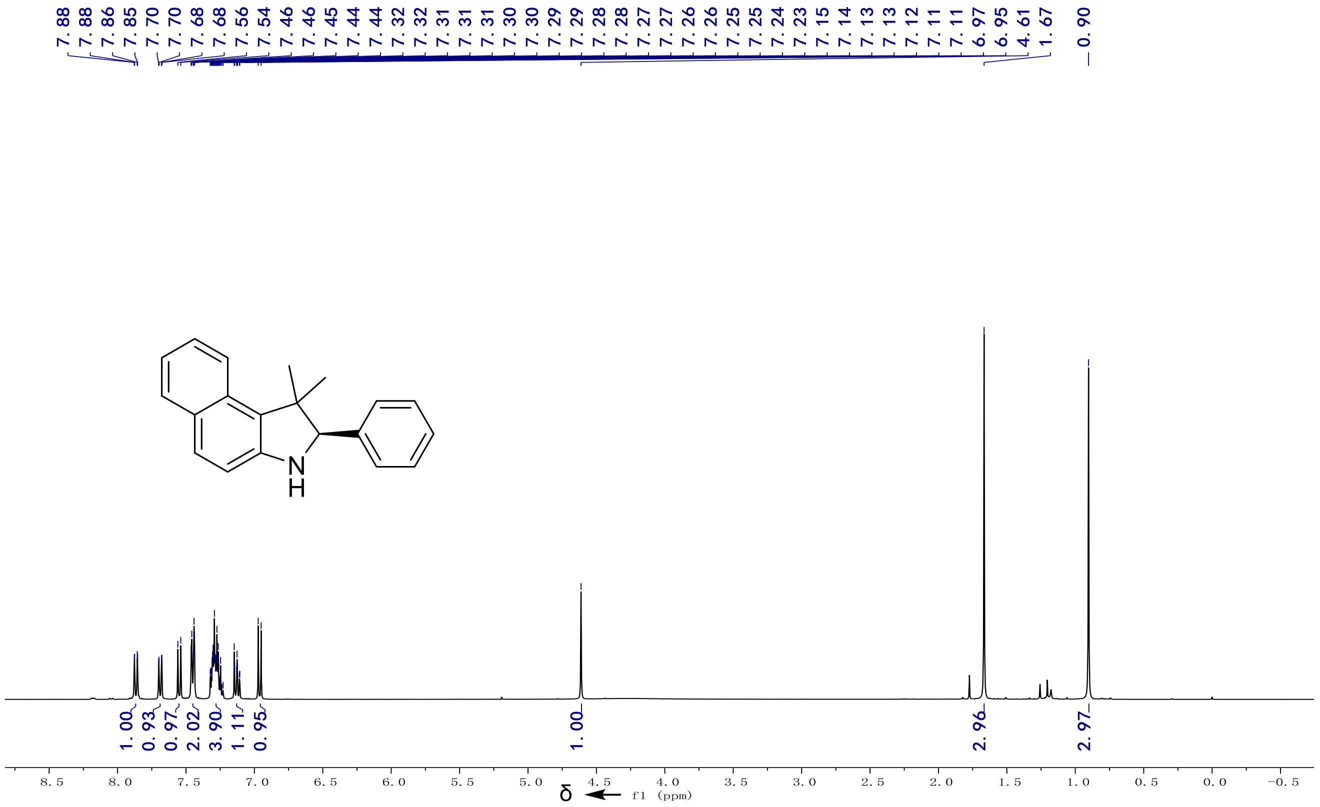

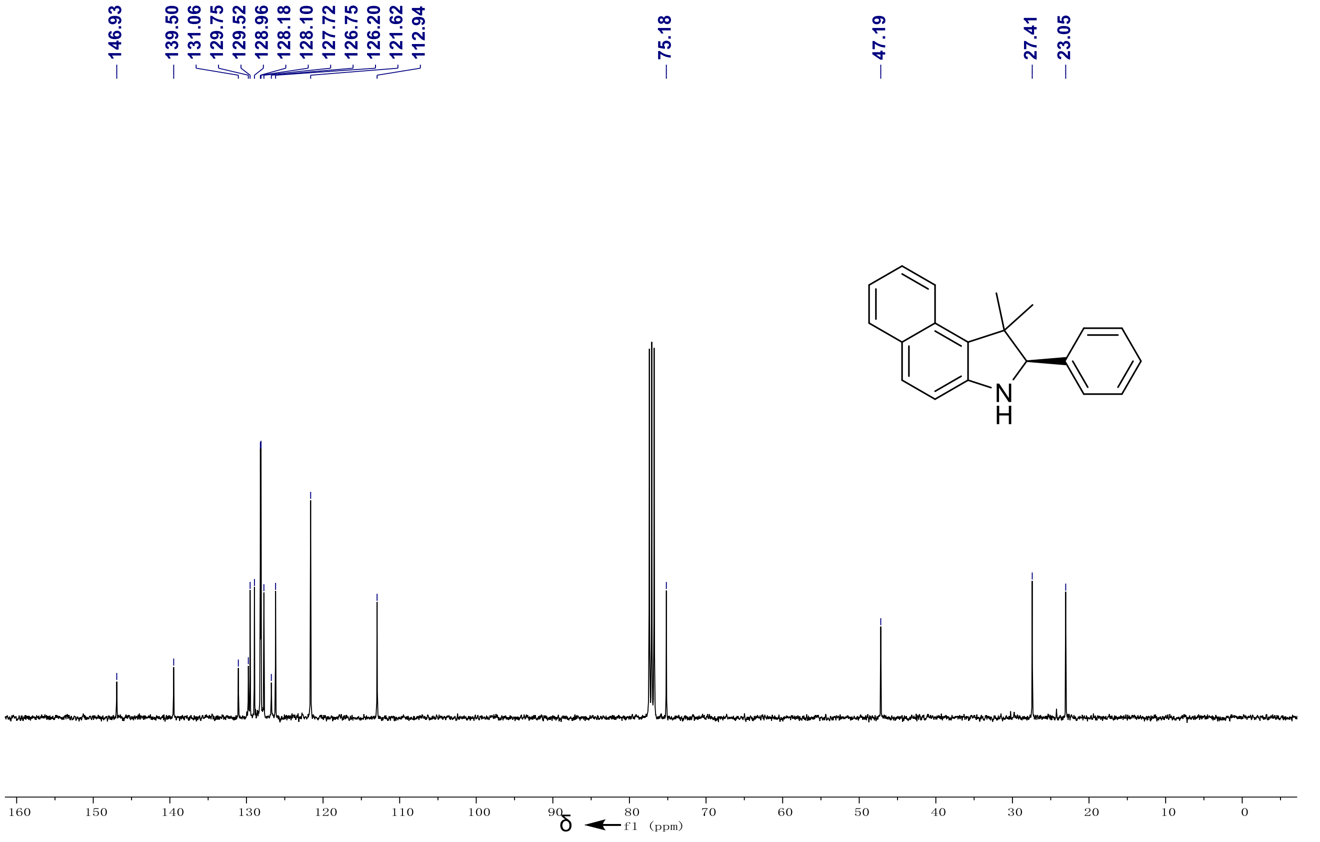

(*R*)-1m：(R)-2-(4-chlorophenyl)-3,3-dimethylindoline.

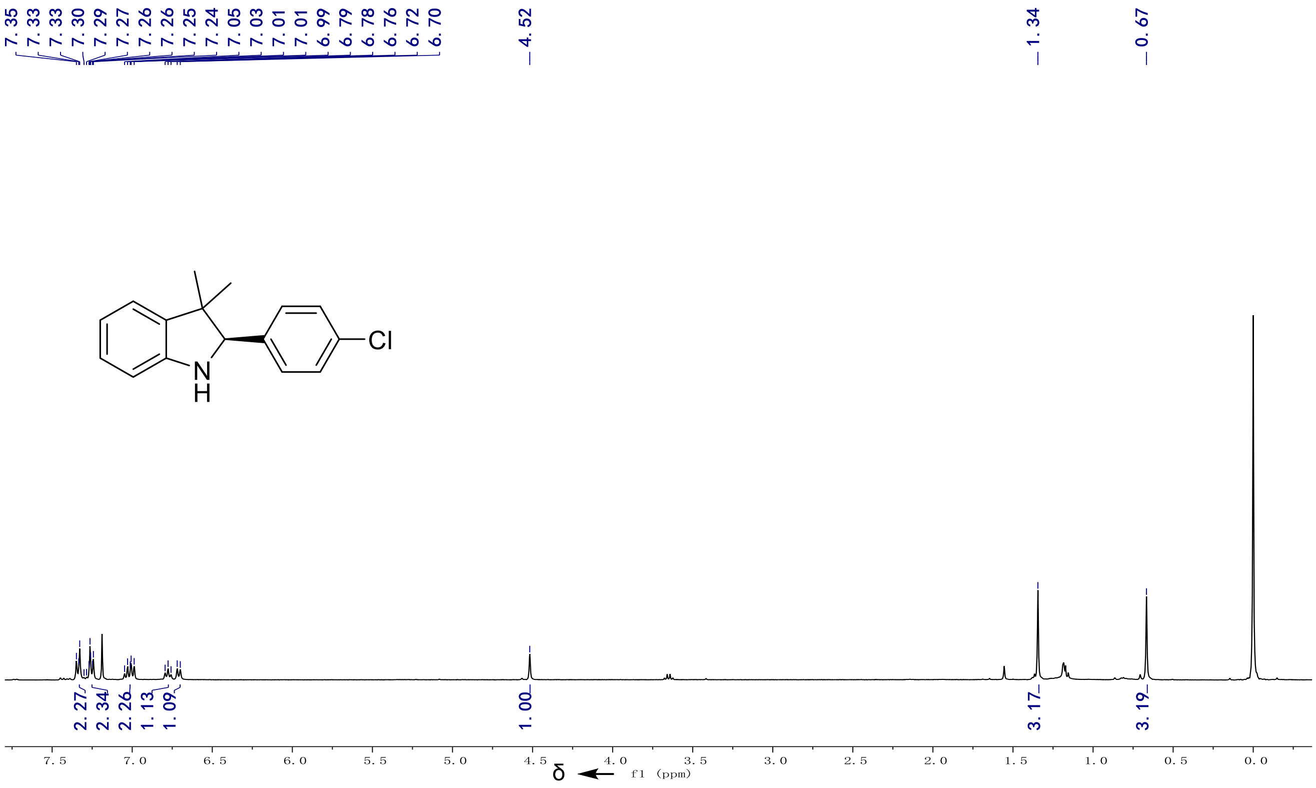

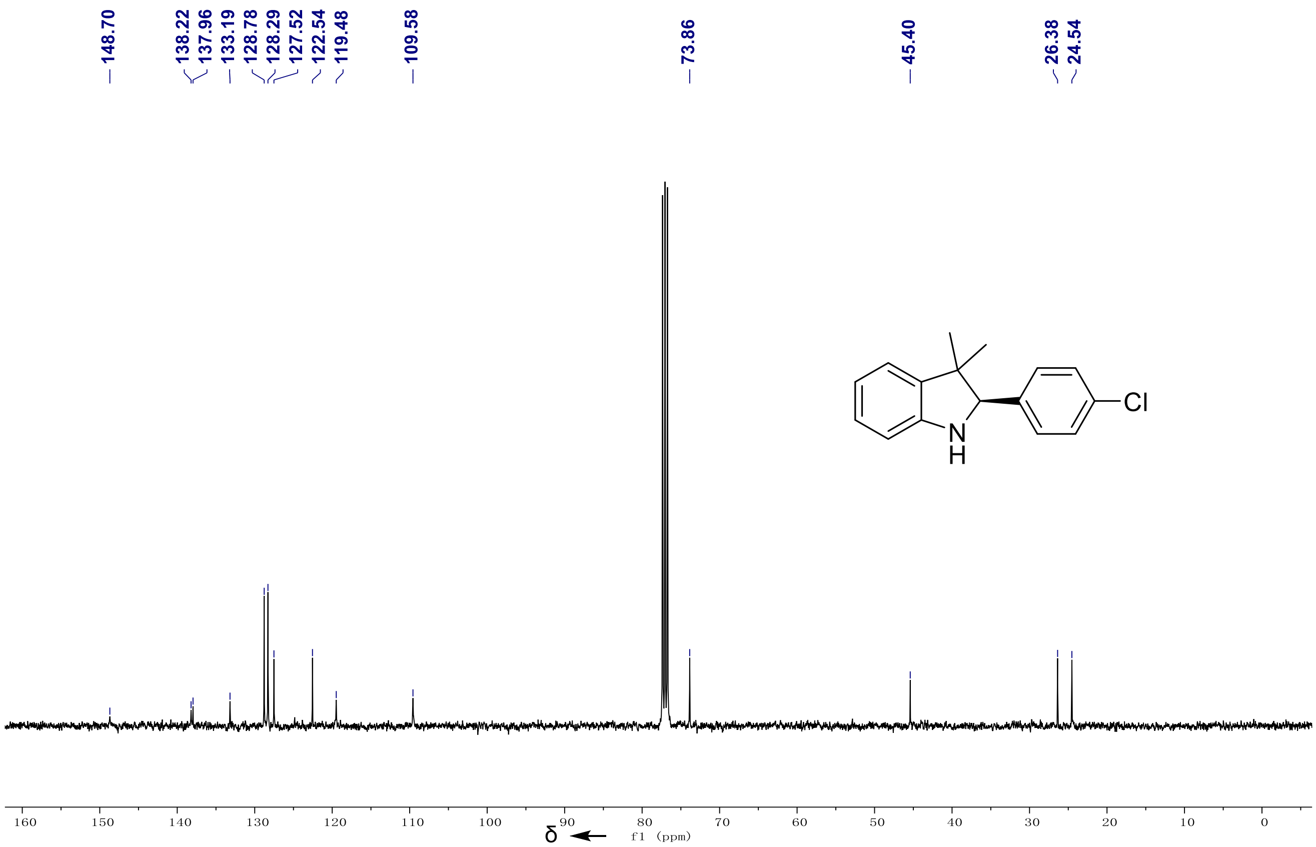

(*R*)-1n: (R)-2-(4-bromophenyl)-3,3-dimethylindoline.

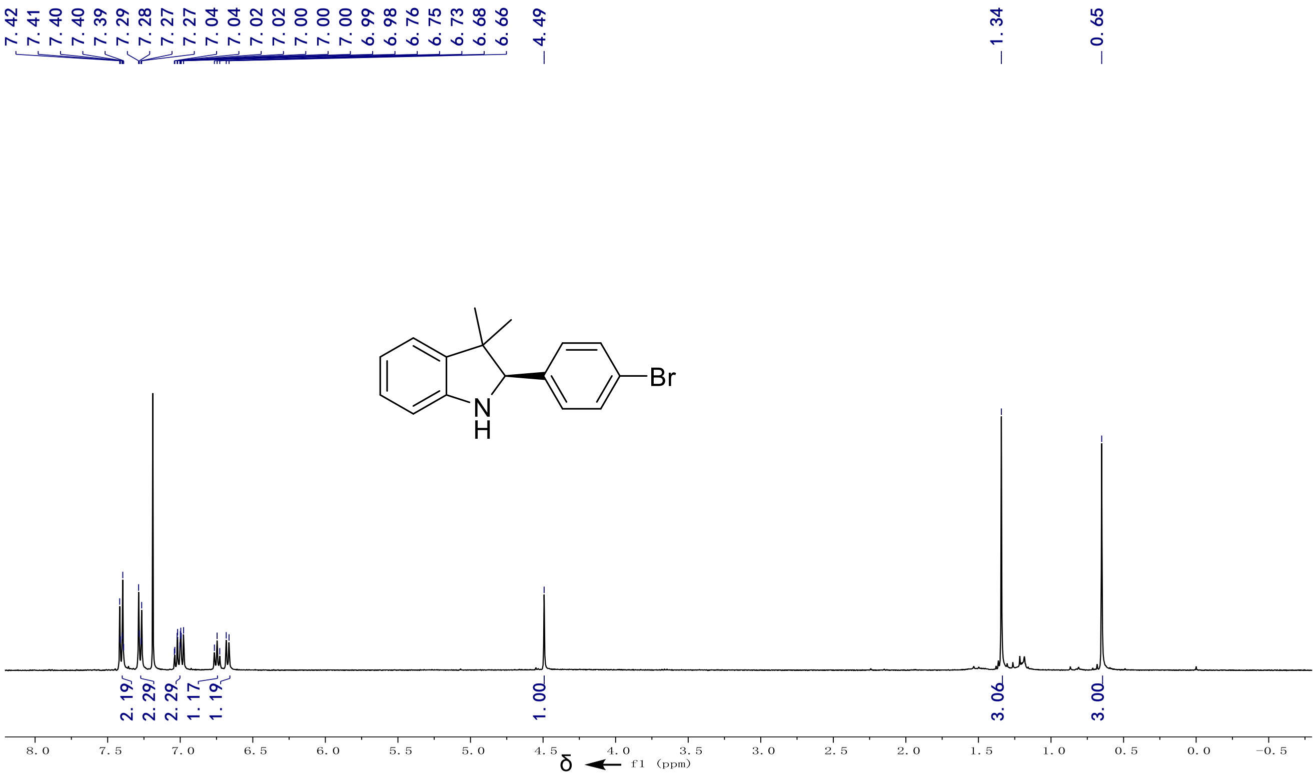

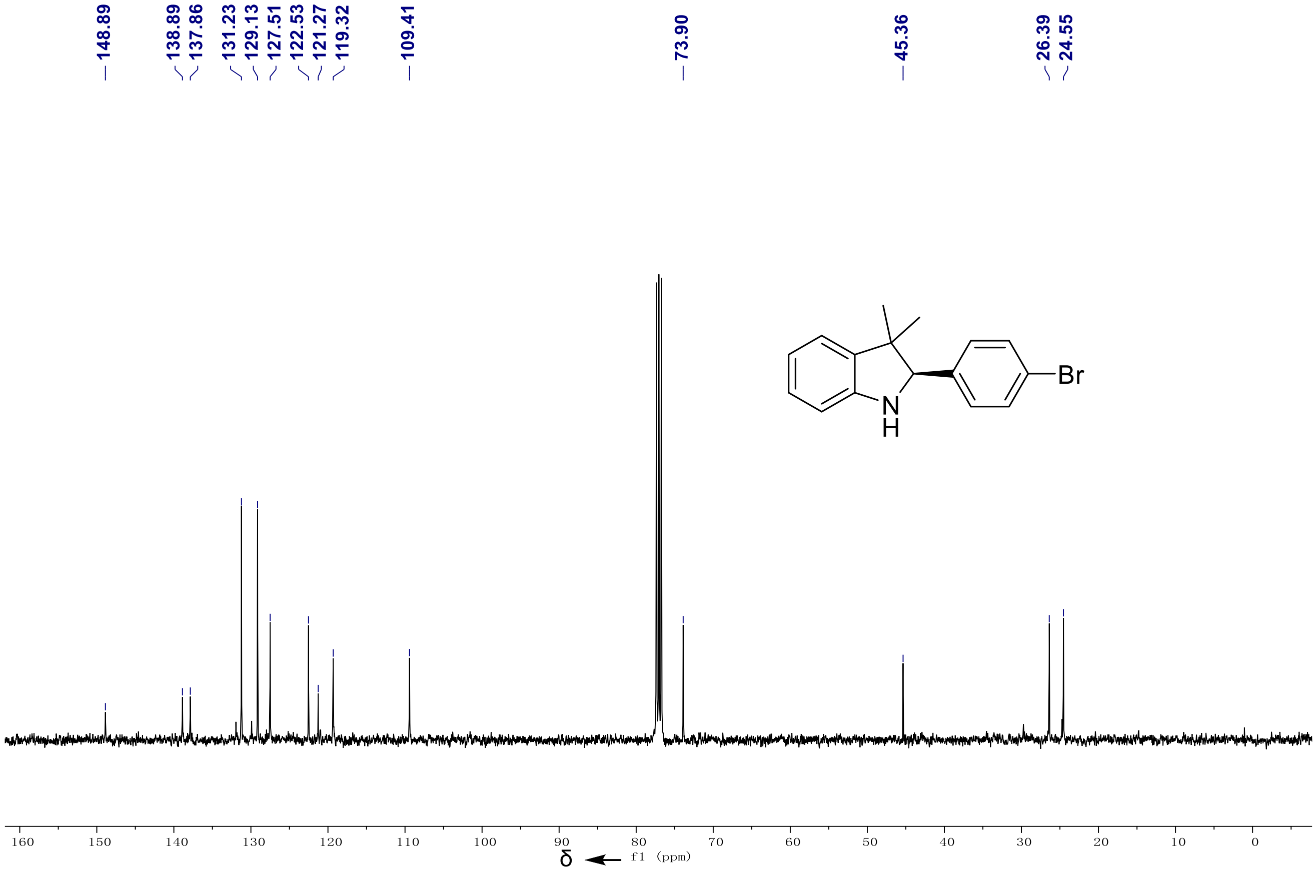

(*R*)-1o：(R)-2-(4-ethylphenyl)-3,3-dimethylindoline

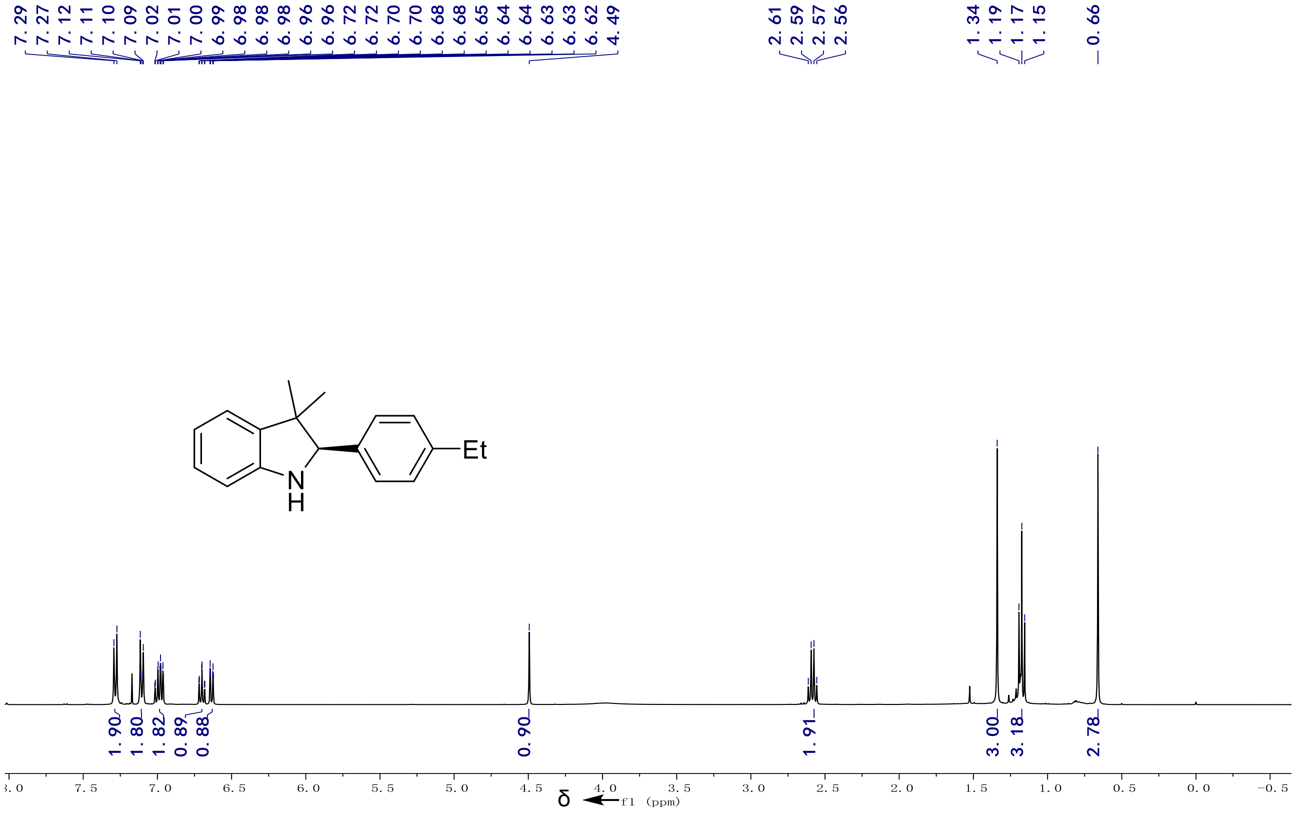

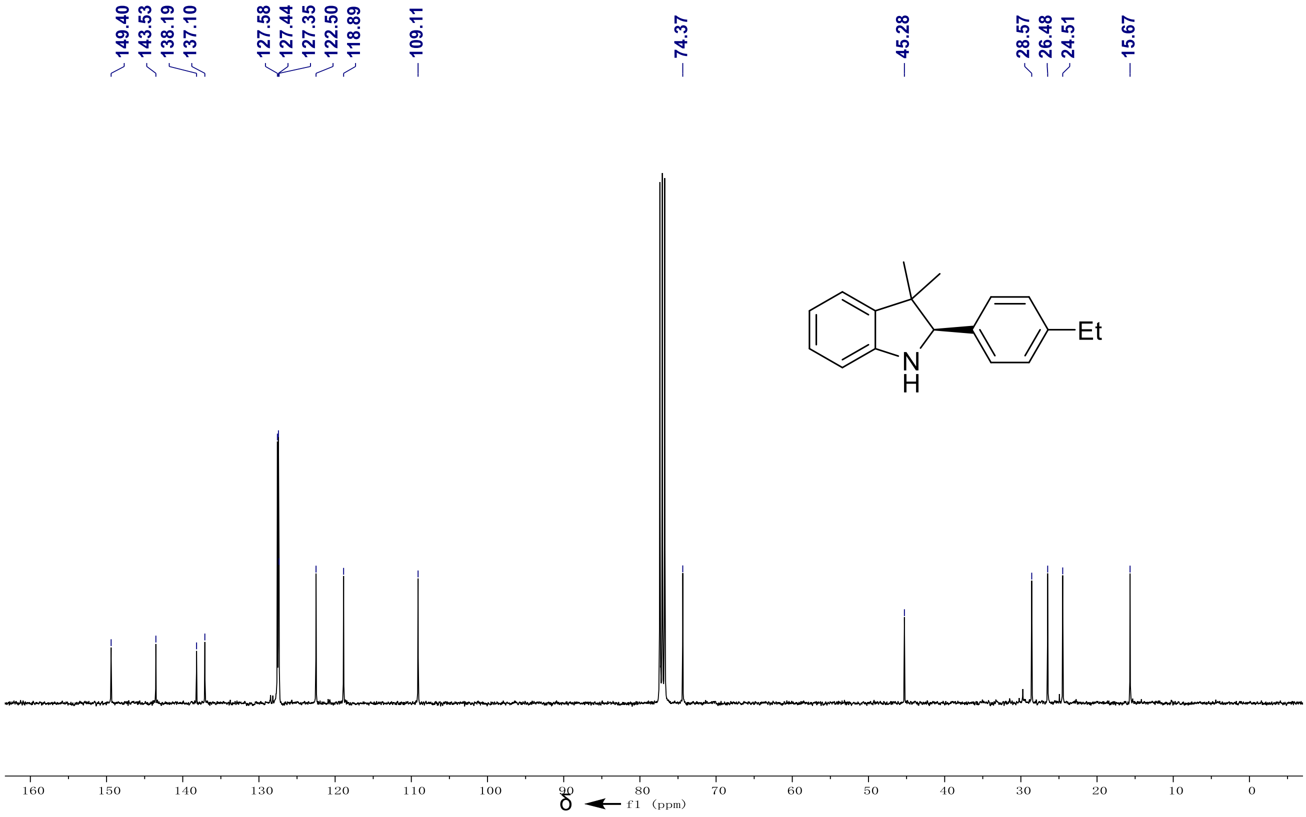

(*R*)-1p: (R)-3,3-dimethyl-2-(4-propoxyphenyl)indoline

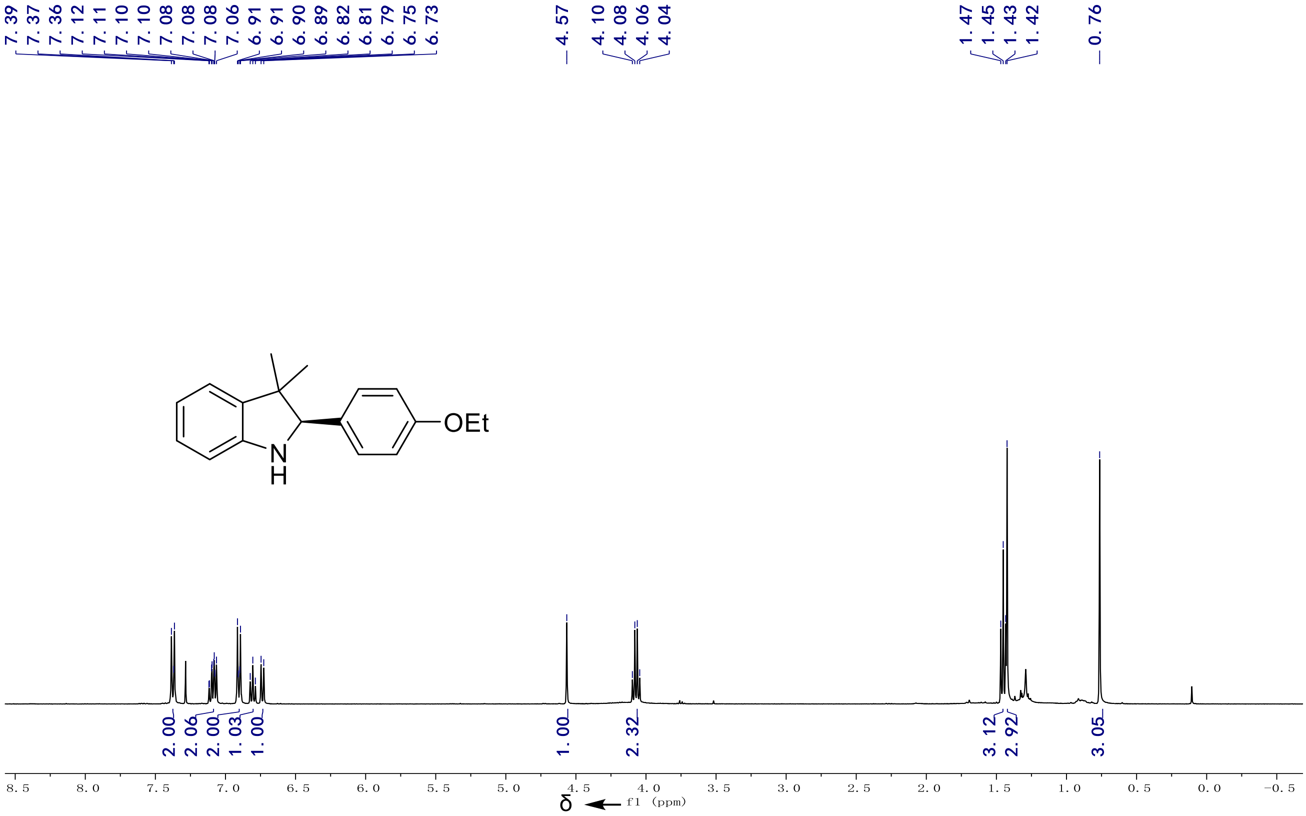

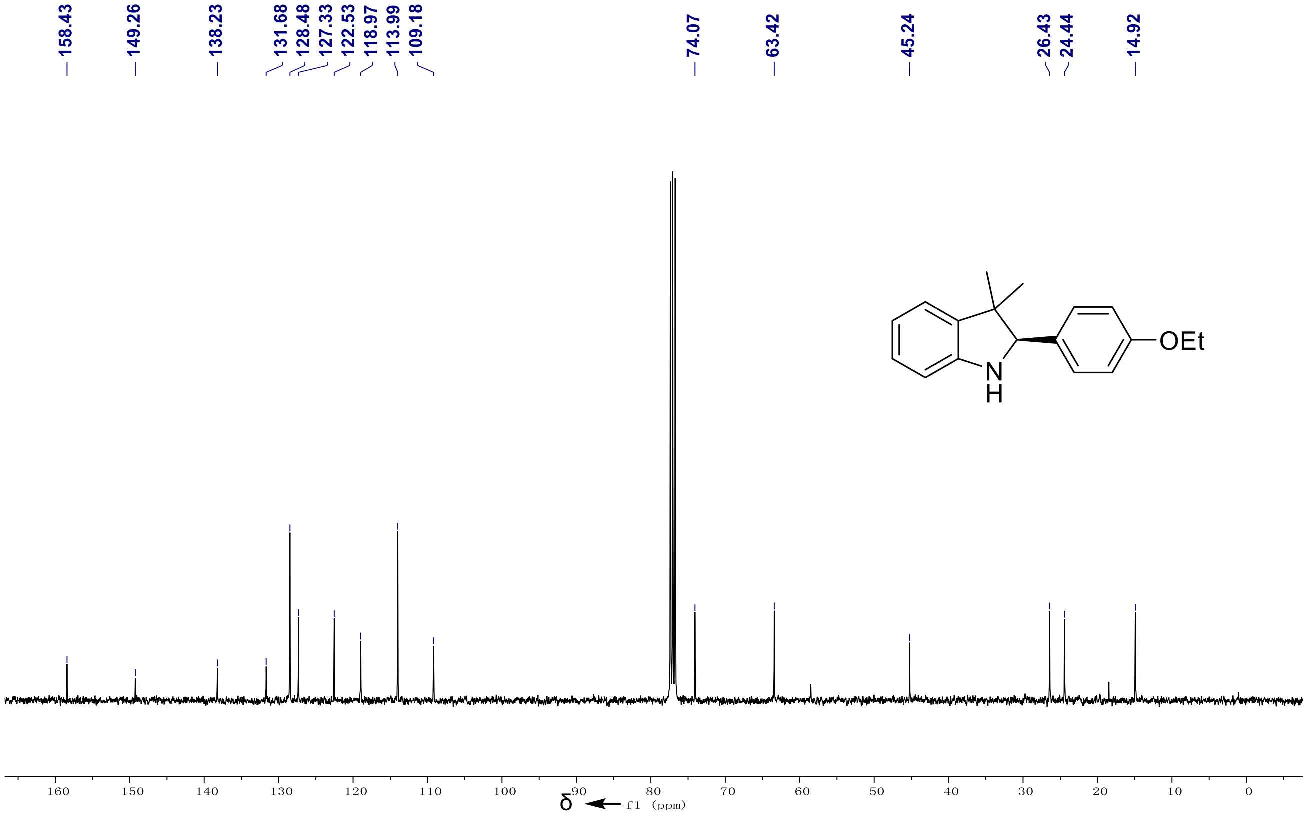

(*R*)-1q: (R)-2'-phenylspiro[cyclopentane-1,3'-indoline].

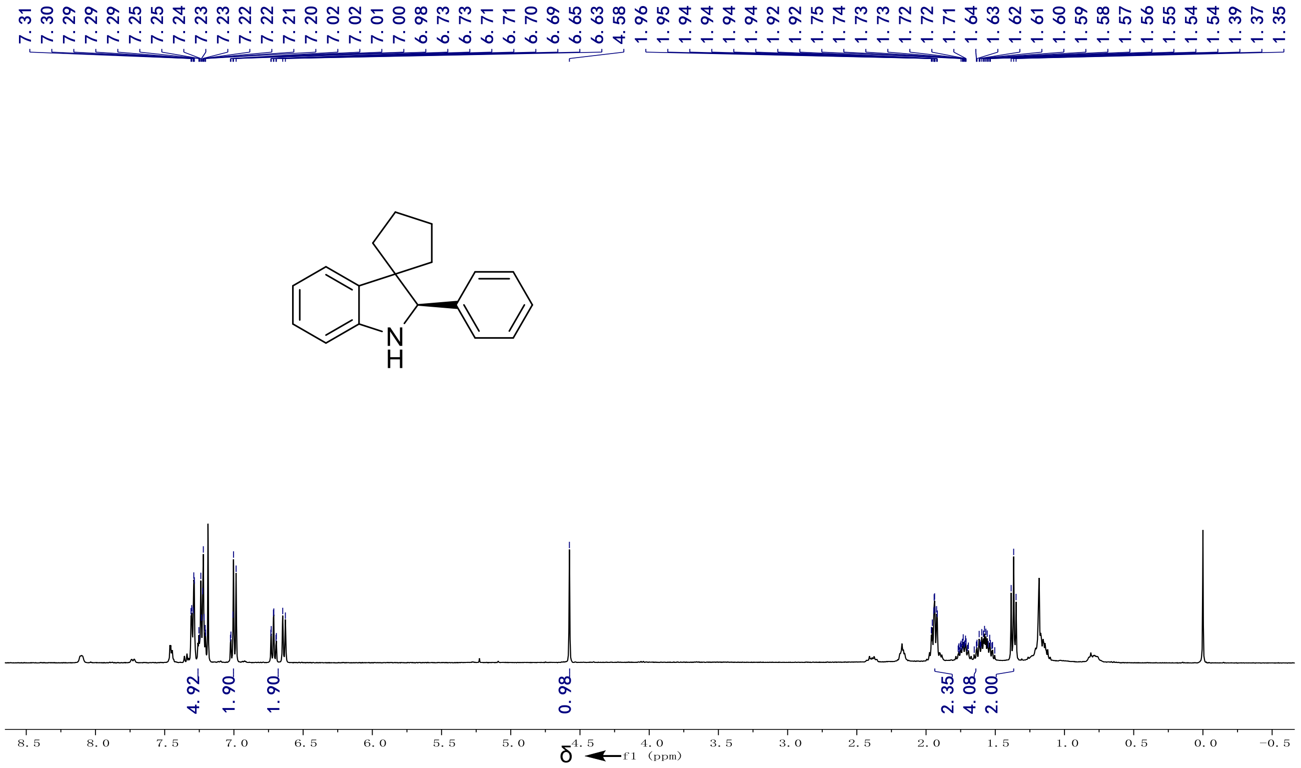

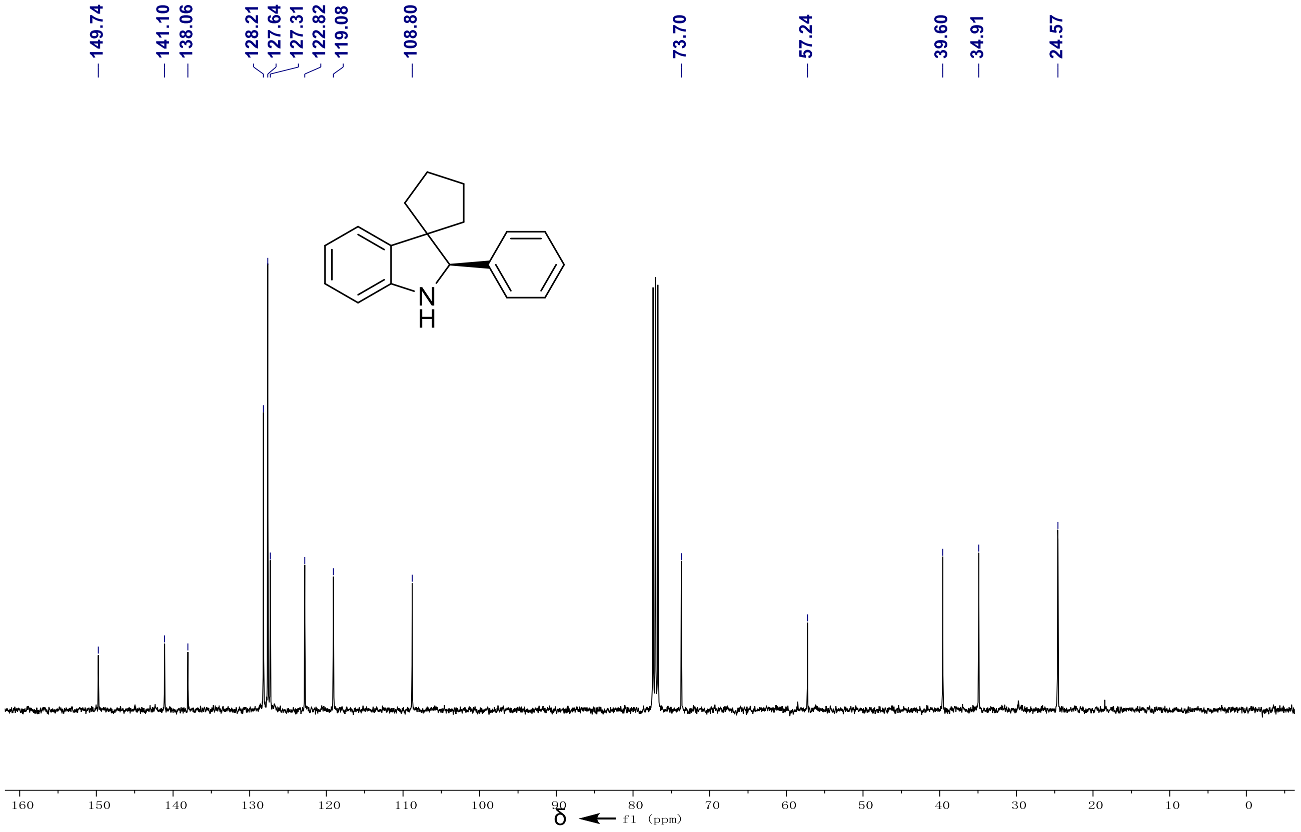

(*R*)-1r：(R)-5'-fluoro-2'-phenylspiro[cyclopentane-1,3'-indoline]

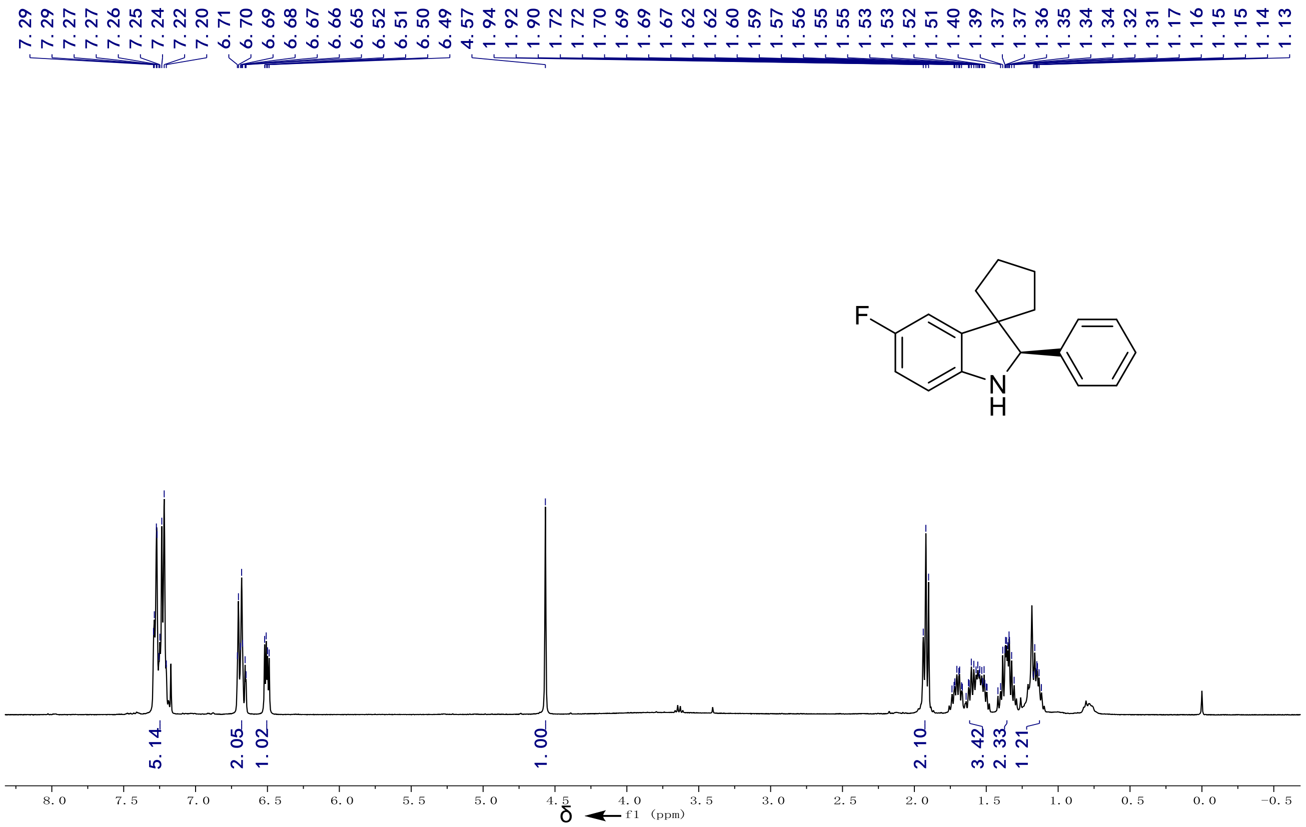

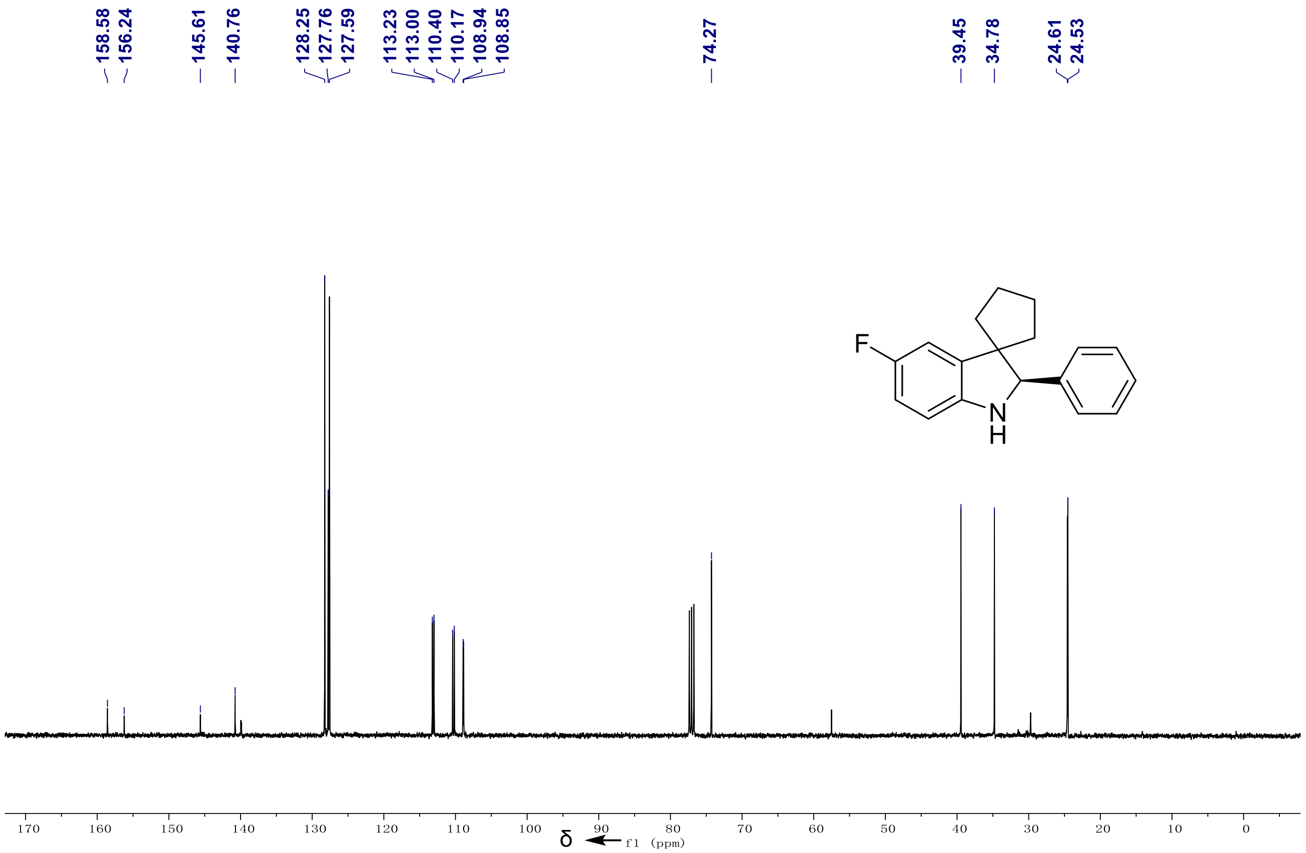

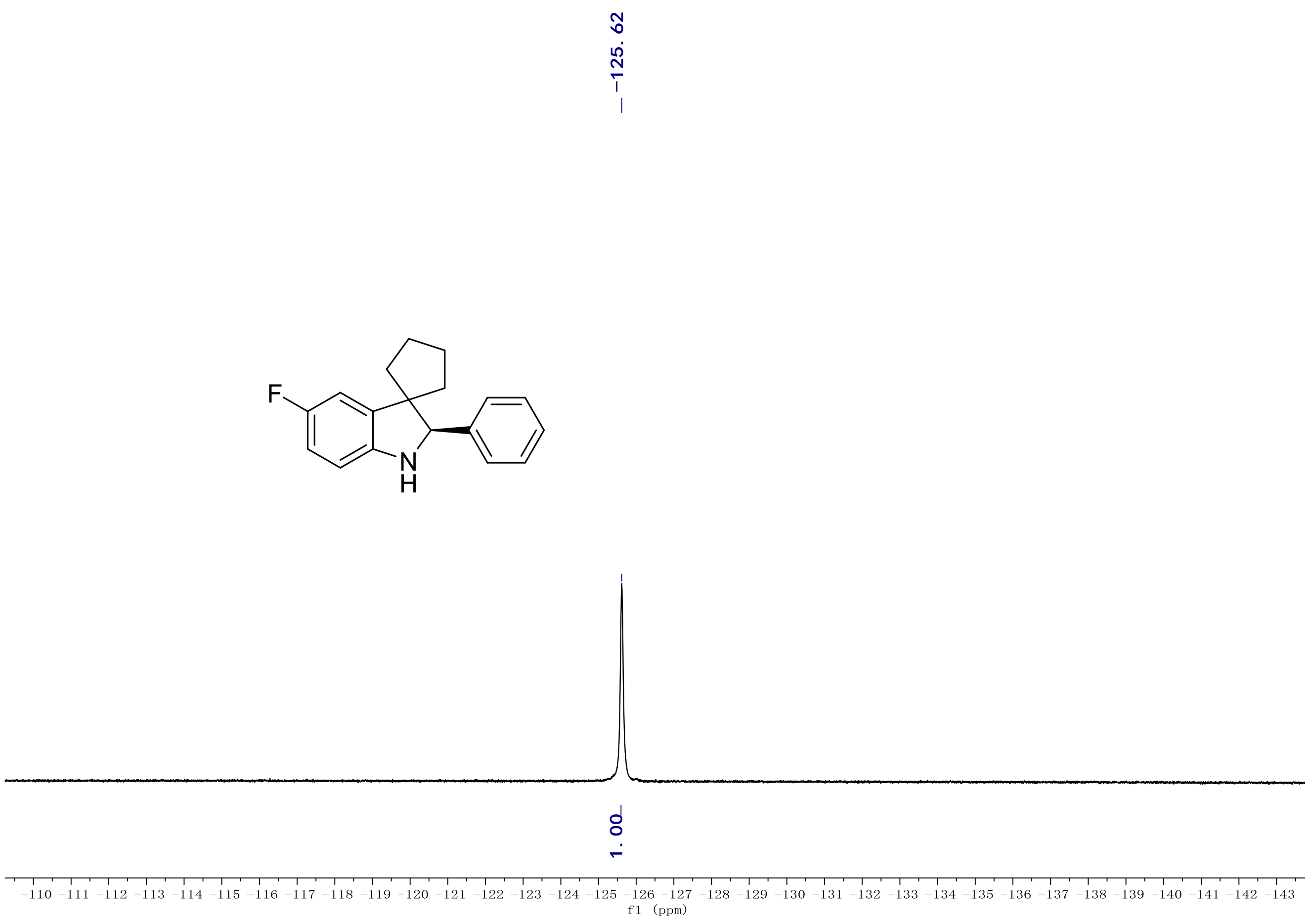

−125.62
F
N
H
1.00
−110 −111 −112 −113 −114 −115 −116 −117 −118 −119 −120 −121 −122 −123 −124 −125 −126 −127 −128 −129 −130 −131 −132 −133 −134 −135 −136 −137 −138 −139 −140 −141 −142 −143
f1 (ppm)

(*R*)-1s: (R)-5'-bromo-2'-phenylspiro[cyclopentane-1,3'-indoline].

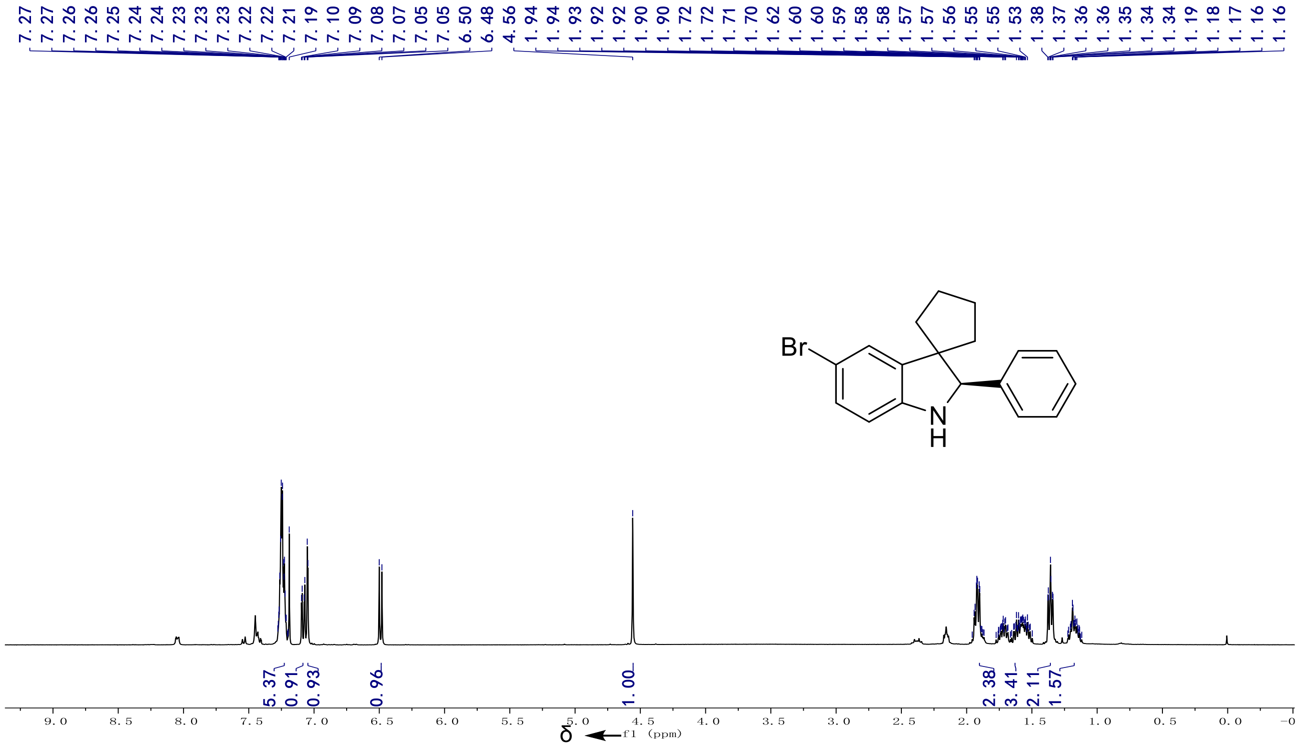

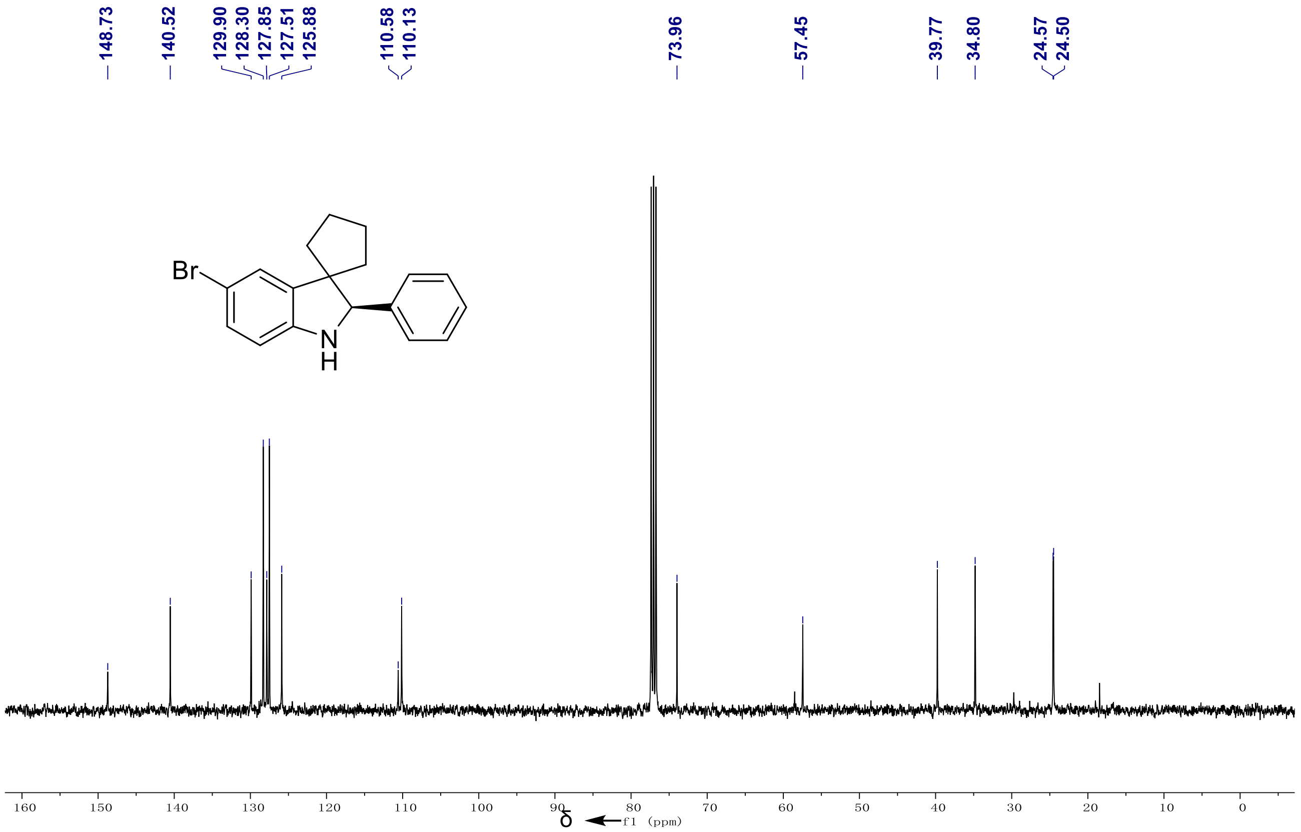

(*R*)-1t: (R)-2'-phenylspiro[cyclohexane-1,3'-indoline].

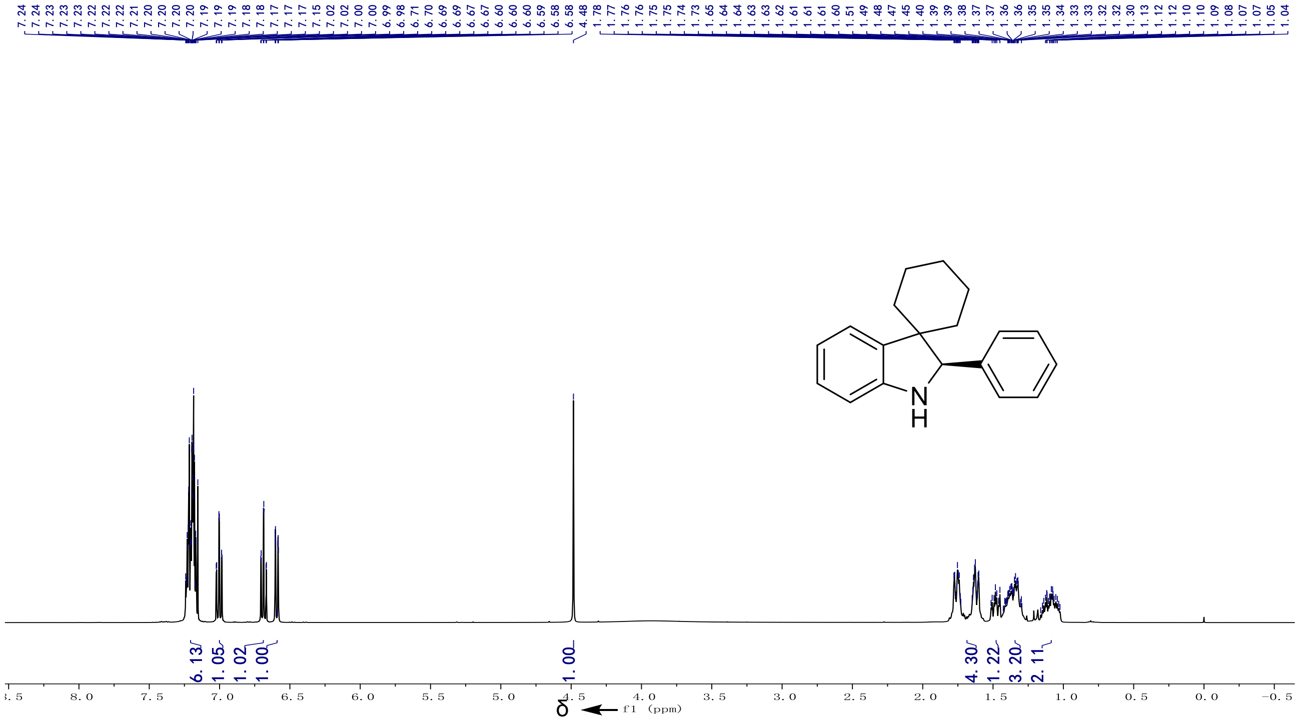

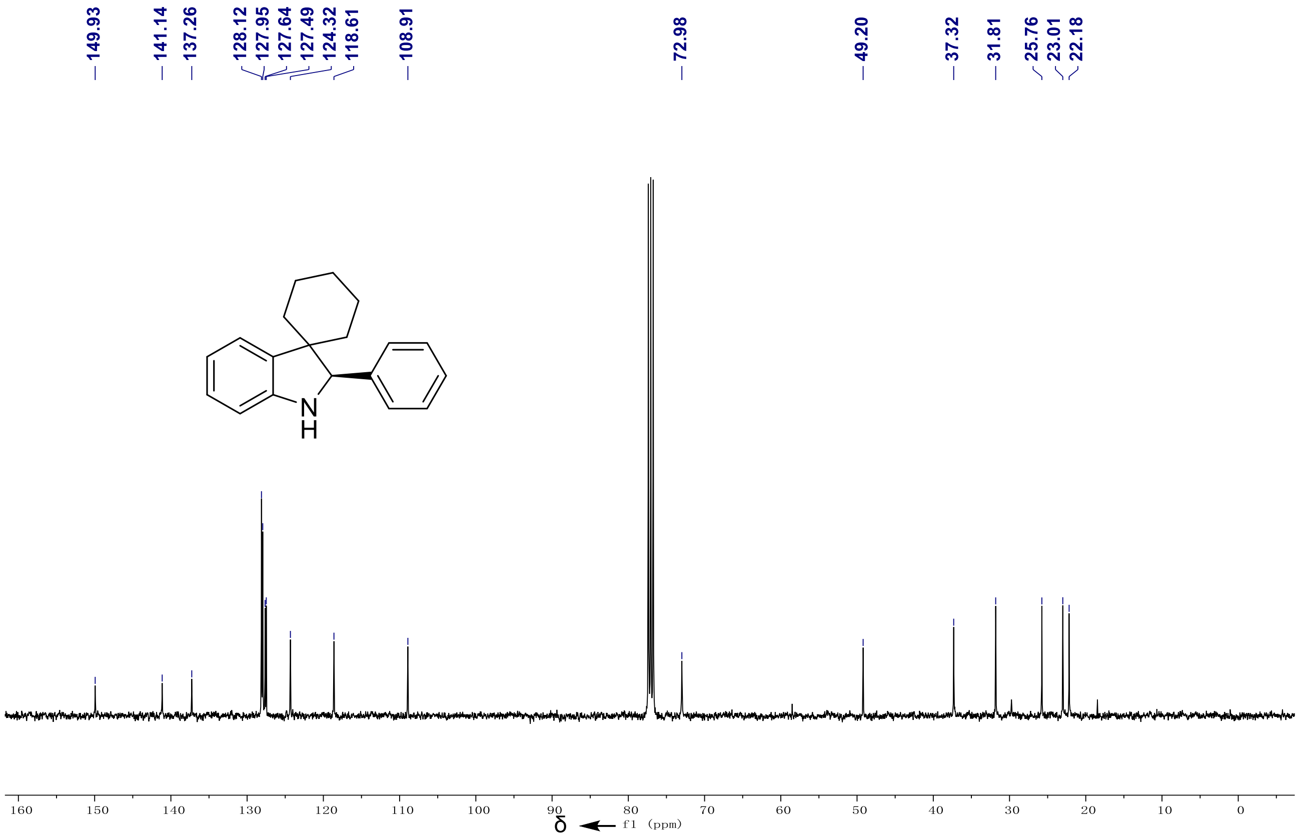

(*R*)-1u: (R)- 5'-fluoro-2'-phenylspiro[cyclohexane-1,3'-indoline].

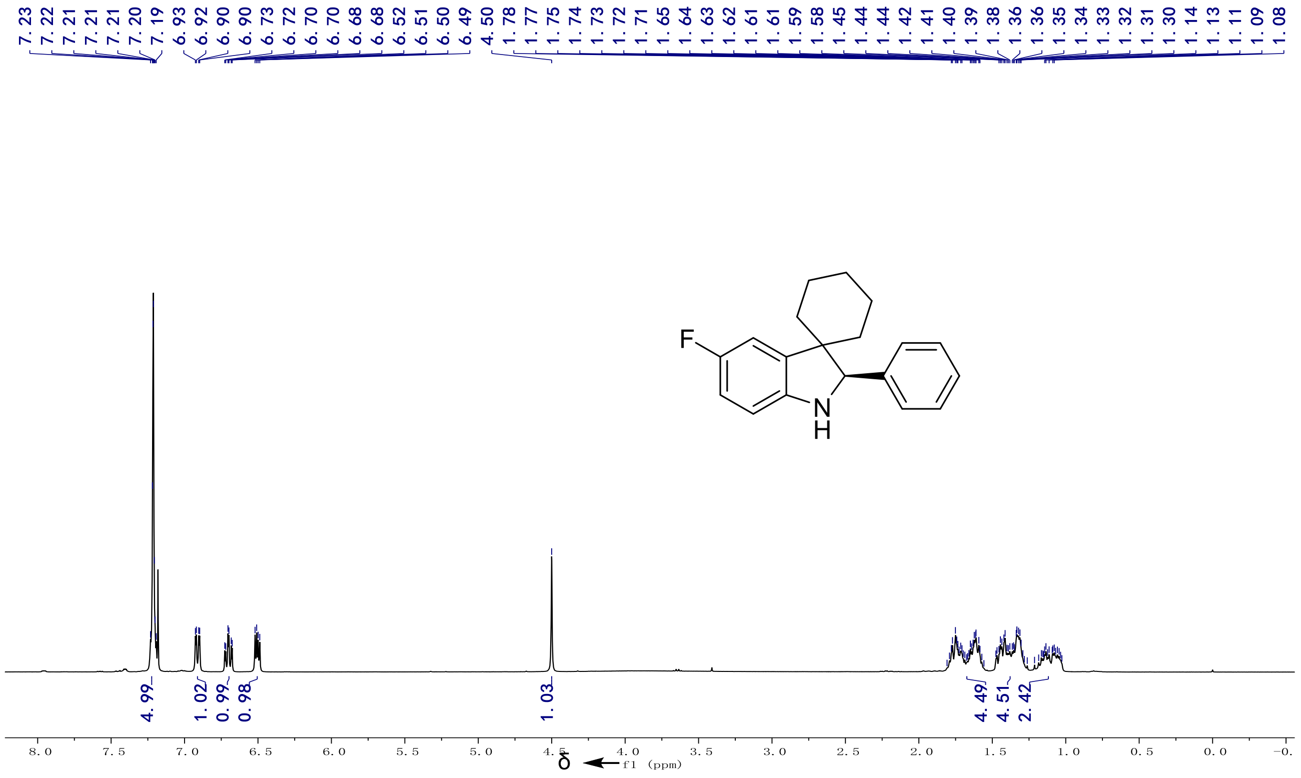

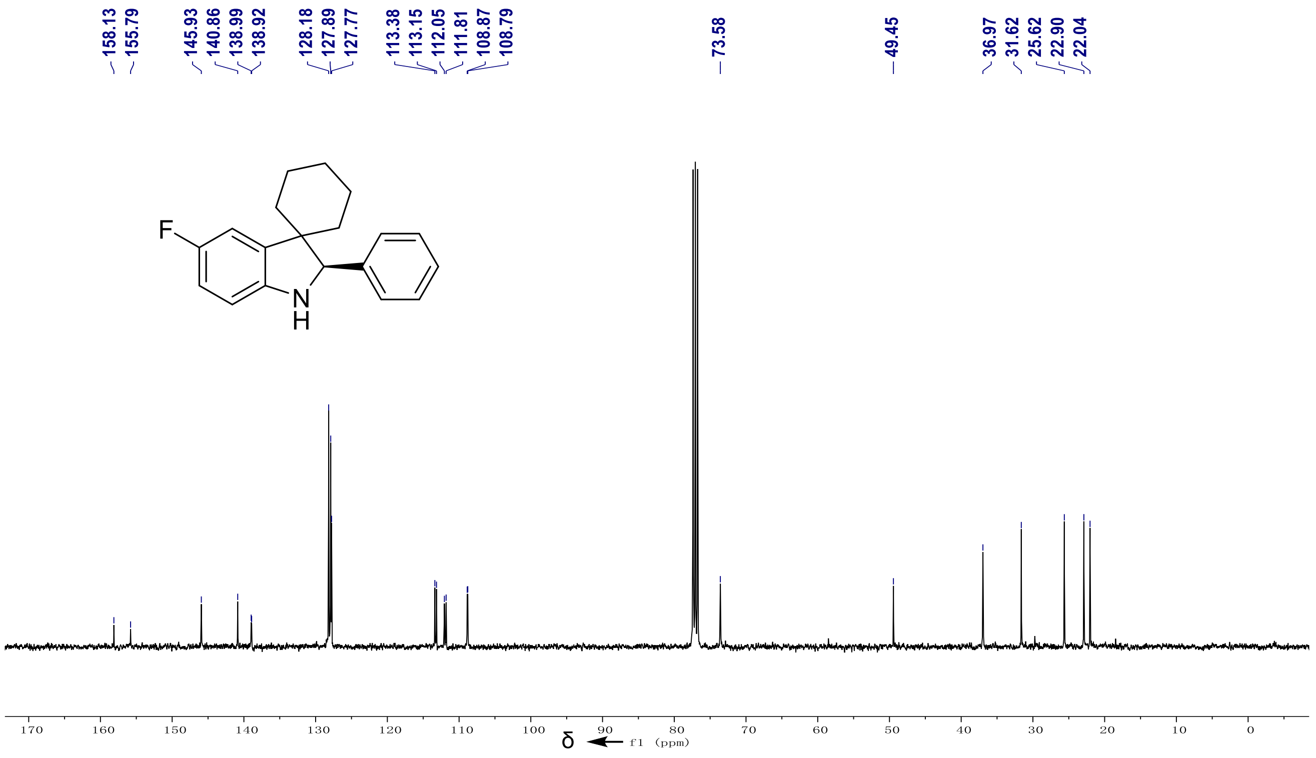

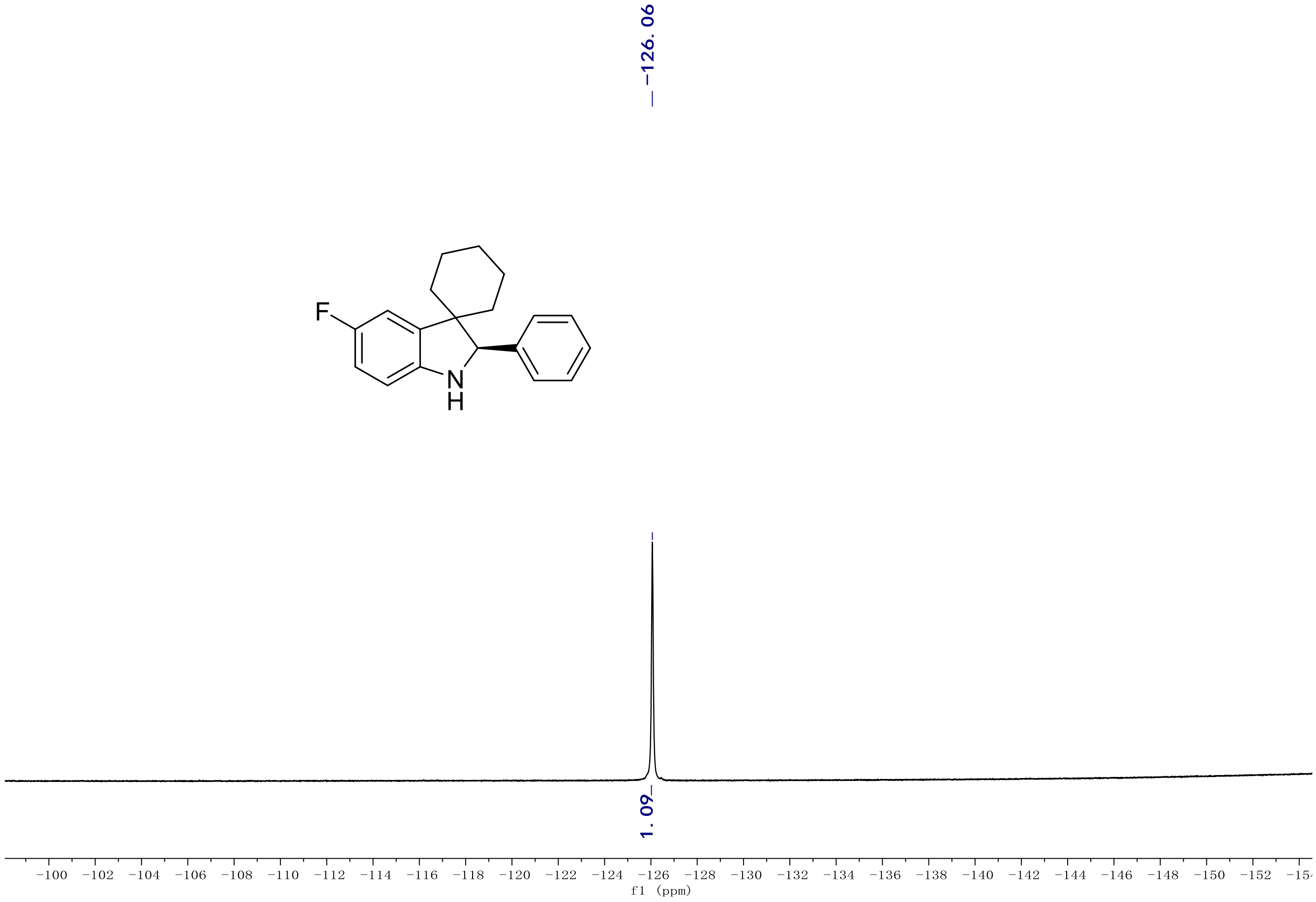

−126.06
F
N
H
1.09
−100 −102 −104 −106 −108 −110 −112 −114 −116 −118 −120 −122 −124 −126 −128 −130 −132 −134 −136 −138 −140 −142 −144 −146 −148 −150 −152
f1 (ppm)

(*R*)-1v: (R)-5'-chloro-2'-phenylspiro[cyclohexane-1,3'-indoline].

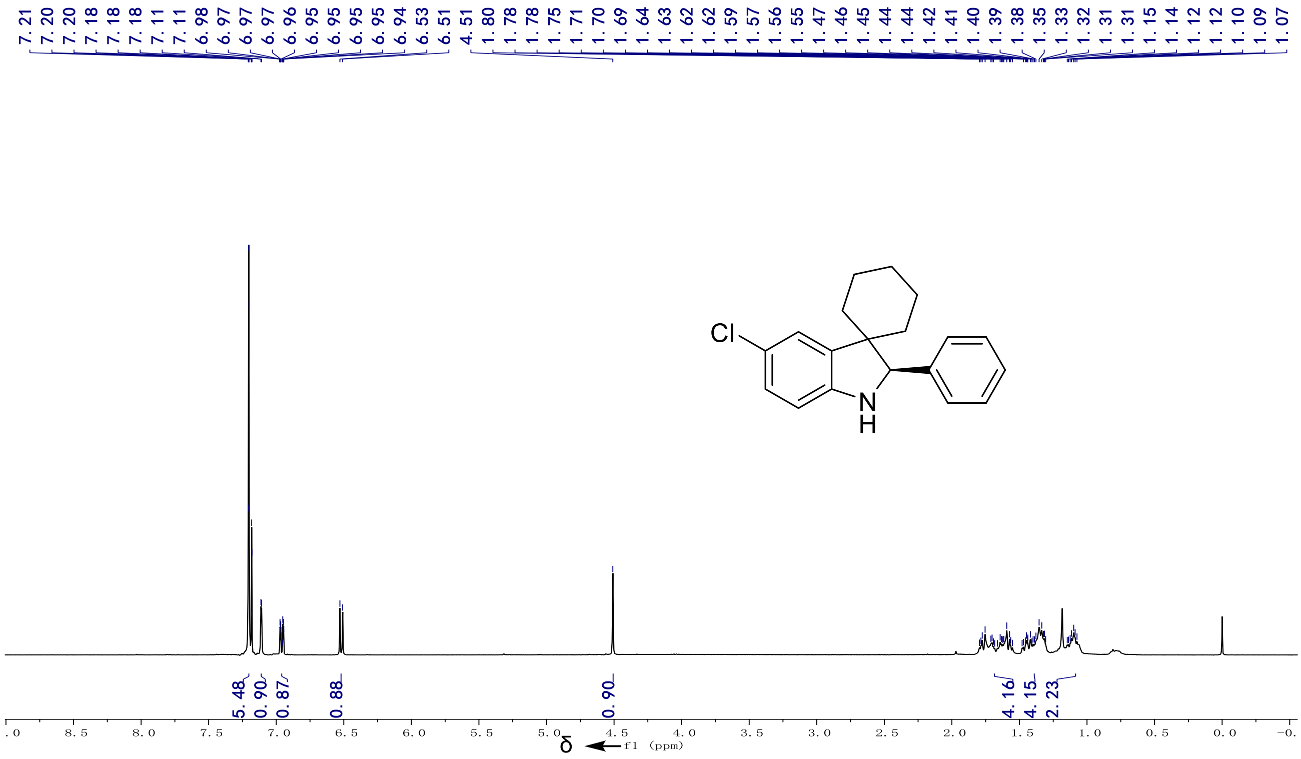

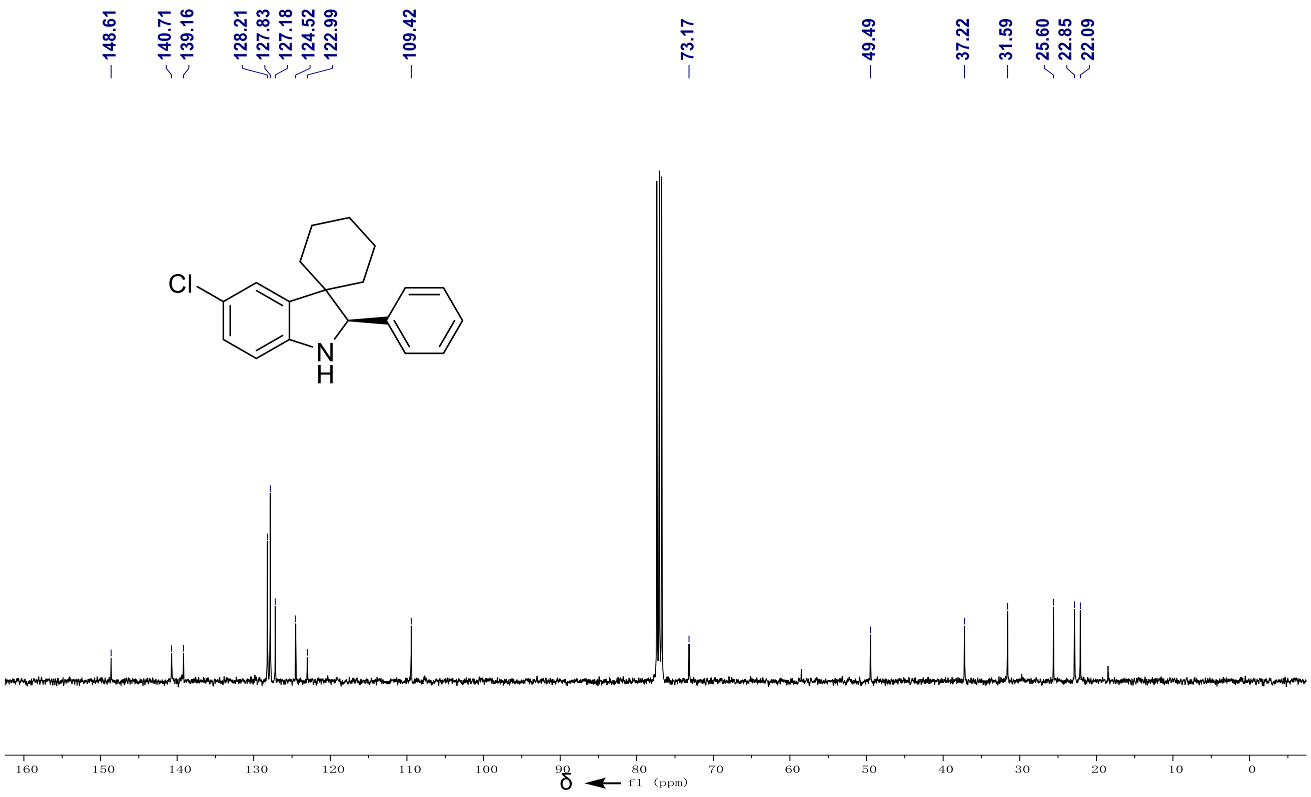

(*R*)-1w: (R)-5'-bromo-2'-phenylspiro[cyclohexane-1,3'-indoline].

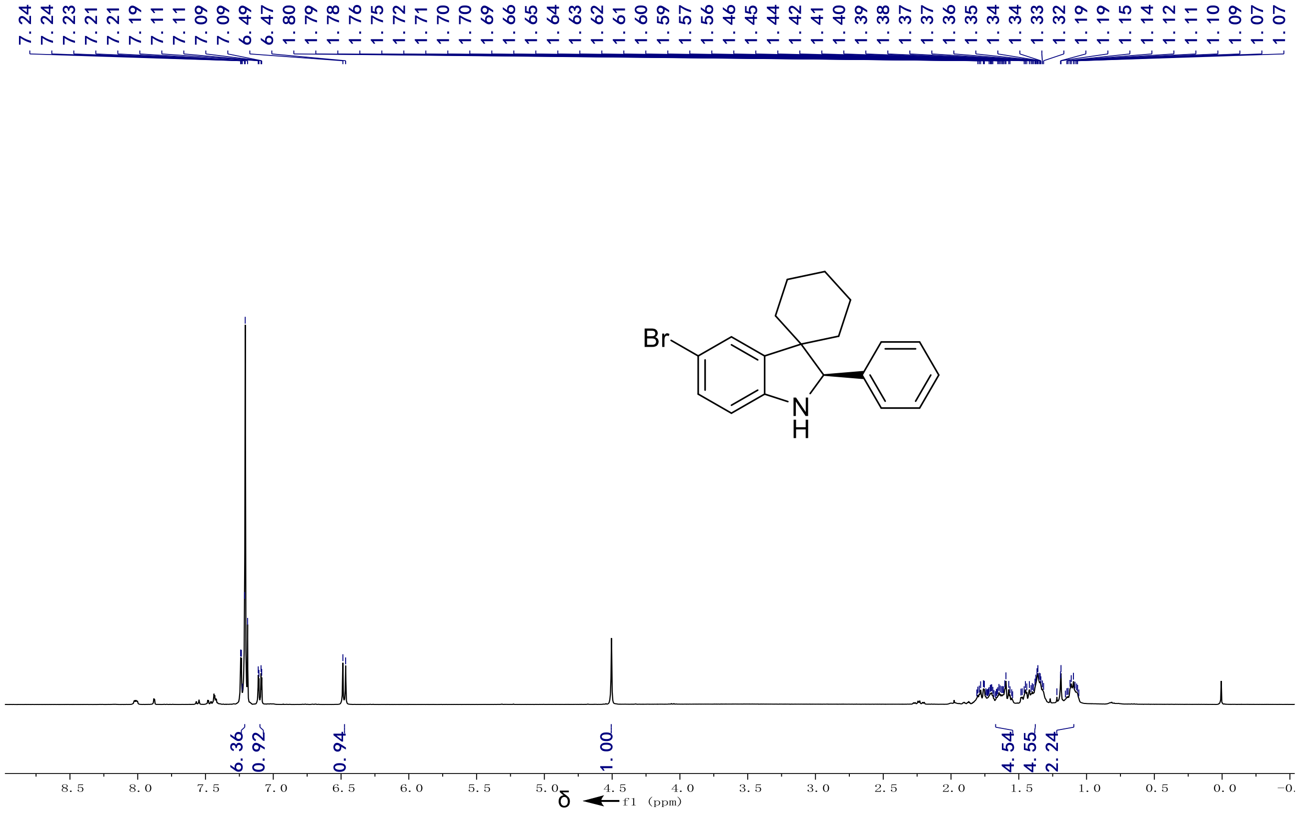

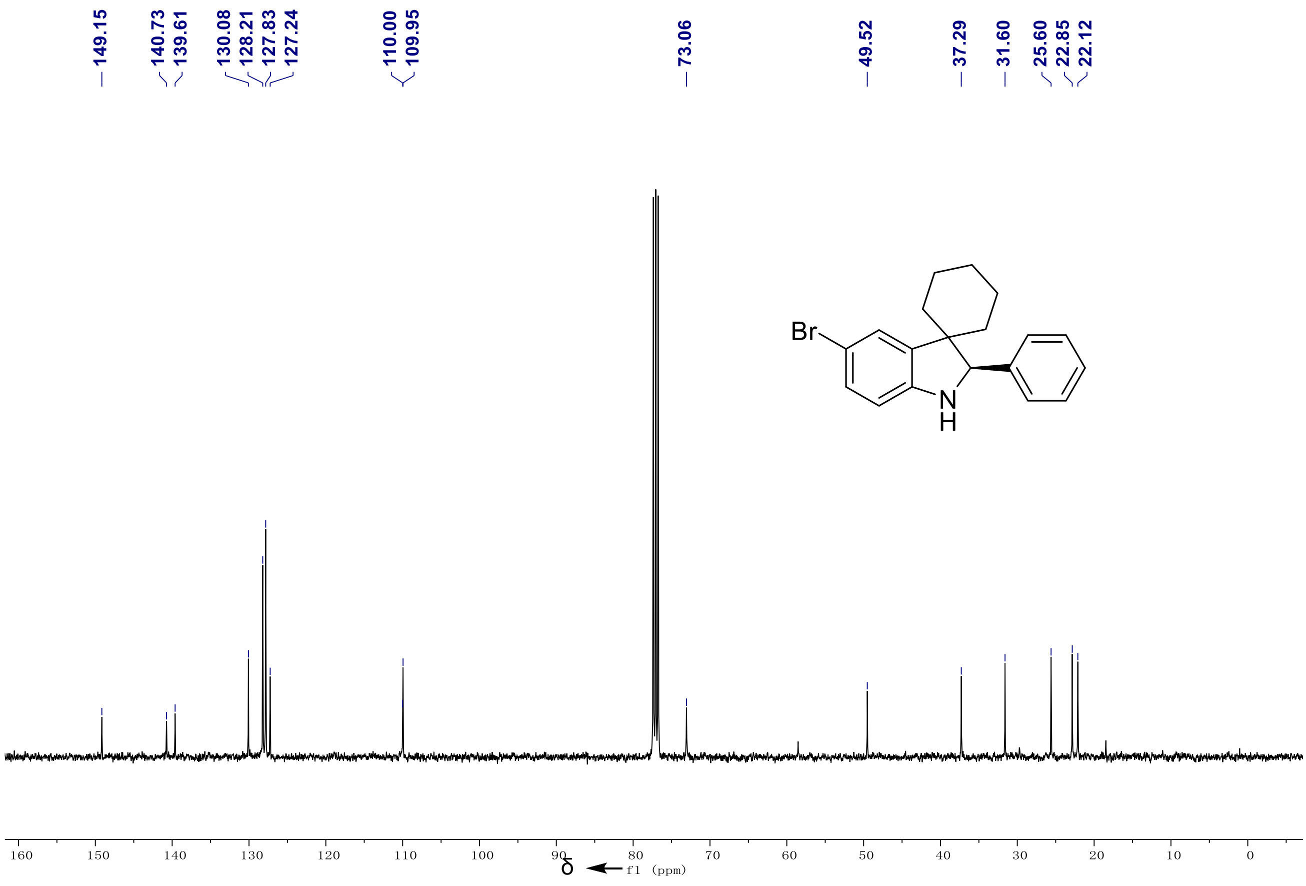

(*R*)-1x：(R)-6'-bromo-2'-phenylspiro[cyclohexane-1,3'-indoline]

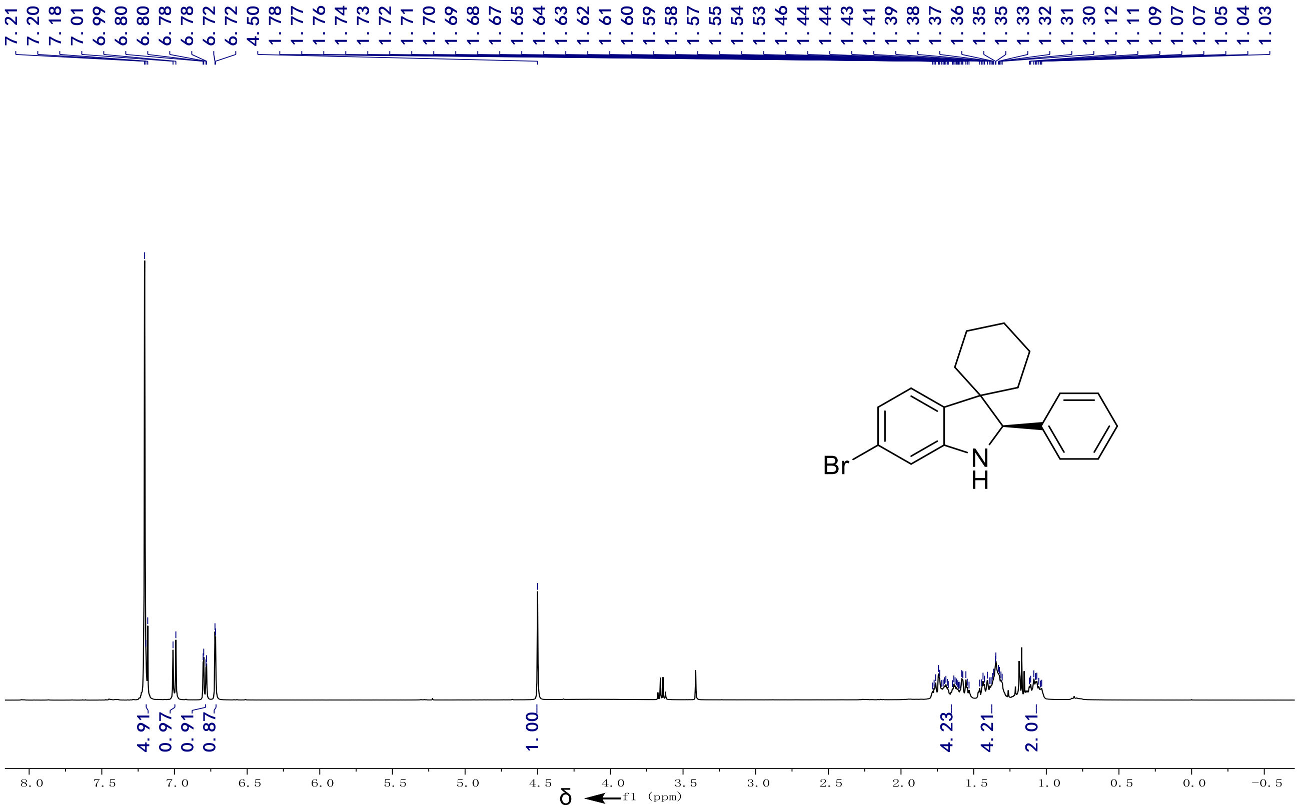

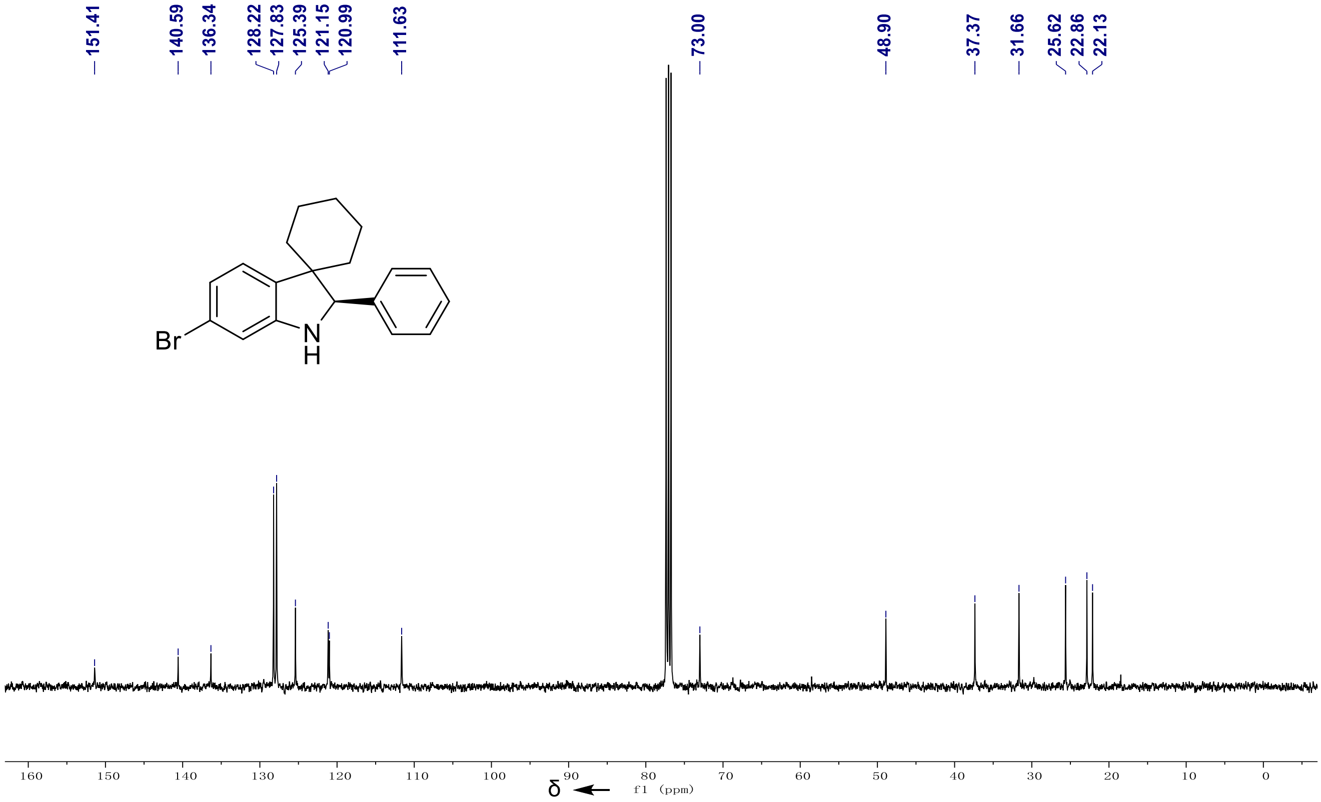

(*R*)-1y：(R)-2'-phenyl-5'-(trifluoromethyl)spiro[cyclohexane-1,3'-indoline]

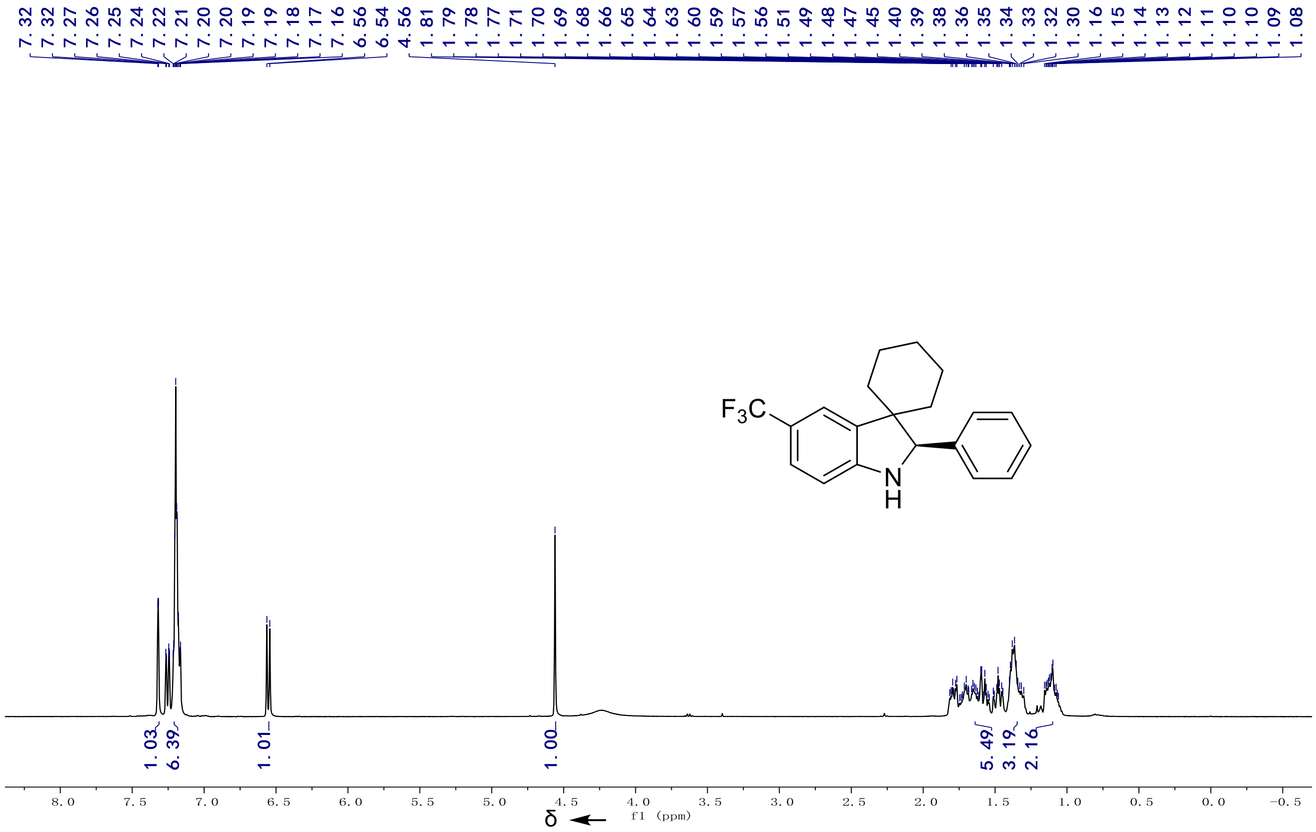

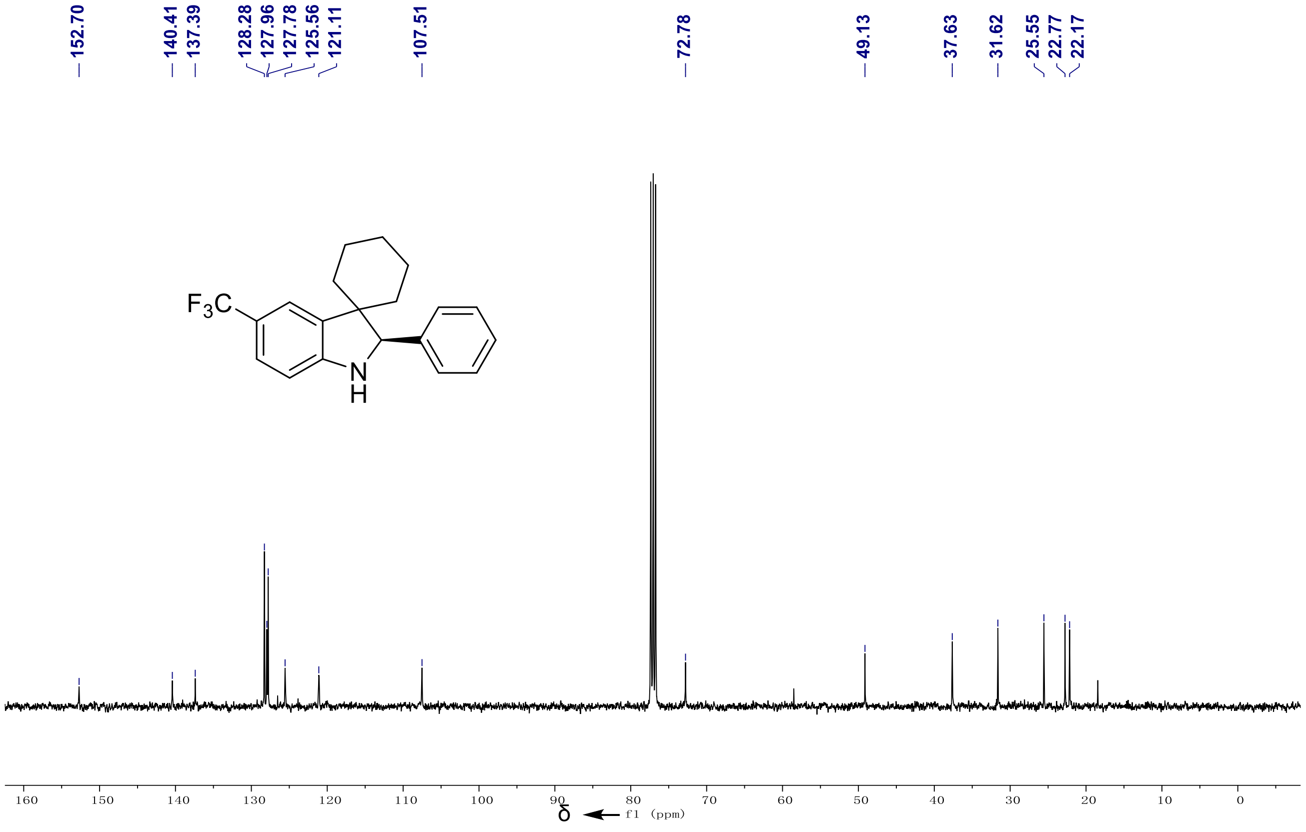

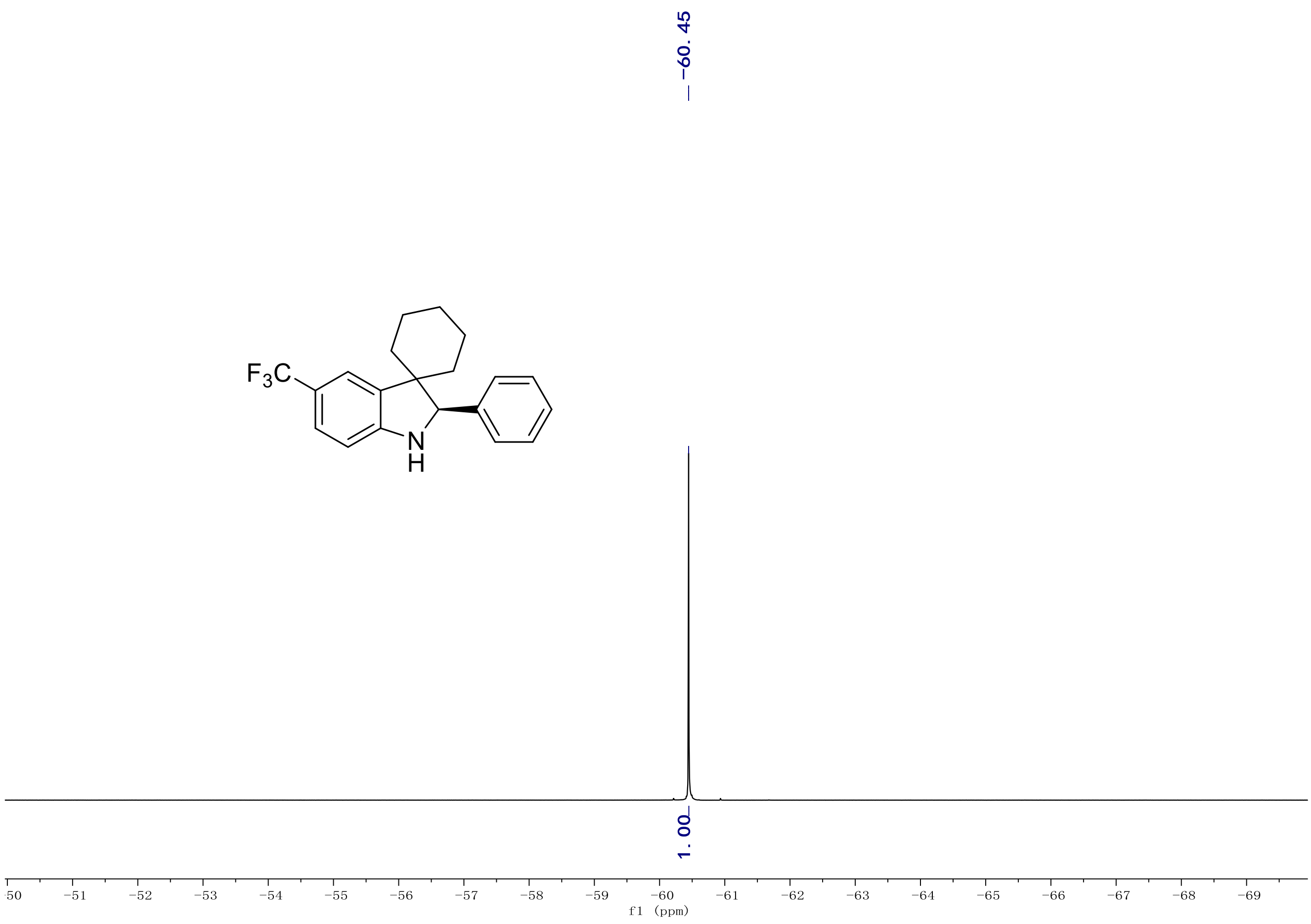
−60.45
F3C
N
H
1.00
50 −51 −52 −53 −54 −55 −56 −57 −58 −59 −60 −61 −62 −63 −64 −65 −66 −67 −68 −69
f1 (ppm)

(*R*)-1z：(R)-2'-phenyl-5'-(trifluoromethoxy)spiro[cyclohexane-1,3'-indoline]

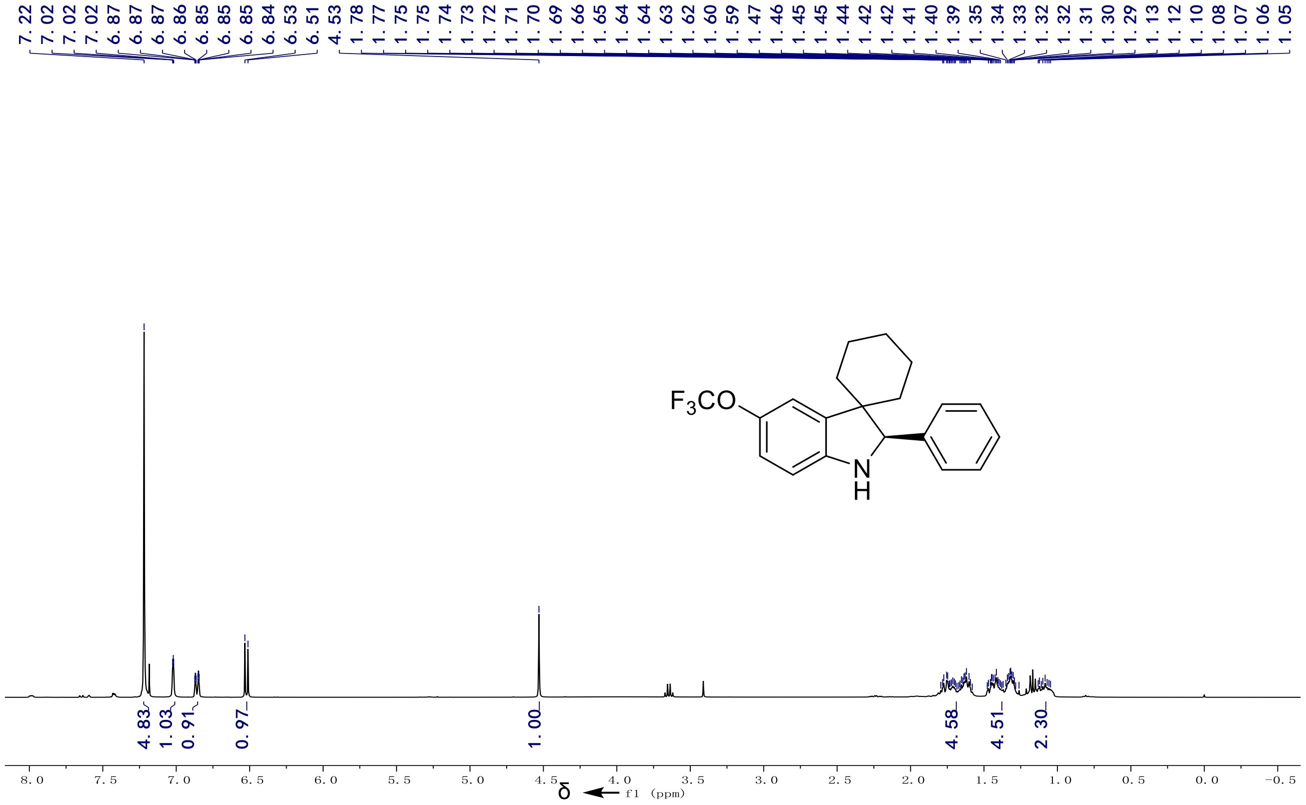

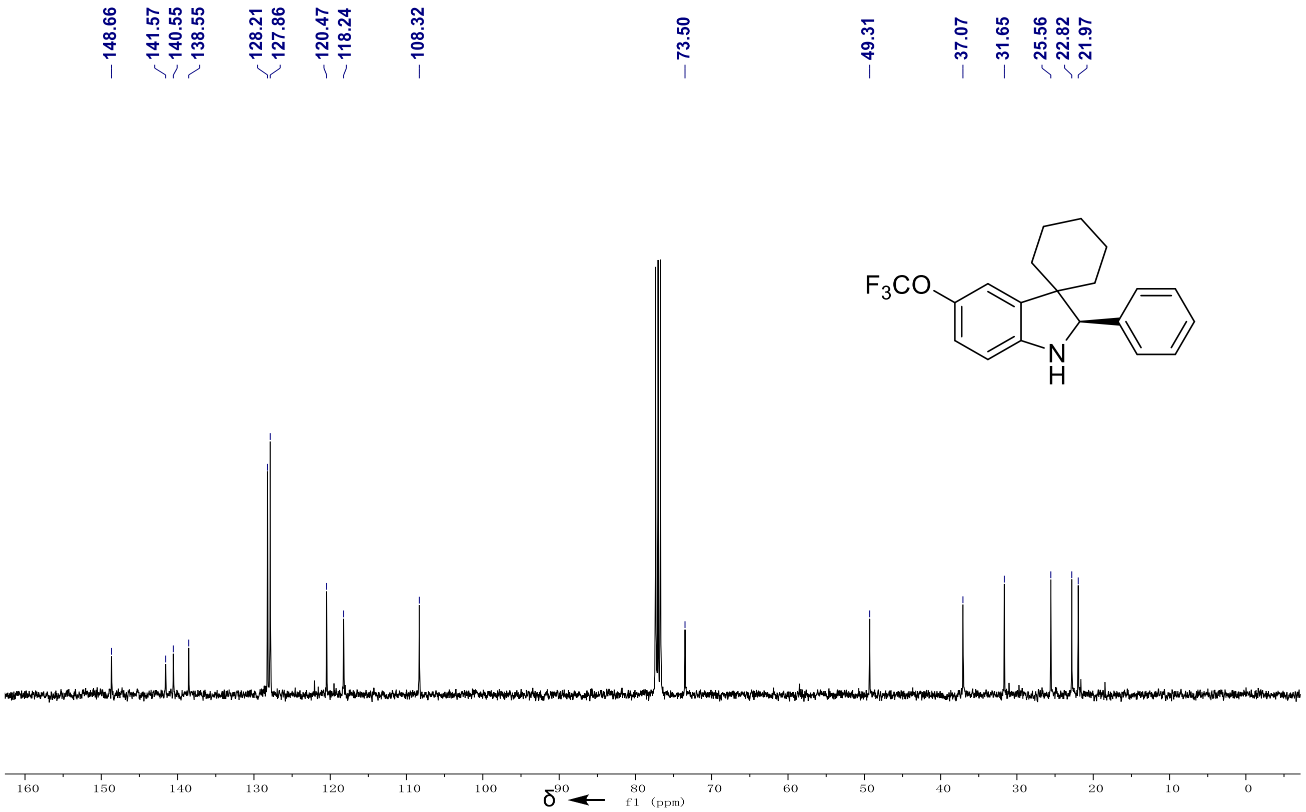

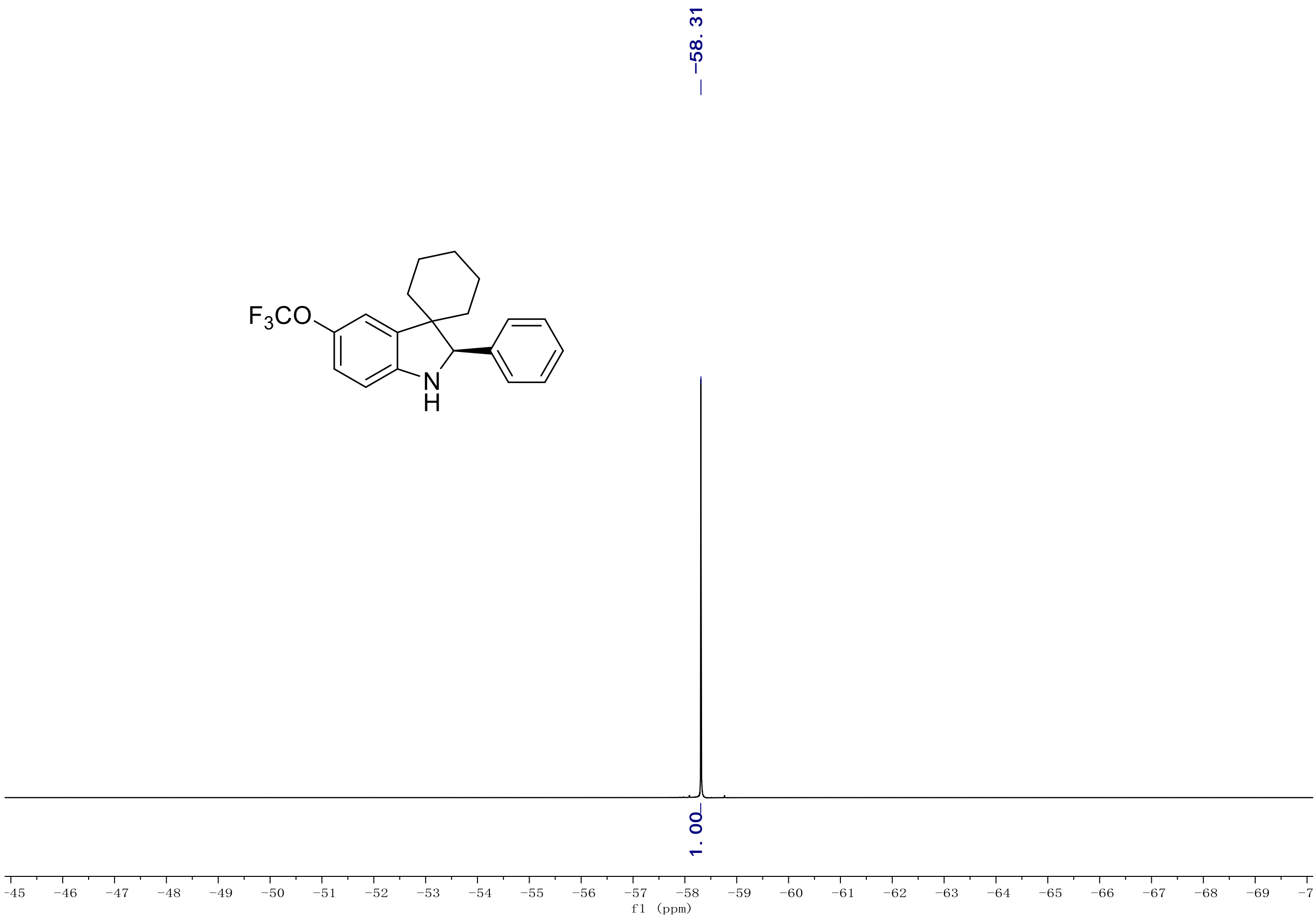
−58.31
F3CO
N
H
1.00
-45 −46 −47 −48 −49 −50 −51 −52 −53 −54 −55 −56 −57 −58 −59 −60 −61 −62 −63 −64 −65 −66 −67 −68 −69 −7
f1 (ppm)

(*R*)-1z': (R)- 5'-methoxy-2'-phenylspiro[cyclohexane-1,3'-indoline].

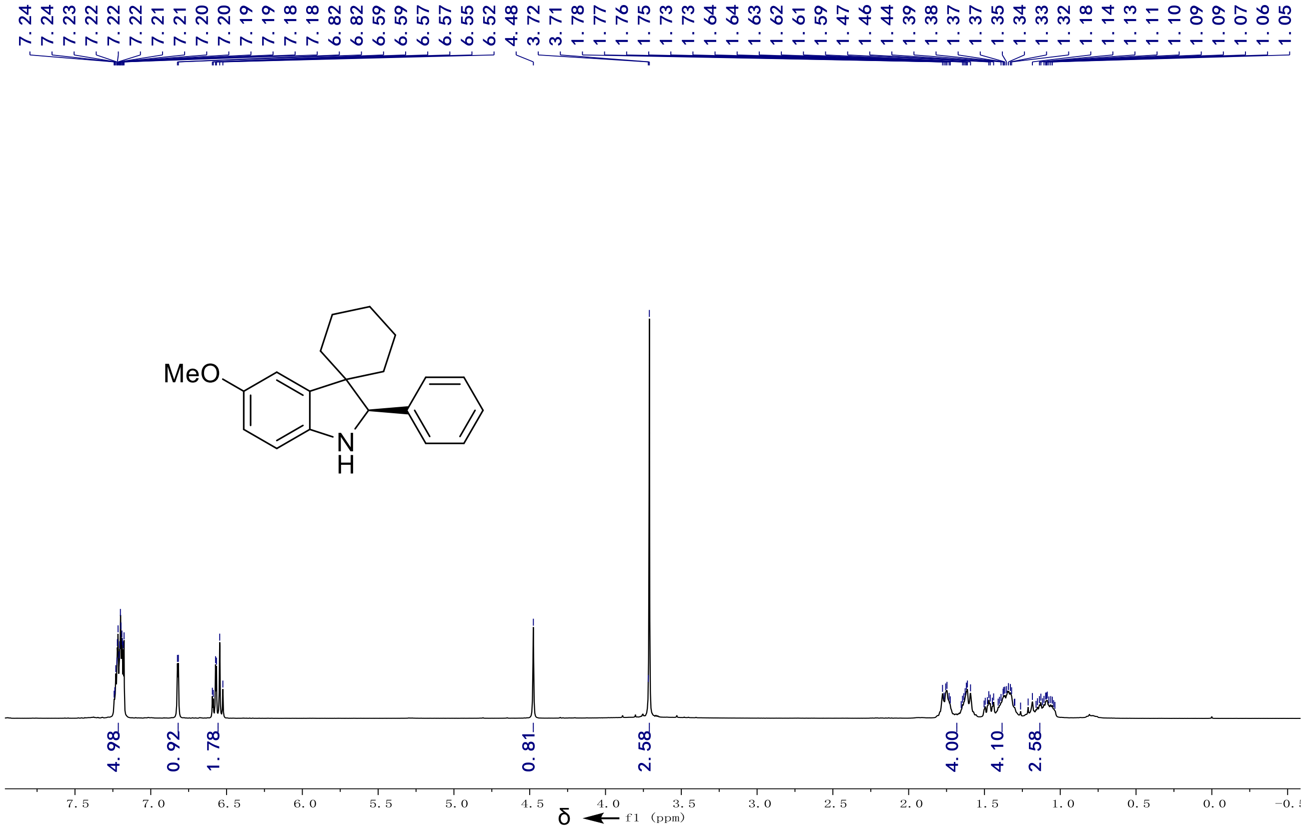

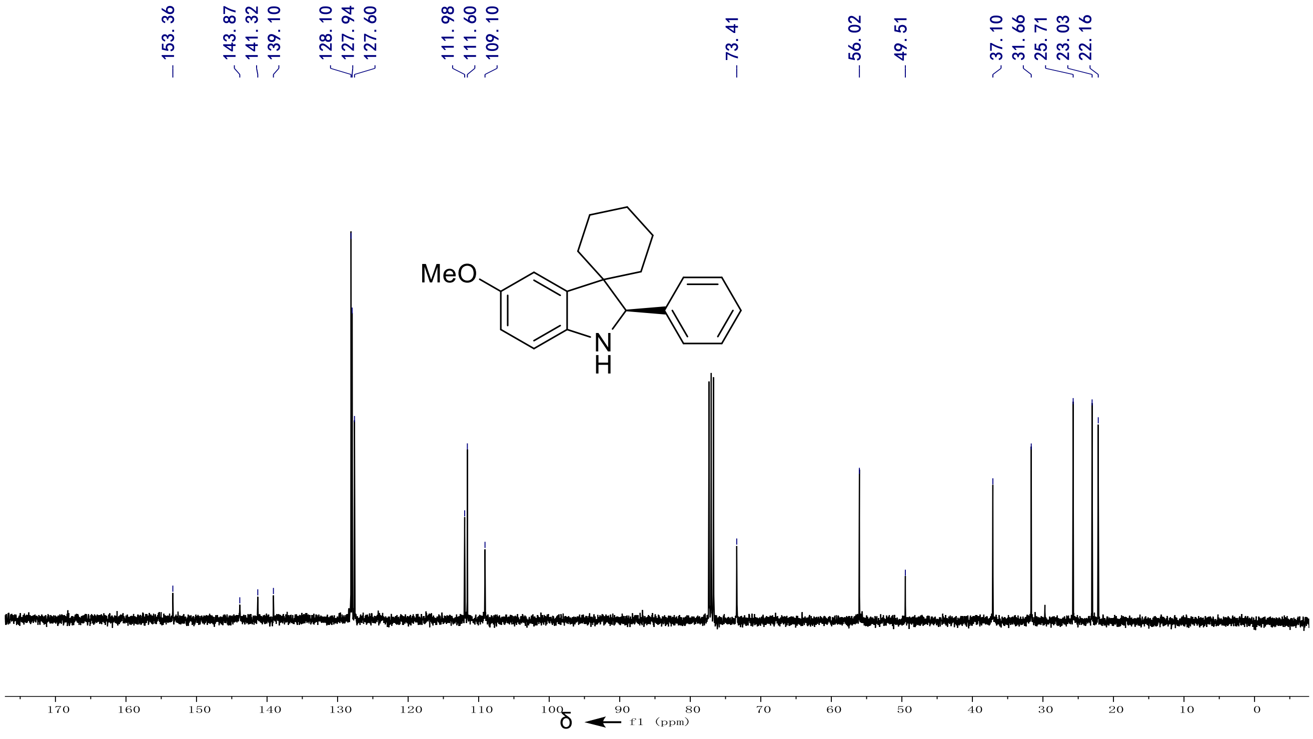

(*S*)-3a: (S)-2-phenyl-1,2,3,4-tetrahydroquinoline.

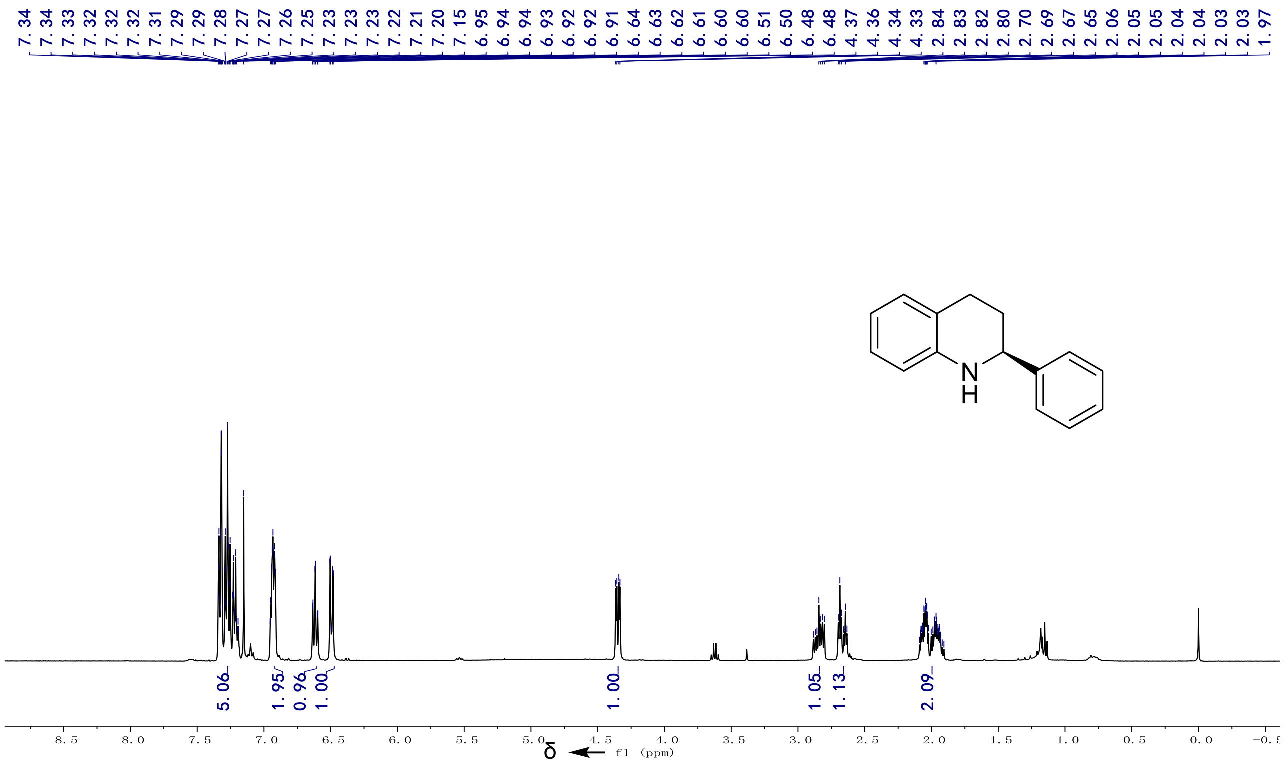

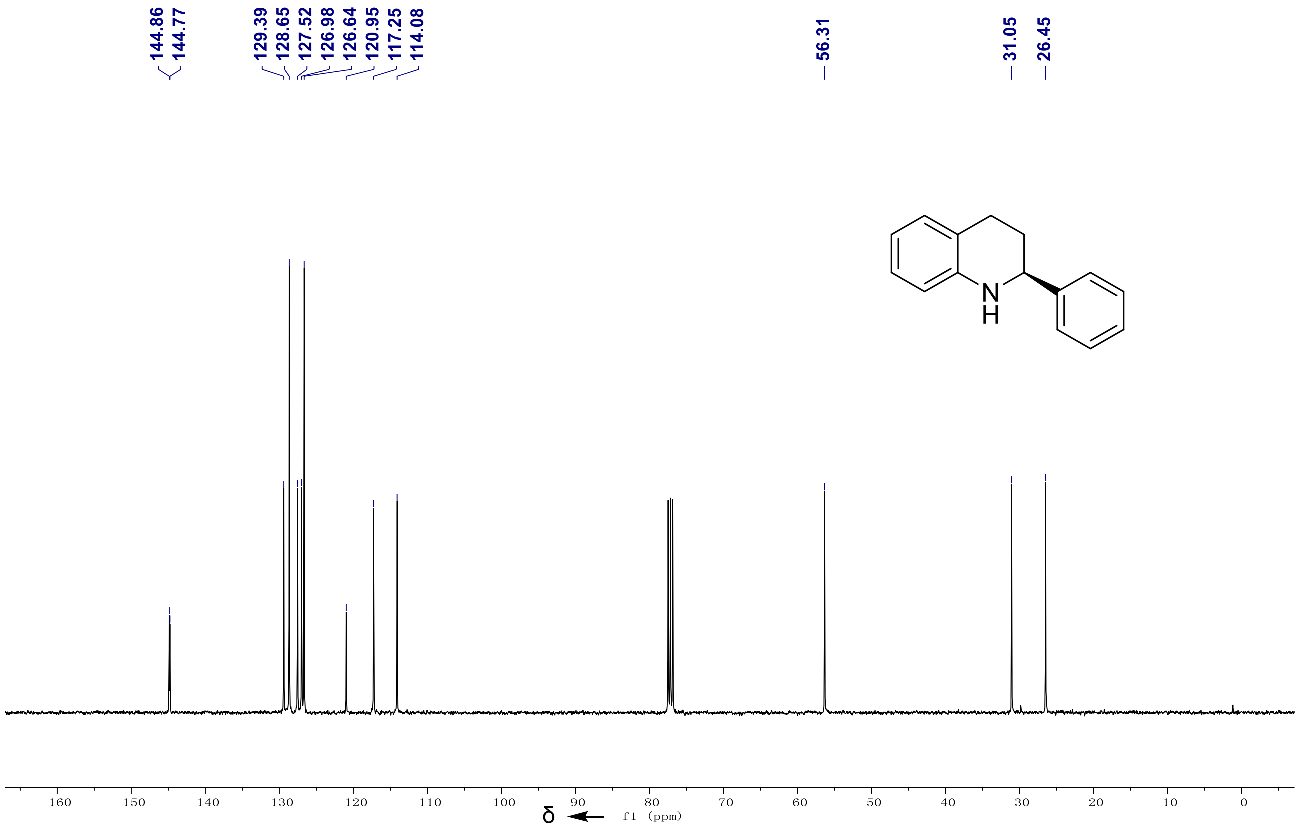

(*S*)-3b: (S)-2-(4-fluorophenyl)-1,2,3,4-tetrahydroquinoline.

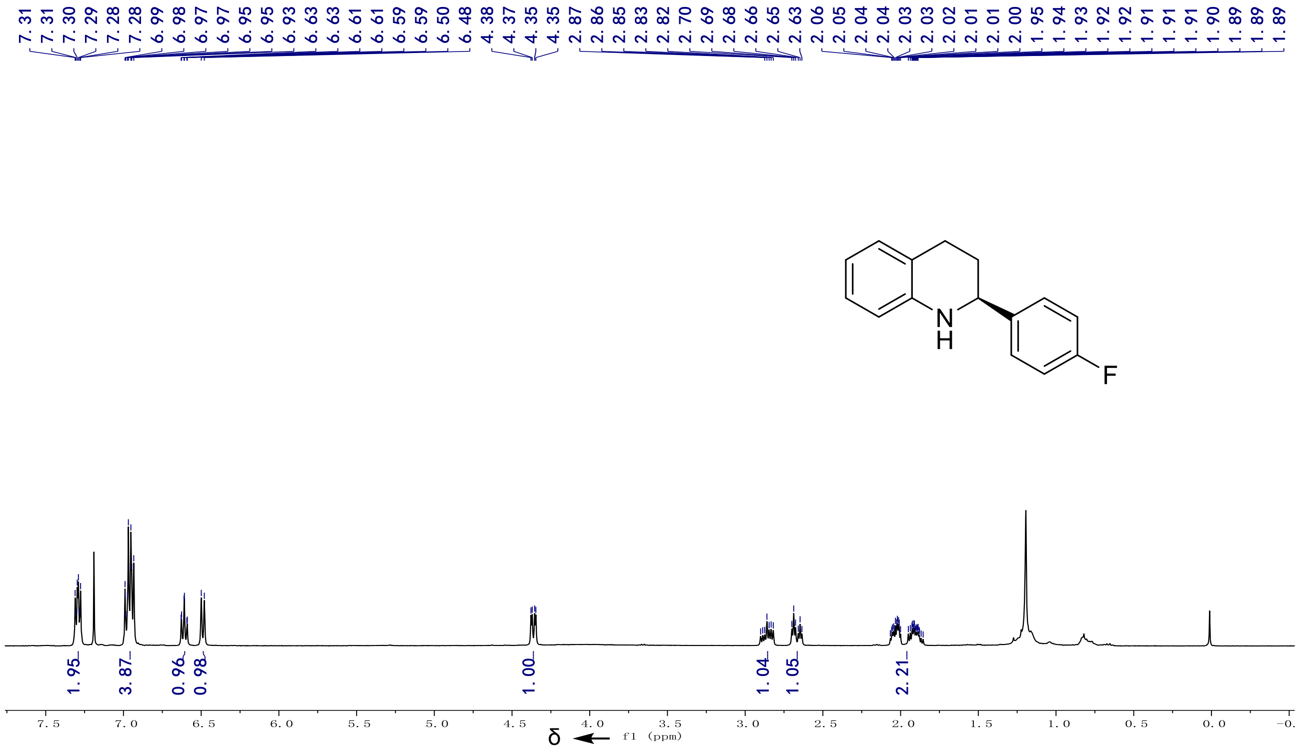

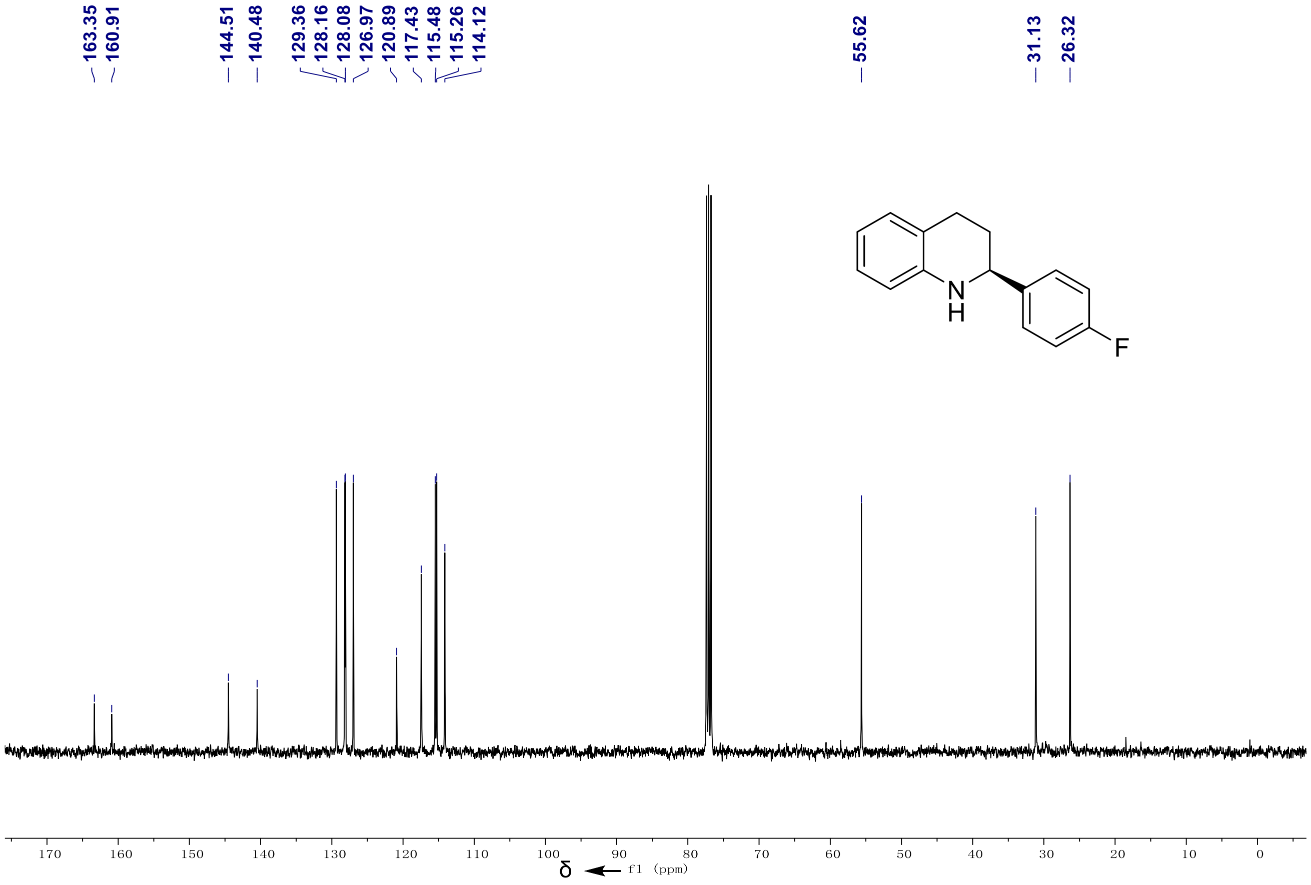

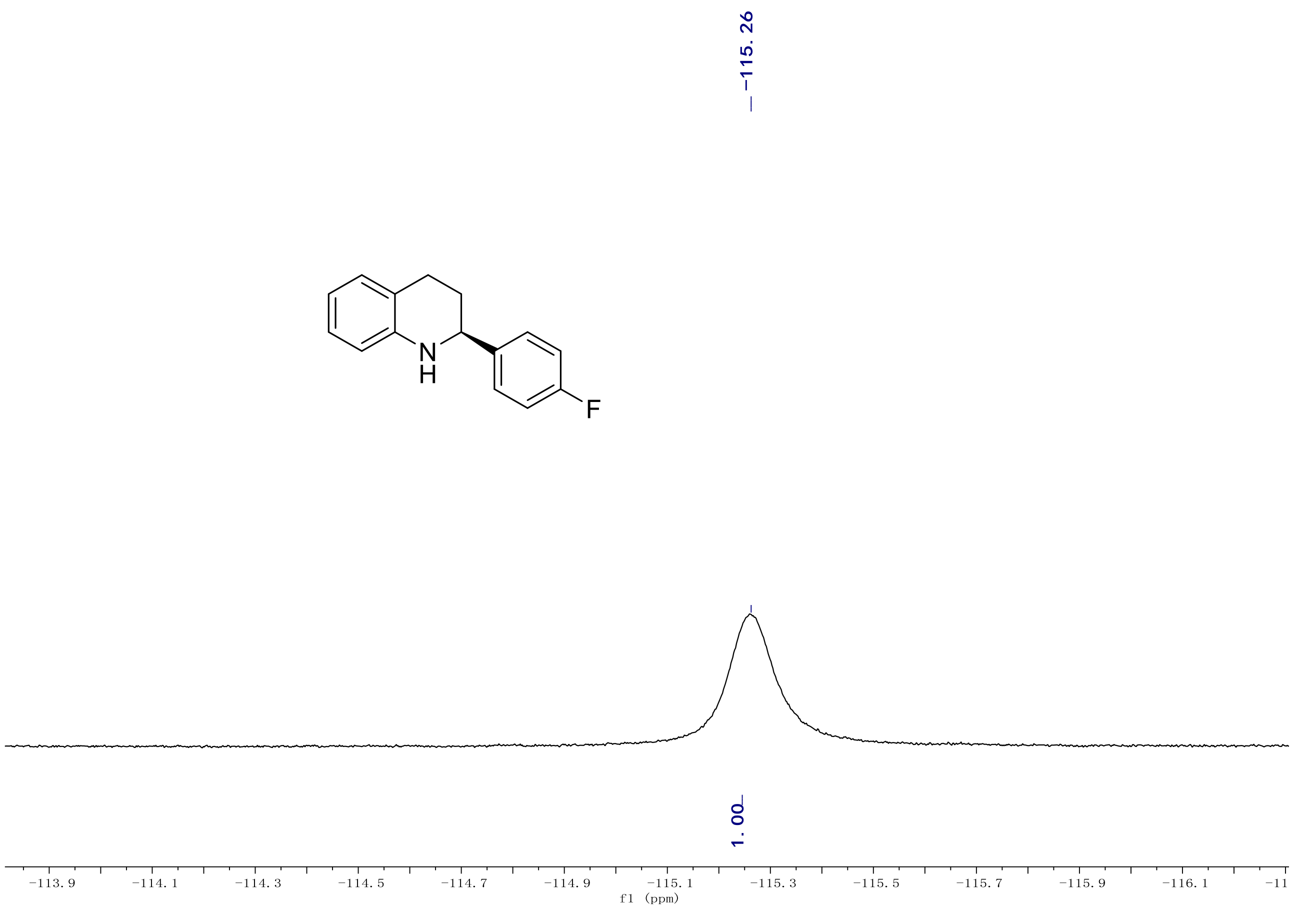

—115.26
1.00
-113.9
-114.1
-114.3
-114.5
-114.7
-114.9
-115.1
-115.3
-115.5
-115.7
-115.9
-116.1
f1 (ppm)

(*S*)-3c: (S)-2-(4-(trifluoromethyl)phenyl)-1,2,3,4-tetrahydroquinoline.

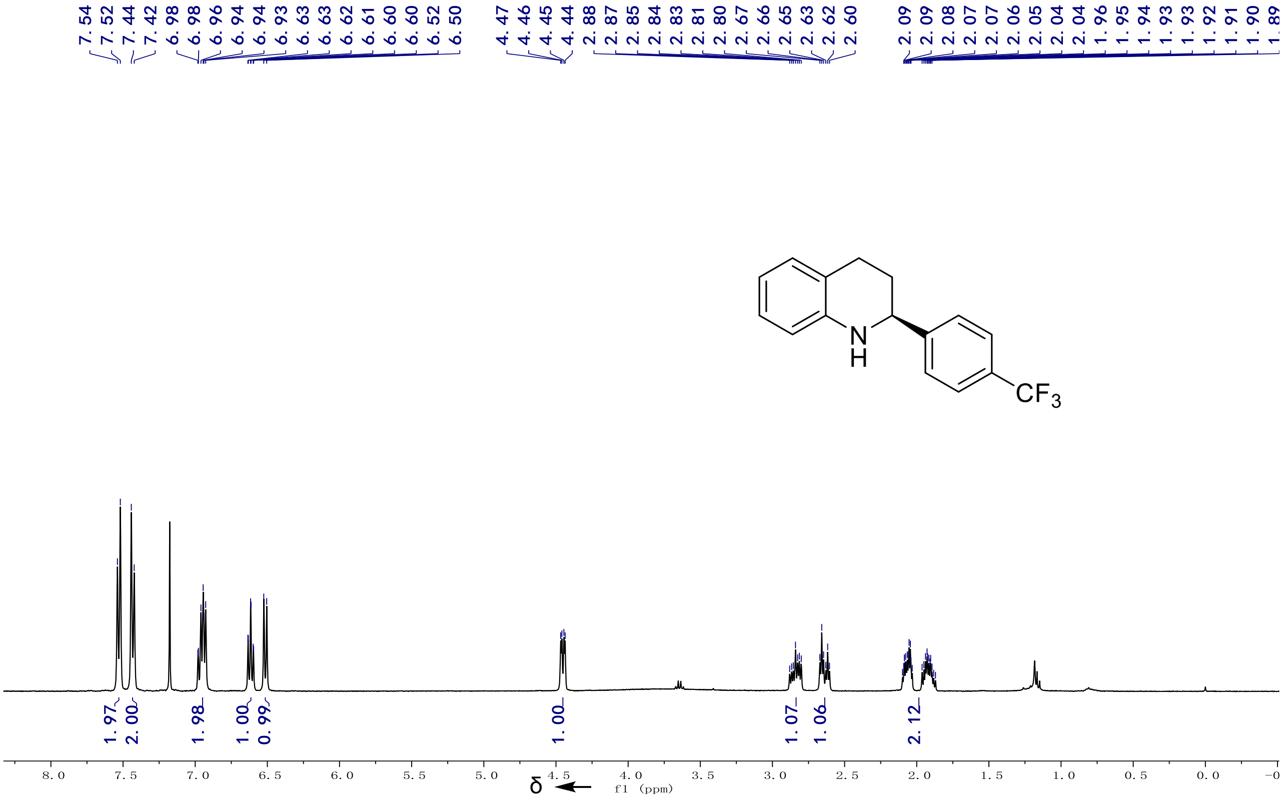

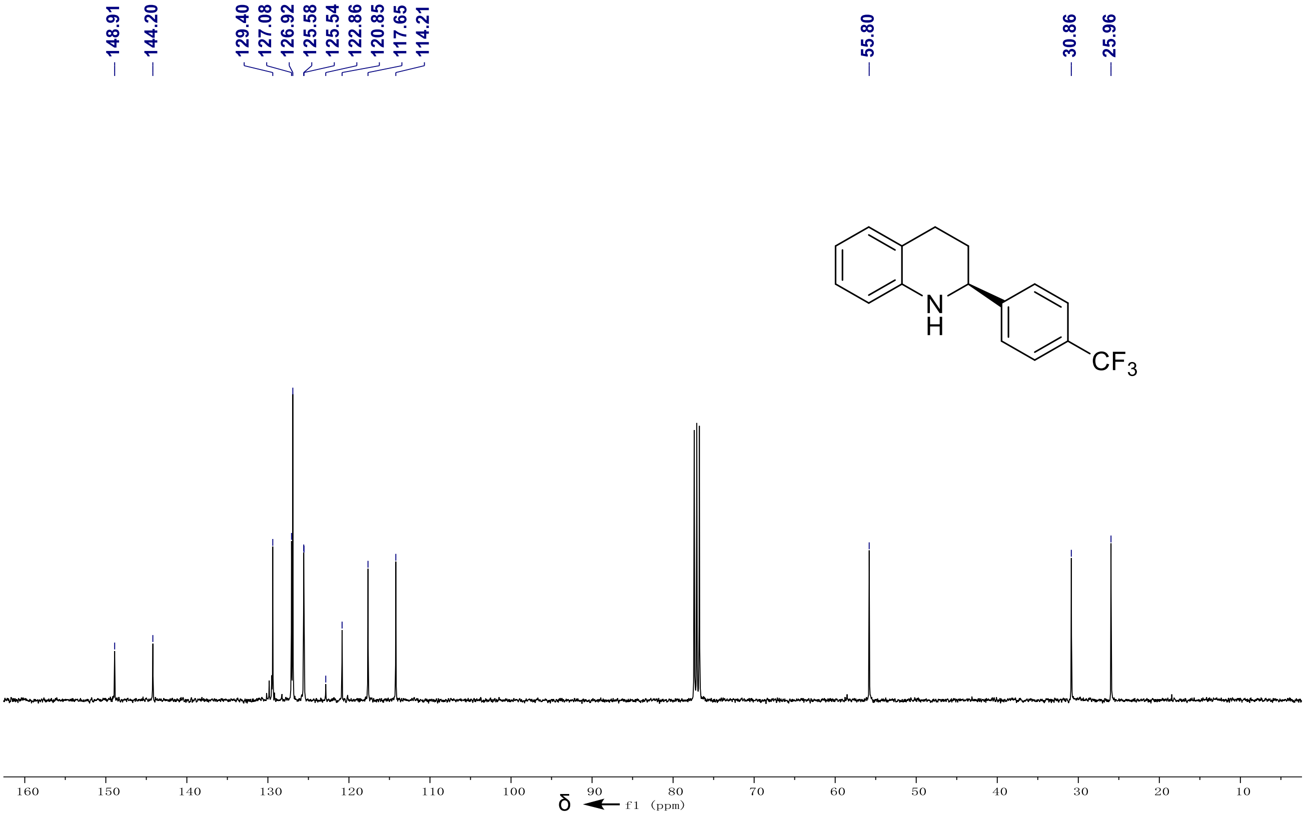

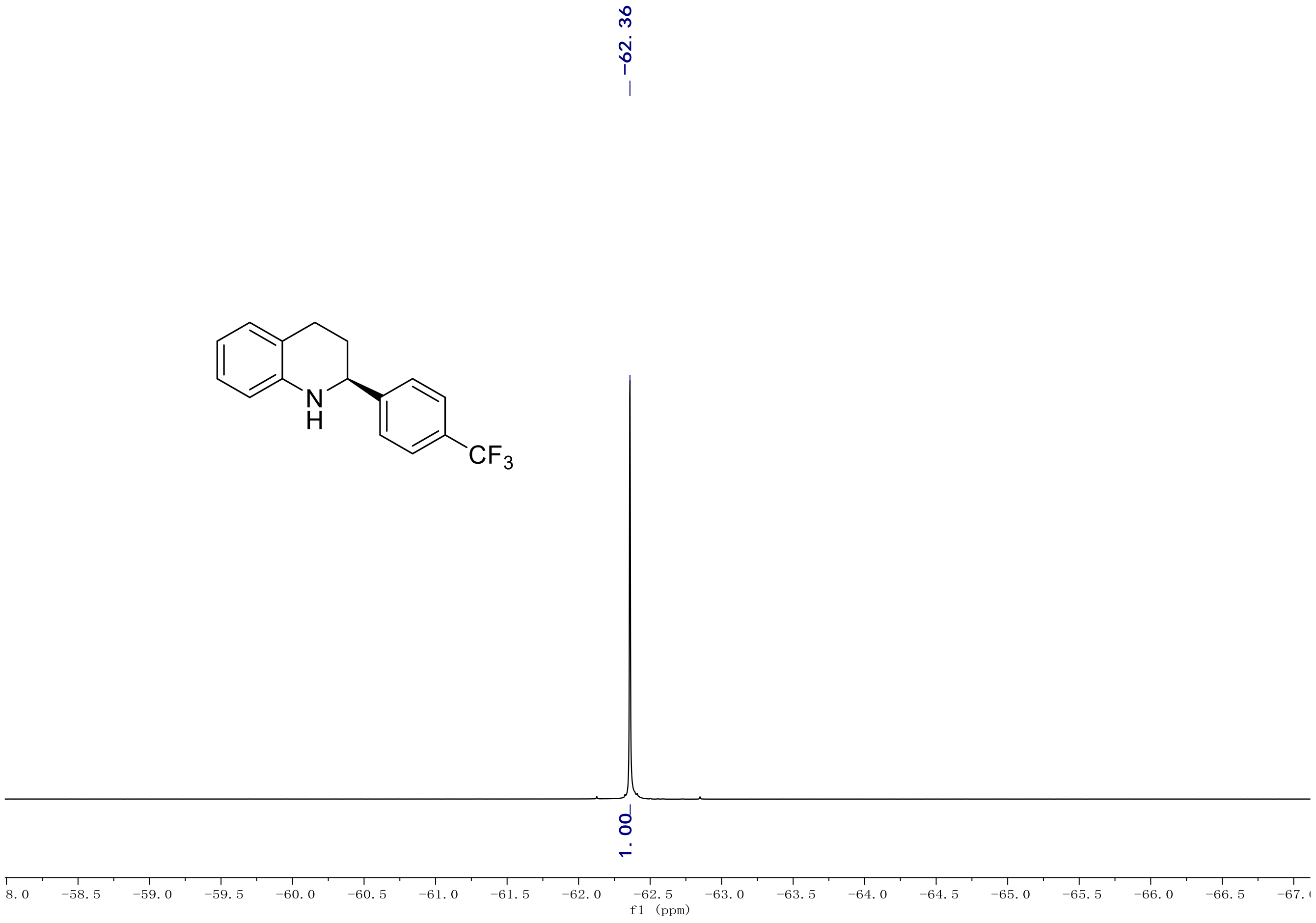

-62.36
1.00
8.0
-58.5
-59.0
-59.5
-60.0
-60.5
-61.0
-61.5
-62.0
-62.5
-63.0
-63.5
-64.0
-64.5
-65.0
-65.5
-66.0
-66.5
-67.
f1 (ppm)

(*S*)-3d: (S)-4-(1,2,3,4-tetrahydroquinolin-2-yl)benzonitrile.

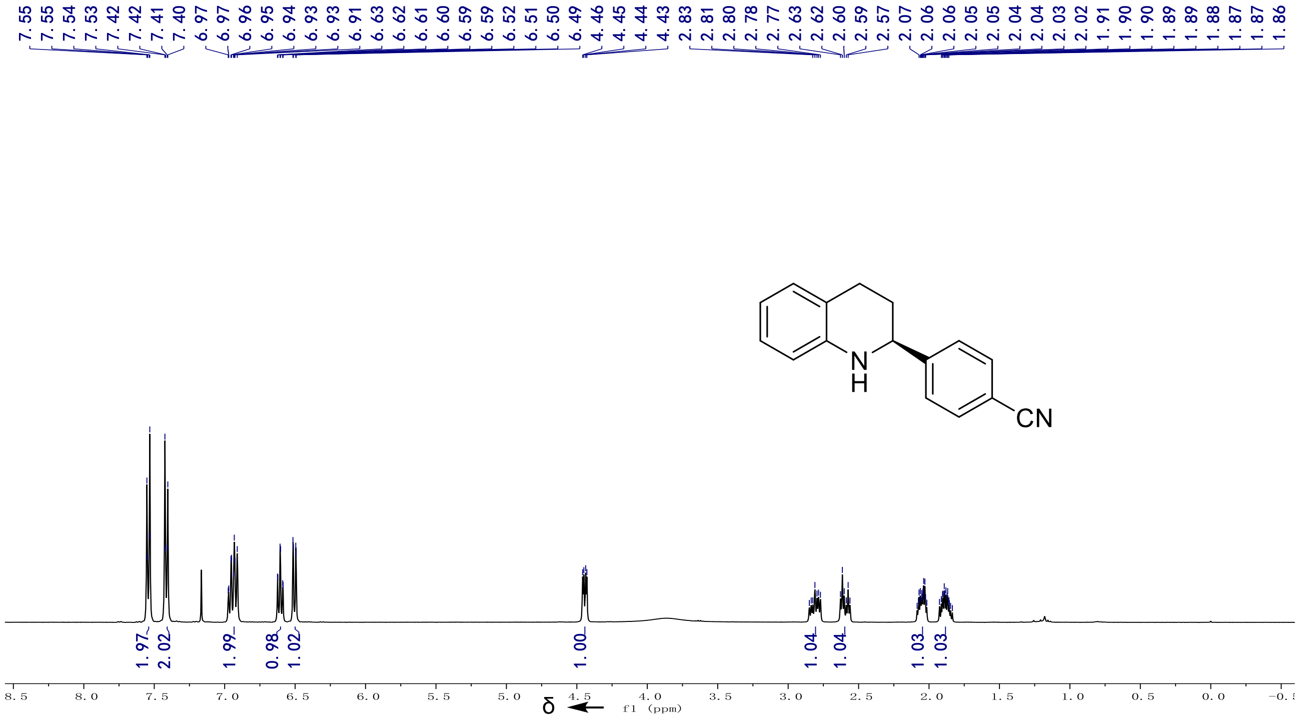

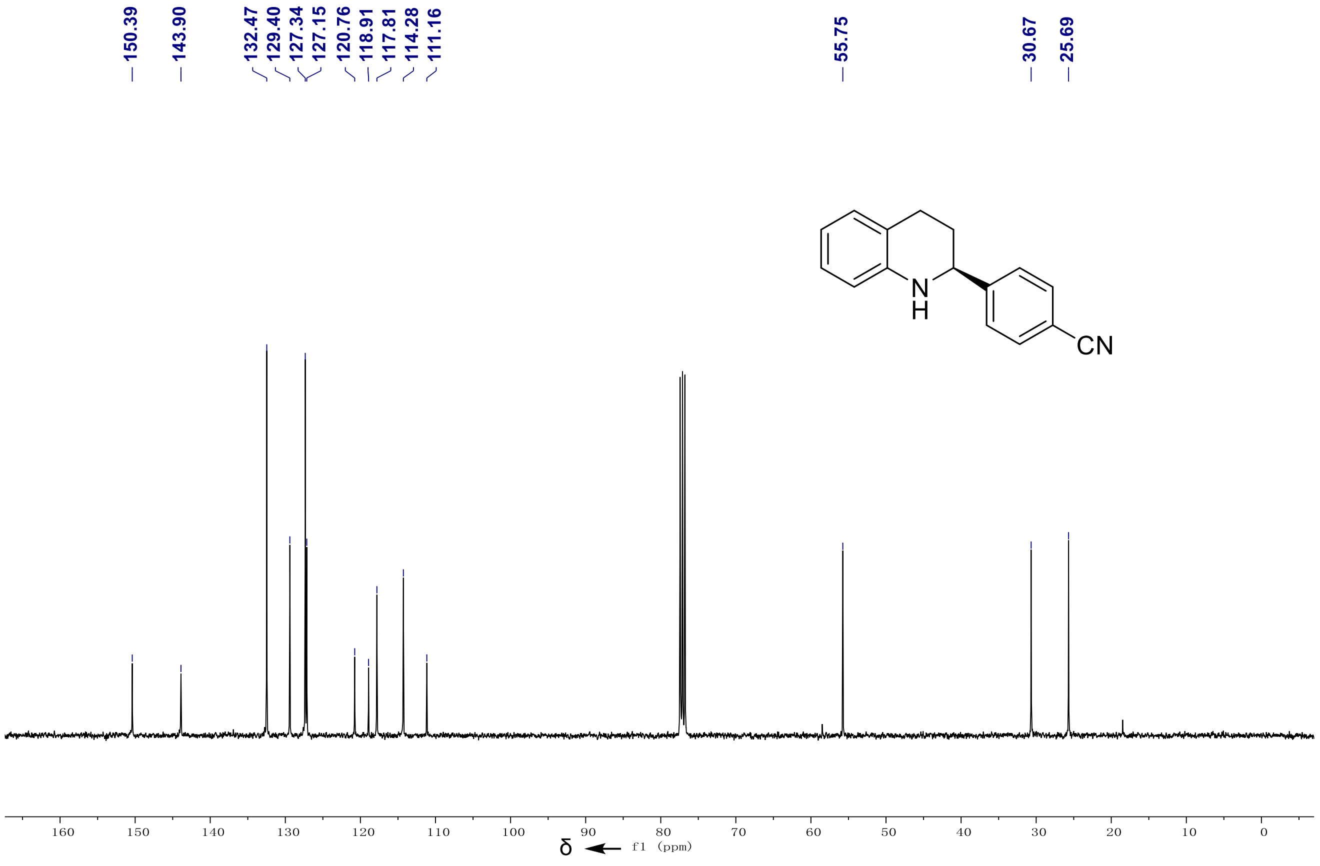

(*S*)-3e: (S)-2-(3-nitrophenyl)-1,2,3,4-tetrahydroquinoline.

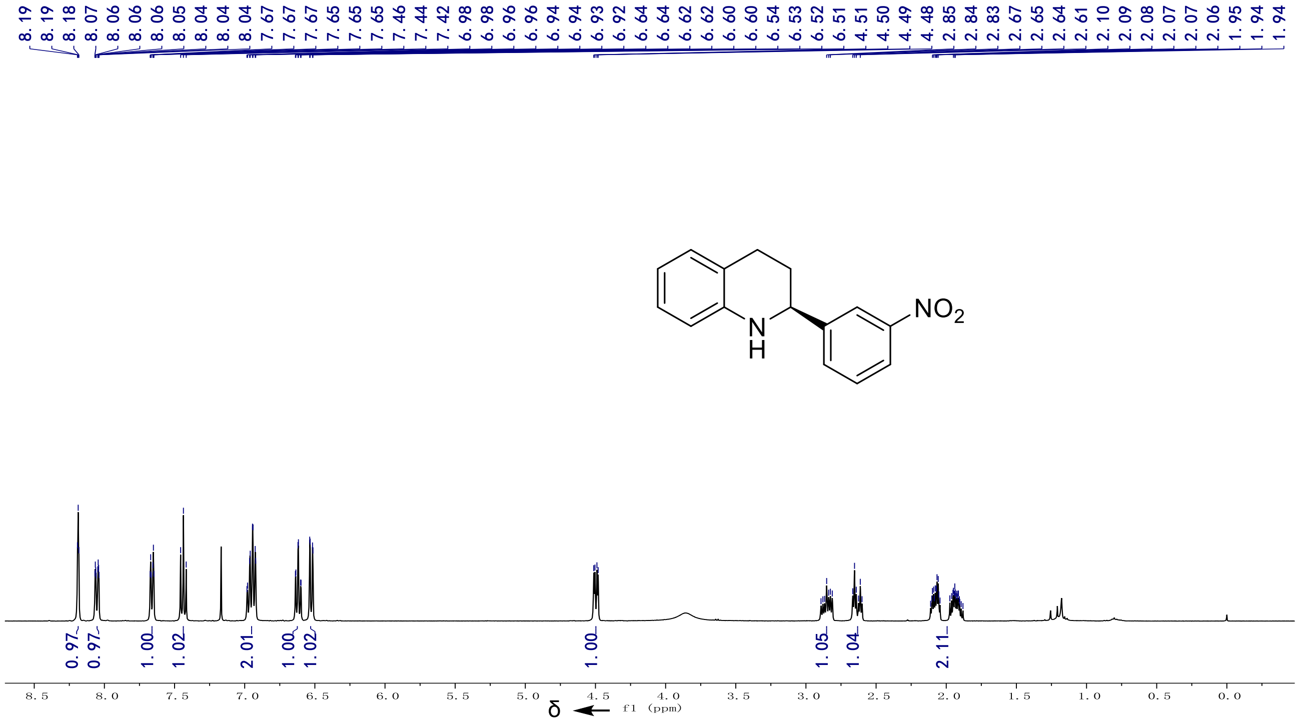

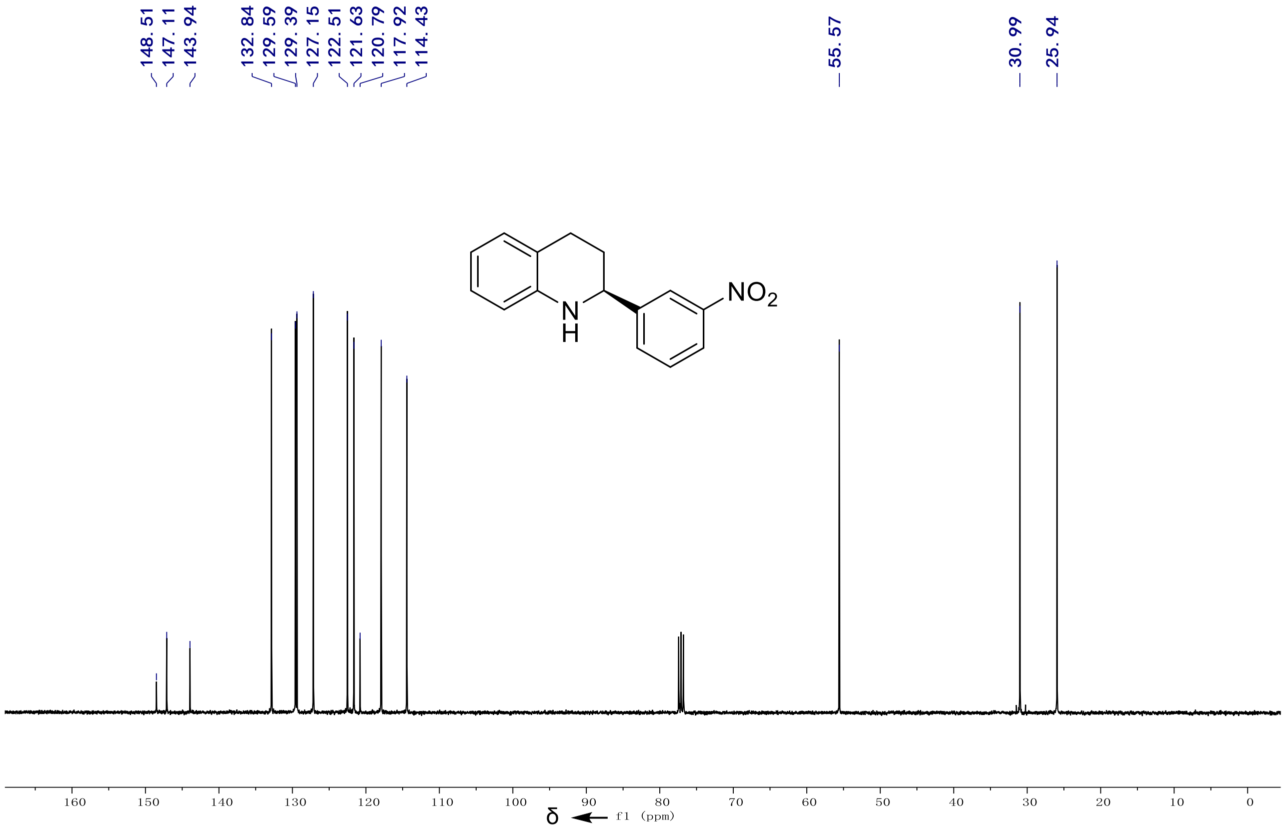

(*S*)-3f：methyl (S)-4-(1,2,3,4-tetrahydroquinolin-2-yl)benzoate.

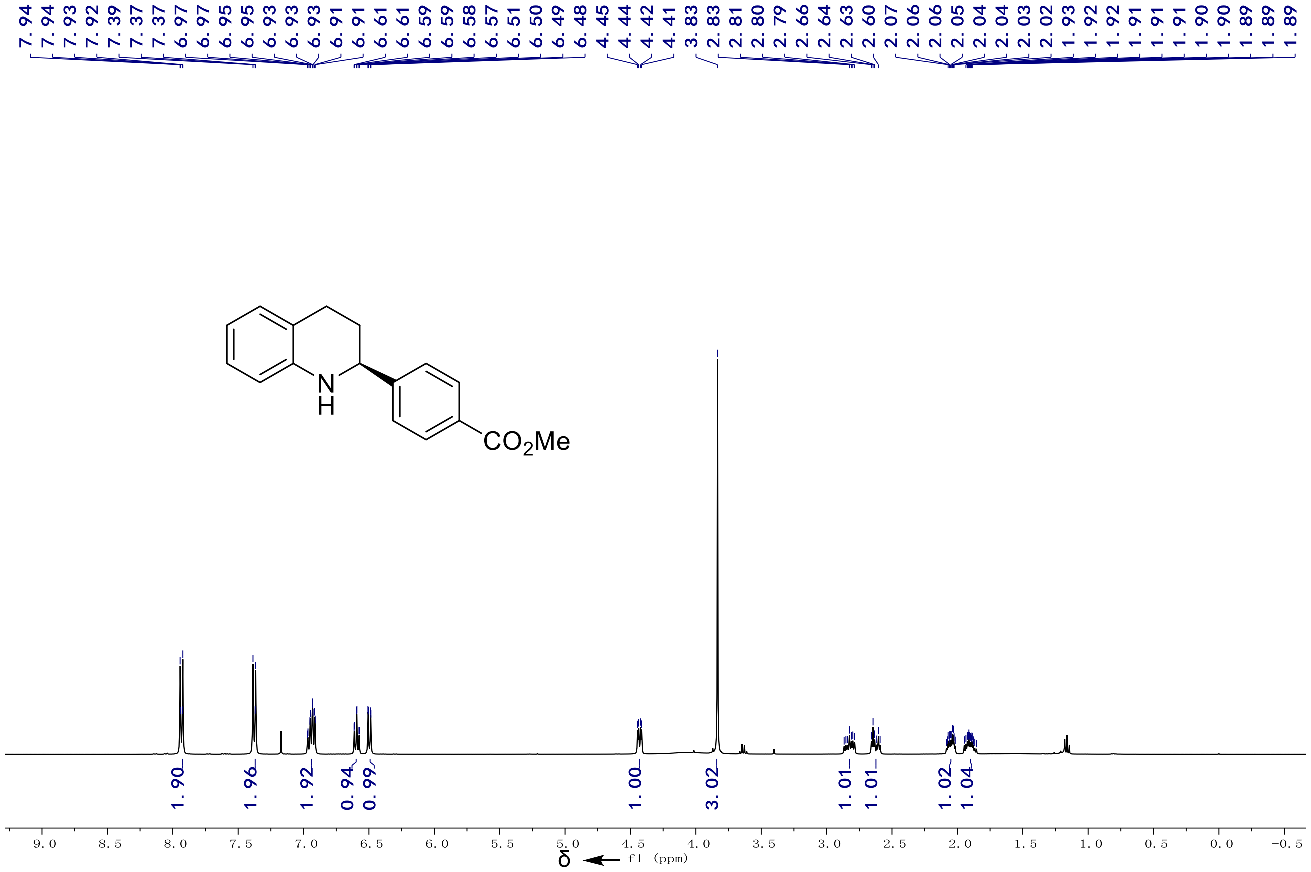

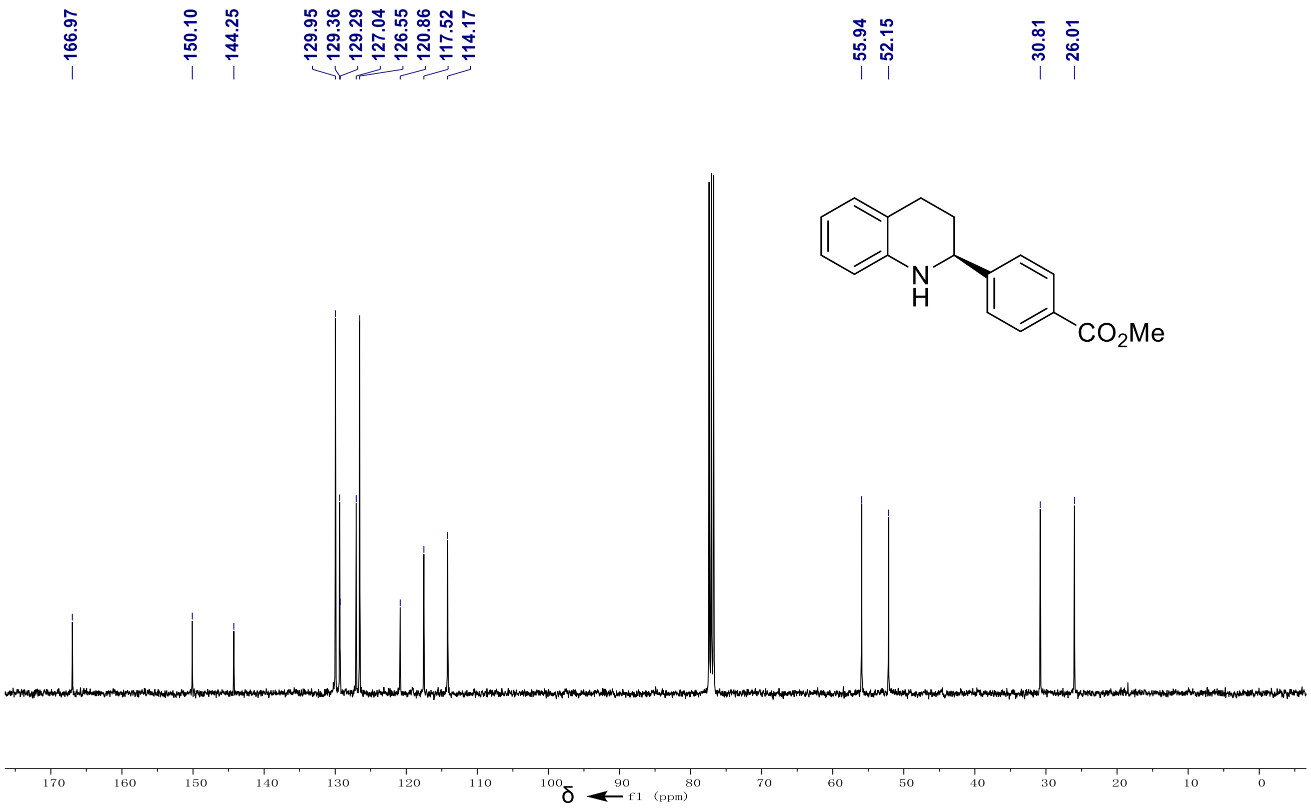

(*S*)-3g: (S)-2-(p-tolyl)-1,2,3,4-tetrahydroquinoline.

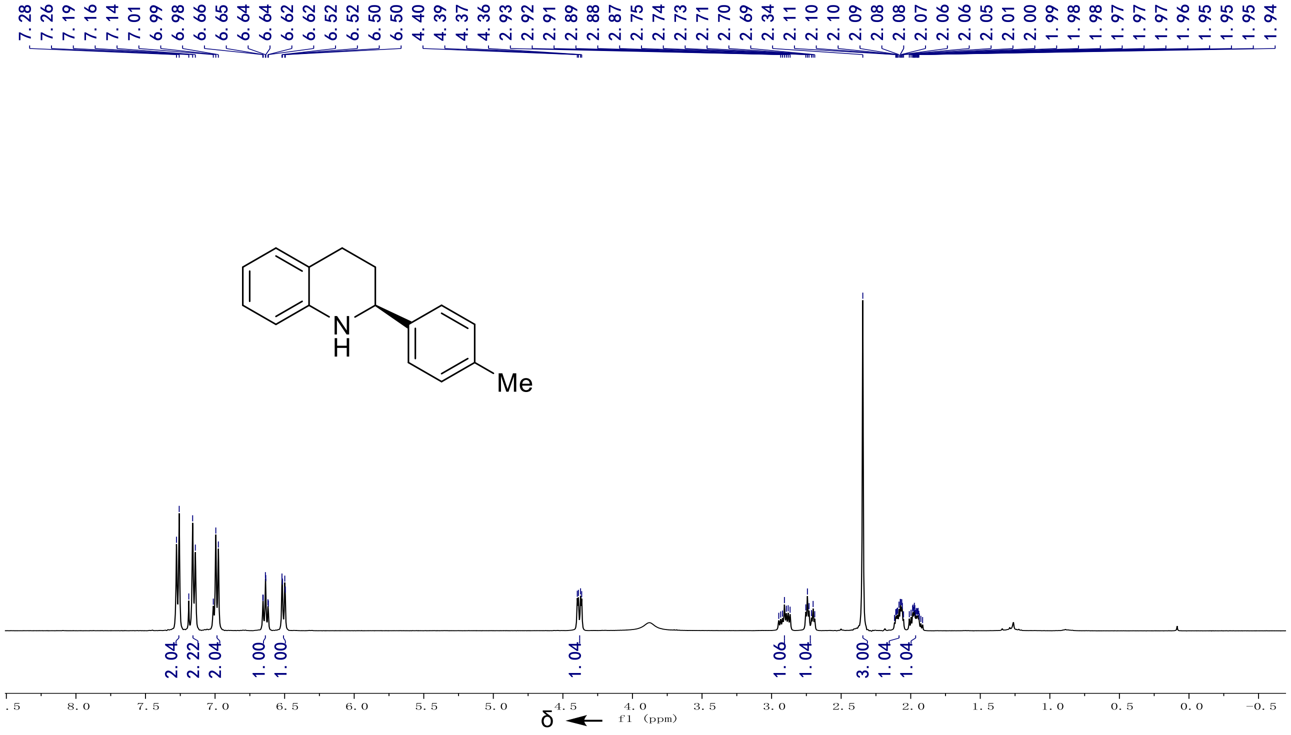

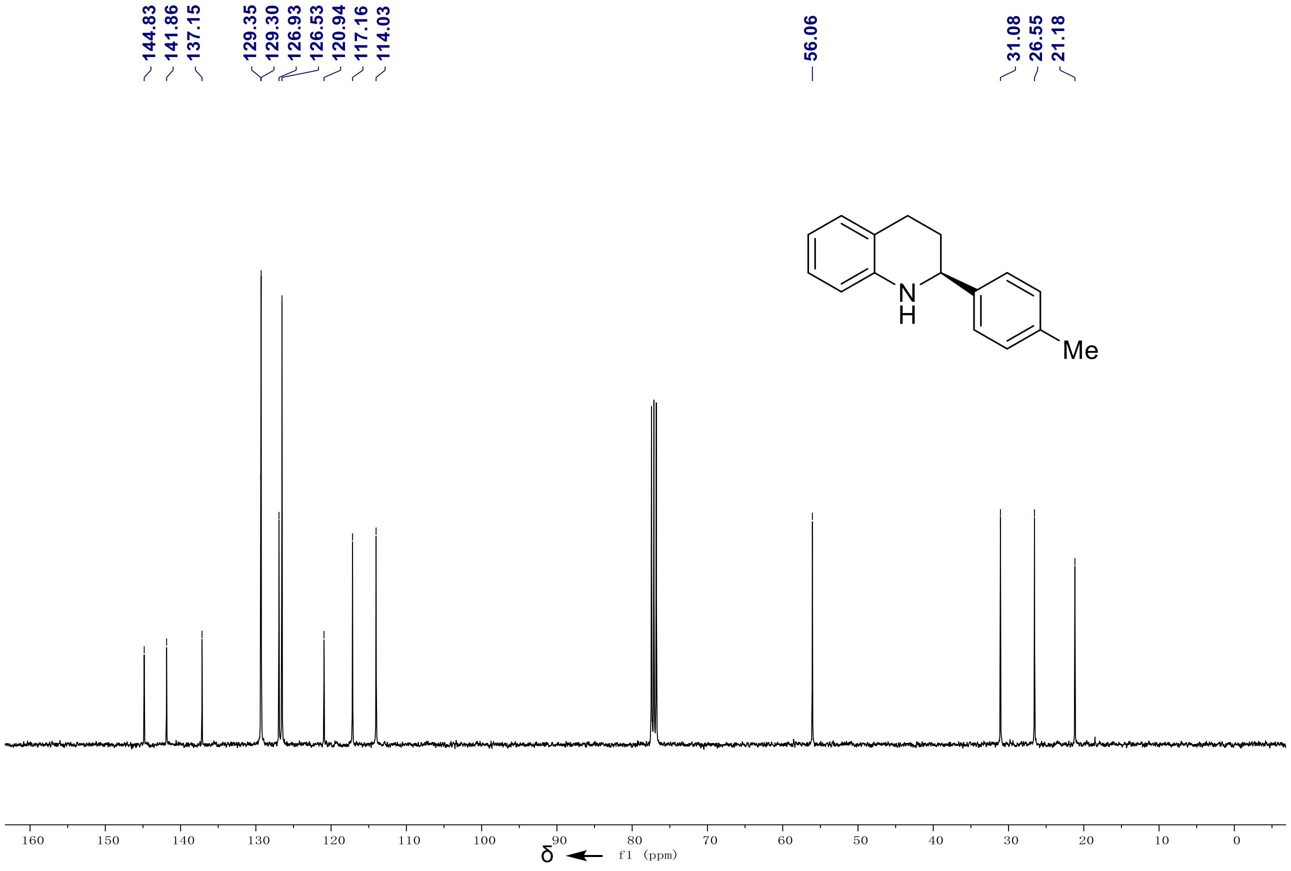

(*S*)-3h: (S)-2-(m-tolyl)-1,2,3,4-tetrahydroquinoline.

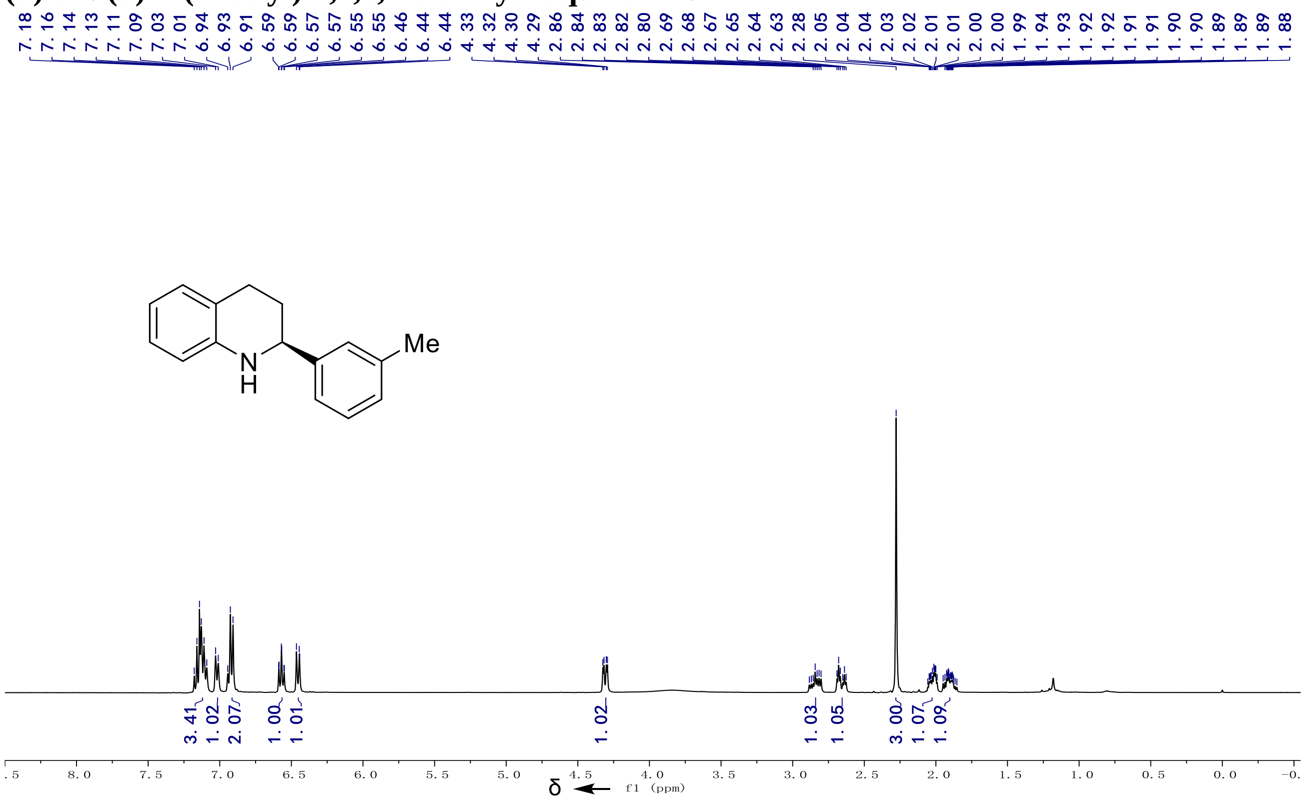

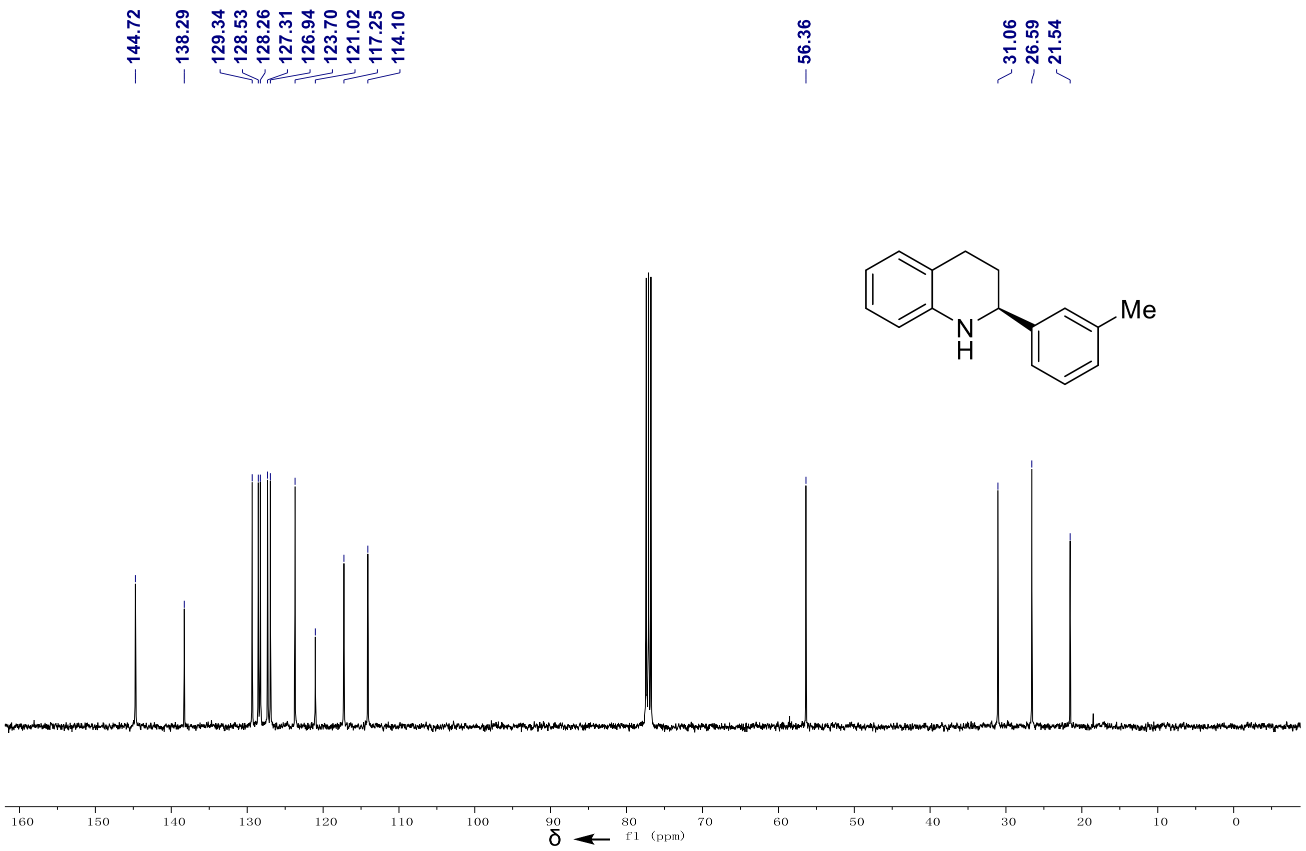

(*S*)-3i: (S)-2-(4-methoxyphenyl)-1,2,3,4-tetrahydroquinoline.

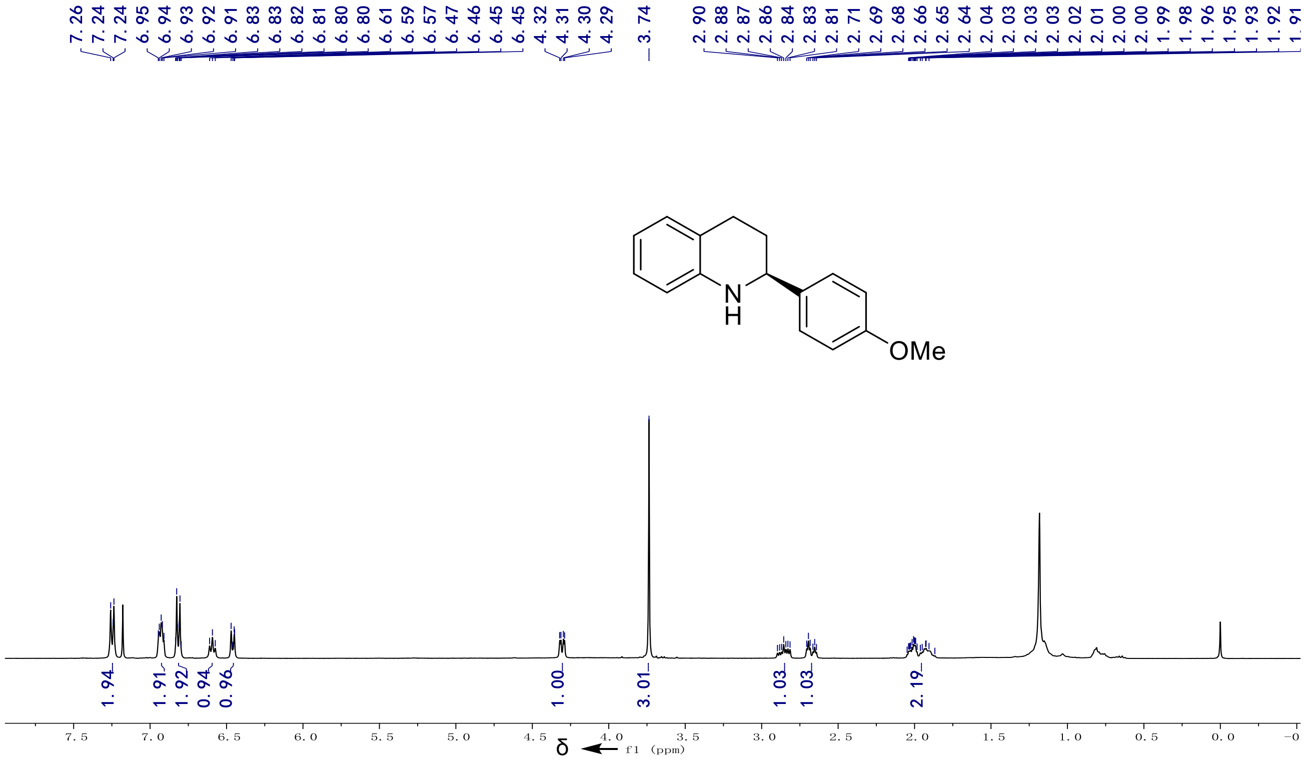

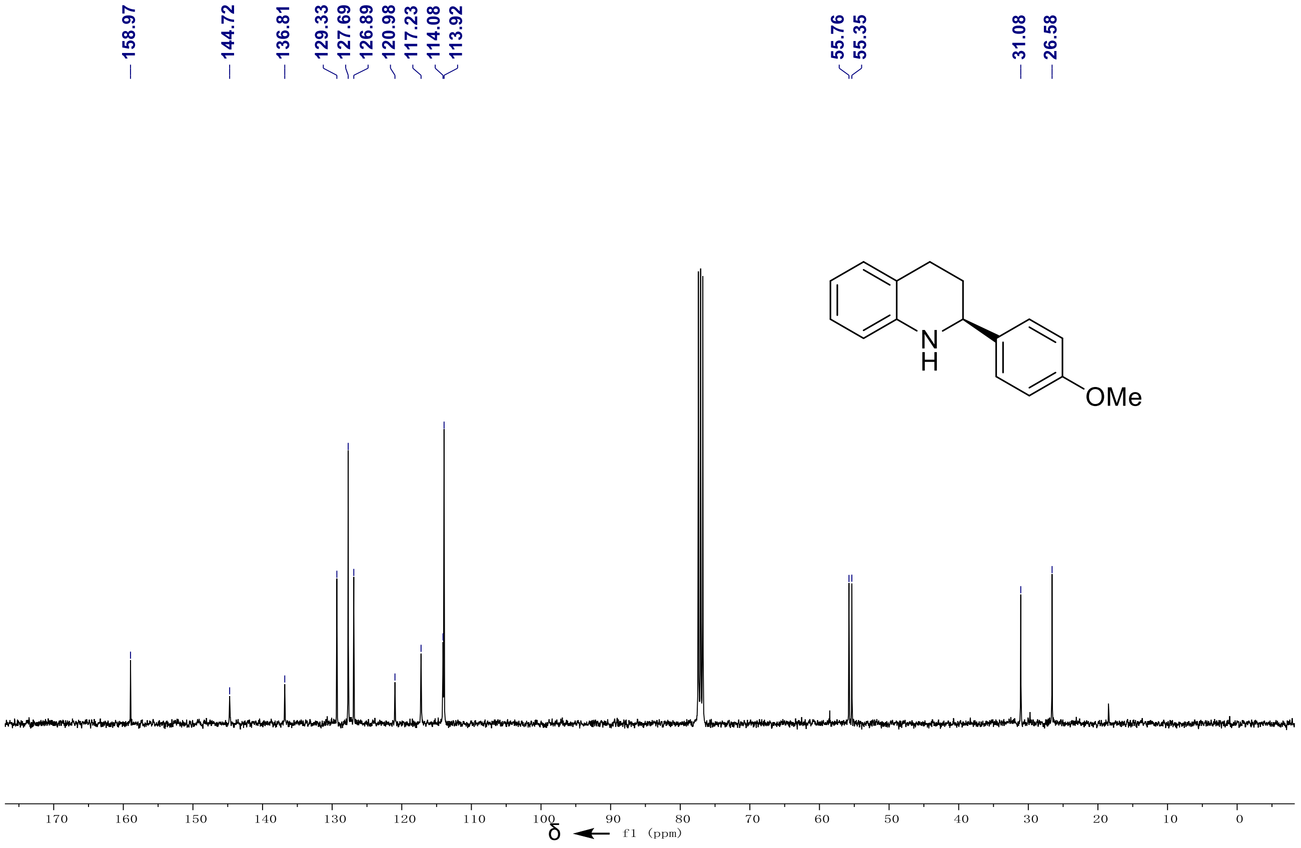

(*S*)-3j: (S)-2-(4-(methylthio)phenyl)-1,2,3,4-tetrahydroquinoline.

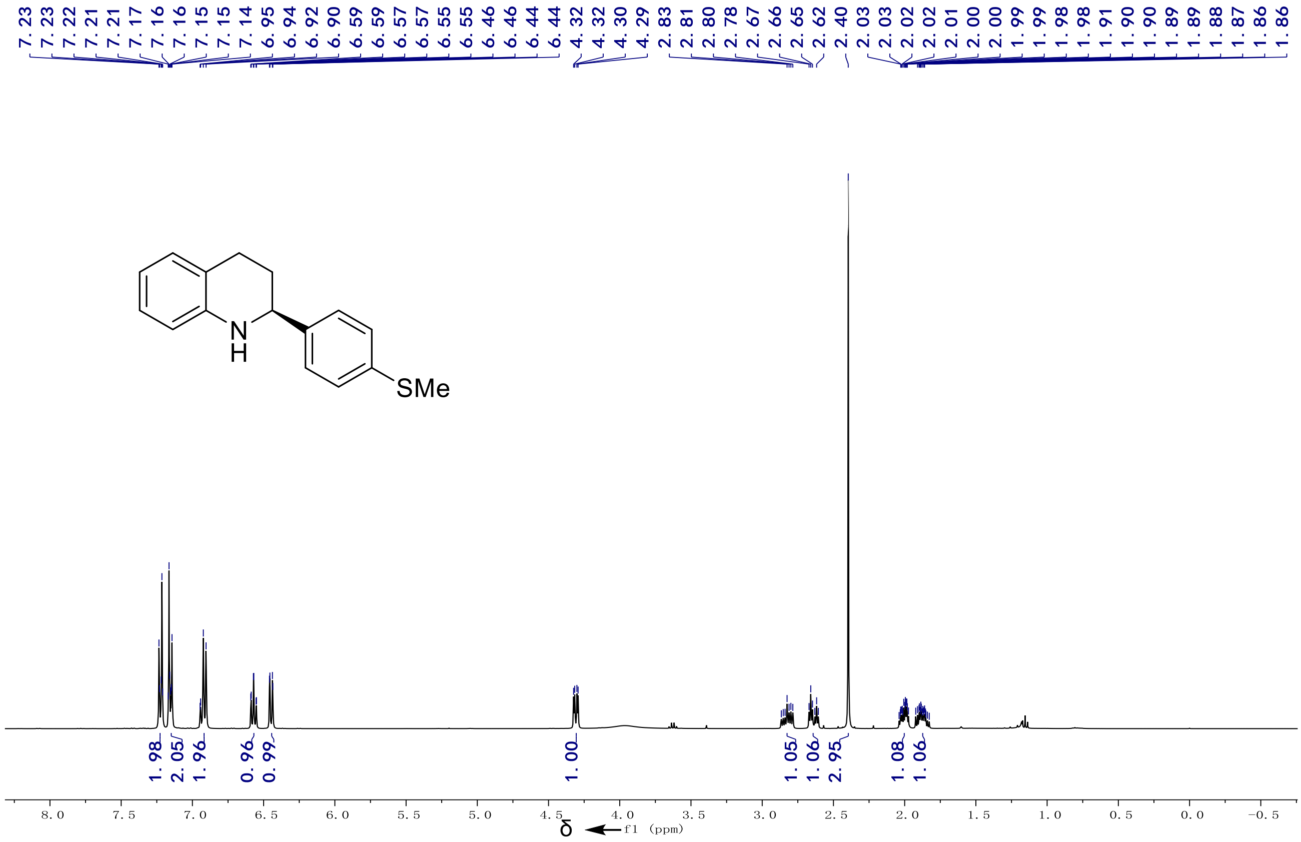

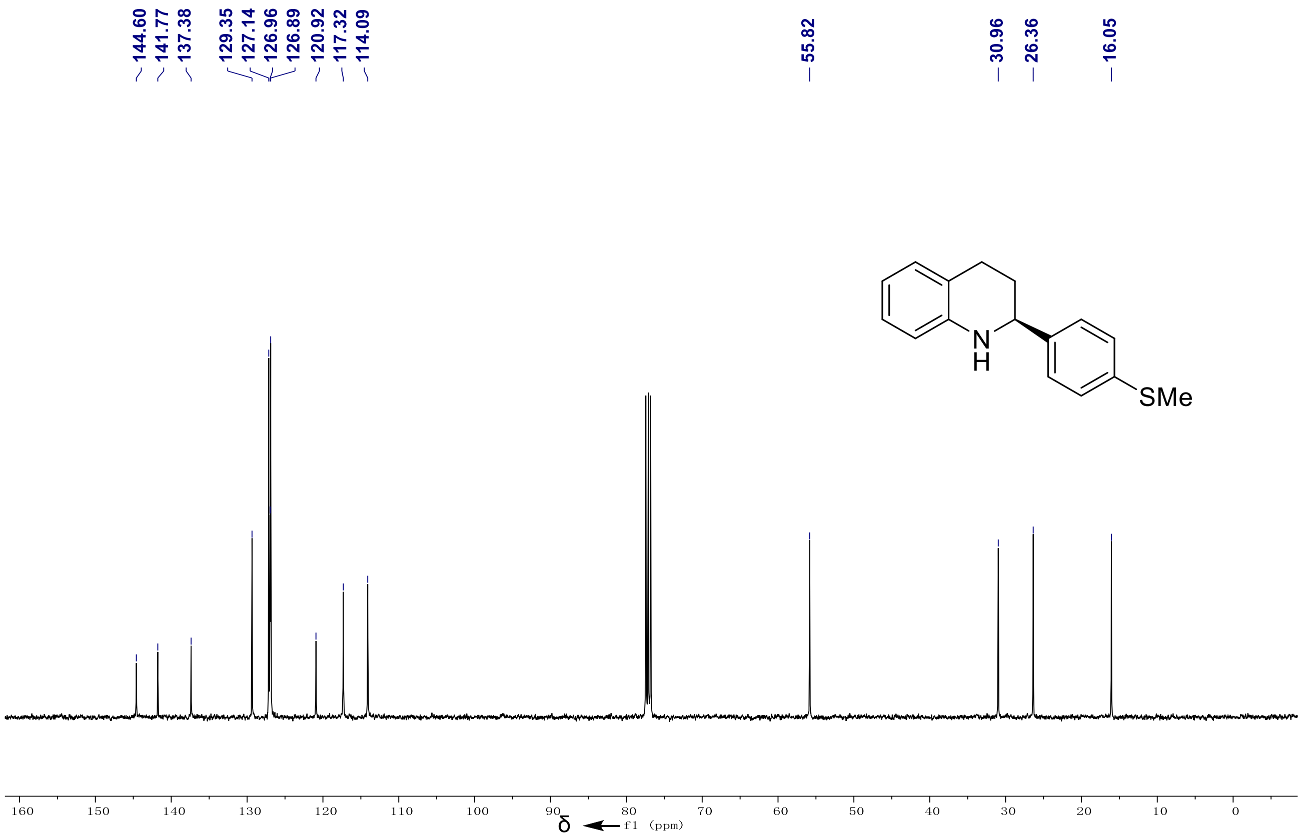

(*S*)-3k: (S)-2-(4-(trimethylsilyl)phenyl)-1,2,3,4-tetrahydroquinoline.

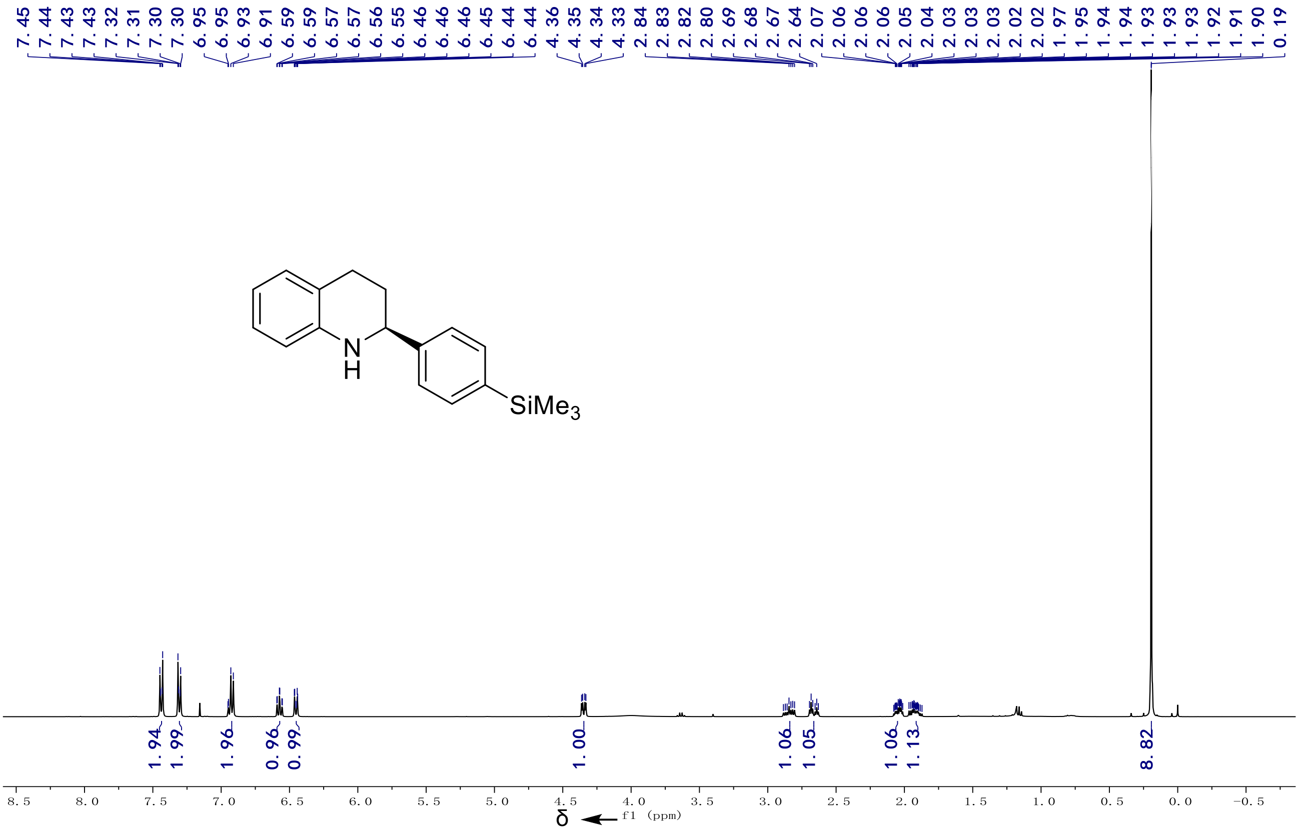

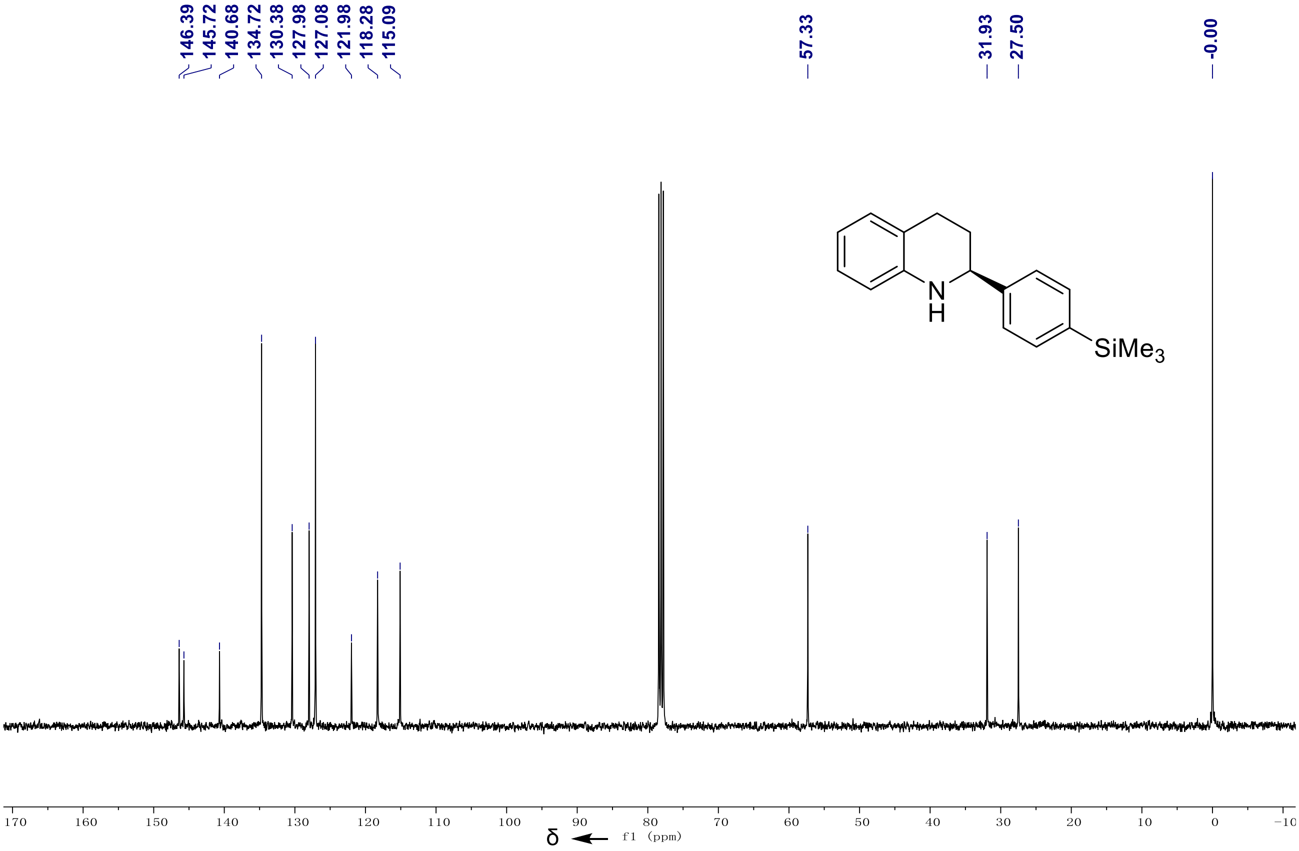

(*S*)-3l: (S)-2-([1,1'-biphenyl]-4-yl)-1,2,3,4-tetrahydroquinoline.

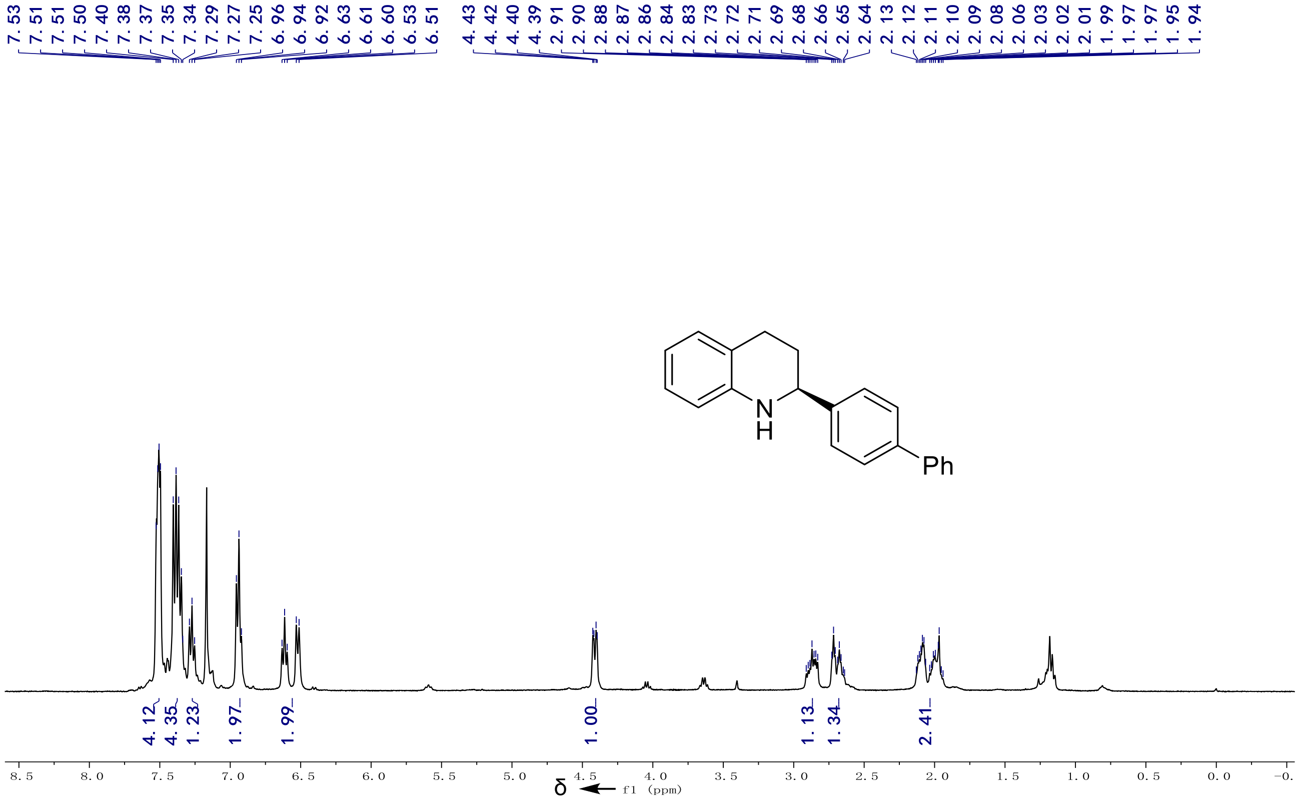

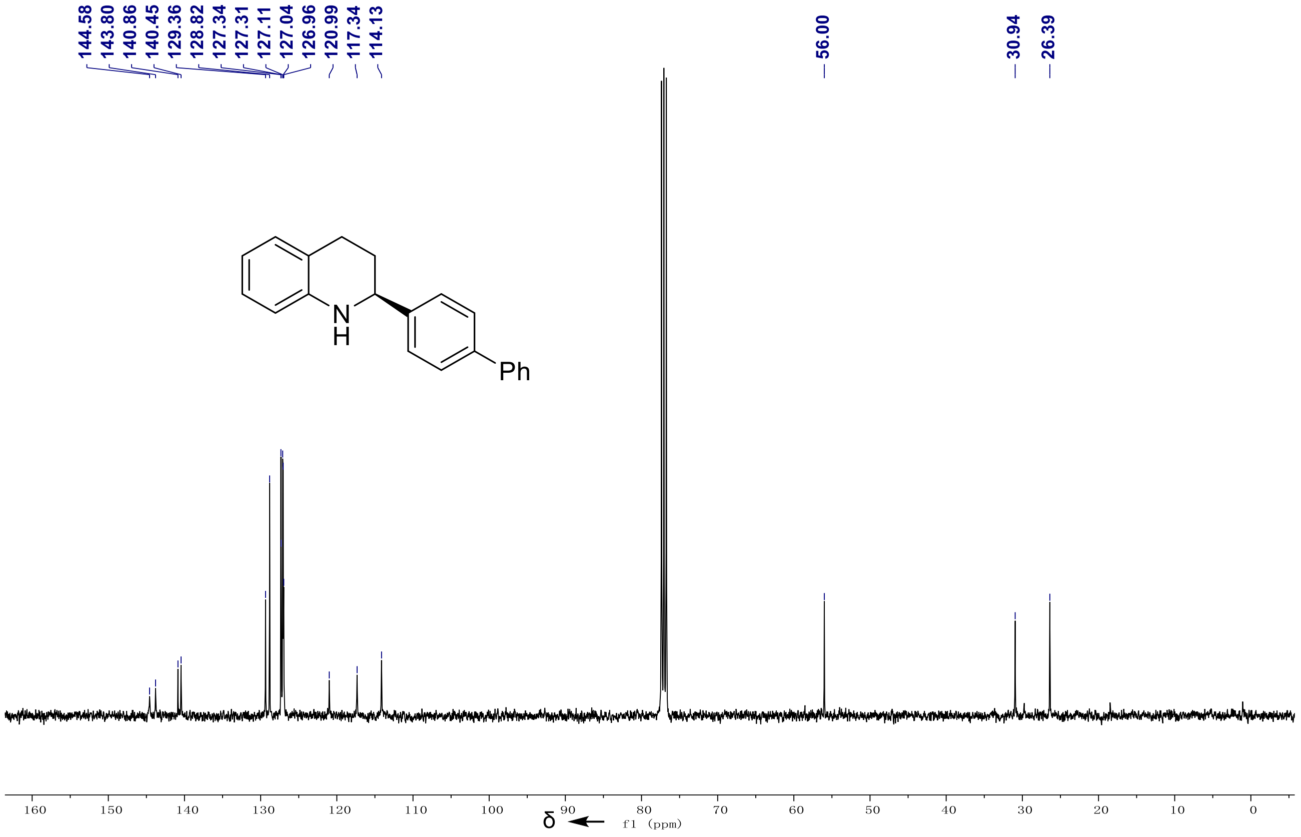

(*S*)-3m: (S)-2-(thiophen-2-yl)-1,2,3,4-tetrahydroquinoline.

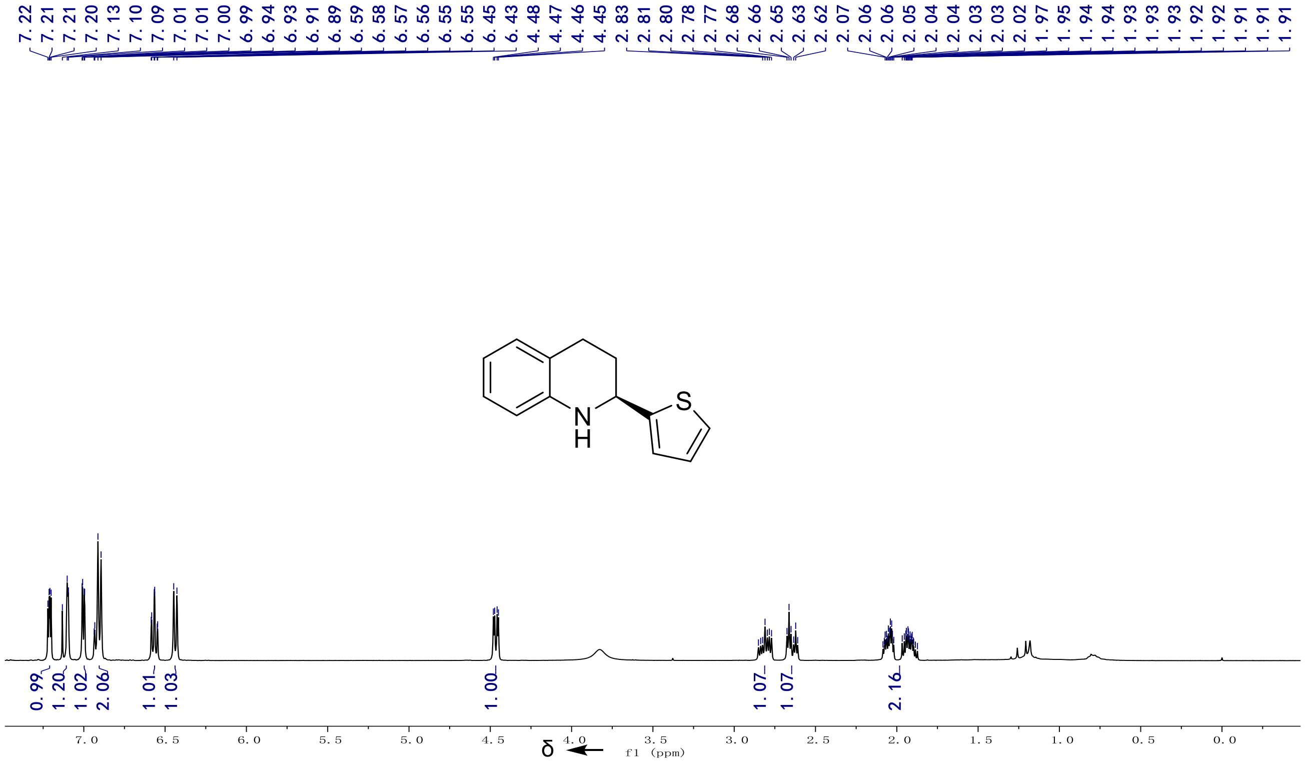

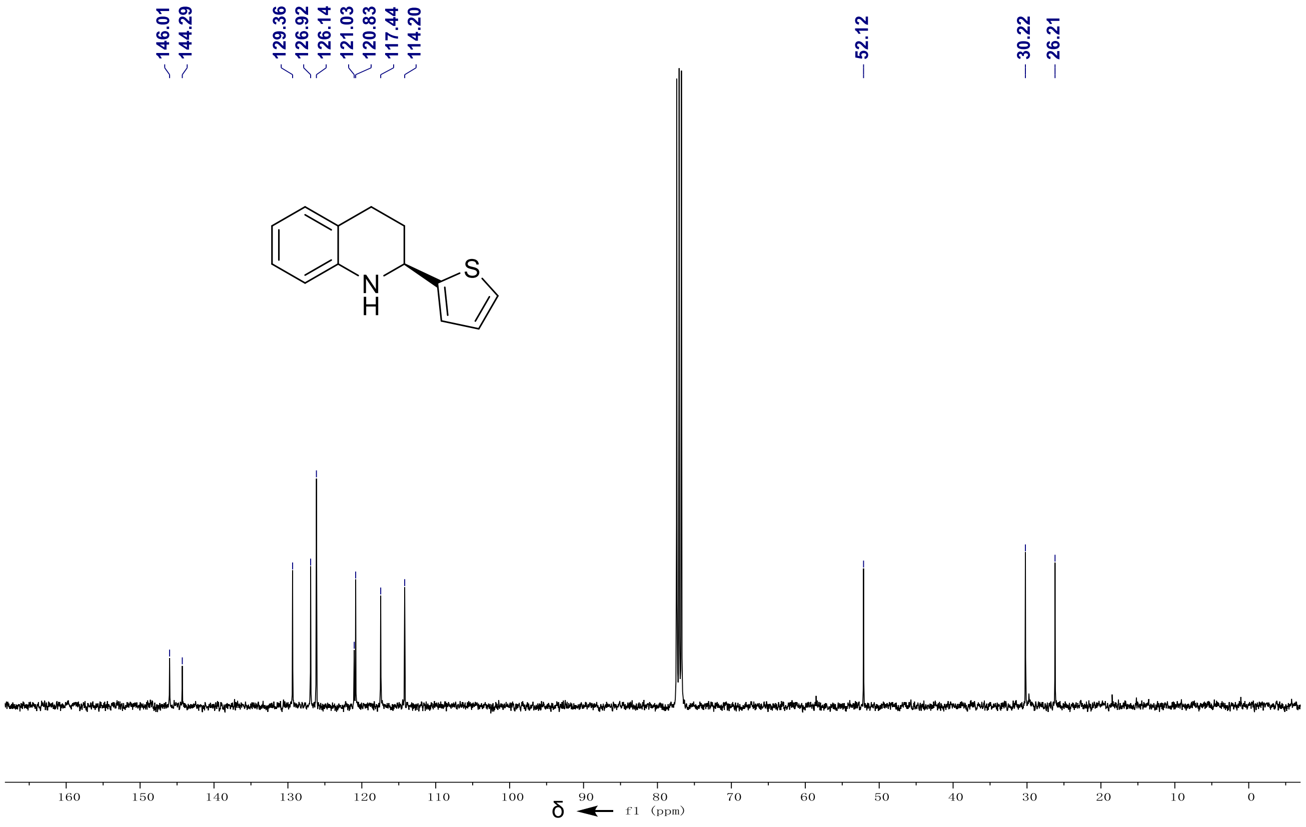

(*S*)-3n: (S)-6-fluoro-2-phenyl-1,2,3,4-tetrahydroquinoline.

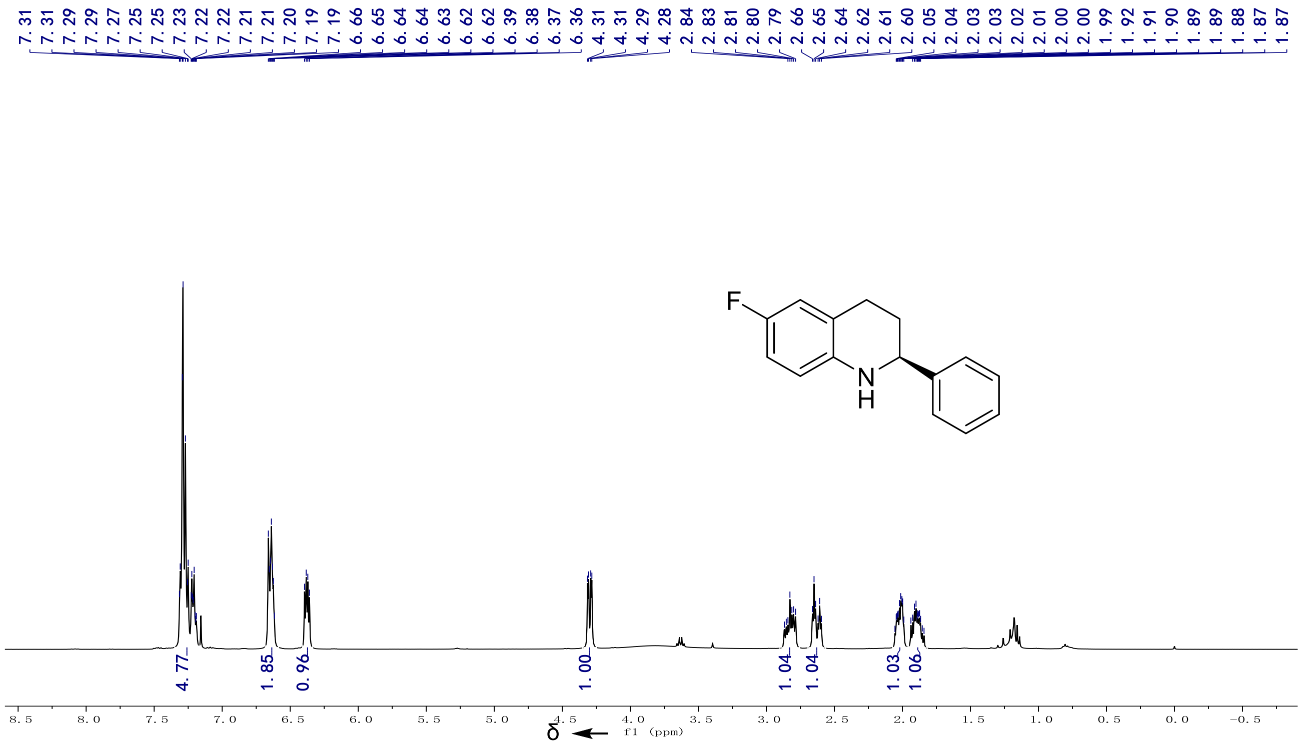

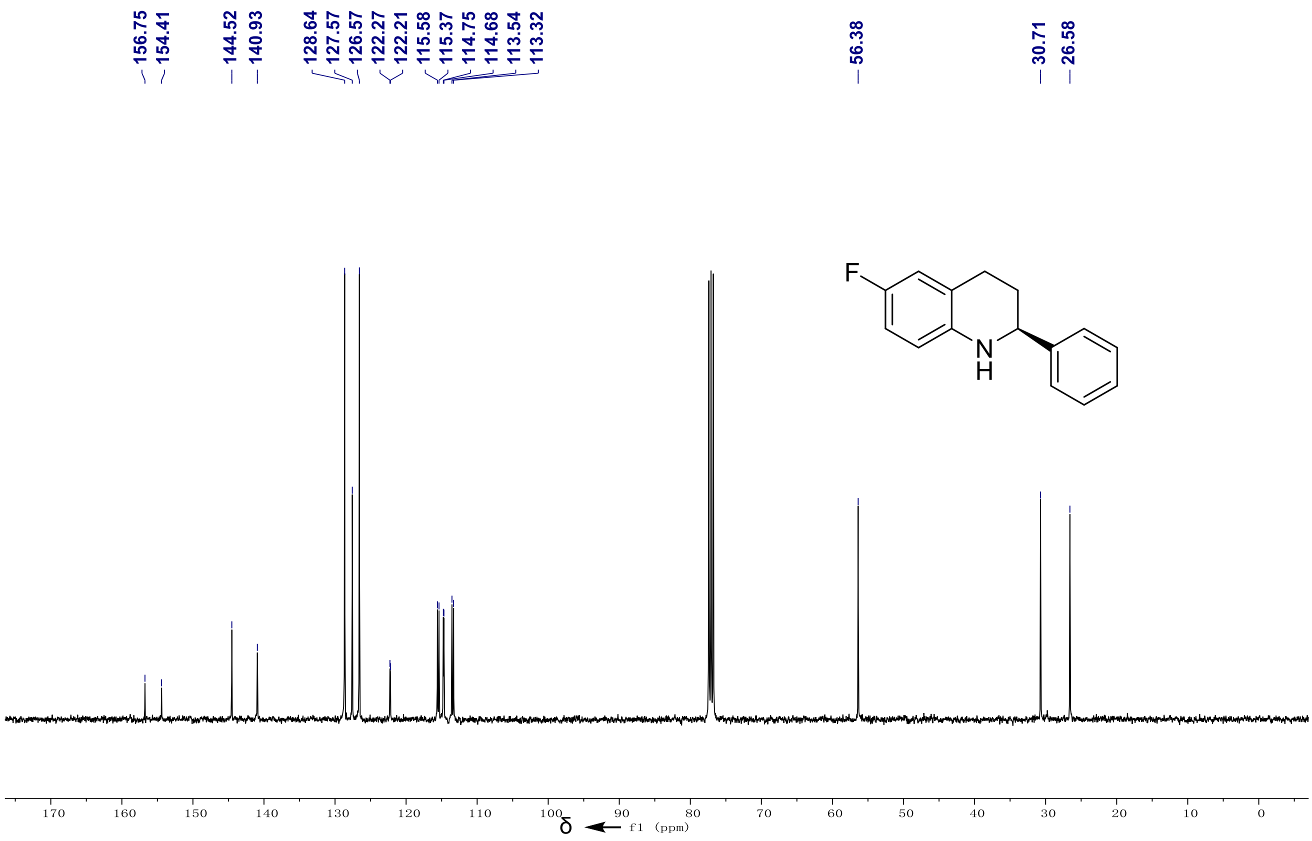

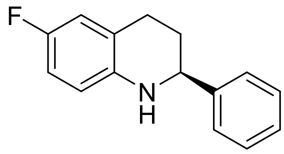

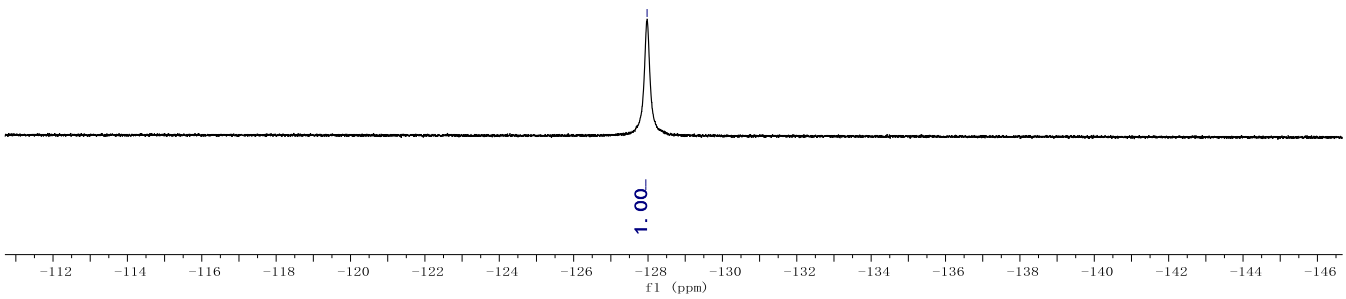

-127.97
1.00
-112
-114
-116
-118
-120
-122
-124
-126
-128
-130
-132
-134
-136
-138
-140
-142
-144
-146
f1 (ppm)

(*S*)-3o：(S)-6-bromo-2-phenyl-1,2,3,4-tetrahydroquinoline.

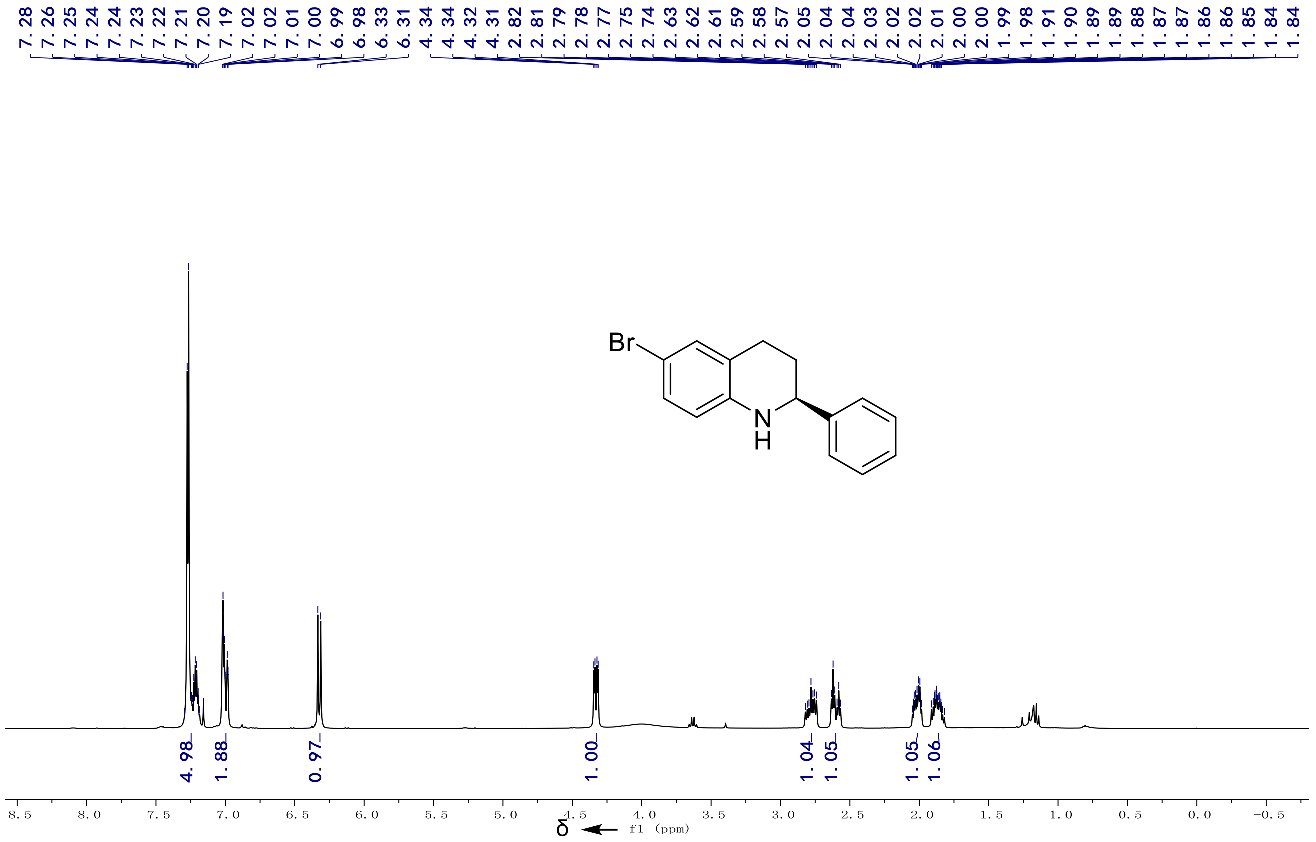

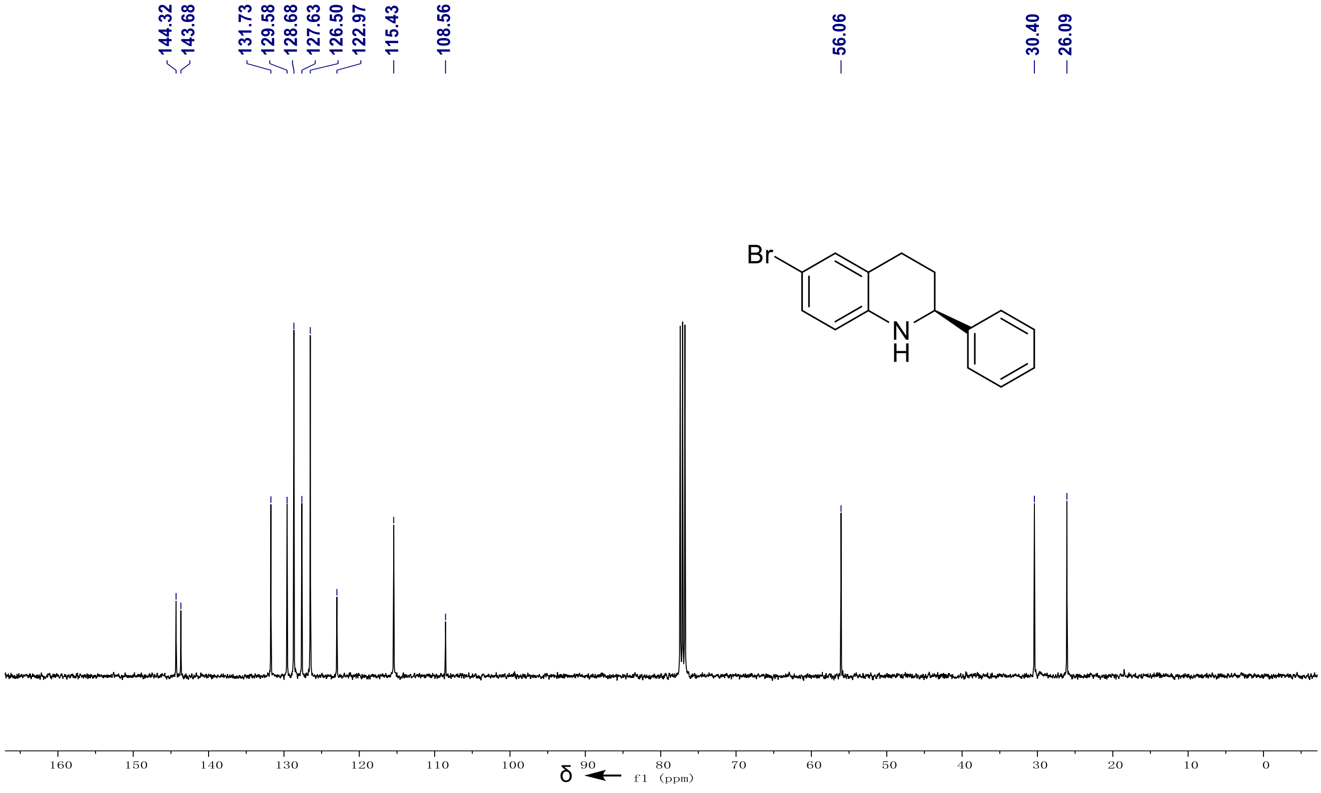

(*S*)-3p: (S)-6-methyl-2-phenyl-1,2,3,4-tetrahydroquinoline.

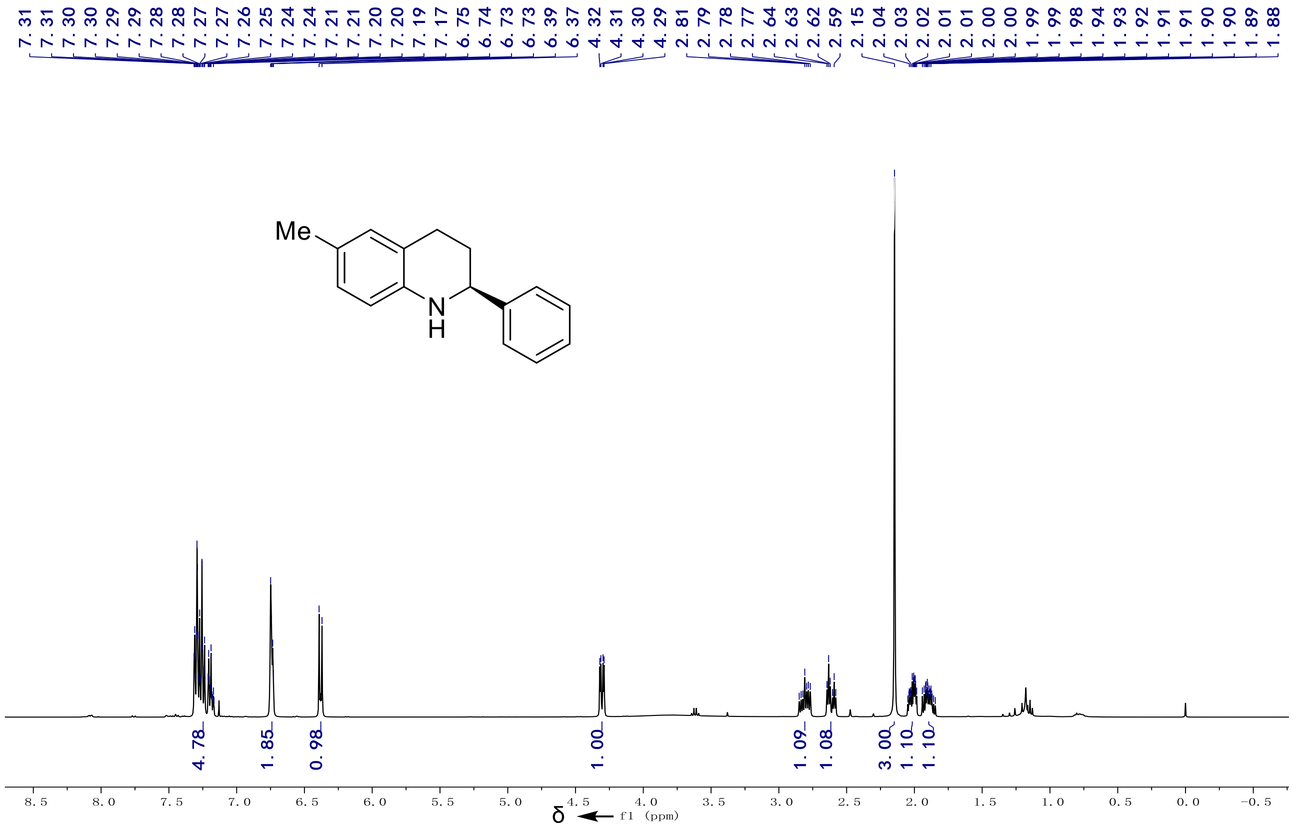

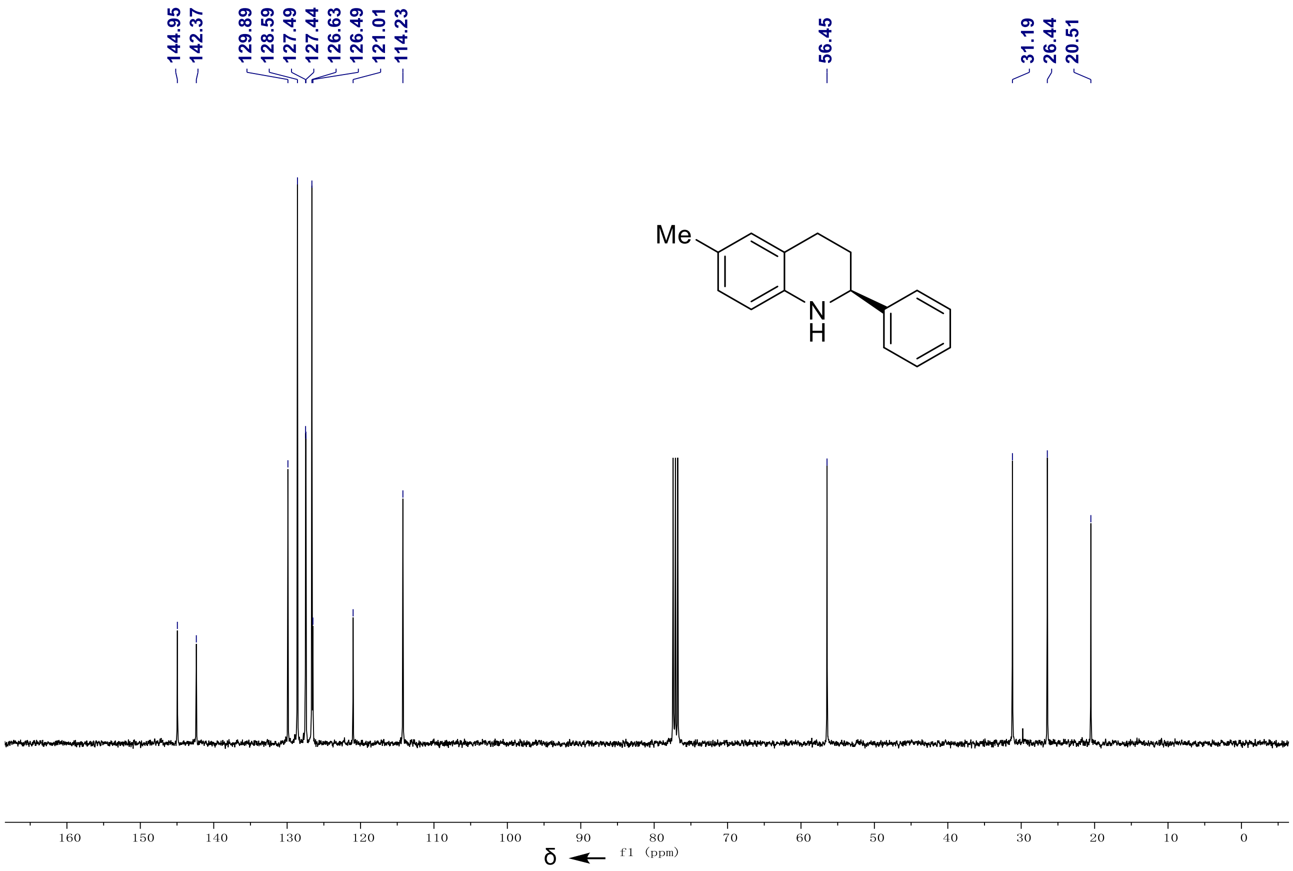

(*S*)-3q: (S)-6-methoxy-2-phenyl-1,2,3,4-tetrahydroquinoline.

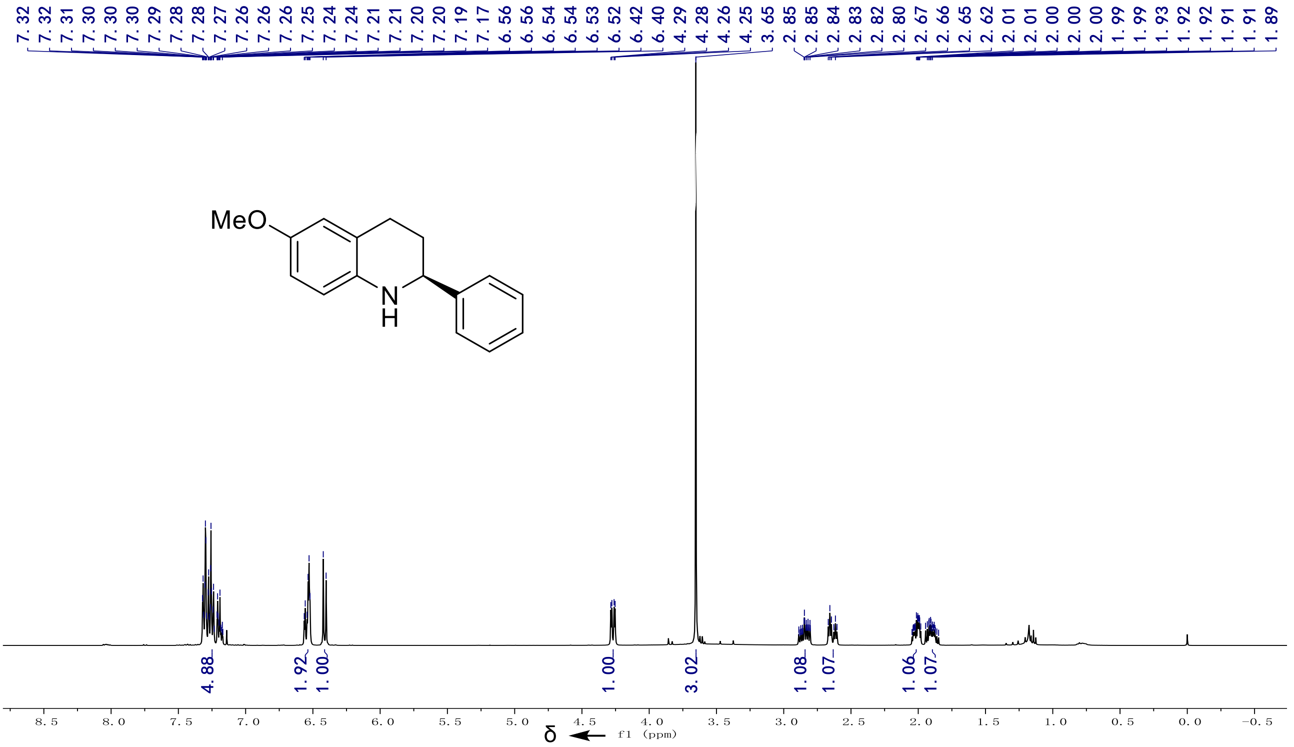

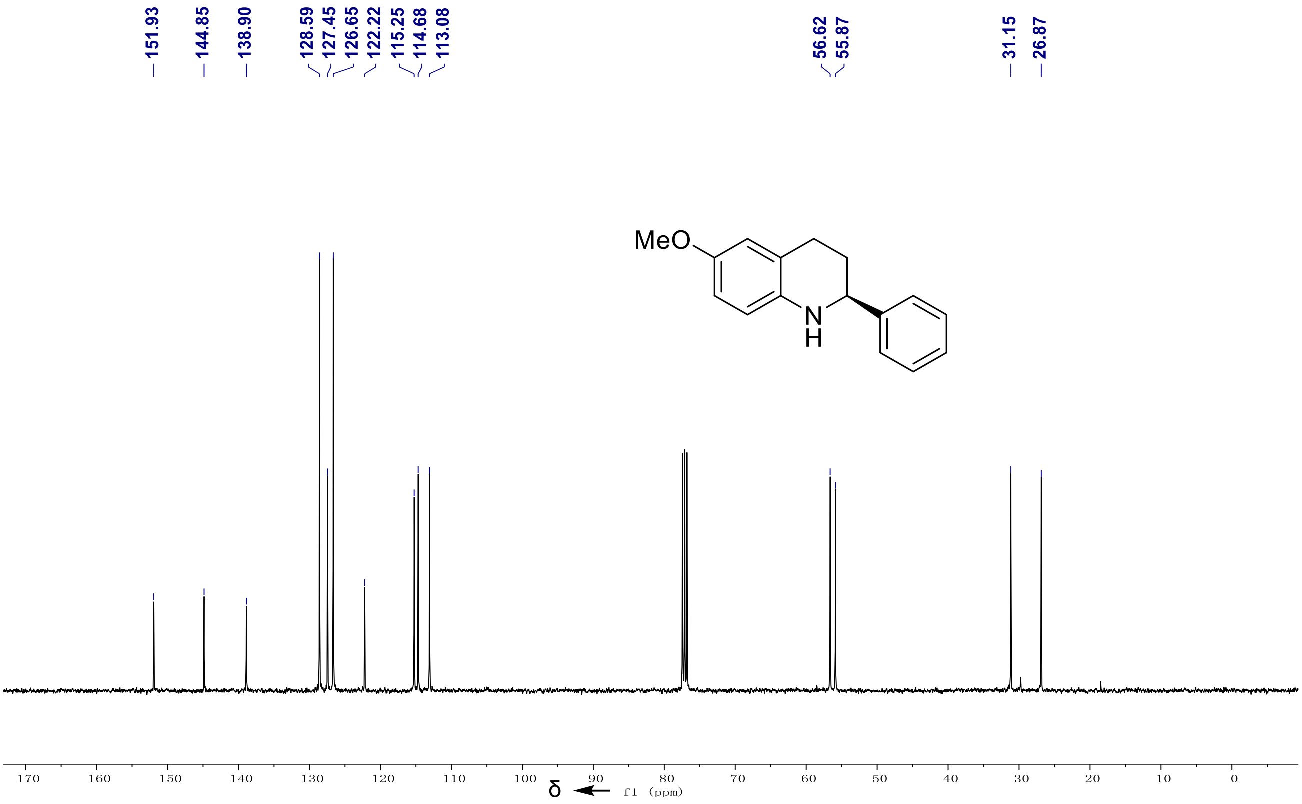

(*S*)-3r: methyl (S)-4-(6-methoxy-1,2,3,4-tetrahydroquinolin-2-yl)benzoate.

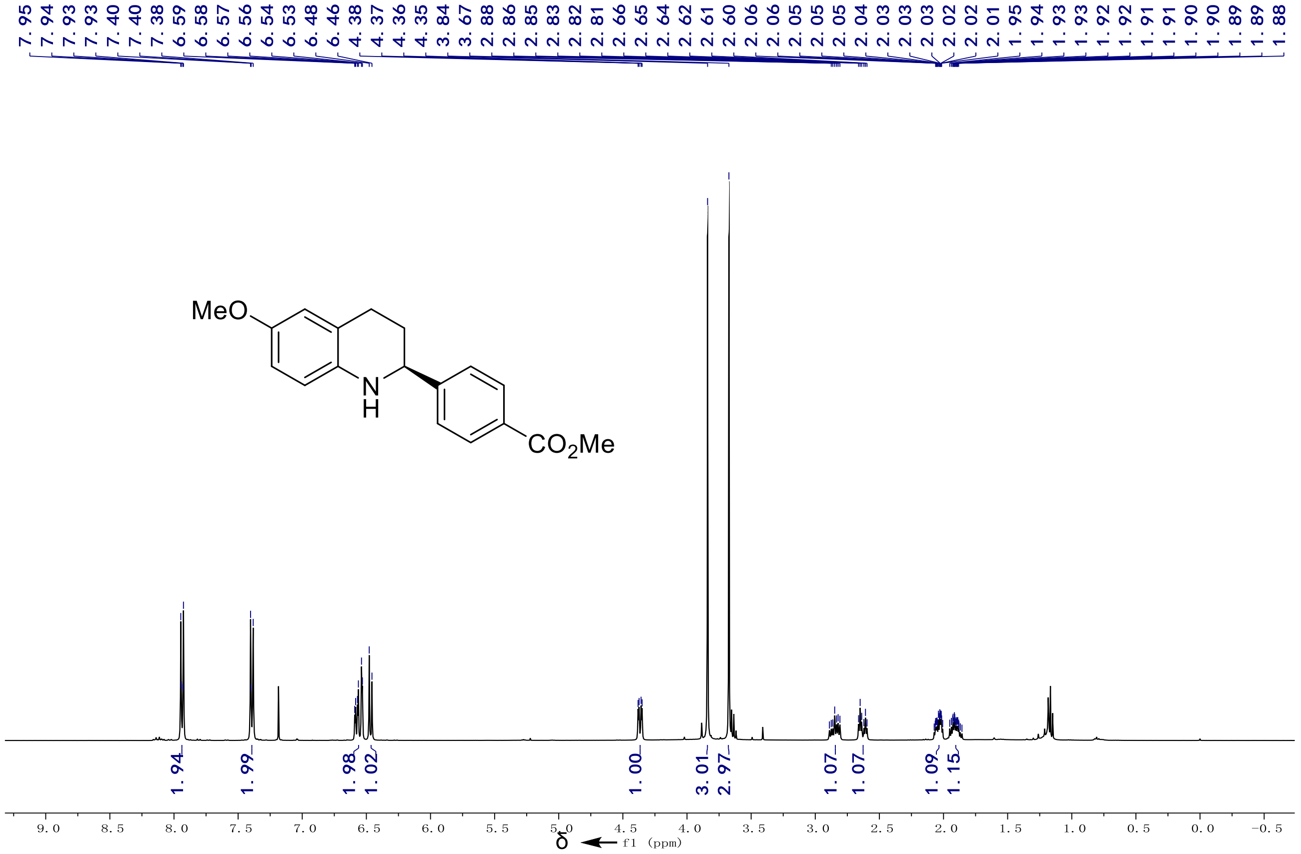

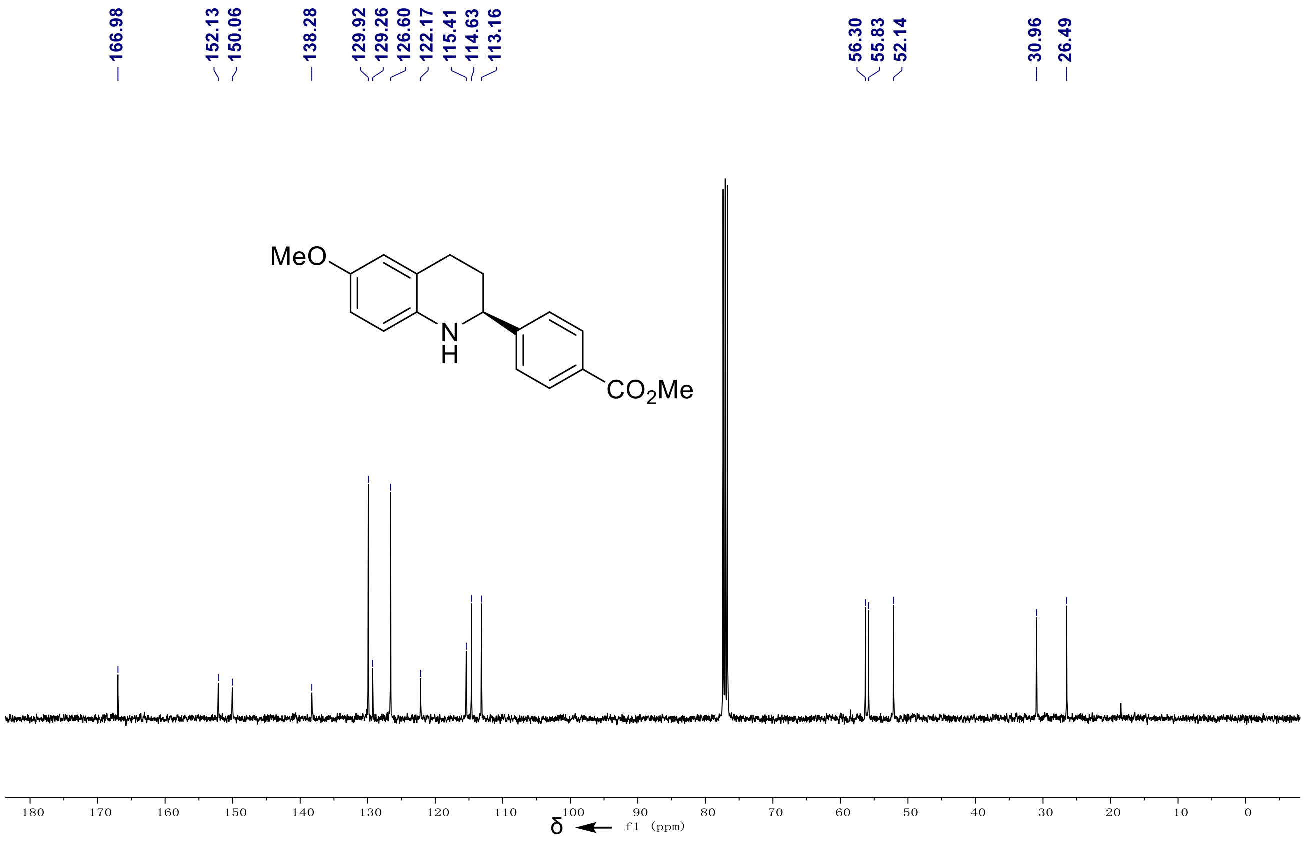

Supplement: SC-014-D2SC06340A-s001 [file SC-014-D2SC06340A-s001.pdf]
